# Targeting CD3L1-NRP2 disarms myeloid-driven tumor immune evasion

**DOI:** 10.1038/s44321-026-00451-3

**Published:** 2026-05-15

**Authors:** Shouyan Deng, Yuan Fang, Jieyuan Xue, Rui Wang, Xiaolin Zhou, Chushu Li, Ning Li, Shuhang Wang, Yibo Zhang, Huanbin Wang, Jianghong Yu, Jie Xu

**Affiliations:** 1 School of Pharmacy, Shanghai Key Laboratory of Oncology Target Discovery and Antibody Drug Development, Zhongshan Hospital, Fudan University, Shanghai, China; 2 Clinical Trials Center, National Cancer Center/National Clinical Research Center for Cancer/Cancer Hospital, Chinese Academy of Medical Sciences and Peking Union Medical College, Beijing, China; 3 BioTroy Therapeutics, Shanghai, China; 4 Department of Gastroenterology and Fujian Institute of Digestive Disease, Fujian Medical University Union Hospital, Fuzhou, China; 5 Shanghai Institute of Materia Medica, Chinese Academy of Sciences, Shanghai, China

**Keywords:** Cancer, Immunology

## Abstract

CD3 ligand 1 (CD3L1, ITPRIPL1), an emerging immune checkpoint, sustains immune privilege in the testis and facilitates tumor immune evasion. Targeting CD3L1 with a monoclonal antibody demonstrates potent antitumor activity in preclinical models and spontaneous tumors in companion animals. In an ongoing clinical trial, anti-CD3L1 therapy unexpectedly activated tumor-associated macrophages (TAMs) within the tumor microenvironment (TME), surpassing its anticipated role in T-cell reactivation. Mechanistic studies identified neuropilin-2 (NRP2) as the primary receptor on macrophages and uncovered the CD3L1-NRP2 axis as a critical driver of immunosuppressive M2 TAM polarization. Strikingly, in T-cell-deficient osteosarcoma models, anti-CD3L1 treatment reprogrammed TAMs toward an anti-tumor M1 phenotype, suppressing tumor progression. Clinical data corroborated these findings, revealing profound TME remodeling in advanced solid tumors. Our results elucidate a dual role for CD3L1 in immune evasion, mediated through both T-cell suppression and macrophage polarization, and highlight anti-CD3L1 as a multifaceted therapeutic strategy that enhances antigen presentation via TAM modulation.

## Synopsis

CD3L1 targeting is proposed as a bifunctional strategy to overcome immune evasion, offering a promising avenue for the treatment of immunotherapy-resistant malignancies and guiding the clinical development of anti-CD3L1 therapies.

- Targeting CD3L1 disrupts its interaction with NRP2, reprogramming TAMs toward an immunostimulatory phenotype while revitalizing T-cell effector functions, even in T-cell sparse tumors like osteosarcoma.
- In an ongoing Phase I trial, anti-CD3L1 monotherapy induced TME remodeling in PD-1 refractory tumors, with concomitant T-cell activation and macrophage infiltration correlating with therapeutic efficacy.
- Integrated multi-omics and pan-cancer analyses reveal that CD3L1 overexpression predicts poor prognosis and is tightly linked to M2 TAM dominance across solid tumors.

## Abstract (web summary)

CD3L1 targeting is proposed as a bifunctional strategy to overcome immune evasion, offering a promising avenue for the treatment of immunotherapy-resistant malignancies and guiding the clinical development of anti-CD3L1 therapies.

## Graphical abstract

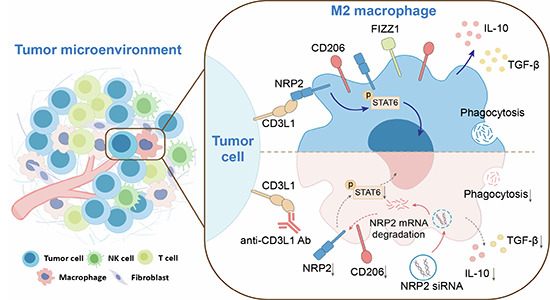

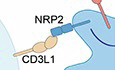

The paper explained **Problem**

Incomplete characterization of the immune evasion mechanisms within the tumor microenvironment (TME) continues to limit the efficacy of immune checkpoint blockade (ICB) therapies.

**Results**

This study identifies CD3L1 (ITPRIPL1) as a dual immunoregulatory molecule that facilitates tumor immune evasion by modulating both T cell and macrophage functions. Targeting CD3L1 with a monoclonal antibody demonstrated potent antitumor activity in preclinical models and in patients, unexpectedly activating TAMs within the TME beyond its anticipated role in T-cell reactivation. Mechanistic studies revealed that CD3L1 interacts with neuropilin-2 (NRP2) on macrophages, driving the polarization of immunosuppressive TAMs. In T-cell-deficient osteosarcoma models, anti-CD3L1 treatment reprogrammed TAMs toward an anti-tumor phenotype, suppressing tumor progression. Clinical data corroborated these findings, showing deep TME remodeling in advanced solid tumors, with reduced immunosuppressive TAM infiltration and enhanced T cell activation. Furthermore, the modulation of TAMs by anti-CD3L1 was found to be Fc-independent, highlighting the direct role of CD3L1-NRP2 interaction in macrophage polarization.

**Impact**

These findings contribute to a deeper understanding of the pivotal role of CD3L1 in immune evasion within the TME and provide valuable insights for guiding anti-CD3L1 treatment in clinical trials.

## Introduction

The advent of immune checkpoint blockade (ICB) therapy has revolutionized cancer treatment (Sharma et al, 2023; Carlino et al, 2021), with anti-PD-L1/PD-1 therapies, a cornerstone of modern immunotherapy, demonstrating remarkable efficacy in diverse cancers (Cercek et al, 2022; Mirza et al, 2023). Nevertheless, persistent gaps in understanding the intricate immune evasion mechanisms within the tumor microenvironment (TME) continue to constrain ICB effectiveness (Morad et al, 2022; Goc et al, 2021; Koikawa et al, 2021), a challenge that also applies to well-characterized targets such as PD-L1 and LAG-3. Notably, insights into PD-L1’s functions in macrophage polarization (Wei et al, 2021) and fungal binding (Li et al, 2024a) were uncovered years after PD-1/PD-L1 inhibitors had become standard first-line therapies. Likewise, FGL1, a novel LAG-3 ligand, was discovered only after its associated antibodies, Relatlimab and Favezelimab, had advanced to clinical trials (Wang et al, 2019). These examples underscore that advancing our comprehension of ICB targets remains both vital and an ever-evolving scientific priority.

In prior work, we identified ITPRIPL1 (CD3L1), a protein highly expressed in normal testicular tissue and aberrantly overexpressed across multiple human cancers, as a suppressor of TCR-CD3 signaling and T cell activation (Deng et al, 2024). Building on this, we engineered a humanized IgG1 anti-CD3L1 monoclonal antibody with high specificity for human CD3L1, demonstrating potent ADCC/CDC activity and robust anti-tumor efficacy in preclinical models (Deng et al, 2023). Early findings from our finished Ia and ongoing Ib clinical trial (NCT06404905) corroborate these results, revealing enhanced T cell activation within the tumor microenvironment (TME) following anti-CD3L1 therapy. Intriguingly, treatment also significantly increased macrophage infiltration while reducing M2 tumor-associated macrophages (TAMs)—a population known to drive immunosuppression via factors like TGF-β and IL-10 (Lu et al, 2025). These cytokines not only foster immunosuppressive cells such as MDSCs and Tregs but also reinforce the TME’s immunosuppressive landscape (Komohara et al, 2016; Ngambenjawong et al, 2017). Despite progress, the regulatory networks governing TAM polarization—particularly the shift between pro-inflammatory M1 and immunosuppressive M2 phenotypes—remain poorly defined. While M1 TAMs secrete immunostimulatory mediators (e.g., IL-1β, IL-12, TNF-α) that recruit cytotoxic effectors like CTLs and NK cells (Xia et al, 2020; Gunassekaran et al, 2021), therapeutic strategies targeting M2 TAM depletion have shown promise in rebalancing the TME (Yang et al, 2022a; Cao et al, 2021; Billerhart et al, 2021; Han et al, 2023). Nevertheless, the mechanistic basis underlying the predominance of M2 TAMs in solid tumors remains unresolved, pointing to unidentified molecular pathways that may govern TAM plasticity and phenotypic fate.

The unanticipated dual functionality of anti-CD3L1—modulating both T cells and macrophages—motivated our exploration of CD3L1’s role in tumor-associated macrophage (TAM) biology. Combining receptor profiling and mechanistic analyses, we identified a CD3L1-NRP2 signaling axis that drives immunosuppressive M2 TAM polarization. In osteosarcoma, a tumor model marked by limited T cell infiltration, anti-CD3L1 treatment shifted TAMs toward an immunostimulatory phenotype, independent of T cell activation. These results delineate an unconventional mechanism of action for anti-CD3L1, establishing its potential as a bifunctional therapeutic strategy that concurrently amplifies T cell effector functions and disrupts M2 TAM-driven immunosuppression within the tumor microenvironment.

## Results

### Identification of a negative correlation between anti-CD3L1 treatment and M2 TAMs in solid tumor samples

**Figure 1:**
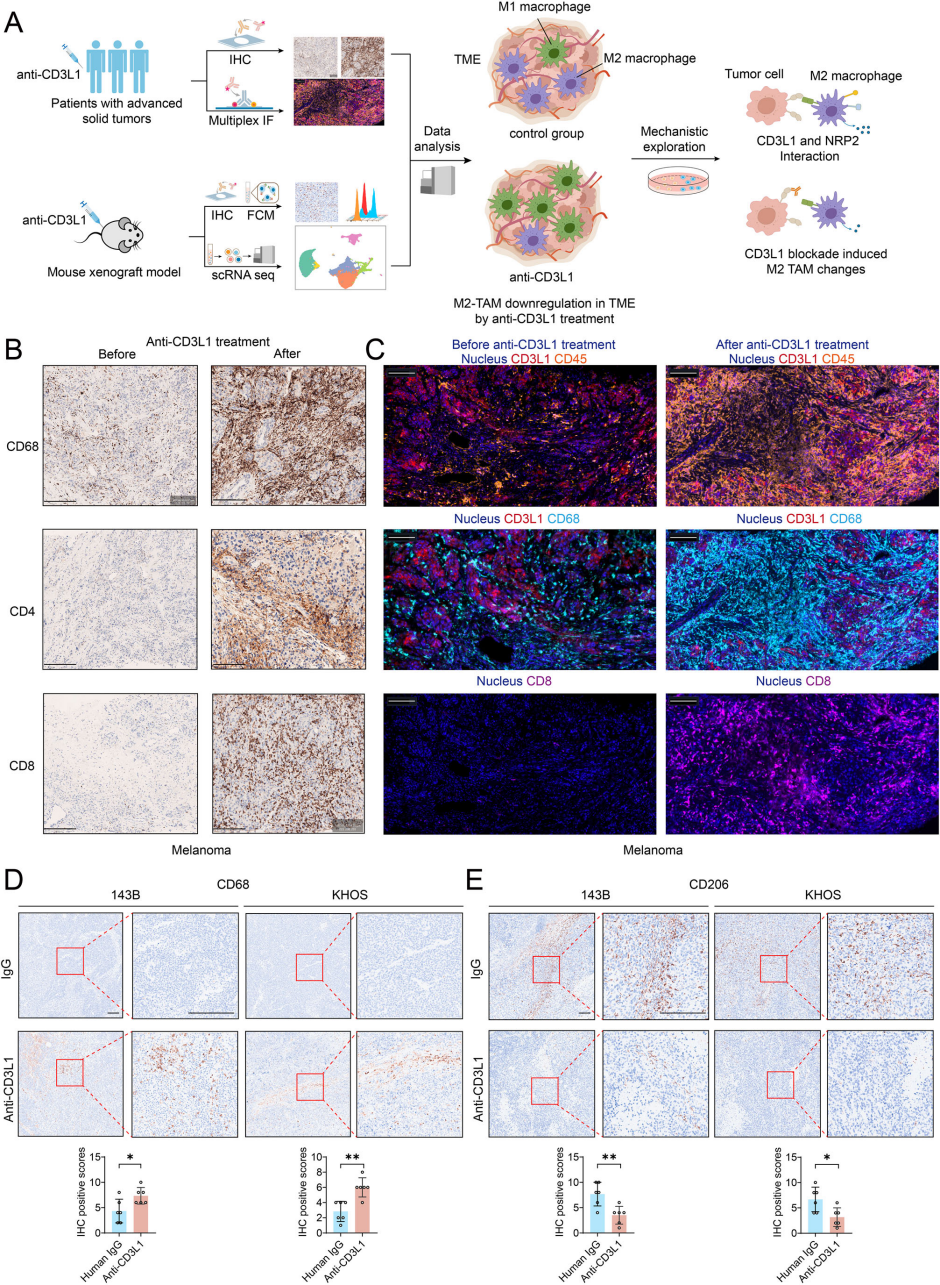
Identification of a negative correlation between anti-CD3L1 treatment and M2 TAMs in advanced tumor samples. (**A**) Schematic description of the design of the study. (**B**) Representative IHC staining of the human macrophage marker CD68 and human T-cell marker CD4/CD8 in a clinical melanoma patient refractory to PD-L1/PD1 treatment before and after 8 mg/kg anti-CD3L1 treatment under ×20 microscopic views (scale bar = 50 μm). (**C**) Representative multiplex immunofluorescence staining of the human blood-derived cell marker CD45, macrophage marker CD68, and human T-cell marker CD8 in the same clinical melanoma patient refractory to PD-L1/PD1 treatment with the expression of CD3L1 in the TME before and after 8 mg/kg anti-CD3L1 treatment under ×20 microscopic views (scale bar = 50 μm). (**D**, **E**) IHC staining of the human macrophage marker (**D**) CD68 and (**E**) M2-TAM marker CD206 in the 143B/KHOS osteosarcoma samples treated by 10 mg/kg control IgG or anti-CD3L1 from the under 5X and 20X microscopic views (scale bar = 100 μm) with IHC positive score calculation (*n* = 6 independent samples). *P < 0.05, **P < 0.01. Data are mean ± s.d. (**D**, **E**) Two-tailed unpaired Student’s *t* test. Exact *P* values: (**D**) 143B: control vs treated: *P* = 0.0276; KHOS: control vs treated: *P* = 0.0018; (**E**) 143B: control vs treated: *P* = 0.0059; KHOS: control vs treated: *P* = 0.0181. Source data are available online for this figure.

The overall design of the entire study is illustrated in Fig. 1A. In our finished Phase Ia clinical trial, we profiled tumor microenvironments using multiplex immunofluorescence and immunohistochemical analysis of immune cell markers. While anti-CD3L1 treatment induced the expected enhancement of T cell infiltration, we strikingly observed concurrent macrophage accumulation specifically in several treatment-responsive tumors (Fig. 1B,C). Notably, in a representative case from a PD-1 refractory melanoma patient, a single 8 mg/kg intravenous dose of anti-CD3L1 elicited robust increases in both T cell and macrophage infiltration and functional activation within the TME.

**Figure EV1:**
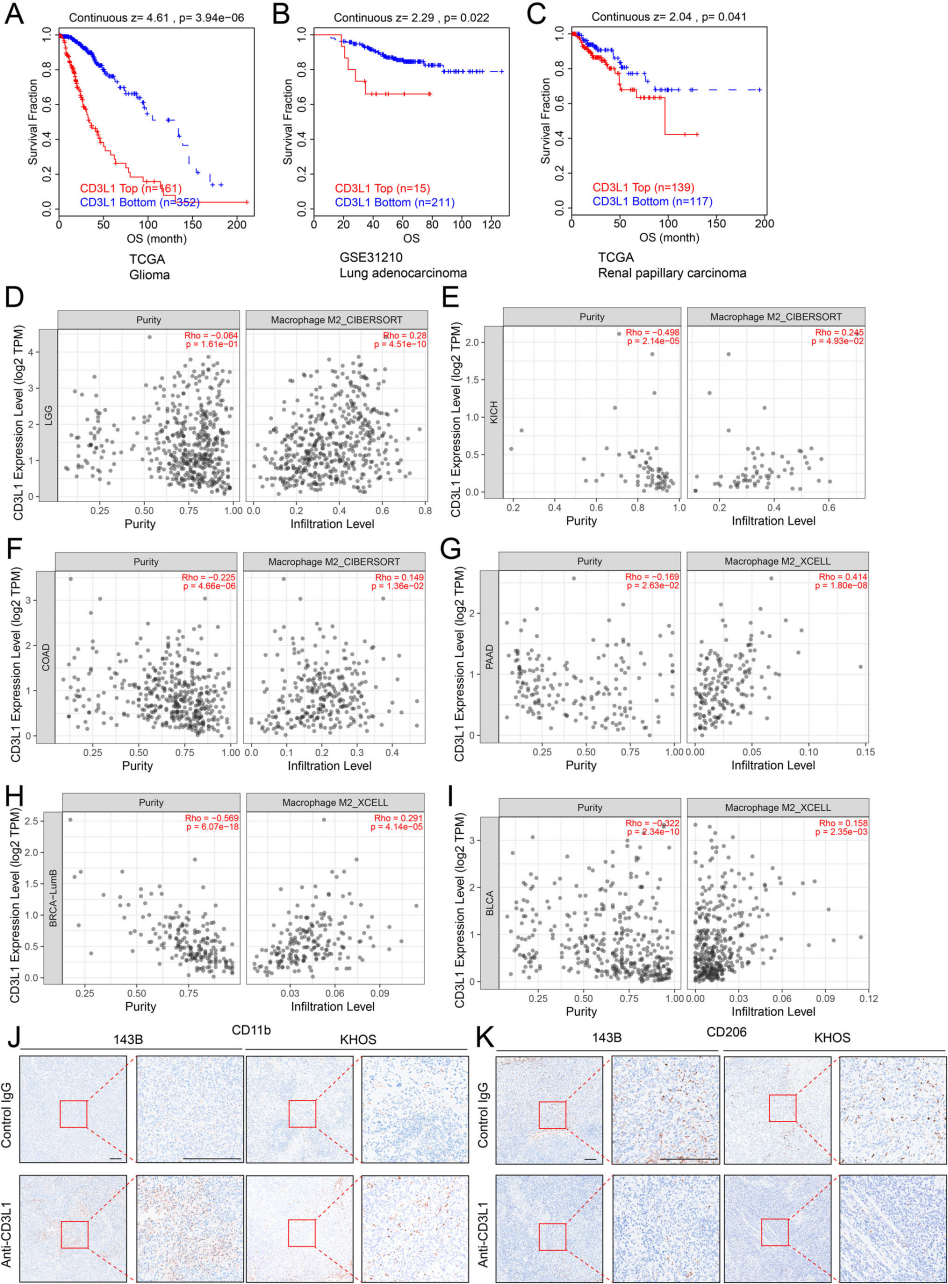
Databases showing the negative correlation between CD3L1 and clinical prognosis or M2 TAM infiltration in patients with different advanced solid tumors, related to Fig. 1. (**A**–**C**) Databases from GSE31210 and TCGA show the negative correlation between CD3L1 expression and survival rate in glioma, lung adenocarcinoma, and renal papillary cancer patients. (**D**–**I**) Databases from TIMER show the significant positive correlation between CD3L1 expression and M2 macrophage infiltration level in different cancers ((**D**) Low-grade glioma, LGG; (**E**) Kidney chromophobe, KICH; (**F**) Colon adenocarcinoma, COAD; (**G**) Pancreatic adenocarcinoma, PAAD; (**H**) Breast carcinoma-luminal B, BRCA-LumB; (**I**) Bladder cancer, BLCA) analyzed based on the TCGA database. (**J**, **K**) IHC staining of the human macrophage marker (**J**) CD11b and (**K**) M2-TAM marker CD206 in the additional 143B/KHOS osteosarcoma samples treated by 10 mg/kg control IgG or anti-CD3L1 from the under ×5 and ×20 microscopic views (scale bar = 100 μm) (*n* = 3 independent samples). (**A**–**C**) Kaplan–Meier survival analysis; (**D**–**I**) Correlation Analysis and Linear Regression Analysis (details in the methodological part).

While our prior work established a significant association between CD3L1 expression, T-cell dysfunction, and unfavorable prognosis across multiple cancers, its relationship with macrophage functionality or infiltration within the TME remained unexplored. To address this gap, we interrogated public transcriptomic datasets to evaluate correlations between CD3L1 levels, M2 macrophage infiltration, and clinical outcomes in pan-cancer cohorts (Fig. EV1A–I). Strikingly, elevated CD3L1 expression exhibited a marked positive association with pro-tumorigenic macrophage activity and a robust negative correlation with patient survival, suggesting CD3L1’s dual role in driving both immunosuppressive macrophage polarization and clinically adverse prognoses.

Given the widely elevated expression levels of CD3L1, the constrained infiltration of T-cells, and the notable significance of TAMs within the osteosarcoma TME, and the clinical efficacy, we selected osteosarcoma as the primary tumor model for investigating the correlation between CD3L1 and TAMs (Lu et al, 2025; Tao et al, 2025; He et al, 2021; Zalacain et al, 2021; Buddingh et al, 2011). Upon revisiting the osteosarcoma mouse models (143B/KHOS tumor cell lines implanted in humanized mouse model) employed in our previous study (Deng et al, 2024), we noted a substantial increase in the expression of CD11b/CD68, the marker of functional macrophages, and a reduction in the expression of CD206, a marker commonly associated with M2 TAMs (Haston et al, 2023; Liu et al, 2024; Ausejo-Mauleon et al, 2023), in these tumor samples after 10 mg/kg anti-CD3L1 treatment (Figs. 1D,E and EV1J,K). These findings collectively suggest that our anti-CD3L1 antibody may be exerting its therapeutic effects by modulating the macrophage population within the TME, specifically by reducing the proportion of immunosuppressive M2 TAMs and enhancing the activation of anti-tumor macrophages.

**Figure 2:**
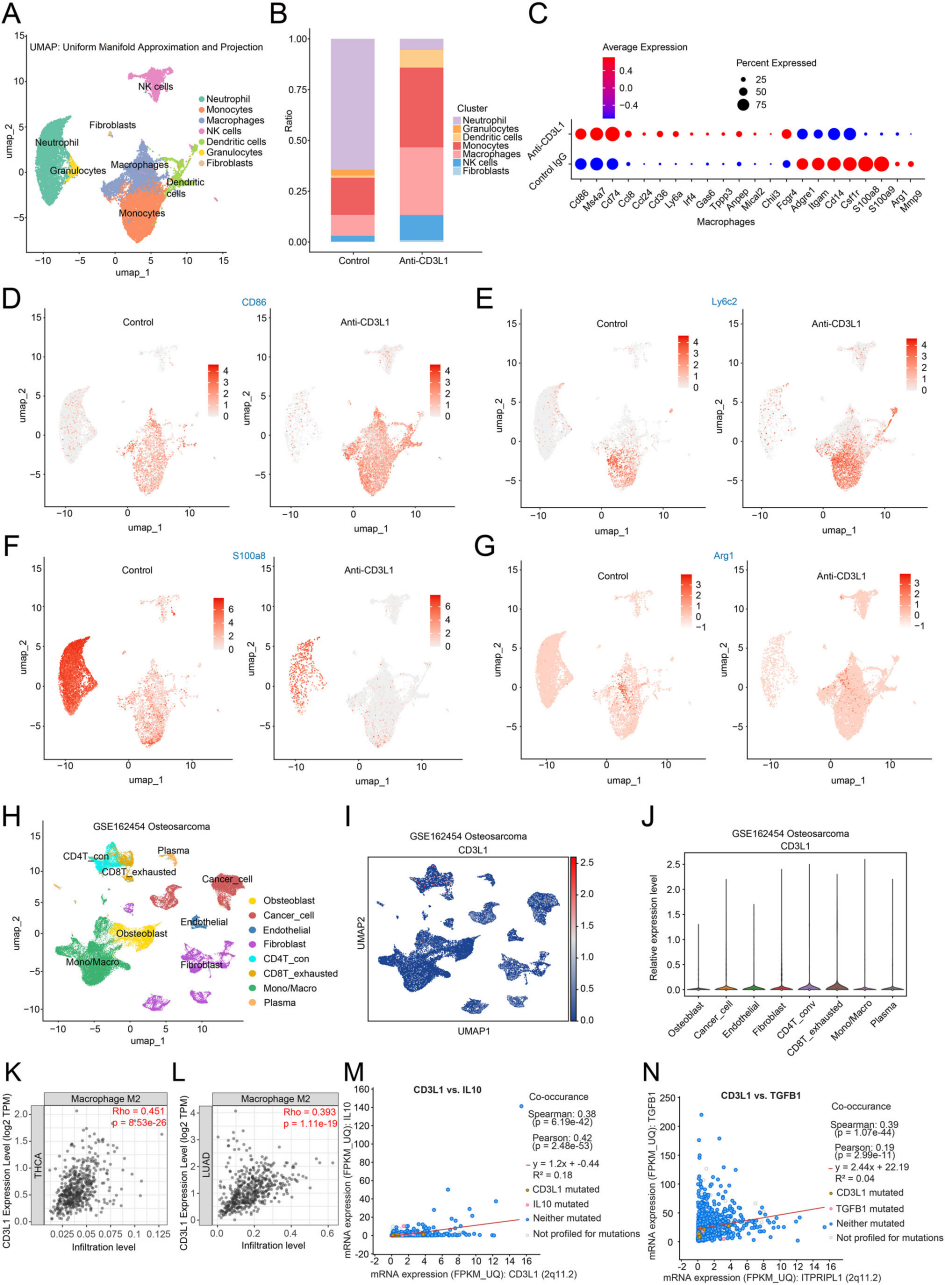
scRNA reveals the negative correlation between anti-CD3L1 treatment and M2 TAMs in the TME. (**A**, **B**) Single-cell RNA sequencing (scRNA seq) data of (**A**) Uniform manifold approximation and proximation (UMAP) plots showing the clustering of (**B**) Pie chart showing the differences in the proportion of CD45^+^ cells infiltrated in the TME of osteosarcoma samples from nude mice. (**C**) A dot plot illustrating the expression profiles of various macrophage-related genes across different macrophage subsets in the context of Anti-CD3L1 and Control IgG treatment conditions. The color gradient represents the average expression level of each gene, with red indicating higher expression and blue indicating lower expression. The size of each dot corresponds to the percentage of cells expressing the respective gene (25%, 50%, or 75%). (**D**–**G**) scRNA seq showing representative signature gene maps showing the upregulation of M1 TAM hallmark genes (**D**) CD86, (**E**) Ly6c2, and downregulation of M2 TAM hallmark genes (**F**) S100a8, (**G**) Arg1 after 10 mg/kg anti-CD3L1 treatment in comparison with control IgG treatment in the TME of osteosarcoma transplants isolated from nude mice. (**A**–**G**: *n* = 3 independent samples) (**H**, **I**) UAMP plots showing (**H**) the cell clustering, (**I**) the spatial expression of CD3L1 within the TME of public single-cell sequencing data from GSE162454 (Osteosarcoma) database. (**J**) The summary of CD3L1 expression level within the TME of GSE162454 (Osteosarcoma) database (*n* = 6 samples). (**K**, **L**) Pan-cancer analysis of different solid tumor cohorts reveals a significant positive correlation between CD3L1 expression and M2 macrophage infiltration in the TME of (**K**) Thyroid carcinoma (THCA) (**L**) Lung adenocarcinoma (LUAD). (**M**, **N**) TCGA pan-cancer atlas reveals the co-occurrence tendency and significant positive correlation between the CD3L1 expression level and expression levels of M2 TAM marker genes, including (**M**) IL-10 (**N**) TGFB1 in the TME across all types of cancer. (**K**–**N**) Correlation Analysis and Linear Regression Analysis (details in the methodological part).

**Figure EV2:**
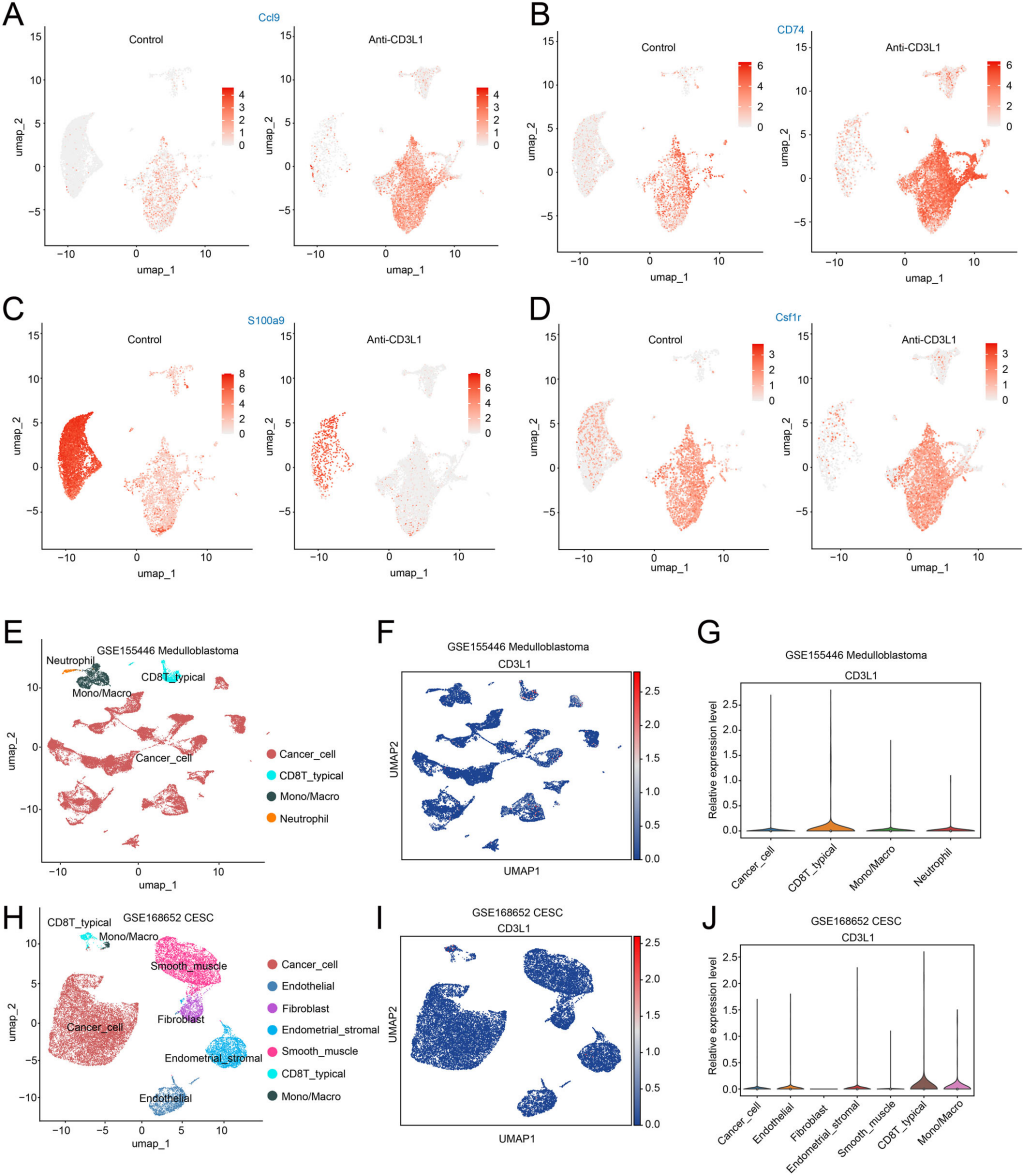
Single-cell sequencing analysis reveals the positive correlation between CD3L1 expression and M2 TAMs in the TME of advanced solid tumors, related to Fig. 2. (**A**–**D**) scRNA seq showing representative signature gene maps showing the upregulation of M1 TAM hallmark genes (**A**) CCL9 (**B**) CD74, and downregulation of M2 TAM hallmark genes (**C**) S100a9 (**D**) Csf1r after 10 mg/kg anti-CD3L1 treatment in comparison with control IgG treatment in the TME of osteosarcoma transplants isolated from nude mice. (**E**, **F**) UAMP plots showing (**E**) the cell clustering (**F**) the spatial expression of CD3L1 within the TME of public single-cell sequencing data from GSE155446 (Medulloblastoma) database. (**G**) The summary of CD3L1 expression level within the TME of GSE155446 (Medulloblastoma) database (*n* = 28 samples). (**H**,** I**) UAMP plots showing (**H**) the cell clustering (**I**) the spatial expression of CD3L1 within the TME of public single-cell sequencing data from GSE168652 (Cervical squamous cell carcinoma and endocervical carcinoma, CESC) database. (**J**) The summary of CD3L1 expression level within the TME of GSE168652 (CESC) database (*n* = 2 samples).

The decline in CD206 expression observed in our study provides compelling evidence for the downregulation of M2 TAM functionality and their infiltration patterns within the osteosarcoma TME. To delve deeper into the potential interplay between anti-CD3L1 treatment and M2 TAM dynamics, we analyzed the TME of osteosarcoma transplanted in nude mice. This approach was taken to ensure that any observed effects were not confounded by potential T-lymphocyte interference, considering the nude mouse model was naturally deficient in T cells while retaining myeloid immune components, which allowed us to directly assess the role of CD3L1 in the absence of T cells without the need to artificially alter the cellular composition of the immune microenvironment (Freedman and Shin, 1974; Martin and Martin, 1974). After treatment by 10 mg/kg control IgG or anti-CD3L1, we isolated immune cells from the implanted osteosarcoma samples by magnetic CD45 microbeads. Utilizing single-cell sequencing techniques, we analyzed the isolated immune cells within the TME (Fig. 2A). Our findings revealed a striking decrease in the percentage of the infiltrated neutrophils and an increase in macrophages in the osteosarcoma mouse models employed after anti-CD3L1 treatment (Fig. 2B). The results of the single-cell sequencing suggested a substantial upregulation of the expression of the signature genes of M1 TAMs with negative modulation of the M2 TAMs (Fig. 2C). The modulation of anti-CD3L1 treatment within TME was characterized by alterations in the expression profiles of genes that are signature markers of M1 or M2 TAM phenotypes. Specifically, we observed a substantial upregulation in the expression of genes typically associated with M1 polarization and function, such as CD86, CD74, CCL9, and Ly6c2 (Figs. 2D,E and EV2A,B), which are indicative of an inflammatory or anti-tumor response (Ni et al, 2025; Guilliams et al, 2022; Obradovic et al, 2021). The results also suggested a remarkable decrease in the genes linked to M2 polarization and function, such as S100A8, S100A9, Csf1r, and Arg1 (Figs. 2F,G and EV2C,D), which are often associated with tissue repair and immune suppression (Yao et al, 2022; Ganta et al, 2019). The details of the signature gene changes are shown in Dataset EV1. To further elucidate the association between CD3L1 and M2 TAMs within the TME, we leveraged publicly available single-cell sequencing datasets. Our analysis revealed the wide expression of CD3L1 in TME, including cancer cells, exhausted T cells, and TAMs within the TME of osteosarcoma and several other advanced solid malignancies (Figs. 2H–J, and EV2E–J). The spatial overexpression of CD3L1 in the TME provided further evidence of its potential correlation with tumor-promoting functional activities.

**Figure EV3:**
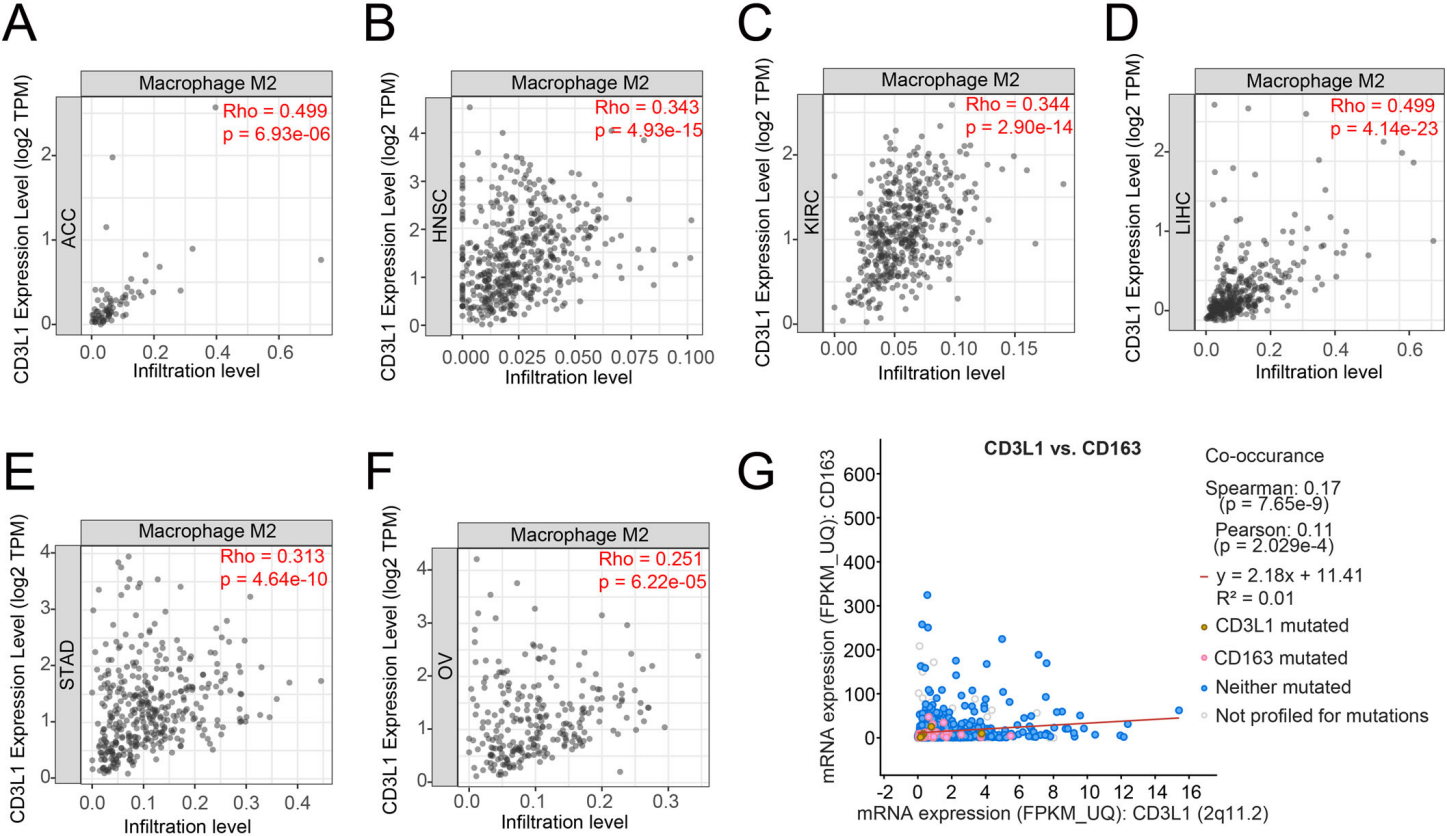
Supplementary information of datasets analysis related to Fig. 2. (**A**–**F**) Pan-cancer analysis of different solid tumor cohorts reveals a significant positive correlation between CD3L1 expression and M2 macrophage infiltration in the TME of (**A**) Adrenocortical carcinoma (ACC), (**B**) Head and neck squamous cell carcinoma (HNSC), (**C**) Kidney renal clear cell carcinoma (KIRC), (**D**) Liver hepatocellular carcinoma (LIHC), (**E**) Stomach adenocarcinoma (STAD), (**F**) Ovarian serous cystadenocarcinoma (OV). (**G**) TCGA pan-cancer atlas reveals the co-occurrence tendency and significant positive correlation between the CD3L1 expression level and the expression levels of M2 TAM marker genes CD163 in the TME across all types of cancer. (**A**–**G**) Correlation Analysis and Linear Regression Analysis (details in the methodological part).

Moreover, pan-cancer cohort studies further substantiated a positive correlation between CD3L1 expression and M2 TAM infiltration across the TME of diverse advanced solid tumors (Figs. 2K,L and EV3A–F). A subsequent pan-cancer analysis utilizing data from TCGA demonstrated a co-occurrence tendency between CD3L1 expression and the expression of M2 TAM marker genes (including IL-10, CD163, and TGFB1) within the TME (Figs. 2M,N and EV3G). This finding further underscores the positive correlation between CD3L1 and M2 TAMs, suggesting a potential functional link in the context of tumor progression and immune evasion.

In summary, our results demonstrate a robust negative correlation between anti-CD3L1 treatment and the presence or activity of M2 TAMs within the osteosarcoma TME. These findings contribute to evidence suggesting that, besides the rejuvenation of T cells, targeting CD3L1 may also offer therapeutic benefits by modulating TAM polarization and function, thereby influencing tumor progression and immune evasion mechanisms in osteosarcoma samples.

### Negative modulation of M2 TAMs function by anti-CD3L1 treatment

**Figure 3:**
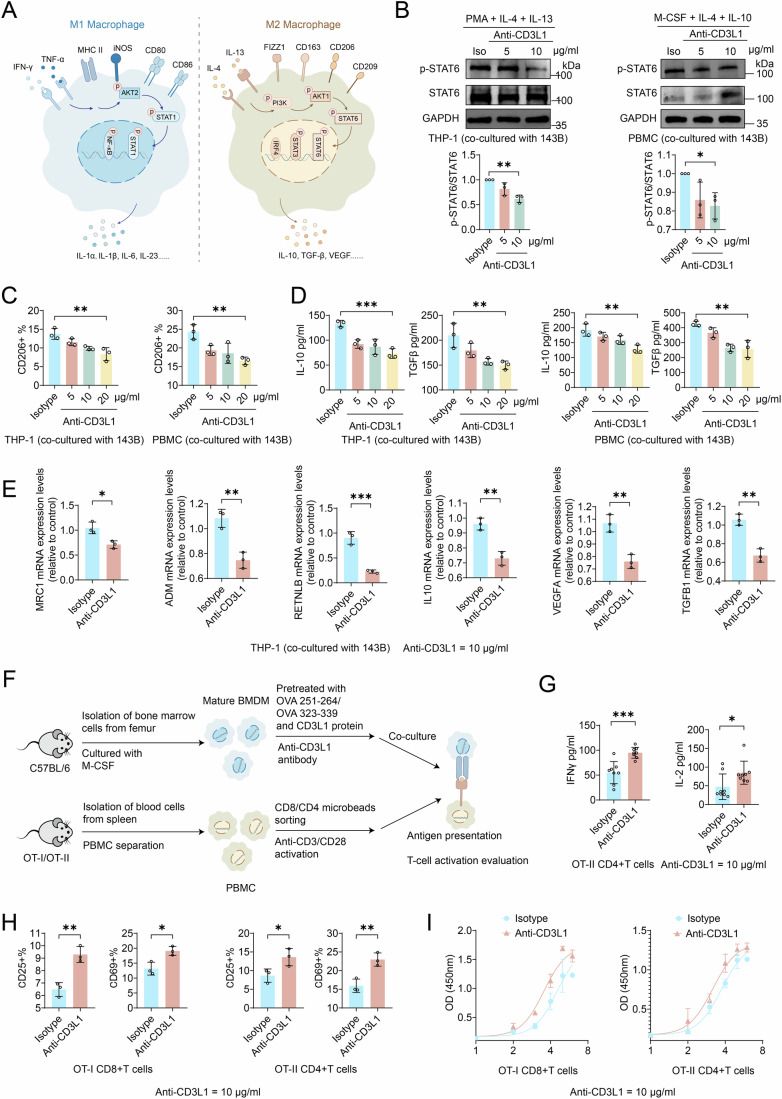
Negative modulation of M2 TAMs function by anti-CD3L1 treatment. (**A**) Schematic description of the possible impact of anti-CD3L1 on the M2 TAM function. (**B**) Immunoblot showing a substantial decrease in the phosphorylation of STAT6 after anti-CD3L1 treatment in induced M2 TAMs from THP-1 cells or PBMCs co-cultured with 143B cells (*n* = 3 independent experiments). (**C**) Representative flow cytometry results showing a substantial downregulation of M2 TAM marker CD206 after anti-CD3L1 treatment in the co-culture system (*n *= 3 independent samples). (**D**) ELISA showing a remarkable decrease in the IL-10 and TGF-β secretion after anti-CD3L1 treatment in the co-culture system (*n* = 3 independent samples). (**E**) qPCR showing a significant downregulation of the signature genes of M2 TAMs (TGFB1, IL-10, VEGFA, ADM, MRC1, RETNLB) after anti-CD3L1 treatment in the induced M2 TAMs (THP-1) from the co-cultured systems (*n* = 3 independent experiments). (**F**) Schematic description of the antigen presentation assay. (**G**) ELISA showing a significant increase in the secreted cytokines (IFNγ, IL-2) after anti-CD3L1 treatment from the co-cultured CD4^ +^ T cells isolated from OT-II mice (*n* = 8 independent wells). (**H**) Representative flow cytometry results showing the upregulation of antigen-presentation capacity of BMDMs after anti-CD3L1 treatment by evaluating the upregulated activation markers (CD25/CD69) of co-cultured CD8/CD4^+^ T cells isolated from OT-I or OT-II mice (*n* = 3 independent samples). (**I**) CCK-8 assay showing the growth curve of co-cultured CD8/CD4^+^ T cells isolated from OT-I or OT-II mice (*n* = 3 independent samples). *P < 0.05, **P < 0.01, ***P < 0.001. Data are mean ±  s.d. (**B**–**D**) One-way ANOVA; (**E**, **G**, **H**) Two-tailed unpaired Student’s *t* test. Exact *P* values: (**B**) THP-1 model: *P* = 0.0044; PBMC model: *P* = 0.0454; (**C**): THP-1 model: *P* = 0.0041; PBMC model: *P* = 0.0057. (**D**) THP-1 IL-10: *P* = 0.0006; TGFB: *P* = 0.0056; PBMC IL-10: *P* = 0.0077; TGFB: *P* = 0.0013. (**E**) IL-10: *P* = 0.0028; TGFB1: *P* = 0.0020; MRC1: *P* = 0.0139; RETNLB: *P* = 0.0009; VEGFA: *P* = 0.0041; ADM: *P *= 0.0039. (**G**) IL-2: *P* = 0.0368; IFNγ: *P* = 0.0004. (**H**) OT-I: CD25: *P* = 0.0045; CD69: *P* = 0.0175; OT-II: CD25: *P *= 0.0429; CD69: P = 0.0085. Source data are available online for this figure.

To further elucidate the regulatory mechanisms between anti-CD3L1 treatment and M2 TAMs, we conducted a comprehensive series of experiments aimed at assessing the impact of anti-CD3L1 on the functional characteristics of M2 TAMs (Fig. 3A). Specifically, we utilized M2 TAMs derived from the THP-1 monocyte cell line, which were induced to differentiate into M2 phenotype macrophages through a sequential treatment with proper concentration of phorbol 12-myristate 13-acetate (PMA), followed by interleukin-4 (IL-4) and interleukin-13 (IL-13) (Gunassekaran et al, 2021; Huang et al, 2018; Su et al, 2015; Genin et al, 2015). Consequently, we co-cultured the induced M2 macrophages with 143 tumor cells at 5:1 ratio for 24 h, to build a tumor-macrophage co-culture model for the following in vitro studies.

Subsequently, we conducted immunoblotting analyses to investigate the downstream signaling pathways affected by anti-CD3L1 in these M2 TAMs. The canonical signaling of M2 TAMs involves the PI3K axis, the PPARγ axis, and the JAK-STAT axis (Chen et al, 2023; Fichtner-Feigl et al, 2006; Ghosh et al, 2024). Our findings revealed that anti-CD3L1 treatment significantly downregulated the phosphorylation of signal transducer and activator of transcription 6 (STAT6), a key regulatory molecule in M2 TAM signaling in the induced M2 TAMs (Fig. 3B). IL-4 and IL-13 were generally considered as the initiator and activator of the M2 TAMs signaling, and the dephosphorylation of STAT6 further indicated the inhibition of M2 TAMs function by anti-CD3L1 treatment. Comparable results were observed in M2 TAMs derived from peripheral blood mononuclear cells (PBMCs), which were induced to differentiate by macrophage colony-stimulating factor (M-CSF), followed by IL-4 and interleukin-10 (IL-10), that anti-CD3L1 treatment can substantially downregulate the downstream signaling of the M2 TAMs.

**Figure EV4:**
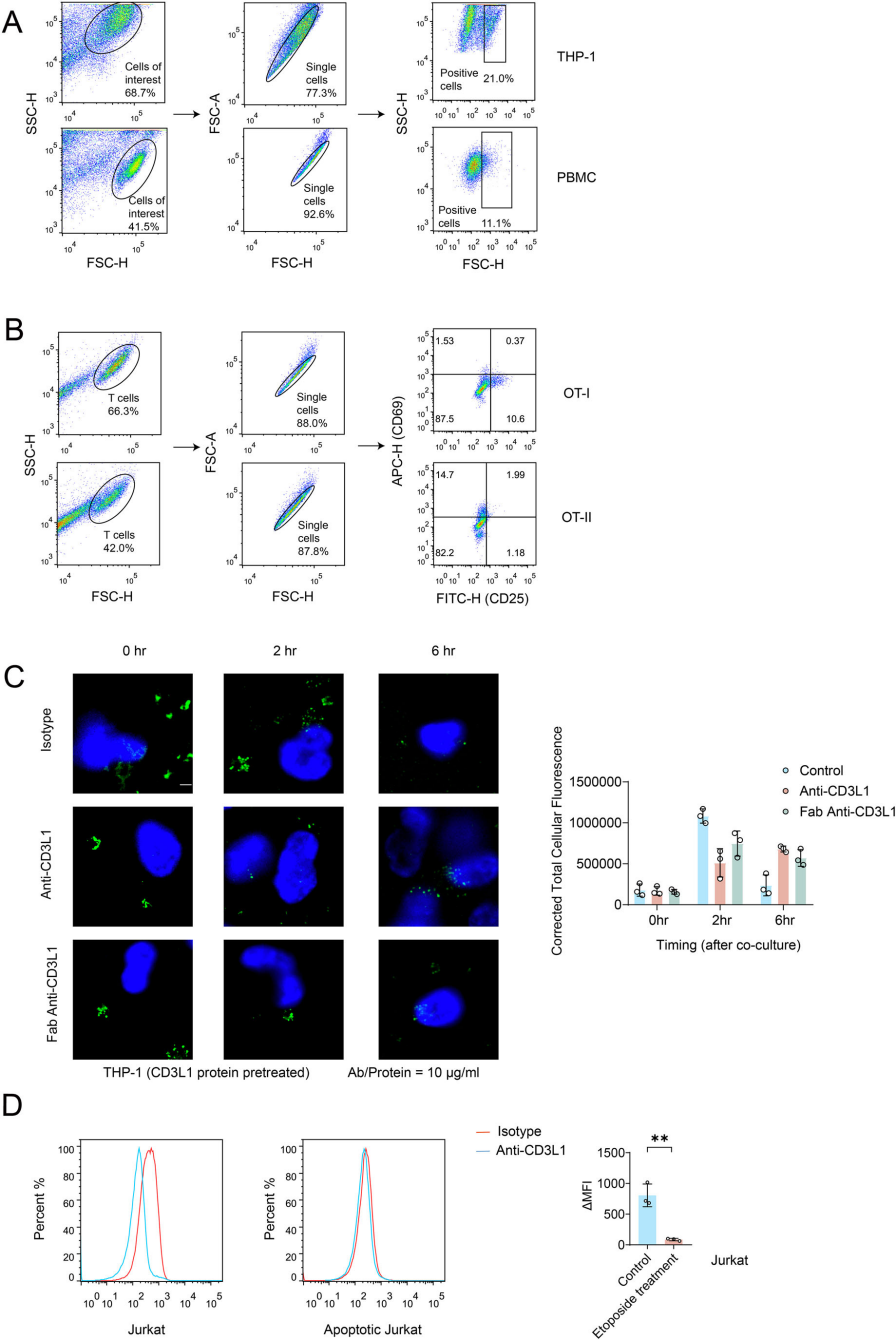
Supplementary information of the experiments related to Figs. 3–4. (**A**) The gating strategy of the induced cells: cells were first gated by FSC-H/SSC-H to select out the main cell populations, and then gated by FSC-A/FSC-H to select out the single cells, and then gated by APC-H/SSC-H to select out the CD206-positive populations. (**B**) The gating strategy of the T cells: cells were first gated by FSC-H/SSC-H to select out the main cell populations, and then gated by FSC-A/FSC-H to select out the single cells, and then gated by APC-H/SSC-H and FITC-H/SSC-H to select out the CD69/CD25 positive populations. (**C**) The microscopic views (×60) of efferocytosis assay with induced THP-1 cells (pre-treated by CD3L1 protein) treated by control isotype or anti-CD3L1 or Fab anti-CD3L1 protein engulfing stained apoptotic Jurkat cells at timing 0, 2 and 6 h after the efferocytosis with the respective analysis showing significant downregulation in efferocytotic capacity of the induced M2-like TAMs from THP-1 cells after 10 μg/ml anti-CD3L1 treatment (*n* = 3 independent samples) (scale bar = 5 μm). (**D**) Representative flow cytometry results showing significant diminished binding between Jurkat cells and anti-CD3L1 after etoposide treatment (*n* = 3 independent samples). **P < 0.01. Data are mean ± s.d. (**D**) Two-tailed unpaired Student’s *t* test. Exact *P* values: Control vs Etoposide: *P* = 0.0034.

To further corroborate our findings, flow cytometry analysis was utilized to evaluate the CD206 surface expression directly, which demonstrated a downregulation of CD206 expression in the induced M2 TAMs in response to anti-CD3L1 treatment of different concentrations (Figs. 3C and EV4A). The signature cytokines secreted by M2 TAMs, namely IL-10 and transforming growth factor-beta (TGF-β) (Cai et al, 2024), were analyzed using enzyme-linked immunosorbent assay (ELISA). Our results indicated a remarkable decrease in the secretion of both IL-10 and TGF-β following anti-CD3L1 treatment in a concentration-dependent pattern in the induced M2 TAMs derived from THP-1 or PBMC (Fig. 3D).

To gain insights into the broader impact of anti-CD3L1 on TAM polarization, we conducted quantitative polymerase chain reaction (qPCR) analyses to evaluate the expression of signature genes associated with M2 TAMs. Specifically, the mRNA expression of signature genes indicative of M2 TAMs (*CD206(MRC1), RETNLB, TGFB1, IL-10, VEGFA, ADM*) in THP-1-induced M2 TAMs was evaluated and analyzed (Cai et al, 2024; Han et al, 2023) (Fig. 3E; Table EV1). The substantial downregulation of the relative mRNA expression levels of the M2 TAMs signature genes in the induced M2 TAMs by 10 μg/ml anti-CD3L1 treatment indicated the negative impact of anti-CD3L1 on M2 TAM polarization.

Besides the functions above, key immune cells within the innate immune system, macrophages, exhibit a multifaceted role in orchestrating immune responses, particularly as antigen-presenting cells (APC) (Waibl Polania et al, 2025; Wang et al, 2025; Zhang et al, 2025). Previous studies have confirmed that M1 TAMs were equipped with more robust antigen-presentation capacities. After selecting bone marrow-derived macrophages (BMDMs) as antigen-presenting cells (APCs) (Ghosh et al, 2024; Wang et al, 2023; Zhou et al, 2022a; He et al, 2022), we employed ovalbumin (OVA) as a model antigen and utilized CD8^+^/CD4^+^ T cells isolated from OT-I/OT-II transgenic mice as responder T cells (Lin et al, 2024; Stein et al, 2021). We co-cultured the CD3L1-pre-treated, OVA-processed mature BMDMs with the T cells with different concentrations of anti-CD3L1 antibody for a proper period, and activation markers of T cells (CD25/CD69), the growth curve of the T cells, and the activation cytokines produced (IL-2/IFNγ) were tested to evaluate the antigen-presentation process (Figs. 3F–I and EV4B). The results suggested that after anti-CD3L1 treatment, the percentage of activated T cells, the proliferation of the T cells, and the cytokine production were substantially upregulated, indicating the reinforcement of antigen-presentation capacity of the macrophages by anti-CD3L1 treatment. Collectively, these results provide compelling evidence for a substantial downregulation of M2 TAM function by anti-CD3L1 treatment, suggesting the potential role of CD3L1 in M2 TAM polarization and immune modulation in TME.

### The modulation of M2 TAMs function by anti-CD3L1 treatment was Fc-independent

**Figure 4:**
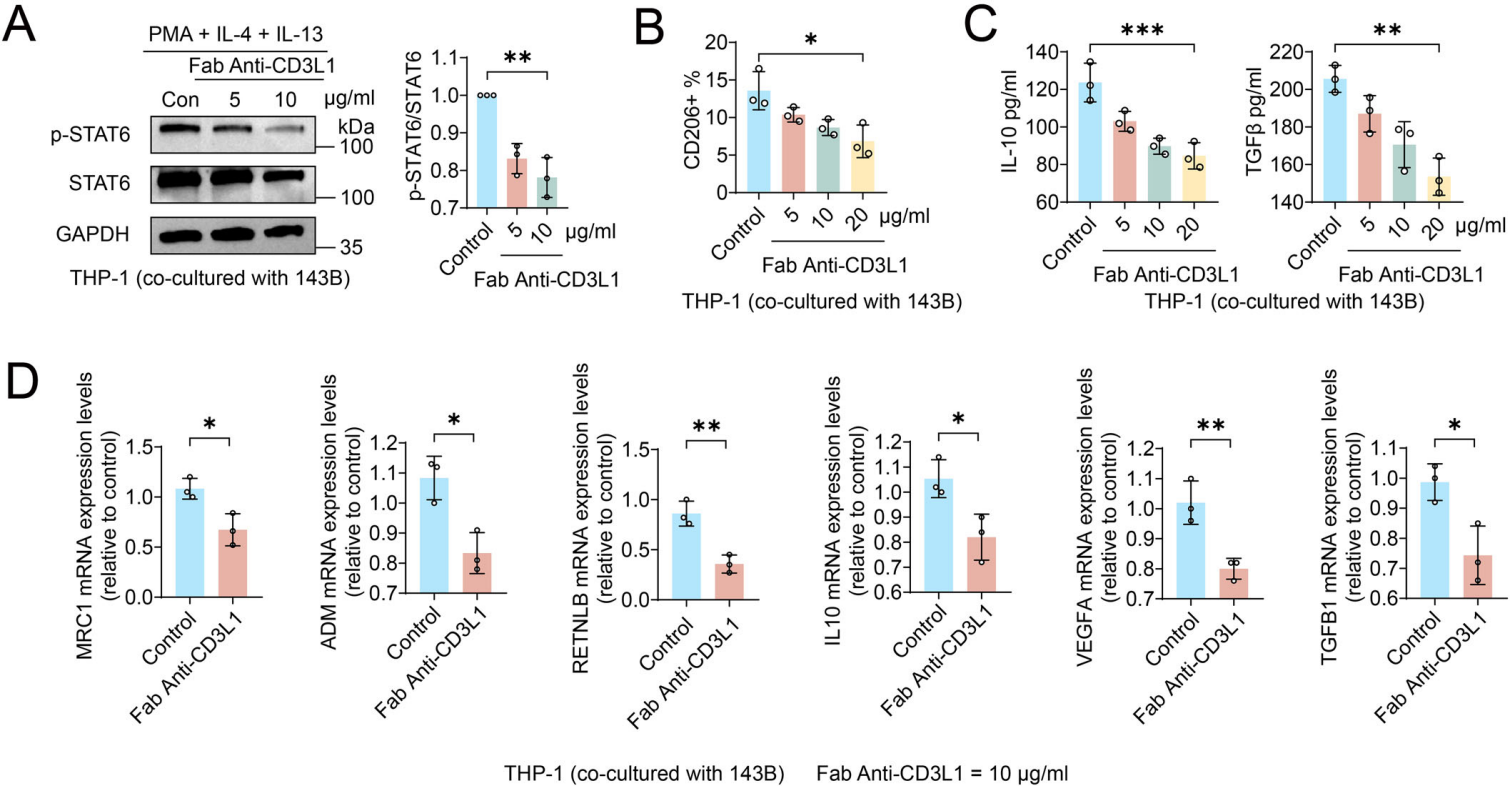
Anti-CD3L1 modulates M2 TAMs through an Fc-independent pattern. (**A**) Immunoblot showing a substantial decrease in the phosphorylation of STAT6 after Fab anti-CD3L1 protein treatment in induced M2 TAMs from THP-1 cells co-cultured with 143B cells (*n* = 3 independent experiments). (**B**) Representative flow cytometry results showing a substantial downregulation of M2 TAM marker CD206 after Fab anti-CD3L1 protein treatment in the co-culture system (*n* = 3 independent samples). (**C**) ELISA showing a remarkable decrease in the IL-10 and TGF-β secretion after Fab anti-CD3L1 protein treatment in the co-culture system (*n* = 3 independent samples). (**D**) qPCR showing a significant downregulation of the signature genes of M2 TAMs (TGFB1, IL-10, VEGFA, ADM, MRC1, RETNLB) after Fab anti-CD3L1 protein treatment in the induced M2 TAMs (THP-1) from the co-cultured systems (*n* = 3 independent experiments). *P < 0.05, **P < 0.01, ***P < 0.001. Data are mean ± s.d. (**A**–**C**) Two-tailed unpaired Student’s *t* test. (**D**) One-way ANOVA. Exact *P* values: (**A**) *P* = 0.0010. (**B**) *P* = 0.0107. (**C**) IL-10: *P* = 0.0004; TGFB: *P* = 0.0012. (**D**) IL-10: *P* = 0.0273; TGFB1: *P* = 0.0213; MRC1: *P *= 0.0206; RETNLB: *P* = 0.0028; VEGFA: *P* = 0.0089; ADM: *P* = 0.0121. Source data are available online for this figure.

Previous studies have demonstrated that the Fc region of antibodies can be recognized and bound by various Fc gamma receptors (FcγRs), including FcγRI/CD64, FcγRII/CD32, and FcγRIII/CD16, which are expressed on the surface of macrophages (Bond et al, 2024; Lopez-Sanz et al, 2023; Danzer et al, 2020; Zhang et al, 2020; Lo Russo et al, 2019). This binding interaction is essential for the opsonization process, wherein antibodies facilitate the phagocytosis of antigens by macrophages. In addition, it promotes intracellular signaling, enhances phagocytotic activity, and augments the inflammatory response. Given the well-established close correlation between macrophage regulation and the Fc fragment of antibodies, we conducted functional evaluation experiments again, encompassing immunoblotting, flow cytometry, cytokine ELISA, and qPCR (Fig. 4A–D). These experiments were performed using Fab proteins derived from anti-CD3L1 (Fab anti-CD3L1 protein) to eliminate any potential interference from the Fc fragments of anti-CD3L1. The results obtained were comparable to those from treatments with intact anti-CD3L1 antibodies, indicating that the negative modulation of M2 TAMs by anti-CD3L1 was Fc-independent.

Previous studies have highlighted the robust phagocytotic and efferocytotic capabilities of M2 macrophages, emphasizing their specialized role in immune regulation, tissue repair, and waste clearance in the microenvironment (Kumar Jha et al, 2024; Govindappa and Elfar, 2022; Vergadi et al, 2017). Therefore, we investigated whether anti-CD3L1 treatment affected these functions in M2 TAMs (Fig. EV4C). Utilizing apoptotic Jurkat cells stained by pHrodo green STP ester as the efferocytotic targets, our findings revealed a significant diminution in the efferocytotic functions of the induced M2 TAMs following anti-CD3L1/Fab anti-CD3L1 protein treatment. In addition, our findings indicate that apoptotic Jurkat cells failed to exhibit binding to the anti-CD3L1 antibody, potentially due to the specificity of the epitope recognized by this antibody. The anti-CD3L1 antibody targets the “RLLEMEFEERKRAAE” sequence, and it is plausible that this epitope undergoes modification or becomes inaccessible as a result of apoptotic processes, thereby abrogating antibody binding (Fig. EV4D). The results collectively suggested that the regulation of M2 TAMs by anti-CD3L1 was independent of the Fc fragments.

### CD3L1 interacts with NRP2 to promote M2 TAMs polarization

**Figure 5:**
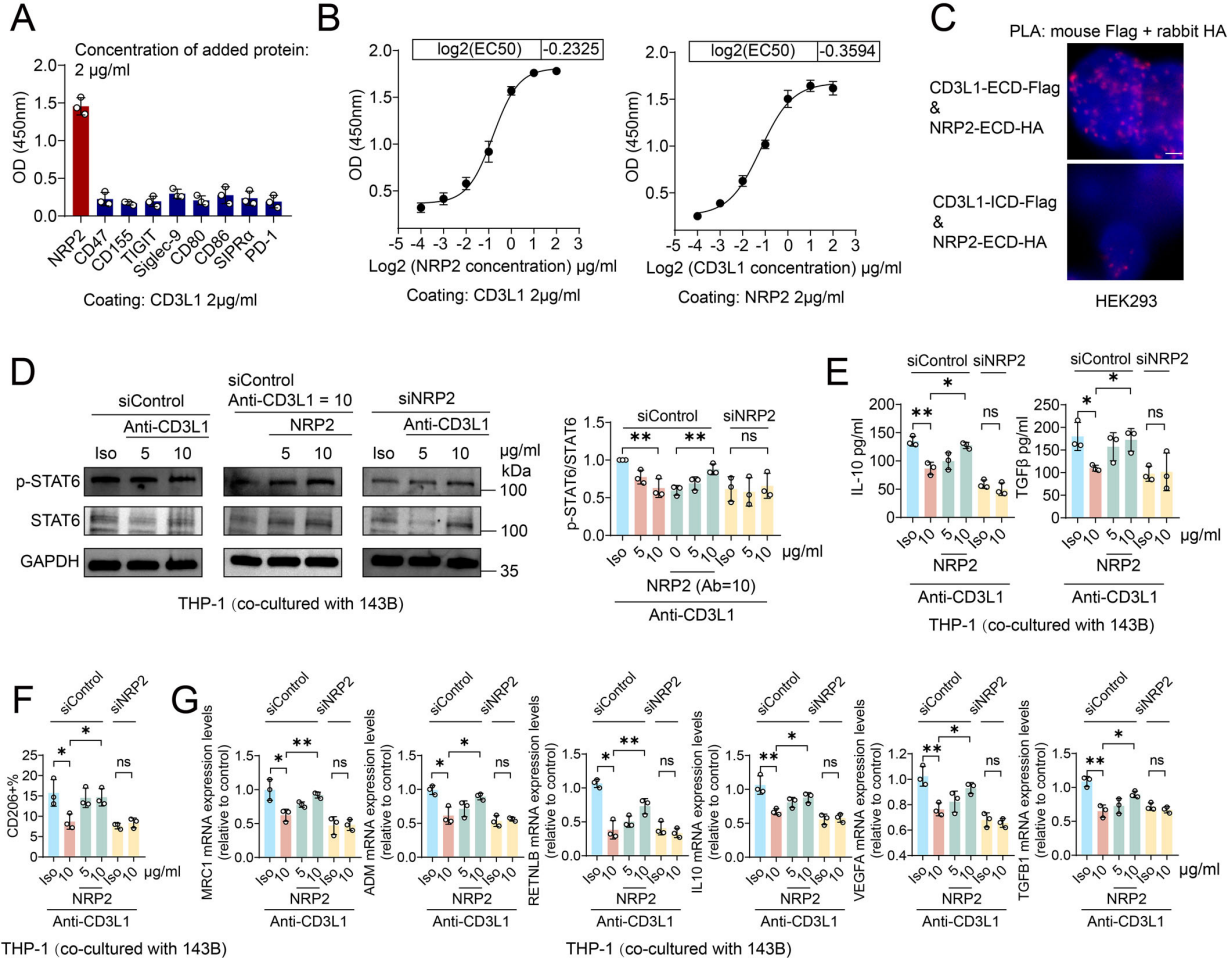
CD3L1 interacts with NRP2 to promote M2 TAMs polarization. (**A**) Screening ELISA showing that NRP2 was the sole protein closely correlated with macrophage-associated immunity with high affinity to CD3L1 (the ectodomains of different proteins were applied, *n* = 3 independent wells). (**B**) S-shaped binding curve between CD3L1 protein and NRP2 protein (*n *= 3 independent wells). (**C**) Representative images of PLA assay showing the binding between CD3L1-ECD but not CD3L1-ICD and NRP2 (*n* = 3 independent samples) (scale bar = 5 μm). (**D**) Immunoblot showing the decrease in the phosphorylation of STAT6, (**E**) ELISA showing the decrease in the IL-10 and TGF-β secretion, (**F**) flow cytometry showing the downregulation of M2 TAM marker CD206, (**G**) qPCR showing the downregulation of the signature genes of M2 TAMs (TGFB1, IL-10, VEGFA, ADM, MRC1, RETNLB) after anti-CD3L1 treatment can be reproducible in M2 TAMs induced from THP-1 cells (co-cultured with 143B cells) transfected with control siRNA but not siRNA knocking down NRP2, and the knockdown of NRP2 results in downregulation of M2 TAMs itself, and the effect of anti-CD3L1 can be rescued by NRP2 protein (*n* = 3 independent experiments). *P < 0.05, **P < 0.01, with ns as no significance. Data are mean ± s.d. (**D**–**G**) One-way ANOVA and two-tailed unpaired Student’s *t* test. Exact *P* values: (**D**) siControl control vs treated: *P* = 0.0064; siControl+anti-CD3L1+control vs siControl+anti-CD3L1 + NRP2: *P* = 0.0107; siNRP2 control vs treated: *P* = 0.8569. (**E**) IL-10: siControl control vs treated: *P* = 0.0059; siControl+anti-CD3L1+control vs siControl+anti-CD3L1 + NRP2: *P* = 0.0108; siNRP2 control vs treated: *P* = 0.3201. TGFB: siControl control vs treated: *P* = 0.0391; siControl+anti-CD3L1+control vs siControl+anti-CD3L1 + NRP2: *P* = 0.0388; siNRP2 control vs treated: *P* = 0.8768. (**F**) siControl control vs treated: *P* = 0.0328; siControl+anti-CD3L1+control vs siControl+anti-CD3L1 + NRP2: *P* = 0.0230; siNRP2 control vs treated: *P* = 0.3928. (**G**) IL-10: siControl control vs treated: *P* = 0.0059; siControl+anti-CD3L1+control vs siControl+anti-CD3L1 + NRP2: *P* = 0.0245; siNRP2 control vs treated: *P* = 0.7436. TGFB1: siControl control vs treated: *P* = 0.0027; siControl+anti-CD3L1+control vs siControl+anti-CD3L1 + NRP2: *P* = 0.0339; siNRP2 control vs treated: *P* = 0.3223. MRC1: siControl control vs treated: *P* = 0.0142; siControl+anti-CD3L1+control vs siControl+anti-CD3L1 + NRP2: *P* = 0.0033; siNRP2 control vs treated: *P* = 0.9162. RETNLB: siControl control vs treated: *P* = 0.0003; siControl+anti-CD3L1+control vs siControl+anti-CD3L1 + NRP2: *P* = 0.0252; siNRP2 control vs treated: *P* = 0.3734. VEGFA: siControl control vs treated: *P* = 0.0105; siControl+anti-CD3L1+control vs siControl+anti-CD3L1 + NRP2: *P* = 0.0426; siNRP2 control vs treated: *P* = 0.5725. ADM: siControl control vs treated: *P* = 0.0123; siControl+anti-CD3L1+control vs siControl+anti-CD3L1 + NRP2: *P* = 0.0420; siNRP2 control vs treated: *P* = 0.6159. Source data are available online for this figure.

**Figure EV5:**
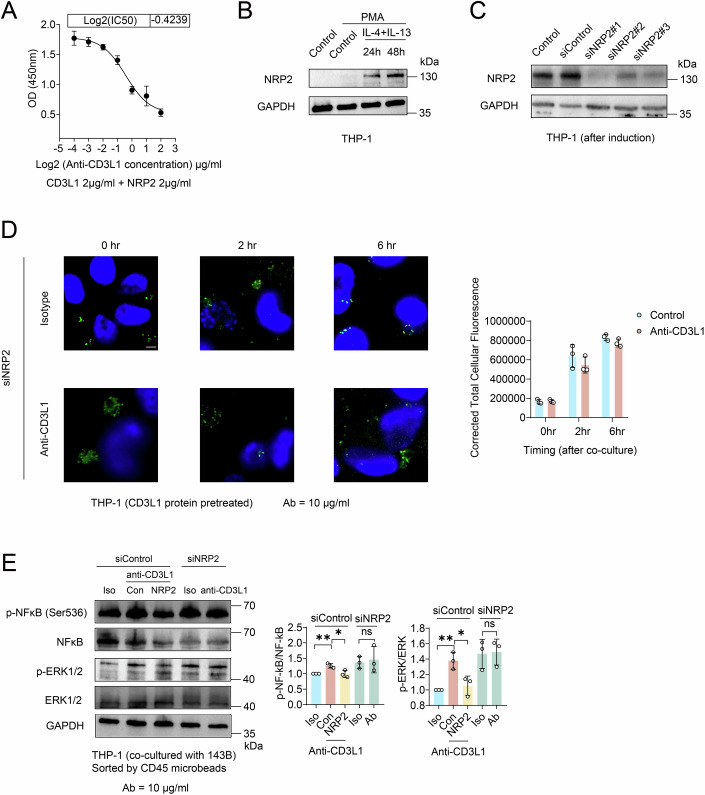
Supplementary information of the experiments related to Fig. 5. (**A**) ELISA showing blockade of CD3L1-NRP2 interaction by different concentrations of anti-CD3L1 (*n* = 3 independent wells). (**B**) Immunoblot showing the NRP2 expression in different stages of induced THP-1 cells (*n* = 3 independent experiments). (**C**) Immunoblot showing the knockdown effect of NRP2 by different siRNAs in THP-1 cells (*n* = 3 independent experiments). (**D**) The microscopic views (×60) of efferocytosis assay with induced THP-1 cells (pre-treated by CD3L1 protein) after siRNAs treatment treated by control isotype or anti-CD3L1 engulfing stained apoptotic Jurkat cells at timing 0, 2, and 6 h after the efferocytosis with the respective analysis showing no significant downregulation in efferocytotic capacity of the M2-like TAM induced THP-1 cells with siRNA knocking down NRP2 treatment after 10 μg/ml anti-CD3L1 treatment (*n* = 3 independent samples) (scale bar = 5 μm). (**E**) Representative immunoblot results showing that anti-CD3L1 can counteract the inhibition of NRP2 on the downstream signaling pathways (NFκB and MAPK-ERK), which can be restored by NRP2 protein and diminished by NRP2 knockdown (*n* = 3 independent experiments). *P < 0.05, **P < 0.01, with ns as no significance. Data are mean ± s.d. (**E**) Two-tailed unpaired Student’s *t* test. Exact *P* values: pNF-κB/NF-κB: siControl: Isotype vs anti-CD3L1: *P* = 0.0040; anti-CD3L1 vs NRP2 protein: *P* = 0.0296; siNRP2: Isotype: anti-CD3L1: *P* = 0.7501;pERK/ERK: siControl: Isotype vs anti-CD3L1: *P* = 0.0040; anti-CD3L1 vs NRP2 protein: *P* = 0.0281; siNRP2: Isotype: anti-CD3L1: *P *= 0.8837.

To conduct a comprehensive investigation of the regulatory network between CD3L1 and M2 TAMs, we started with the screening of potential CD3L1-interacting molecules that are highly correlated with macrophage functions. Through this analysis, we identified that among the myriad of important macrophage-associated immune-related molecules, only Neuropilin-2 (NRP2) demonstrated a robust interaction with the CD3L1 protein (Billerhart et al, 2021; Cao et al, 2021; Zhou et al, 2022b; Guan et al, 2024; Zhu et al, 2022; Mei et al, 2023; Bader et al, 2024; Zhang et al, 2023) (Fig. 5A). To further validate this interaction, we utilized ELISA technique, employing various concentrations of the proteins. The results from these assays demonstrated a substantial affinity between CD3L1 and NRP2 (Fig. 5B). We further performed a proximity ligation assay (PLA) using HEK293 cells engineered to overexpress either the extracellular domain (ECD) or the intracellular domain (ICD) of CD3L1, in conjunction with the ECD of NRP2 (Fig. 5C). The results suggested that the ECD of CD3L1 interacted with the ECD of NRP2, which were consistent with the ELISA findings. Additionally, our further exploration revealed that the anti-CD3L1 antibody could significantly block the interaction between CD3L1 and NRP2 in a concentration-dependent pattern, supporting the potential role of CD3L1-NRP2 interaction in the regulation of macrophage functions (Fig. EV5A).

Previous studies have established that NRP2 plays a pivotal role in promoting the polarization and functional activities of M2 TAMs in TME (Roy et al, 2018; Yang et al, 2022b). We first confirmed the expression of NRP2 in different stages of THP-1 cells during the M2 TAMs induction (Fig. EV5B). Given the notable expression and function of NRP2 on M2 TAMs, we adhered to standardized protocols to knock down NRP2 using siRNA during the induction of M2 TAMs from THP-1 cells. The siRNA sequences are shown in Table EV2. By immunoblotting assay analysis, we selected the siRNA with the best NRP2 knockdown effect for further experiments (Fig. EV5C). To assess the functional consequences of NRP2 knockdown and the role of CD3L1-NRP2 interaction in the M2 TAMs polarization and function, we tested several key aspects of M2 TAMs as mentioned above, with or without anti-CD3L1 treatment. The evaluations included the phosphorylation of STAT6 by immunoblot, CD206 expression by flow cytometry, IL-10 and TGF-β secretion by ELISA, the expressions of signature genes associated with M1 and M2 TAMs by qPCR, and the efferocytotic function of M2 TAMs by immunofluorescence in the THP-1 derived M2 TAMs co-cultured with tumor cells. These evaluations were conducted in comparison with control siRNA-treated cells (Figs. 5D–G and EV5D). We noticed that in the THP-1 induced M2 TAMs treated by control siRNA, the results after 10 μg/ml anti-CD3L1 treatment observed in our previous experiments were reproducible and comparable. In comparison, after NRP2 knockdown by the siRNA, the results of these experiments demonstrated that the administration of anti-CD3L1 no longer exhibited substantial changes in the polarization and functional activities of the induced M2 TAMs, thereby highlighting the crucial role of NRP2 in the regulatory interplay between CD3L1 and M2 TAMs. Additionally, after NRP2 knockdown, the polarization and function of the induced M2 TAMs were significantly compromised, which was in accordance with previous studies and the effect of anti-CD3L1 treatment. Furthermore, considering knockdown of NRP2 might induce a maximal effect, potentially masking any additional effects conferred by anti-CD3L1, we designed a series of experiments employing varying concentrations of human NRP2 protein as a competitive binder to NRP2 in the presence of anti-CD3L1. The results of these experiments revealed a significant rescue effect, providing compelling evidence that the influence of anti-CD3L1 on macrophages is indeed contingent upon the CD3L1-NRP2 axis (Fig. 5D–G).

Previous studies have demonstrated that in macrophages, NRP2 exhibits strong correlations with NF-κB and MAPK-ERK signaling pathways. Specifically, NRP2 activation has been shown to downregulate both NF-κB and MAPK-ERK pathways, thereby suppressing M1 macrophage polarization (Dhupar et al, 2024; Yang et al, 2022b; Li et al, 2024b; Schellenburg et al, 2017; Rey-Gallardo et al, 2011). Given that evaluating downstream NRP2 signaling provides a more direct reflection of the regulatory effects mediated by the CD3L1-NRP2 axis on macrophage function, we selected NF-κB p65 and ERK1/2 as representative markers of these pathways. We assessed the phosphorylation status of NF-κB p65 (Ser536) and ERK1/2 (Thr202/Tyr204) to investigate the impact of anti-CD3L1 treatment (Fig. EV5E). Our results indicated that anti-CD3L1 significantly counteracts the inhibitory effects of NRP2 on downstream signaling, an effect that could be restored by exogenous NRP2 protein supplementation. Conversely, NRP2 knockdown attenuated the regulatory effects of anti-CD3L1. These findings further elucidate the direct modulation of TAM polarization by anti-CD3L1 through the CD3L1-NRP2 signaling axis.

Taken together, our data suggest that, in addition to the previously discovered receptor CD3ε, CD3L1 also interacts with NRP2, a significant receptor that is widely expressed by monocytes/macrophages. Furthermore, the anti-CD3L1 antibody effectively blocks the CD3L1-NRP2 interaction, leading to the downregulation of M2 TAM polarization and function. The impact of anti-CD3L1 treatment on the induced M2 TAMs was consistent with NRP2 knockdown, and NRP2 was crucial for anti-CD3L1 treatment to take effect, indicating the pivotal role of CD3L1-NRP2 interaction.

### In vivo enhancement of anti-tumor macrophage function via anti-CD3L1 therapy in osteosarcoma models

**Figure 6:**
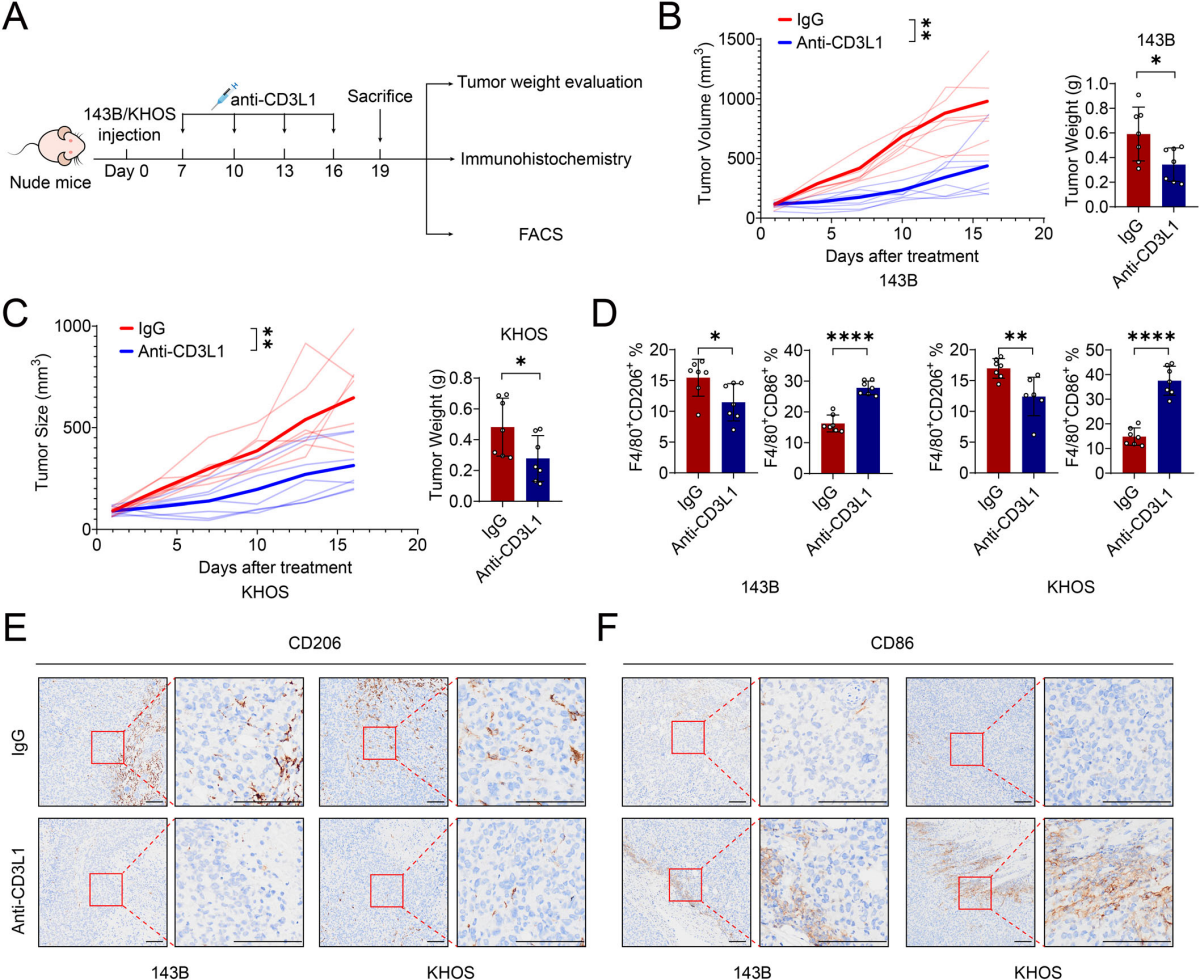
In vivo retardation of osteosarcoma growth with downregulation of M2 TAMs via anti-CD3L1 therapy in nude mice model. (**A**) Schematic description of the design of in vivo experiments. (**B**, **C**) Tumor volume curve and tumor weight measurement showing significant tumor growth retardation of (**B**) 143B, (**C**) KHOS by 10 mg/kg anti-CD3L1 in comparison with 10 mg/kg isotype human IgG treatment in xenograft nude mouse models (*n* = 7 mice per group). (**D**) Flow cytometry of macrophages isolated from the tumors of the xenograft nude mouse models showing a substantial decrease in F4/80^+^CD206^+^ M2 TAMs and an increase in F4/80^+^CD86^+^ M1 TAMs after 10 mg/kg anti-CD3L1 treatment in comparison with 10 mg/kg isotype human IgG treatment (*n* = 7 independent samples per group). (**E**, **F**) IHC staining results showing the substantial differences of the markers of (**F**) M1 (CD86) and (**E**) M2 (CD206) TAMs infiltrated in the 143B/KHOS TME by human 10 mg/kg human IgG treatment and 10 mg/kg anti-CD3L1 treatment in nude mice (*n* = 7 independent samples per group) under ×5 and ×20 microscopic views (scale bar = 100 μm). *P < 0.05, **P < 0.01, ****P < 0.0001. Data are mean ± s.d. (**B**–**D**) Two-tailed unpaired Student’s *t* test. Exact *P* values: (**B**) Tumor volume: *P* = 0.0046; Tumor weight: *P* = 0.0252. (**C**) Tumor volume: *P* = 0.0044; Tumor weight: *P* = 0.0441. (**D**) 143B: F4/80^+^ CD86^+^ control vs treated: *P* = 1.6503363511E-06; F4/80^+^ CD206^+^ control vs treated: *P* = 0.0292; KHOS: F4/80^+^ CD86^+^ control vs treated: *P* = 1.4443459295E-06; F4/80^+^ CD206^+^ control vs treated: *P* = 0.0049. Source data are available online for this figure.

**Figure EV6:**
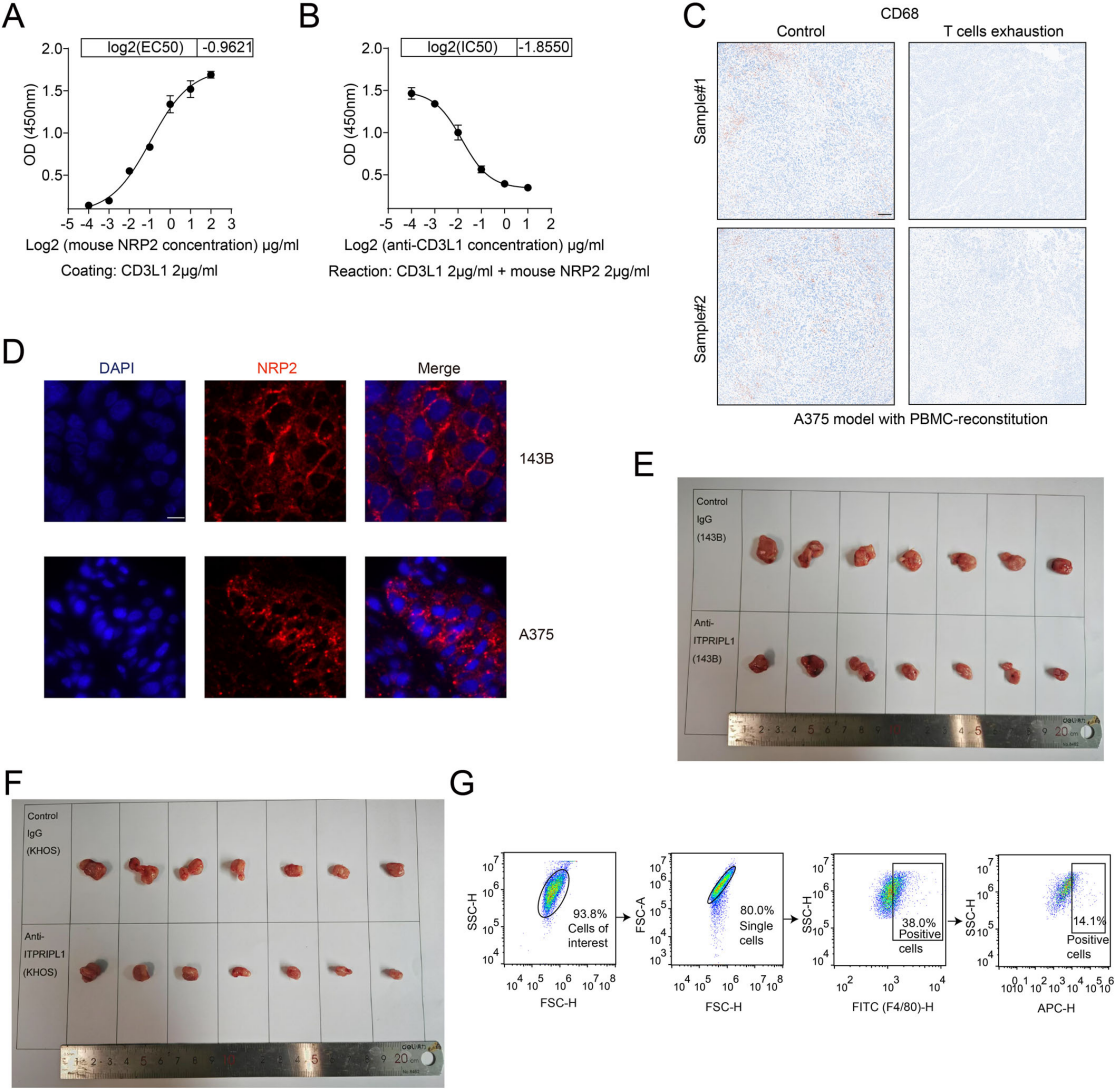
Supplementary information of the in vivo experiments related to Fig. 6. (**A**, **B**) ELISA binding curve showing great affinity between human CD3L1 and mouse NRP2 protein, which can be blocked by anti-CD3L1 antibody (*n* = 3 independent wells). (**C**) IHC staining of the human macrophage marker CD68 in the A375 transplant samples in our previous PBMC-reconstituted model under ×5 and ×20 microscopic views, showing a significant decrease in CD68-positive cells after T-cell exhaustion (*n* = 3 independent samples, scale bar = 50 μm). (**D**) Representative images showing the expression of NRP2 in the 143B (osteosarcoma) and A375 (melanoma) transplant samples under ×60 microscopic view (*n* = 3 independent samples) (scale bar = 10 μm). (**E**, **F**) Macroscopic views of the (**E**) 143B (**F**) KHOS tumors isolated from nude mice tumor transplant (*n* = 7 mice per group). (**G**) The gating strategy of the isolated immune cells infiltrated in the tumor microenvironment of the nude mice: cells were first gated by FSC-H/SSC-H to select out the main cell populations, and then gated by FSC-A/FSC-H to select out the single cells, and then gated by FITC-H/SSC-H to select out the F4/80-positive populations, and then gated by APC-H/SSC-H to select out the CD86/CD206-positive groups. Data are mean ±  s.d.

To further investigate the in vivo effects of anti-CD3L1 in the context of T lymphocyte deficiency in osteosarcoma models, and given the pivotal role of TAMs modulation in these specific tumor types, we utilized nude mice to establish our tumor implantation model (Fig. 6A). Prior to the model building, The strong interaction between mouse NRP2 and human CD3L1 protein (which can be effectively blocked by the anti-CD3L1 antibody) was validated prior to the model building (Fig. EV6A,B).

Our previous findings have shown that in tumor models such as A375 melanoma transplants, where cytotoxic T cells play a dominant role, the depletion of T cells leads to a significant reduction in the therapeutic efficacy of anti-CD3L1 treatment (Deng et al, 2024). Upon re-evaluating our A375 models, we conducted IHC staining of tumor samples using CD68 as a marker of macrophages. Our findings indicate that following the depletion of CD4^+^ and CD8^+^ T cells in PBMC-reconstituted NPSG mice, there was a significant reduction in the infiltration of functional macrophages (Fig. EV6C). It is likely attributable to the inherent limitation that the PBMC-reconstituted humanized immune mouse model fails to perfectly replicate the natural immune microenvironment. This phenomenon may be attributed to the limitations of immune reconstitution in accurately replicating the natural clinical microenvironment. Specifically, given the intricate interplay between T cells and macrophages within the tumor microenvironment, the exclusion of T cells may compromise macrophage survival in the PBMC-reconstituted NPSG model. We also evaluated the NRP2 expression in the A375 (melanoma) and 143B (osteosarcoma) TME (Fig. EV6D). Furthermore, in contrast to the melanoma microenvironment, the osteosarcoma microenvironment exhibits a greater reliance on the anti-tumor immune function mediated by macrophages, whereas T cells contribute relatively minimally to anti-tumor immune responses. In osteosarcoma models implanting 143B or KHOS cells in nude mice, both exhibited notable tumor retardation following anti-CD3L1 treatment in the context of lack of T cells, indicating the promotion of anti-tumor response by anti-CD3L1 antibody other than T-cell rejuvenation (Figs. 6B,C and EV6E,F). Through a comprehensive single-cell mRNA sequencing analysis of the immune cells isolated from the osteosarcoma tumor samples, sorted by CD45 microbeads, we gained further insights into the mechanisms underlying these observations (Figs. 2A–G and EV2A–D). Our analysis revealed a substantial increase in the proportion of M1 macrophages, accompanied by a remarkable decrease in M2 TAMs, with activation of the M1 TAMs signaling pathways and inhibition of the M2 TAMs functions. A deeper examination of the single-cell sequencing data further indicated a significant downregulation in the expression of M2 signature genes with upregulation of the M1 signature genes, suggesting that anti-CD3L1 treatment negatively modulates the M2 TAMs polarization and function, which contributed to an immunoactivated TME, enhancing anti-tumor responses. In addition, we analyzed the proportions of M1 and M2 TAMs within the TME using flow cytometry and immunohistochemical staining (Figs. 6D–F and EV6G). By assessing the F4/80^+^ population with CD86^+^/CD206^+^ markers, we found a significant increase in M1 TAMs and a decrease in M2 TAMs after anti-CD3L1 treatment. These results combined with the single-cell analysis data further confirmed that anti-CD3L1 can promote the anti-tumor immune response by modulating M2 TAMs when T cells were absent in this specific scenario.

**Figure 7:**
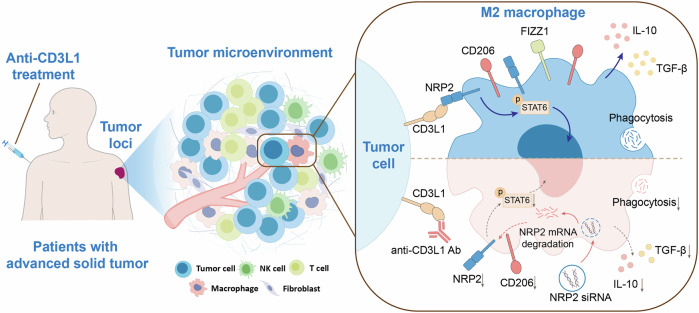
CD3L1 interacted with NRP2 to promote M2 TAM polarization and function within TME, which can be counteracted by anti-CD3L1 treatment. Schematic description of the pivotal role of blocking CD3L1-NRP2 interaction in the negative regulation of M2 TAMs by anti-CD3L1 in our hypothesis.

In conclusion, these results strongly suggest that through the negative modulation of M2 TAMs within the TME, anti-CD3L1 treatment demonstrates significant therapeutic efficacy in osteosarcoma transplant models. The proposed mechanism underlying this regulatory network is depicted in Fig. 7. These findings contribute to a deeper understanding of the mechanisms by which anti-CD3L1 exerts its antitumor effects in the context of lymphocyte deficiency, highlighting the potential of this therapeutic approach in the treatment of osteosarcoma.

## Discussion

Our findings reveal a dual immunoregulatory role for CD3L1 in shaping the osteosarcoma tumor microenvironment (TME). While prior work established CD3L1 as a CD3ε-binding ligand that dampens T-cell activation, we now identify a *hitherto unrecognized* mechanism: CD3L1 interacts with Neuropilin-2 (NRP2) to skew tumor-associated macrophages (TAMs) toward an immunosuppressive M2 phenotype. This axis creates a permissive niche for immune evasion, particularly in osteosarcoma—a malignancy characterized by dense M2 TAM infiltration and scant cytotoxic T-cell presence, features that likely contribute to its historically poor immunotherapy responses. Critically, our data demonstrate that anti-CD3L1 therapy not only restores T-cell functionality but also disrupts M2 TAM polarization, providing mechanistic insight into its efficacy in osteosarcoma models. These results position CD3L1 as a nexus linking adaptive and innate immune suppression in the TME, offering a plausible explanation for how targeting this molecule overcomes the immune-resistant nature of osteosarcoma.

Recent research has extensively examined the positive feedback loop between M1 TAMs and cytotoxic T cells (van Elsas et al, 2024; Garrido-Martin et al, 2020). The presence of functional M1 TAMs is recognized as a pivotal step in activating cytotoxic T cells, whereas M2 TAMs significantly impede the regulatory network between M1 TAMs and cytotoxic T cells. The synergistic effect of promoting M1 TAM polarization and activating cytotoxic T cells further underscores the potential of CD3L1 as a therapeutic target in tumor immunotherapy.

Our prior research has delineated the function of CD3L1 as a natural inhibitory ligand of CD3ε (also known as CD3 ligand 1, CD3L1) in suppressing T-cell function. In this study, we identified NRP2 as another crucial receptor for CD3L1. Prior studies have shown that NRP2 promotes M2 TAM polarization and function, including enhancing efferocytosis by M2 TAMs. Anti-CD3L1 treatment significantly blocks the CD3L1-NRP2 interaction, resulting in the downregulation of downstream signaling phosphorylation, secretion of functional cytokines, expression of signature genes and proteins, and efferocytotic function of M2 TAMs. The blockade of the CD3L1-NRP2 interaction and subsequent downregulation of M2 TAM polarization and function provide additional support for the therapeutic potential of anti-CD3L1 in tumor immunotherapy.

The CD3L1-NRP2 axis represents a novel regulatory pathway for M2 TAMs, characterized by distinct mechanisms that diverge from well-established pathways governing M2 TAM polarization, such as CSF1R or CD47-SIRPα signaling. While CSF1R signaling activates PI3K-AKT and MAPK cascades to promote anti-inflammatory cytokine secretion (e.g., IL-10, TGF-β) and M2 polarization, the CD47-SIRPα axis functions as an inhibitory “do not eat me” signal, suppressing phagocytosis by macrophages. In contrast, the CD3L1-NRP2 axis directly contributes to M2 TAM polarization and functional activation, potentially through mechanisms involving cytokine modulation, metabolic reprogramming, or cell–cell interactions. Notably, therapeutic targeting of CD3L1 may synergize with existing anti-M2 TAM strategies (e.g., CSF1R inhibitors or CD47 blockers) to enhance macrophage reprogramming and restore anti-tumor immunity in the TME. Further mechanistic studies are warranted to elucidate the precise signaling networks and crosstalk between the CD3L1-NRP2 axis and classical TAM-regulatory pathways.

In T cell-deficient settings, our study provides a scientifically rigorous explanation for the pivotal link between CD3L1-NRP2-TAM modulation and effective tumor control. Single-cell analysis revealed that macrophages constitute the most significantly altered cell population in the treatment group. Among CD45^+^ immune cells, macrophages were the predominant subset, indicating their central role in the immune response within the tumor microenvironment. Further, based on previous studies, macrophages function as the main regulators of the remaining immune cells, orchestrating their activation, differentiation, and functional polarization (Wang et al, 2026; Zou et al, 2025; Yang et al, 2025). This regulatory capacity positions macrophages as critical mediators of the anti-tumor immune response, especially in settings where T cell function is compromised. In addition, given the well-established association between M2 macrophage polarization and adverse clinical outcomes in cancer, strategies aimed at reprogramming these macrophages towards a pro-inflammatory phenotype emerge as promising therapeutic avenues (Yang et al, 2025; Ni et al, 2025; Goddard et al, 2024; Zhang et al, 2024; Yang et al, 2020). Currently, we are actively engaged in model experiments designed to independently modulate macrophages alongside other immune cell types, with the aim of dissecting their specific contributions to tumor suppression; the results of these ongoing investigations will be detailed in forthcoming reports, ultimately aiming at overcoming the challenges posed by T-cell deficiency in clinical cancer treatment.

However, it is important to acknowledge the limitations of the nude mouse model, which, despite its utility in initial investigations of CD3L1-NRP2-mediated macrophage modulation, introduces confounding factors that undermine its physiological relevance and translational applicability. The absence of functional adaptive immunity in nude mice creates an artificial environment where macrophage behavior may be influenced by compensatory mechanisms that do not occur in immunocompetent hosts. The model is also compounded by incomplete characterization of cross-species receptor-ligand interactions, such as human CD3L1 binding to murine NRP2, which may exhibit altered affinity or downstream signaling compared to human-human interactions, thereby introducing unquantified variability into experimental outcomes. The interpretation of findings, such as the observed reduction in macrophage infiltration following CD4^+^/CD8^+^ T-cell depletion in A375 tumors, should be careful in this context, as it may reflect incomplete immune reconstitution and artificial synergies between T cells and macrophages in xenogeneic settings rather than a direct regulatory mechanism. Similarly, the inconsistent reliance on TAMs between melanoma and osteosarcoma models underscores how tumor type-specific factors can interact with model-inherent artifacts to produce divergent results.

Collectively, these limitations suggest that while CD3L1-NRP2 may influence macrophage function in xenograft models, the artificiality of these systems precludes definitive conclusions about its role in human tumors, particularly regarding T-cell-independent mechanisms. To address these gaps, future studies should prioritize syngeneic models with intact immune systems. Additionally, more thorough mechanistic dissections are essential to distinguish direct CD3L1-NRP2 effects from indirect confounders.

In conclusion, this study identifies NRP2 as another significant receptor for CD3L1 and further elucidates the modulation of M2 TAMs and the interplay between M2 TAMs and T cells by anti-CD3L1 treatment within the TME, particularly in osteosarcomas. These findings contribute to a deeper understanding of the pivotal role of CD3L1 in immune evasion within the TME and provide valuable insights for guiding anti-CD3L1 treatment in clinical trials.

## Methods

**Table:** Reagents and tools table

Reagent/resource	Reference or source	Identifier or catalog number
**Experimental models**		
Human melanoma patient samples	Cancer Hospital Chinese Academy of Medical Sciences	N/A
Nude mouse	Shanghai Lab. Animal Research Center	N/A
OT-I mouse	GemPharmatech Co., Ltd	N/A
OT-II mouse	GemPharmatech Co., Ltd	N/A
C57 mouse	Shanghai Lab. Animal Research Center	N/A
Jurkat cell line	ATCC	TIB-152
THP-1 cell line	ATCC	TIB-202
KHOS cell line	Zan Shen’s Lab (Shanghai Sixth People’s Hospital)	N/A
143B cell line	Zan Shen’s Lab (Shanghai Sixth People’s Hospital)	N/A
PBMCs	Mt-bio	N/A
293 T cell line	Zhigang Lu’s Lab (Fudan University)	N/A
**Recombinant DNA**		
CD3L1-ECD/ICD-Flag	Shanghai Generay Biotech Co., Ltd	N/A
NRP2-HA	Shanghai Generay Biotech Co., Ltd	N/A
**Antibodies**		
Anti-CD3L1(h) antibody	Biotroy Therapeutics	N/A
Anti-CD3L1(m) antibody	Biotroy Therapeutics	N/A
HRP-conjugated mouse anti-human GAPDH antibody	Aksomics	KC-5G5
Rabbit anti-human NFKB p65 antibody	CST	8242
Rabbit anti-human p-NFKB p65 (Ser536) antibody	CST	3033
Rabbit anti-human p-ERK antibody	CST	4370
Rabbit anti-human ERK antibody	CST	4695
Rabbit anti-human STAT6 antibody	CST	5397
Rabbit anti-human p-STAT6 antibody	CST	56554
Human IgG1 isotype control	InvivoMab	BE0297
HRP-conjugated goat anti-mouse IgG (H + L) antibody	Aksomics	KC-MM-035
HRP-conjugated goat anti-rabbit IgG (H + L) antibody	Aksomics	KC-RB-035
HRP-conjugated goat anti-human Fcγ antibody	Jackson ImmunoResearch	109-035-098
Anti-CD206 antibody	CST	24595
Anti-CD68 antibody	CST	97778
Anti-CD11b antibody	Abcam	ab52478
Anti-CD86 antibody	CST	91882
Anti-CD3 antibody	Invitrogen	16-0037-81
Anti-CD28 antibody	Invitrogen	16-0289-81
Anti-NRP2 antibody	CST	98780
Rabbit anti-HA antibody	CST	3724
Mouse anti-Flag antibody	Abcam	Ab18230
Anti-human CD68-FITC antibody	Invitrogen	11-0689-42
Anti-mouse CD206-APC antibody	Invitrogen	17-2061-82
Anti-mouse CD86-APC antibody	Invitrogen	17-0862-82
Anti-mouse F4/80-FITC antibody	Invitrogen	11-4801-82
Anti-human CD206-APC antibody	Invitrogen	17-2069-42
Anti-human CD86-APC antibody	Invitrogen	17-0869-42
Anti-mouse CD69-APC antibody	Invitrogen	17-0691-80
Anti-mouse CD25-FITC antibody	Invitrogen	11-0251-82
**Oligonucleotides and other sequence-based reagents**		
PCR primers	This study	Table EV1
si Control/NRP2	Genepharm	Table EV2
**Chemicals, enzymes, and other reagents**		
Mouse PBMC isolation kit	Solarbio	P6340
DAB staining kit	Solarbio	DA1010
Human TGF-β kit	Liankebio	EK981
Mouse IL-10 kit	Liankebio	EK210
Mouse TGF-β kit	Liankebio	EK9162
Mouse IL-2 kit	Liankebio	EK202
Mouse IFN-γ kit	Liankebio	EK280
CCK8 kit	Solarbio	CA1210
Human IL-10 kit	Liankebio	EK110
PLA (Duolink) kit	Sigma	DUO92101
BCA kit	Thermo scientific	23227
Mouse PBMC isolation kit	Solarbio	P6340
DAB staining kit	Solarbio	DA1010
Human TGF-β kit	Liankebio	EK981
Mouse IL-10 kit	Liankebio	EK210
Mouse TGF-β kit	Liankebio	EK9162
Mouse IL-2 kit	Liankebio	EK202
Mouse IFN-γ kit	Liankebio	EK280
CCK8 kit	Solarbio	CA1210
Human IL-10 kit	Liankebio	EK110
PLA (Duolink) kit	Sigma	DUO92101
BCA kit	Thermo scientific	23227
Mouse PBMC isolation kit	Solarbio	P6340
DAB staining kit	Solarbio	DA1010
Human TGF-β kit	Liankebio	EK981
Mouse IL-10 kit	Liankebio	EK210
Mouse TGF-β kit	Liankebio	EK9162
Mouse IL-2 kit	Liankebio	EK202
Mouse IFN-γ kit	Liankebio	EK280
CCK8 kit	Solarbio	CA1210
Human IL-10 kit	Liankebio	EK110
PLA (Duolink) kit	Sigma	DUO92101
BCA kit	Thermo scientific	23227
Mouse PBMC isolation kit	Solarbio	P6340
DAB staining kit	Solarbio	DA1010
Human TGF-β kit	Liankebio	EK981
Mouse IL-10 kit	Liankebio	EK210
Mouse TGF-β kit	Liankebio	EK9162
Mouse IL-2 kit	Liankebio	EK202
Mouse IFN-γ kit	Liankebio	EK280
CCK8 kit	Solarbio	CA1210
Human IL-10 kit	Liankebio	EK110
PLA (Duolink) kit	Sigma	DUO92101
BCA kit	Thermo scientific	23227
Mouse PBMC isolation kit	Solarbio	P6340
DAB staining kit	Solarbio	DA1010
Human TGF-β kit	Liankebio	EK981
Mouse IL-10 kit	Liankebio	EK210
Mouse TGF-β kit	Liankebio	EK9162
Mouse IL-2 kit	Liankebio	EK202
Mouse IFN-γ kit	Liankebio	EK280
CCK8 kit	Solarbio	CA1210
Human IL-10 kit	Liankebio	EK110
PLA (Duolink) kit	Sigma	DUO92101
BCA kit	Thermo scientific	23227
Mouse PBMC isolation kit	Solarbio	P6340
DAB staining kit	Solarbio	DA1010
Human TGF-β kit	Liankebio	EK981
Mouse IL-10 kit	Liankebio	EK210
Mouse TGF-β kit	Liankebio	EK9162
Mouse IL-2 kit	Liankebio	EK202
Mouse IFN-γ kit	Liankebio	EK280
CCK8 kit	Solarbio	CA1210
Human IL-10 kit	Liankebio	EK110
PLA (Duolink) kit	Sigma	DUO92101
CD45 microbeads	Miltenyi	130-045-801
CD4/CD8 microbeads	Miltenyi	130-116-480
BCA kit	Thermo scientific	23227
**Software**		
ImageJ	NIH	https://imagej.nih.gov/ij/
Prism 8	GraphPad Software	https://www.graphpad.com/scientific-software/prism/
FlowJo V10	TreeStar	https://www.flowjo.com/
R studio	R Core	https://www.r-project.org/
TIDE	Dana Farber Cancer Institute & Harvard University	https://tide.dfci.harvard.edu/
scCancerExplorer	Nanjing Medical University	https://bianlab.cn/scCancerExplorer/explore/search
TIMER	Dana Farber Cancer Institute & Liu Lab	http://cistrome.dfci.harvard.edu/TIMER/
Cytoscape	National Resource for Network Biology	https://cytoscape.org/
R programming language	Bioconductor	https://bioconductor.org/
Seurat	Satija Lab	https://satijialab.org/seurat/

### Experimental model and study participant details

#### Cell lines

The origins of the cell lines are stated in the reagents table and all are authenticated and mycoplasma-free. THP-1 and PBMCs were incubated in RPMI-1640 (Meilunbio) with 10% FBS (Gibco). 143B and KHOS cells were incubated in DMEM (Meilunbio) with 10% FBS (Gibco). All cells were cultured at 37 °C under 5% CO_2_.

#### Anti-CD3L1 treatment in nude mouse models

Ethics approval was granted from Fudan University (No.2025-02-SY-DSY-07). All animal experiments were performed in strict accordance with ARRIVE guidelines and the relevant ethical guidelines, approved by the Department of Laboratory Animal Science of Fudan University and the Institutional Animal Care. The origins of mice are stated in the reagents table. Mice were housed in the SPF-grade Laboratory Animal Building of Fudan Zhangjiang Campus. After one week of adaptation to the environment, each individual nude mouse (female, 4 weeks old) was randomized into two groups (*n* = 7 for each group) and injected 1.0 × 10^7^ pre-treated KHOS/143B cells subcutaneously in the right flank. Since Day 7 after inoculation, the tumor sizes were recorded every 3 days using a vernier caliper and calculated with the formula 1/2 × A × a^2^ (A and a, respectively, denote the length and the width of the tumor). We treated one group of the mice models with 10 mg/kg human IgG1 isotype control and the other with anti-CD3L1 from the measurement day every three days, four times in total. In accordance with the ethical guidelines, mice would be sacrificed once the tumor volume reached 2 cm^3^ or ulcers appeared. In this experiment, all the mice were sacrificed simultaneously when the average tumor volume of the control group approached 2 cm^3^ and the tumors were resected and weighed, and pre-treated for further experiments.

#### Humanized PBMC anti-CD3L1 treatment mouse models

All animal experiments were performed in strict accordance with ARRIVE guidelines and the relevant ethical guidelines, approved by the Department of Laboratory Animal Science of Fudan University and the Institutional Animal Care. The origins of mice are stated in the reagents table. Mice were housed in the SPF-grade Laboratory Animal Building of Fudan Zhangjiang Campus. After one week of adaptation to the environment, each individual NPSG mouse (female, 5 weeks old) was randomized into two groups (*n* = 6–8 for each group) and injected 1.8 × 10^6^ pre-treated KHOS/143B cells with 6.0 × 10^5^ PBMCs subcutaneously in the right flank. Since Day 7 for the tumors after inoculation, the tumor sizes were recorded every 2–3 days using a vernier caliper and calculated with the formula 1/2 × A × a^2^ (A and a, respectively, denote the length and the width of the tumor). We treated one group of the mice models with 10 mg/kg human IgG1 isotype control and the other with anti-CD3L1 from the measurement day every three days, four times in total. In accordance with the ethical guidelines, mice would be sacrificed once the tumor volume reached 2 cm^3^ or ulcers appeared. In this experiment, all the mice were sacrificed simultaneously when the average tumor volume of control group approached 2 cm^3^ and the tumors were resected and weighed, and pre-treated for further experiments.

#### Human clinical trial samples

Ethics approval was granted from Fudan University (No. 2024-34-S), and informed consent was obtained from all participants. All experiments involving human clinical trial (NCT06404905) samples were conducted in strict adherence to the pertinent ethical guidelines, which had been approved by the Clinical Trials Center of the Chinese Academy of Medical Sciences and Peking Union Medical College. The experiments conformed to the principles set out in the WMA Declaration of Helsinki and the Department of Health and Human Services Belmont Report. The presented samples were from a patient diagnosed with primary melanoma refractory to PD-L1/PD-1 treatment who was then enrolled in Phase I of the clinical trial for anti-CD3L1 treatment. Following informed consent, tumor sites were biopsied using a fine needle aspiration technique from multiple angles, both prior to and following the administration of anti-CD3L1 treatment. The collected samples underwent pre-treatment procedures to facilitate subsequent experimental analyses.

### Method details

#### Antibody dilutions

The dilution ratio of antibodies:

Applied for FACS: 1:500. Applied for WB: primary antibodies: 1:1000; secondary antibodies: 1:5000. Applied for ELISA: 1:20000. Applied for IHC: 1:800. Applied for IF: primary antibodies: 1:500; secondary antibodies: 1:1000. Applied for T-cell activation: 1:1000.

#### Flow cytometry

After TAM induction, the cells (2 × 10^6^/ml) were transferred 100 μl per well into a flat-bottom 96-well plate (Costar). We incubated the plate at 37 °C under 5% CO_2_ for 30 min. After incubation, we washed the samples with flow cytometry staining buffer (Invitrogen) three times. Then we diluted the fluorescent antibodies at the suggested concentrations according to the suppliers in staining buffer and added 200 μl to each sample. We incubated them on ice for 30 min, protected from light. Next, after being washed with staining buffer three times, we transferred the samples into single tubes (Falcon) and analyzed them by Cytoflex (Beckman). We applied FlowJo V10 to analyze the data.

#### Single-cell sequencing

Two sets of tumor samples (each containing three independent samples) were dissected from nude mice and stored in the tissue storage fluid (Miltenyi, 130-100-008). The tissues were then smashed and filtered, and the immune cells were isolated from the tumor tissues using CD45 magnetic beads. The sorted cells were centrifuged at 1000 rpm for 5 min and washed with PBS three times. The cells were proceeded with vitality test (more than 90%) and a quality control process. 10x Genomics Chromium system was used to mix gel beads containing Barcode information with samples to form gel beads-in-emulsions (GEMs). After anti-transcription of the GEMs, Barcode-labeled cDNA was formed. After PCR, digestion, and fragment optimization of the cDNA, a standard DNA sequencing library was established. Then, high-throughput sequencing was performed on the established library through the Illumina platform.

#### Single-cell sequencing data analysis

The raw sequencing data were pre-processed through the Cell Ranger pipeline (v7.1.0, 10×Genomics) by the GENEWIZ company, which outputs matix.mtx.gz files and gzipped TSV files with feature and barcode sequences corresponding to row and column indices, respectively. The feature-barcode matrix of UMI counts was then analyzed with Seurat (v5.1.0) for quality control, normalization, batch effect removal, dimensional reduction, clustering, and visualization. For two samples, the following criteria were applied for quality control: unique feature counts between 200 and 6000, and mitochondrial gene percentage <5%. The count matrix was log-normalized, and the top 2000 most variable genes were identified for selecting integration anchors. Integration removed batch effects with the canonical correlation analysis (CCA), which used the anchors to correct technical differences between samples and to perform comparative scRNA-seq analysis across experimental conditions. The integrated matrix was then scaled, and the top 10 dimensions resulting from the principal component analysis (PCA) were used for the uniform manifold approximation and projection (UMAP). Meanwhile, the shared nearest neighbor graph-based clustering was performed on the PCA-reduced data to identify cell clusters. The resolution was set to 0.2 to obtain the major cell types of the samples. The cell type marker genes were provided by the FindConservedMarkers function. The cell cluster annotations were based on the expression of canonical marker genes. The data were statistically analyzed by Wilcox to draw a *P* value and an adjusted *P* value for each different gene to support our conclusion. The changes in the Macrophages are shown in Dataset EV1.

#### M2-phenotype TAMs induction and tumor cell co-culture

THP-1 cells or human PBMCs were counted and seeded at a concentration of 100,000 cells/ml in antibiotic-free RPMI-1640 media + 10% FBS and maintained at 37 °C, 5% CO_2_ in a humidified tissue culture incubator.

THP-1 cells were differentiated into macrophages by 24-hr incubation with 30 ng/ml phorbol 12-myristate 13-acetate (PMA) followed by 24-hr incubation in PMA-free RPMI-1640 medium. PBMCs were differentiated into macrophages by 24-h incubation with 30 ng/ml M-CSF followed by 24-hr incubation in M-CSF-free RPMI-1640 medium. Then, the macrophage M2 polarization was obtained by incubation with 20 ng/ml of IL-4 and 20 ng/ml of IL-13 for 48 h for THP-1 cells or 20 ng/ml of IL-4 and 20 ng/ml of IL-10 for 48 h for PBMCs. After induction, each well of induced M2 TAMs was co-cultured with 143B cells in 1:1 ratio and treated with different concentrations of control IgG or anti-CD3L1 antibody for 24 h. The mixture was then sorted by CD45 microbeads to isolate the CD45^+^ cells (induced macrophages) for further experiments.

#### Immunofluorescence

The tumor samples were fixed with 4% PFA for 24 h and embedded by paraffin and cut into slides. The slides were first heated for 2 h and sequentially washed by xylene, 100% ethanol, 90% ethanol, 70% ethanol, 50% ethanol, PBS, each for 10 min. The slides were then repaired with a sodium citrate solution by heat. Autofluorescence removal was achieved by putting the slides in 1 mg/mL NaBH4 in PBS for 10 min, three times. Then the tumor specimens were incubated with primary antibodies against CD3L1/CD45/CD68/CD8/NRP2 at proper concentrations under 4 °C overnight. After three times PBS washing, the tissues were then incubated with secondary antibodies against different species conjugated with different fluorophores at 37 °C for 30 min, followed by PBS washing for five times. The slides were sealed with DAPI in glycerol. After drying, the slides were observed under a confocal microscope.

#### BMDM harvest and induction

C57BL/6 mice were euthanized to collect the femurs and tibias, which were then cleaned of surrounding muscle tissue. Sterile instruments were applied to remove the epiphyses at both ends of the bones, exposing the marrow cavity, and then the bone marrow cells were flushed out of the cavity using a sterile syringe filled with PBS into a collection tube. The cell suspension was transferred into a cell strainer to obtain a single-cell suspension and then red blood cells were lysed using the red blood cell lysis buffer and then washed with PBS. The bone marrow cells were then plated in culture dishes containing medium supplemented with a macrophage colony-stimulating factor (M-CSF) to induce differentiation. Every 2 days, replace the medium with fresh M-CSF/GM-CSF-containing medium to remove non-adherent cells and promote macrophage maturation. After 7–10 days of culture, the adherent cells are mature BMDMs, which can be harvested for further experiments.

#### Antigen presentation evaluation

OT-I/OT-II mice were sacrificed, and CD8^+^/CD4^+^ T cells were isolated from the blood using the mouse PBMC isolation kit and sorted by CD8/CD4 microbeads. The cells were cultured in complete RPMI-1640 supplemented with 1 μg/ml anti-CD3/CD28 antibodies to be stimulated for 7 days. The mature BMDMs were first pre-treated with OVA257-264/OVA323-339 and 8 μg/ml CD3L1-His protein for 6 h, and then treated with control IgG or different concentrations of anti-CD3L1 antibody for another 6 h. Then the BMDMs were co-cultured with the CD8^+^/CD4^+^ T cells isolated from OT-I/OT-II mice in a 1:1 ratio for 18 h. Then, flow cytometry was performed to evaluate the activation status of the T cells by assessing the expression of activation markers CD25 and CD69, a CCK-8 assay was employed to evaluate the growth curve of the T cells, and mouse IFNγ/IL-2 kits were used to evaluate the cytokine secretion.

#### CCK8 assay

After co-culturing with the BMDMs, the T cells were seeded in a 96-well bottom plate at 1 × 10^3^/well. Then the plate was incubated at 37 °C with 5% CO_2_ for 7 days. 10 μL of CCK-8 reagent was added to each well. The plate was then incubated for an additional 4 h and placed in SpectraMax i3x and read at 450 nm every day (repeated seven times).

#### NRP2 knockdown of THP-1 cells

The sequences of the siRNAs are shown in Table EV2. The siRNA-NRP2 vector was synthesized by Genepharma, Shanghai. After the induction of THP-1 cells by PMA and IL-4/IL-13, the 2 μg siRNAs were transfected into the THP-1 cells with 6 μl Fugene SI for each well of THP-1 cells for 48 h. After treatment, each well of the siRNA-transfected M2 TAMs was treated with different concentrations of control IgG or anti-CD3L1 antibody.

#### Efferocytosis detection

Apoptosis was induced in Jurkat cells through the application of 50 mmol/L etoposide, with the cells being maintained at 37 °C, 5% CO_2_ for a duration of 12 h. This treatment protocol typically resulted in the induction of apoptosis in 80–90% of the cells. Subsequently, the cells were centrifuged at 2000 rpm for 10 min, and the cell pellet was washed twice with PBS. The cells were then incubated with pHrodo Green Maleimide, adhering strictly to the manufacturer’s instructions. Following incubation, the cells were once again centrifuged at 2000 rpm for 10 min. The pellet was washed twice with PBS and resuspended in RPMI-1640 medium. In parallel, THP-1 cells, which had been induced as described previously, were plated in eight-well chambers with 10 μg/ml CD3L1 protein. Apoptotic Jurkat cells were then added to the macrophages at a target-to-effector ratio of 10:1 for a period of 1 h (referred to as the pulse phase) at 37 °C. Phagocytosis was halted by the addition of ice-cold PBS, and the cells were rigorously washed with ice-cold PBS to eliminate any non-phagocytosed or loosely bound cells. Fresh, complete RPMI-1640 medium was added to the cells, and the clearance of apoptotic cells was monitored over the specified time period (referred to as the chase phase). The slides were then sealed with DAPI and analyzed using confocal microscopy. The images were analyzed by ImageJ for the corrected total cellular fluorescence.

#### Immunohistochemistry

The tumor samples were fixed with 4% PFA for 24 h and embedded in paraffin and cut into slides. The slides were first heated for 2 h and sequentially washed by xylene, 100% ethanol, 90% ethanol, 70% ethanol, 50% ethanol, PBS, each for 10 min. Then the slides were tapped with 3% perhydrol for 15 min and washed with PBS for 5 min, three times. The slides were then repaired with a sodium citrate solution by heat. Then the murine tumor specimens were incubated with antibodies against CD86/CD206. The human tumor specimens were incubated with antibodies against CD206. After three PBS washes, the tissues were then incubated with a biotin-conjugated secondary antibody, followed by avidin-biotin–peroxidase complex. The slides were sealed with neutral balsam. The images were all scanned and analyzed by the IHC assessment standard. The cutoff standard was assessed by the IHC assessment standard, that the staining scores can be calculated by multiplying area (1: 0–25%, 2: 26–50%, 3: 51–75%, 4: 76–100%) and intensity (0: negative, no yellow staining; 1: shallow yellow staining; 2: brown yellow staining; 3: dark brown staining), that 0 should be considered as negative, 1–3 as low, 4–8 as medium and 9–12 as high according to pathological studies.

#### Binding and blocking ELISA

The ectodomains of the proteins were diluted in coating buffer (Solarbio) to indicated concentrations and added 100 μl per well into a 96-well ELISA plate (Costar). We placed the plate at 4 °C overnight. We washed the plate with PBST (Applied Cells, Inc.) five times and added 100 μl 5% BSA (VWR Life Sciences) into each well and incubated it at 37 °C for 90 min. We repeated washing and added 100 μl protein of indicated concentrations with or without different concentrations of antibody into each well and incubated at 37 °C for 60 min. For the antibody-blockade study, we coated 1 μg/ml ectodomains of human CD3L1 on the plate, and added 1 μg/ml ectodomains of human or mouse NRP2 simultaneously with different concentrations of anti-CD3L1 antibodies (mouse) together for interaction, and human Fc-HRP secondary antibodies (hFc) were applied to test the NRP2-hFc signal (it was confirmed that there was no cross-interaction between the secondary antibody and human Fc receptors). We repeated washing and added 100 μl associated secondary antibodies conjugated with HRP into each well and incubated at 37 °C for 30 min. After five times PBST washing, we added 100 μl TMB buffer (Invitrogen) into each well and incubated at 37 °C for 15 min, and added 50 μl stop buffer (Abcam) to terminate the reaction. The plate was placed in SpectraMax i3x and read at 450 nm.

#### Immunoblot analysis

Cells were lysed with RIPA buffer (Beyotime) supplemented with 1% proteinase and phosphatase inhibitors cocktail (Thermofisher Scientific). The collected cell lysates were centrifuged for 15 min at 12000 rpm (4 °C). The supernatant was reserved, and the protein concentration was determined by the BCA Protein Assay Kit (Thermofisher Scientific). 5×SDS–PAGE loading buffer (Applied Cells, Inc.) was diluted to 1× with the protein sample and heated at 100 °C for 8 min. The protein extracts were subjected to appropriate concentrations of SDS–PAGE for electrophoresis and transferred to PVDF membranes (Bio-Rad). Membranes were blocked with 5% bovine serum albumin (Thermofisher Scientific) for one hour at room temperature, and then incubated with the primary antibodies overnight at four degrees. Membranes were incubated with secondary HRP-conjugated antibodies (Aksomics) at room temperature for one hour. Before and after the incubation, the membranes were washed five times with TBST and then examined by ChemiDoc imaging system (Bio-Rad). The quantification of the bands were performed by ImageJ (v1.51).

#### Transfection of plasmids

Cells (HEK293) were seeded in six-well plates to reach a density of around 70% at the time of transfection. Twenty-four hours later, transfection was performed using 1.5 mg plasmid together with 4.5 ml FuGENE HD (Promega) and 100 ml Opti-MEM per well according to the manufacturer’s guidance. The negative control in each experiment was cells mock-transfected with an empty control vector.

#### In situ proximity ligation assay

Each sample of 1 × 10^6^ transfected HEK293 cells was fixed with 4% PFA for 15 min at room temperature, and then transferred to eight-well chamber slides (C7182, Sigma). Cells were permeabilized with 0.2% Triton X-100 for 1 h at room temperature. Cells were then treated according to the manufacturer’s instructions with the Duolink kit (Sigma). The antibody combination used was rabbit anti-Flag and mouse anti-HA. Nuclei were stained with DAPI (Sigma). Images were taken at a confocal microscope and analyzed using ImageJ software (v1.51).

#### Cytokine production ELISA

The induced M2 TAMs/CD4^+^ T cells isolated from OT-II mice were centrifuged at 2000 rpm for 20 min. The supernatant was then collected, and the next steps were performed in accordance with the instructions of the suppliers’ ELISA kits.

#### Tumor macrophages harvest

After sacrifice of the mice the resection of the tumors, each tumor was cut 50 mg into 35 mm culture dish immersed in a small amount of RPMI 1640 culture medium. The tumors were cut into small pieces by scissors and transferred into a 5-ml EP tube and digested by 5 ml RPMI 1640 medium + 200 U/ml DNase + 1 mg/ml collagenase IV under 37 °C 220 rpm for 45 min. The mixture was smashed and run through a 100 μm filter into a 50-ml centrifugal tube, titrated by RPMI1640 to reach 20 ml in total. The mixture was centrifuged at 400 rcf for 5 min, and the supernatant was discarded. Then 4 ml 40% percoll was added to resuspend the pellet and transferred into 15 ml centrifugal tubes. The mixture was centrifuged at 2500 rpm for 20 min. The middle white membrane layer was carefully collected and washed by 10 ml PBS, centrifuged at 400 rcf for 5 min, three times. The pellet was resuspended by PBS and sorted by magnetic CD45 beads (Miltenyi). The CD45^+^ cells were harvested and stained with F4/80 with CD86/CD206 antibodies for further experiments.

#### q-PCR

RNA was extracted from induced THP-1 cells using the TRIzol (Invitrogen, 15596018) method following the manufacturer’s instructions. In the RT-qPCR procedure, 1 µg of RNA was first reverse transcribed with the HiScript II Q RT SuperMix (Vazyme, R222-01), and qPCR was subsequently conducted utilizing SYBR Green (Bimake, B21202). Relative mRNA expression levels were calculated by the ΔΔCt method and were normalized to the expression of RPLP0. The primers utilized for gene expression analysis are detailed in Table EV1.

#### Public database analysis

The selection standards of the data were in accordance with each cohort dataset.

For Fig. 2K–N, we investigated the relationships between CD3L1 and macrophage infiltration or IL-10/TGFB1 mRNA expression levels through the following procedures:

Correlation analysis: (1) Spearman’s rank correlation coefficient: Rank transformation: convert the raw mRNA expression values for each gene into ranks, with ties handled by assigning the average rank; Difference calculation: for each paired observation, calculate the difference in ranks between the two genes; Correlation coefficient calculation: compute Spearman’s ρ using the formula based on the sum of squared rank differences; Significance testing: assess the significance of ρ using a *t* test with n-2 degrees of freedom, where n is the number of paired observations, to determine if the observed correlation is significantly different from zero. (2) Pearson’s correlation coefficient: covariance and standard deviations: calculate the covariance between the mRNA expression levels of the two genes and their respective standard deviations; Correlation coefficient calculation: compute Pearson’s *r* as the covariance divided by the product of the standard deviations; Significance testing: use a *t* test to evaluate the significance of *r* to test the null hypothesis of no linear association.

Linear regression analysis: Specify the linear regression model as y =  mx + b + ε, where *y* is the dependent variable (e.g., IL-10 or TGFB1 mRNA expression), *x* is the independent variable (CD3L1 mRNA expression), m is the slope, b is the intercept, and ε is the error term. Use the ordinary least squares method to estimate the slope (m) and intercept (b) by minimizing the sum of squared residuals between the observed and predicted values of *y*. Calculate the coefficient of determination (*R*²) to quantify the proportion of variance in the dependent variable explained by the independent variable. The co-occurrence/mutual exclusion was determined based on the slope (m).

#### Exclusion/inclusion criteria of samples

Sample sizes are indicated in figure legends. Inclusion Criteria: Samples were freshly prepared, free of contamination, and consistent with the experimental design (e.g., cell samples in logarithmic growth phase, tissue samples with intact structure). Exclusion Criteria: Samples with obvious contamination, degradation, or abnormal morphology; samples that did not meet the experimental specifications or failed pre-experiment detection; samples with incomplete experimental records

### Quantification and statistical analysis

Statistical analyses were performed using GraphPad Prism 8.0.2 (GraphPad Software). The statistical significance was set to *P* < 0.05 and represented as *P < 0.05, **P < 0.01, ***P < 0.001, ****P < 0.0001, with ns as no significance, as indicated in the figure legends. The exact numbers (*n*) of each panel are also indicated in the figure legends. Data are mean ± s.d.

To address multiple testing, we applied the Benjamini–Hochberg procedure to control the false discovery rate (FDR) at 5%. Significance was declared for tests with adjusted *P* values ≤ 0.05, balancing Type I and Type II errors.

### Blinding statement

This study employed a double-blind design, where neither the participants nor the researchers involved in data collection, outcome assessment, or intervention delivery were aware of the group assignments (e.g., treatment vs. control). This approach minimizes the risk of bias related to participant expectations or investigator behavior, thereby strengthening the validity and reliability of the study findings.

## Supplementary information

Table EV1

Table EV2

Peer Review File

Dataset EV1

Source data Fig. 1

Source data Fig. 3

Source data Fig. 4

Source data Fig. 5

Source data Fig. 6

Expanded View Figures

## Data Availability

The single-cell sequencing data generated in this study are deposited at GEO and are publicly available as of the date of publication under the accession number GSE304961. The source data of this paper are collected in the following database record: biostudies:S-SCDT-10_1038-S44321-026-00451-3.
