# Supplementary material for: Targeting CD3L1-NRP2 disarms myeloid-driven tumor immune evasion
Source: EMBO Mol Med. 2026 May 15;18(7):2635–66. doi: 10.1038/s44321-026-00451-3 (PMC13365830; doi:10.1038/s44321-026-00451-3)
Supplement: Supplementary file 1 — Table EV1 [file 44321_2026_451_MOESM1_ESM.pdf]

Table EV1. Information of the primers of qPCR, related to Figure 3,4,5 and methods.

Genes	Forward seq	Reverse seq
TGFB1	CTAATGGTGGAAACCCACAACG	TATCGCCAGGAATTGTTGCTG
IL10	GACTTTAAGGGTTACCTGGGTTG	TCACATGCGCCTTGATGTCTG
VEGFA	AGGGCAGAATCATCACGAAGT	AGGGTCTCGATTGGATGGCA
ADM	ATGAAGCTGGTTTCCGTCG	GACATCCGCAGTTCCCTCTT
MRC1	GGGAAAGGTTACCCTGGTGG	GTCAAGGAAGGGTCGGATCG
RETNLB	CCTTCTCATCCTAATCCCCCTT	TGACAGCCATCCCAGCA
