# Supplementary material for: Targeting CD3L1-NRP2 disarms myeloid-driven tumor immune evasion
Source: EMBO Mol Med. 2026 May 15;18(7):2635–66. doi: 10.1038/s44321-026-00451-3 (PMC13365830; doi:10.1038/s44321-026-00451-3)
Supplement: Supplementary file 2 — Table EV2 [file 44321_2026_451_MOESM2_ESM.pdf]

Table EV2. Information of the siRNAs, related to Figure 5 and methods.

Genes	Sequences	
	sense（5'-3'）	antisense（5'-3'）
NRP2 (human)		
NRP2-Homo-3215	GGACAUCCCAGAAAUACAUTT	AUGUAUUUCUGGGAUGUCCTT
NRP2-Homo-2677 (siNRP2 #2)	GCAGCUUUGAGGAUGACAATT	UUGUCAUCCUCAAAGCUGCTT
NRP2-Homo-3517 (siNRP2 #3)	GCCUUAAGCACAAGGUCAATT	UUGACCUUGUGCUUAAGGCTT
