# Supplementary figures and images for: Targeting CD3L1-NRP2 disarms myeloid-driven tumor immune evasion
Source: EMBO Mol Med. 2026 May 15;18(7):2635–66. doi: 10.1038/s44321-026-00451-3 (PMC13365830; doi:10.1038/s44321-026-00451-3)

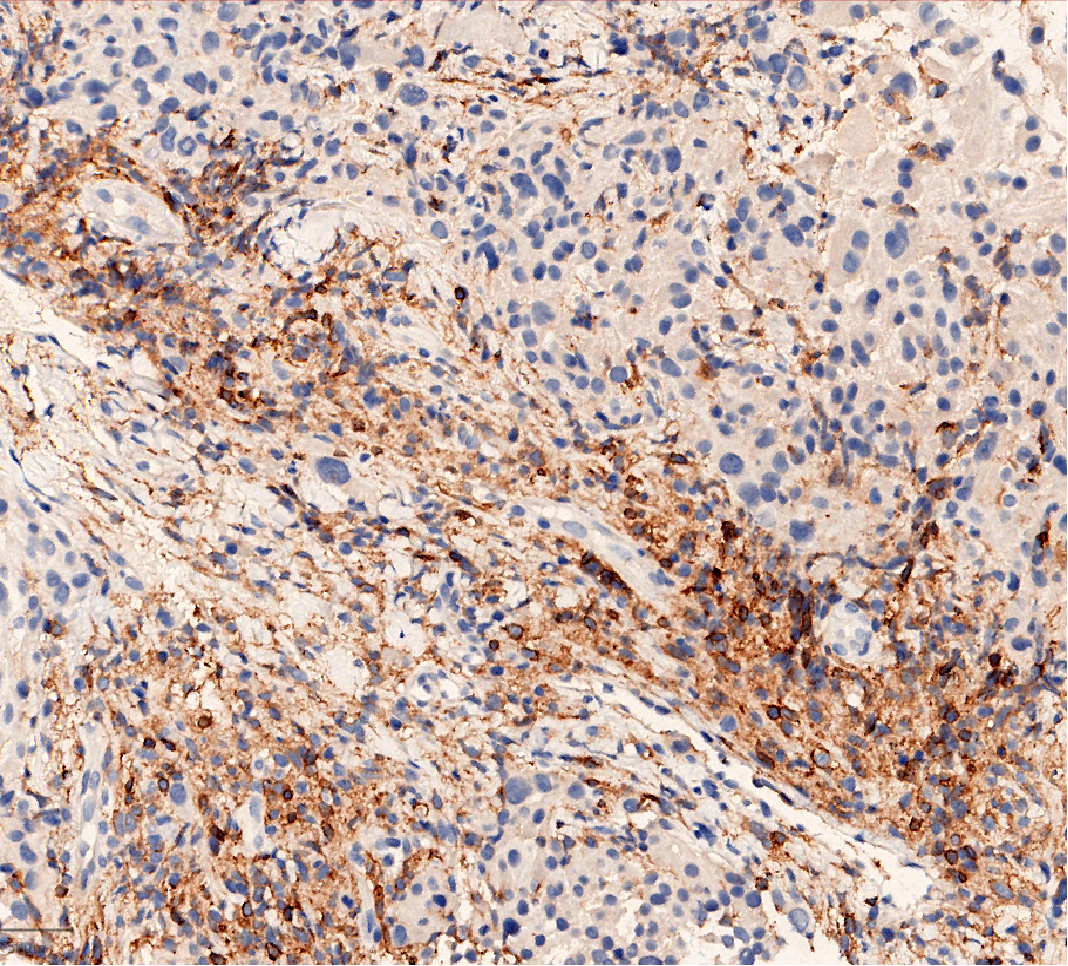

Supplement: Supplementary file 5 — Source data Fig. 1 [file 44321_2026_451_MOESM5_ESM.zip › Fig.1B/After treatment CD4.png]

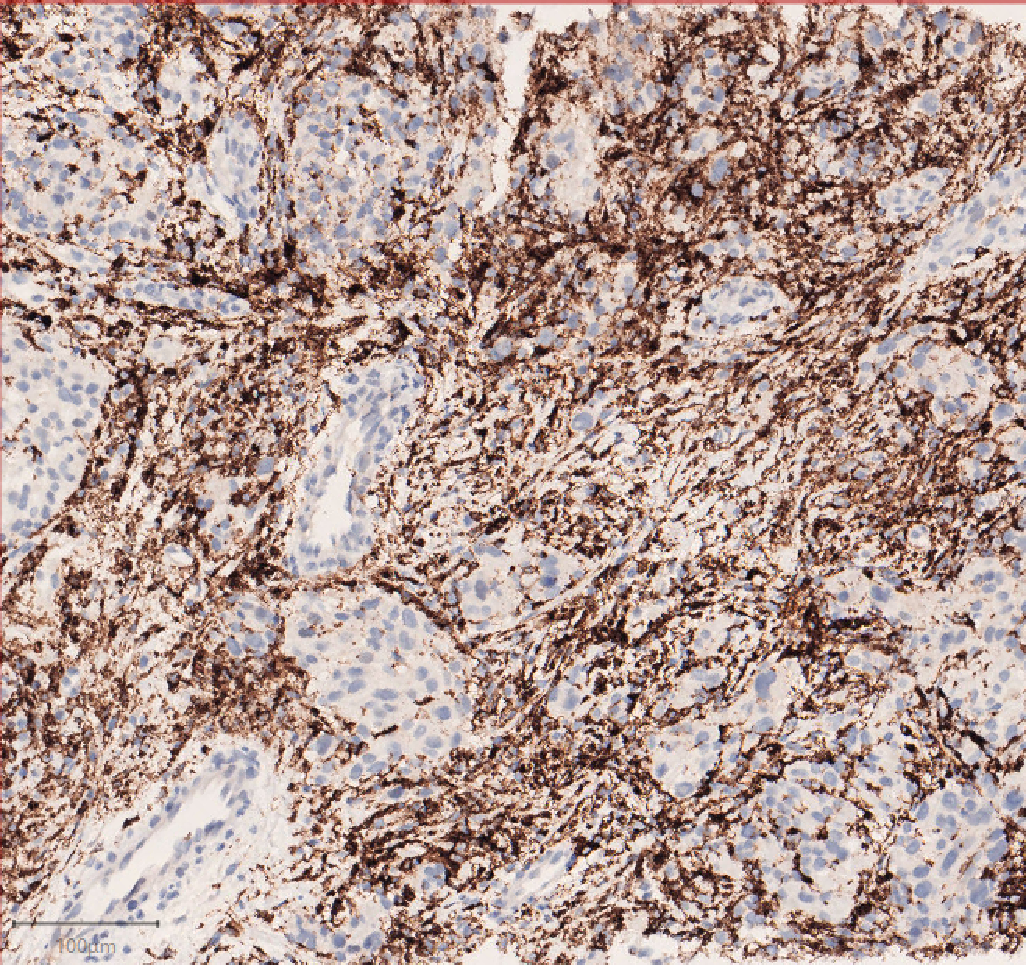

Supplement: Supplementary file 5 — Source data Fig. 1 [file 44321_2026_451_MOESM5_ESM.zip › Fig.1B/After treatment CD68.png]

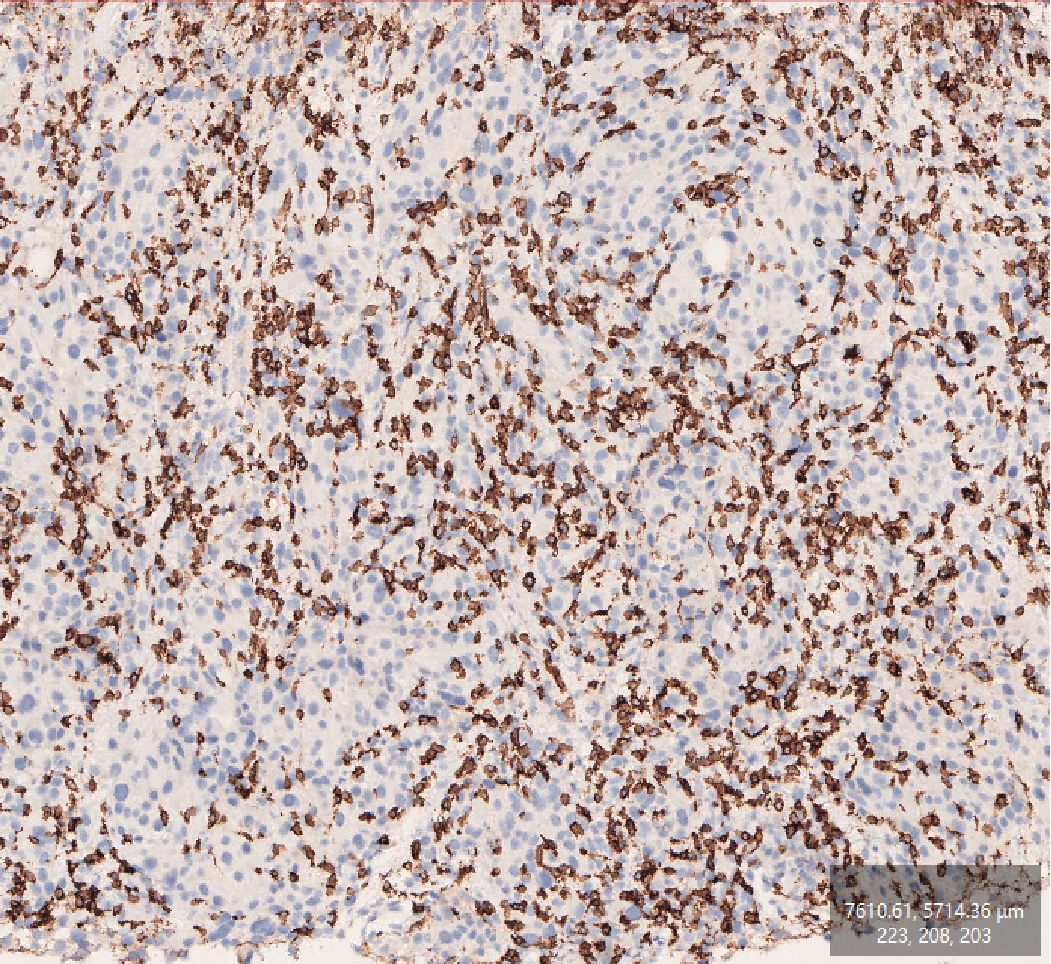

Supplement: Supplementary file 5 — Source data Fig. 1 [file 44321_2026_451_MOESM5_ESM.zip › Fig.1B/After treatment CD8.png]

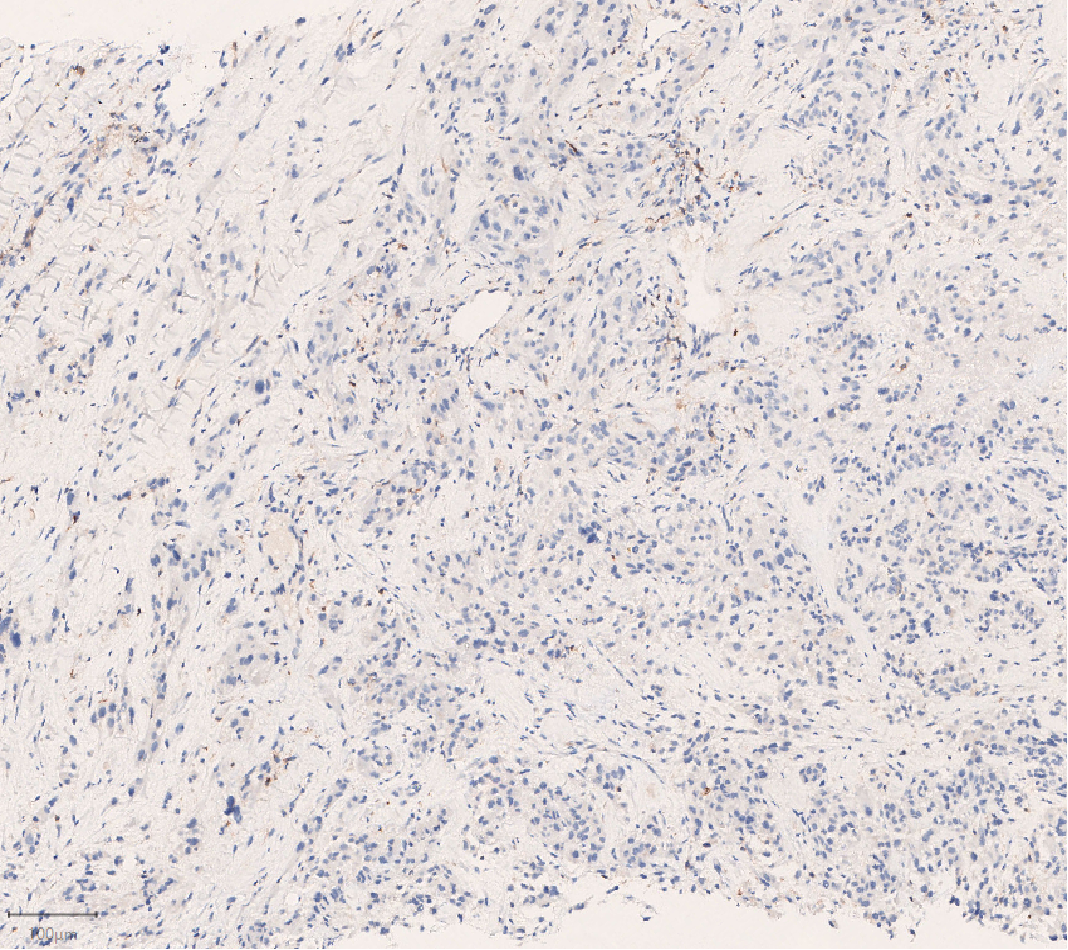

Supplement: Supplementary file 5 — Source data Fig. 1 [file 44321_2026_451_MOESM5_ESM.zip › Fig.1B/Before treatment CD4.png]

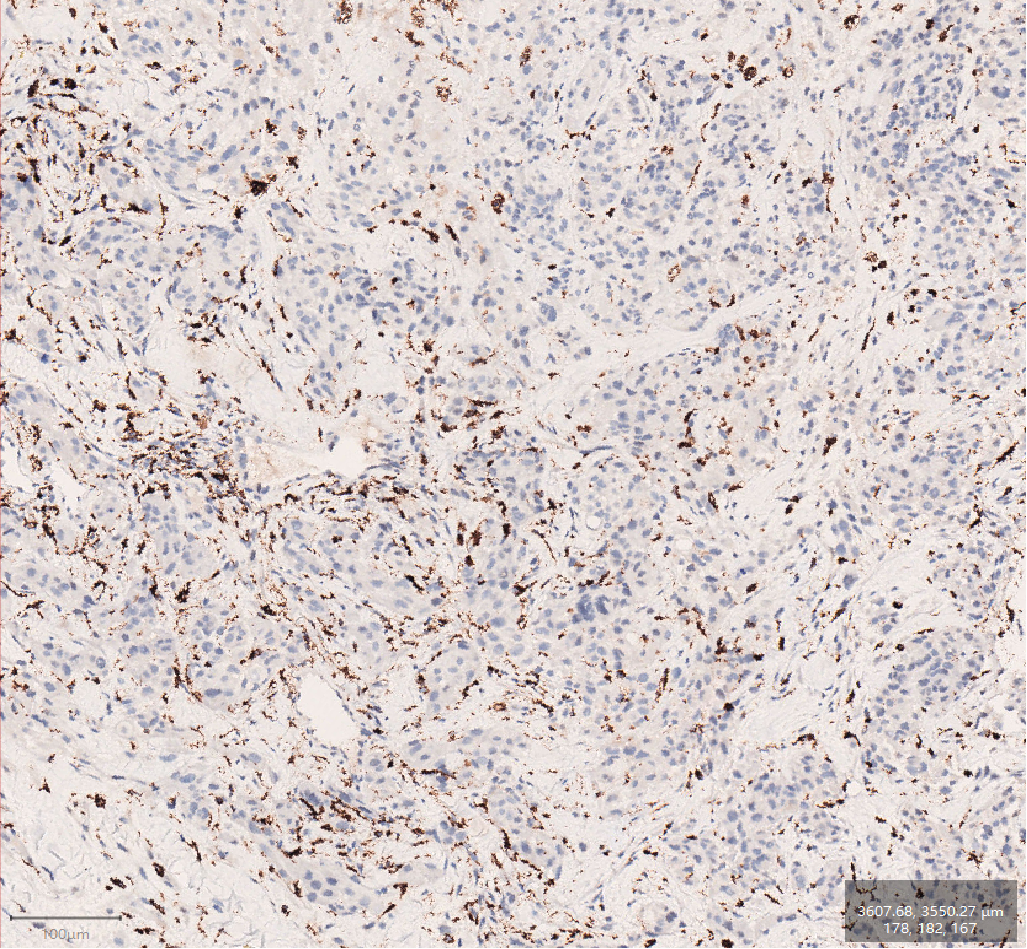

Supplement: Supplementary file 5 — Source data Fig. 1 [file 44321_2026_451_MOESM5_ESM.zip › Fig.1B/Before treatment CD68.png]

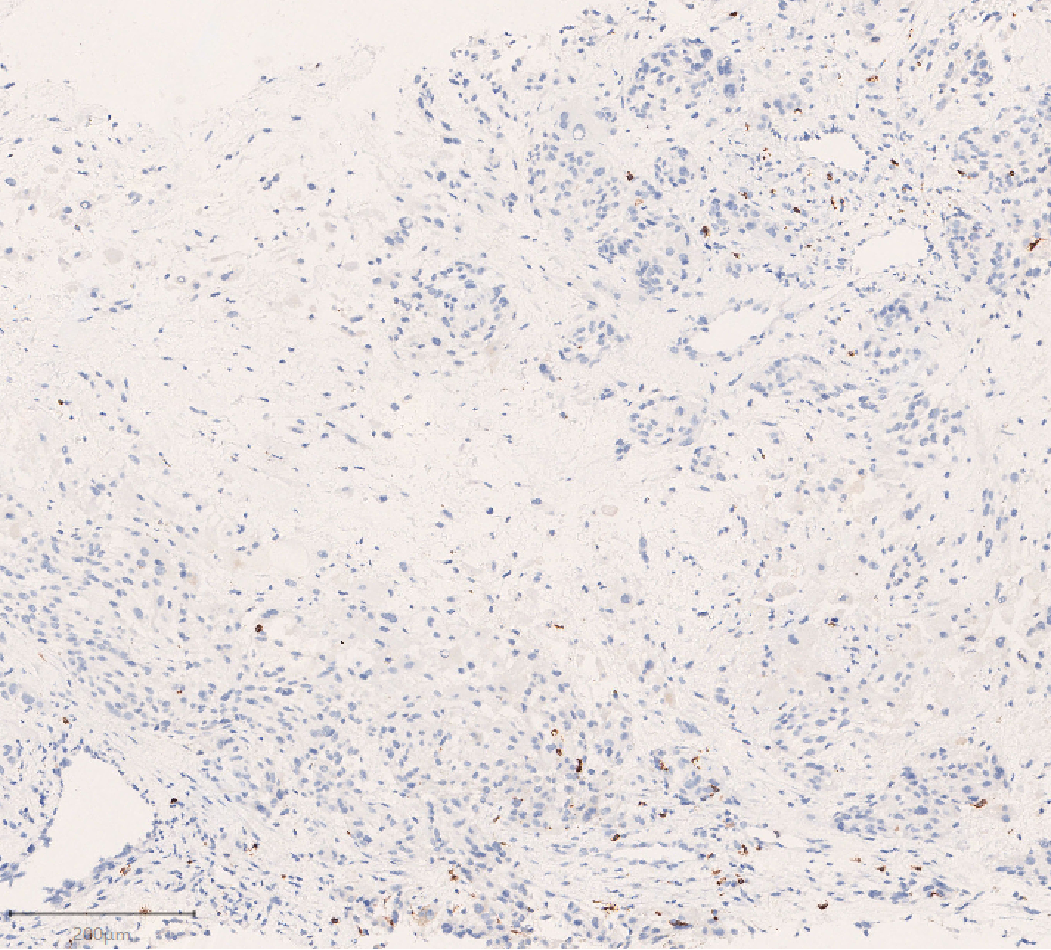

Supplement: Supplementary file 5 — Source data Fig. 1 [file 44321_2026_451_MOESM5_ESM.zip › Fig.1B/Before treatment CD8.png]

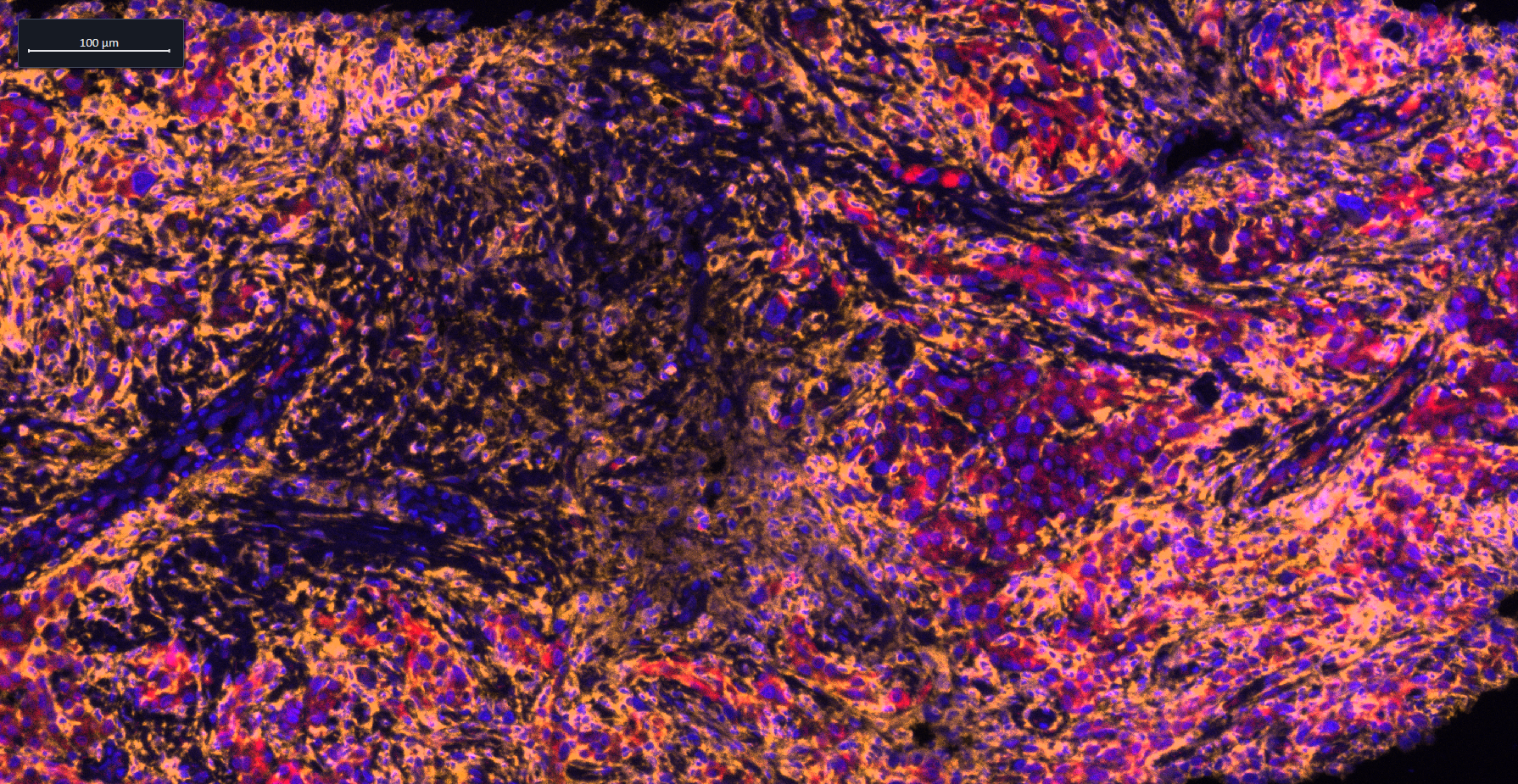

Supplement: Supplementary file 5 — Source data Fig. 1 [file 44321_2026_451_MOESM5_ESM.zip › Fig.1C/After treatment CD3L1 CD45.PNG]

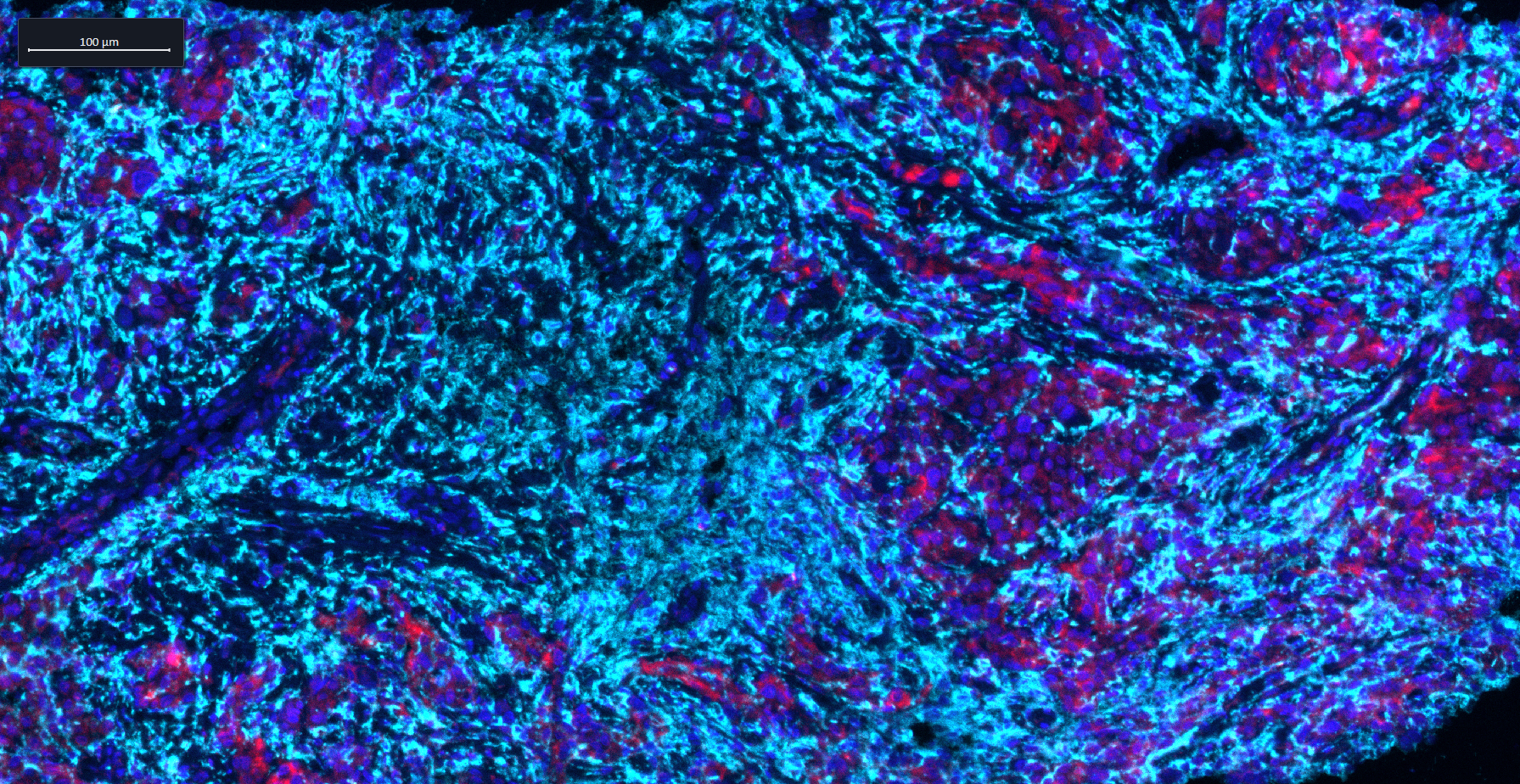

Supplement: Supplementary file 5 — Source data Fig. 1 [file 44321_2026_451_MOESM5_ESM.zip › Fig.1C/After treatment CD3L1 CD68.PNG]

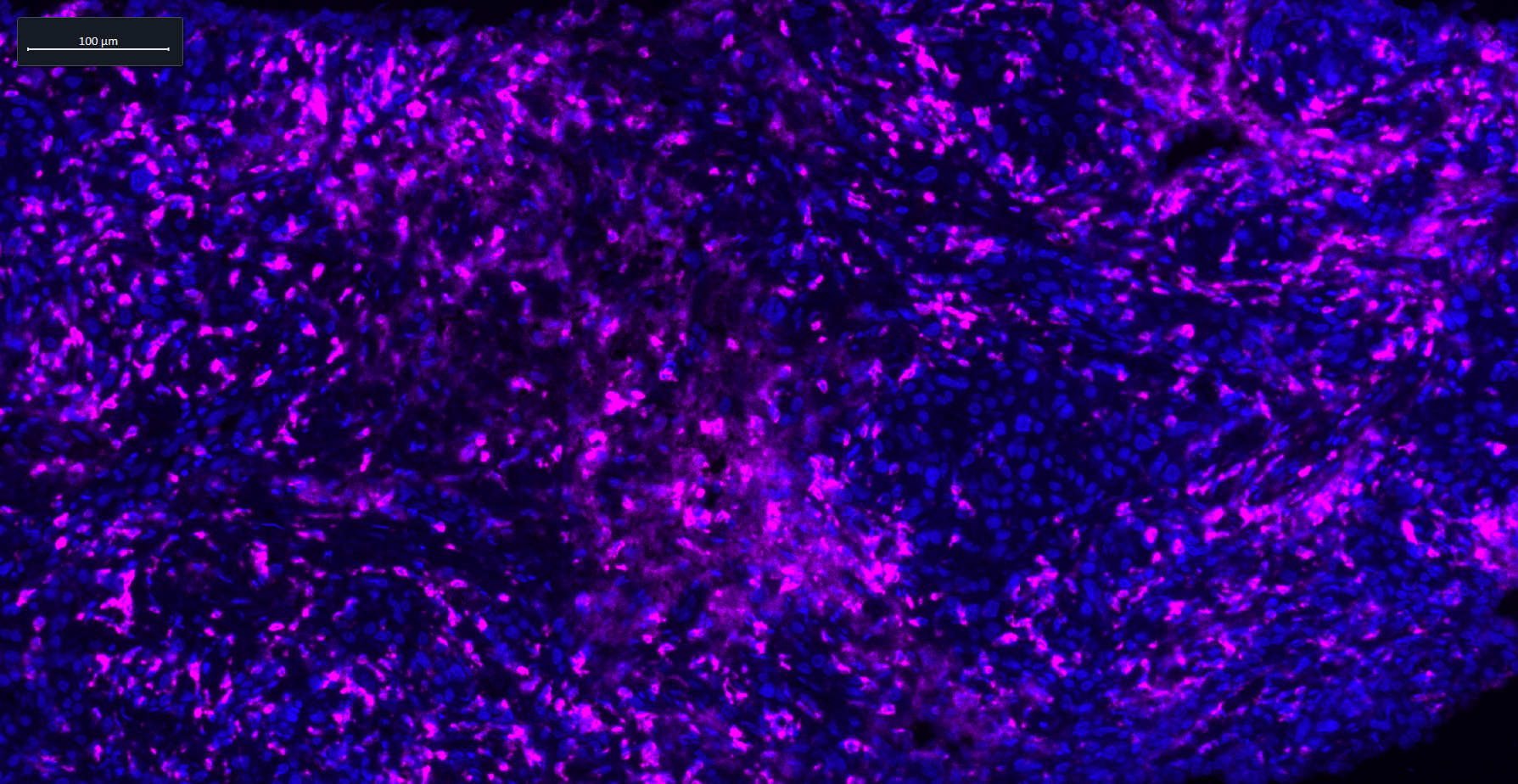

Supplement: Supplementary file 5 — Source data Fig. 1 [file 44321_2026_451_MOESM5_ESM.zip › Fig.1C/After treatment CD8.PNG]

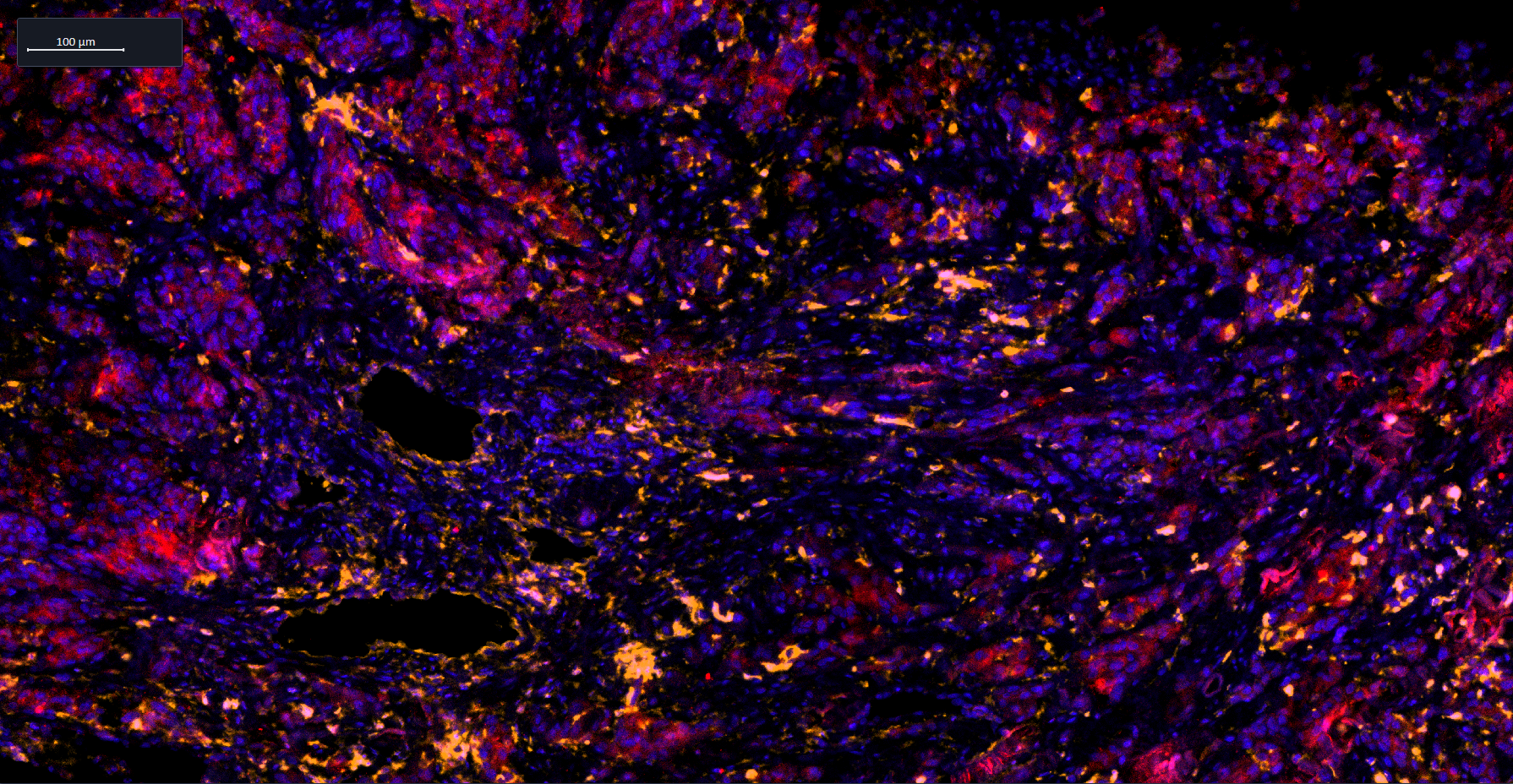

Supplement: Supplementary file 5 — Source data Fig. 1 [file 44321_2026_451_MOESM5_ESM.zip › Fig.1C/Before treatment CD3L1 CD45.PNG]

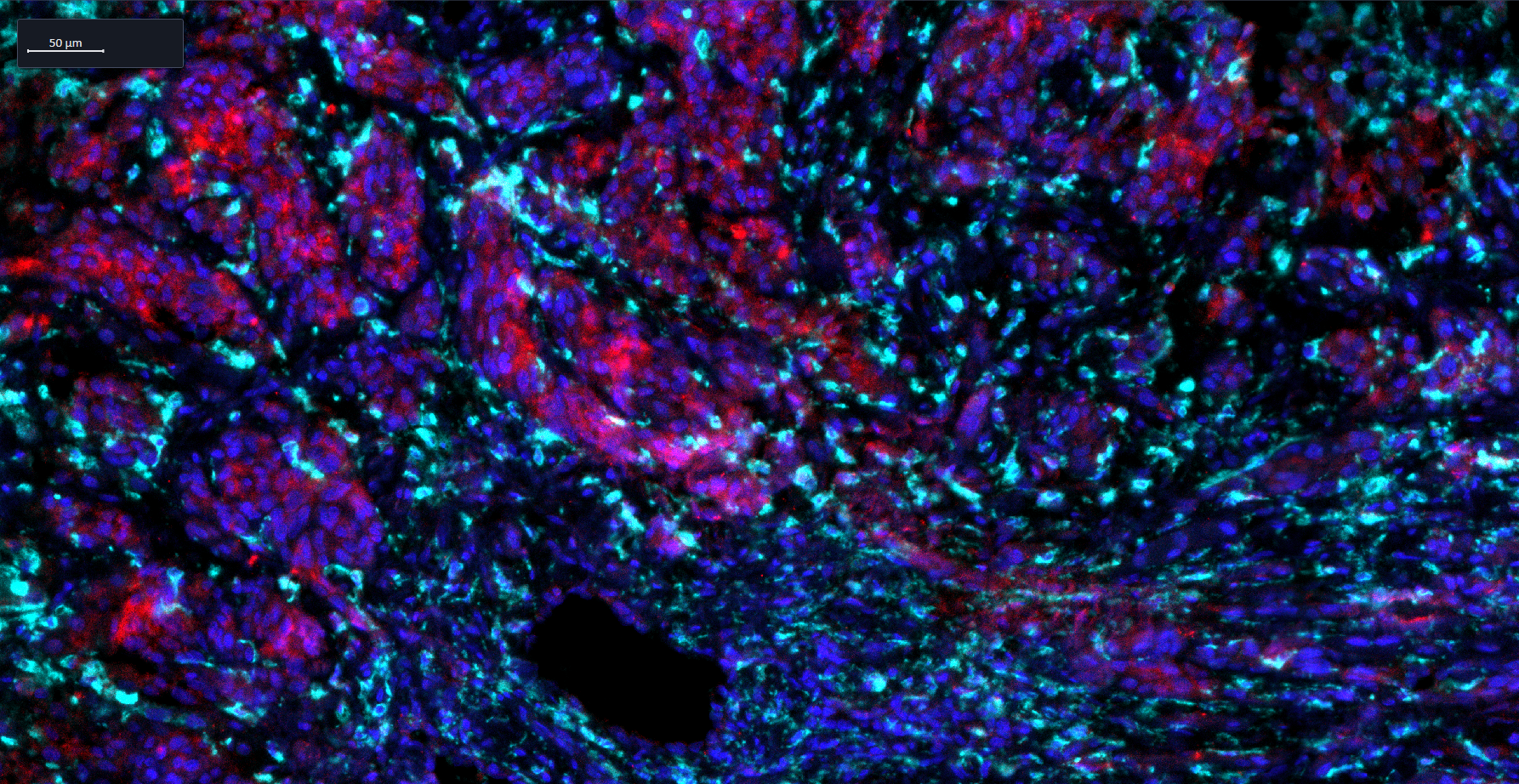

Supplement: Supplementary file 5 — Source data Fig. 1 [file 44321_2026_451_MOESM5_ESM.zip › Fig.1C/Before treatment CD3L1 CD68.PNG]

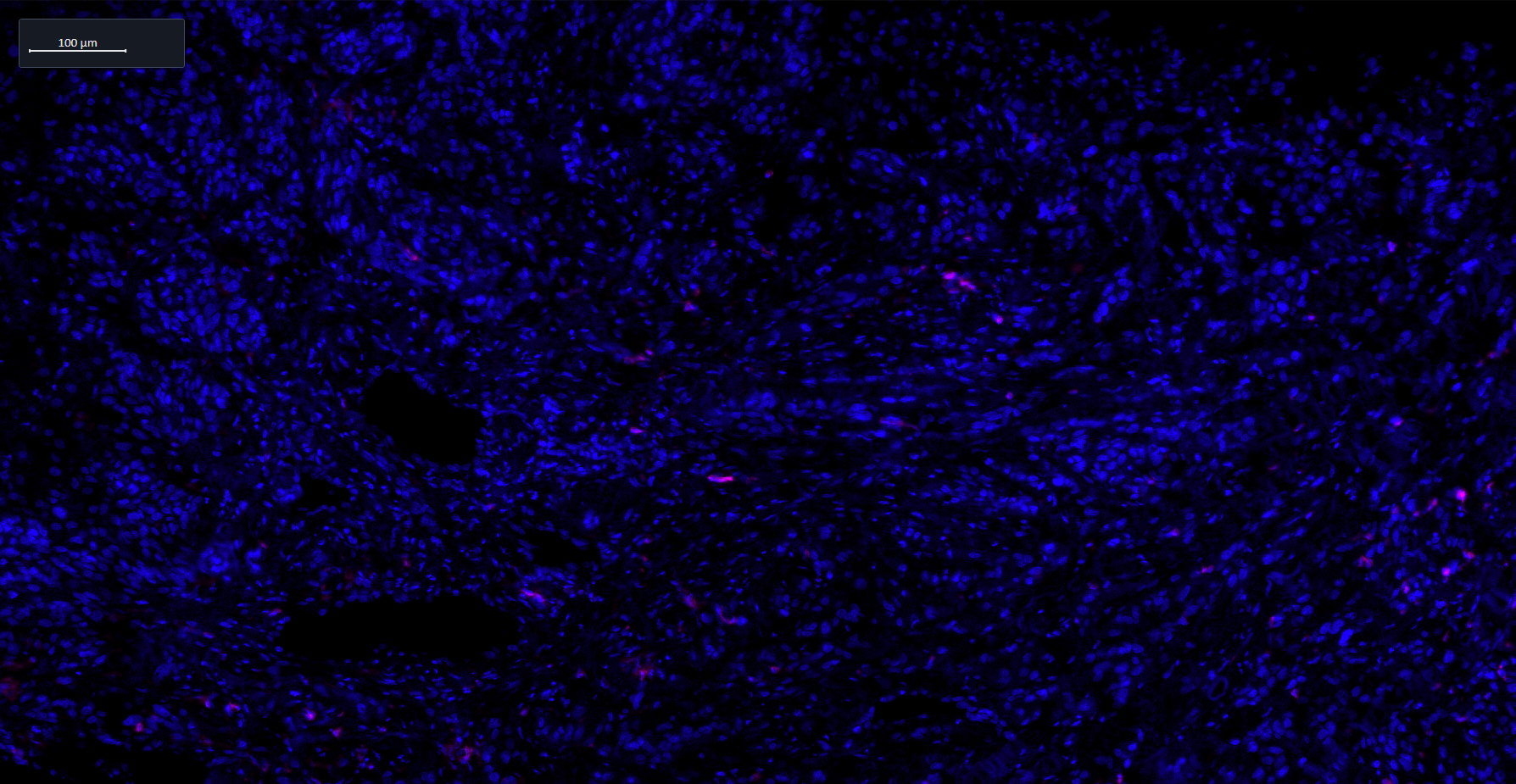

Supplement: Supplementary file 5 — Source data Fig. 1 [file 44321_2026_451_MOESM5_ESM.zip › Fig.1C/Before treatment CD8.PNG]

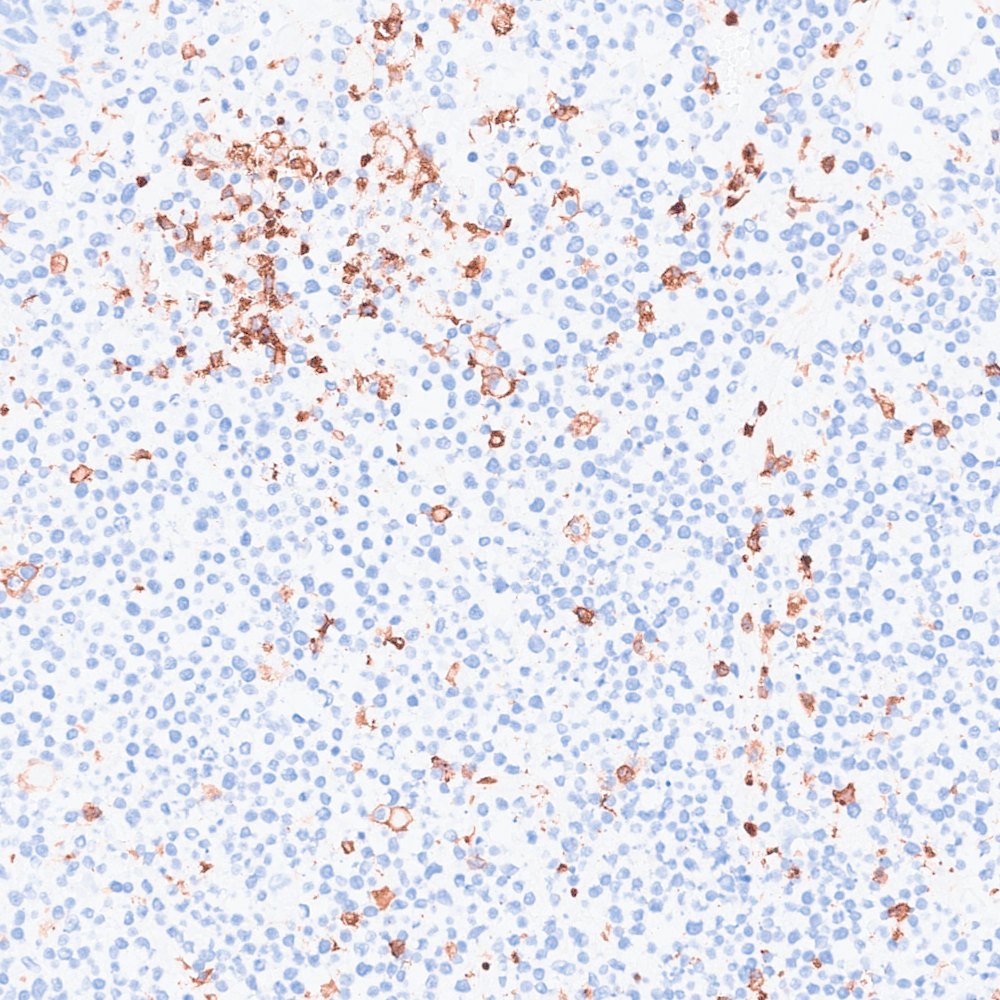

Supplement: Supplementary file 5 — Source data Fig. 1 [file 44321_2026_451_MOESM5_ESM.zip › Fig.1D/CD68 143B anti-CD3L1 zoom.png]

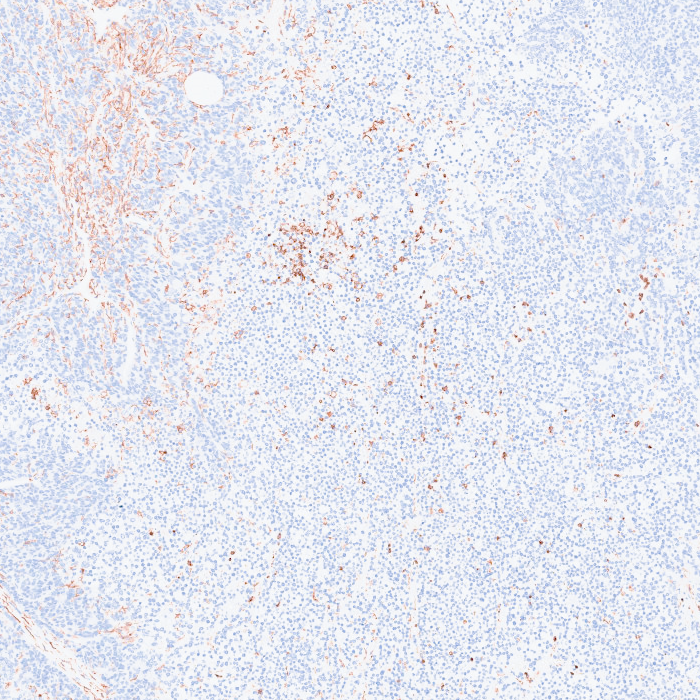

Supplement: Supplementary file 5 — Source data Fig. 1 [file 44321_2026_451_MOESM5_ESM.zip › Fig.1D/CD68 143B anti-CD3L1.png]

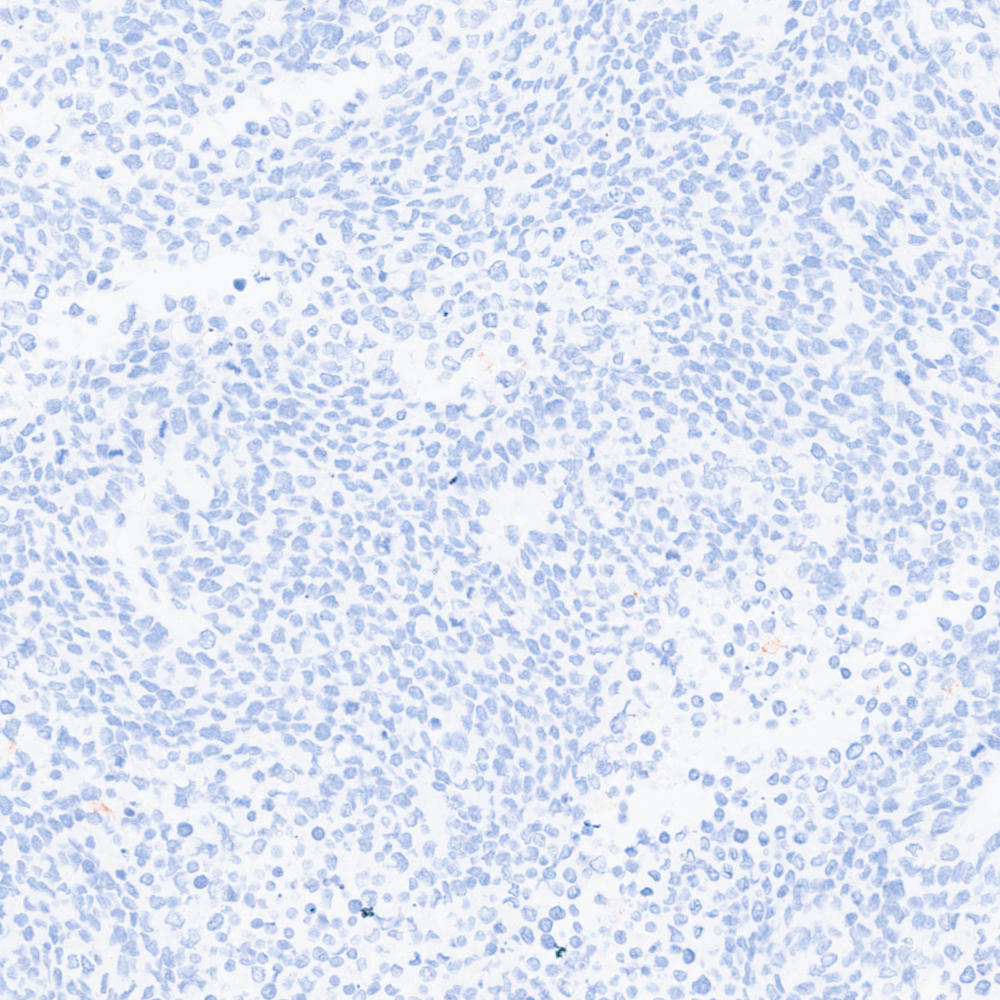

Supplement: Supplementary file 5 — Source data Fig. 1 [file 44321_2026_451_MOESM5_ESM.zip › Fig.1D/CD68 143B IgG zoom.png]

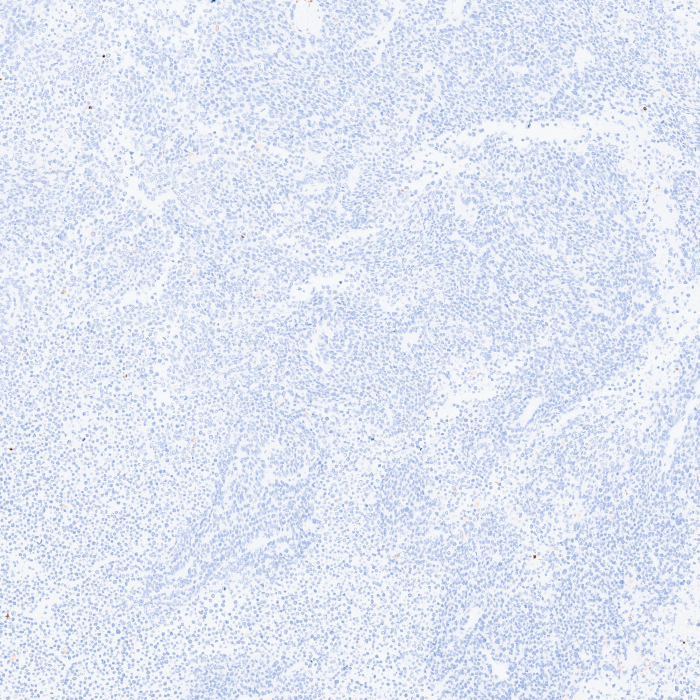

Supplement: Supplementary file 5 — Source data Fig. 1 [file 44321_2026_451_MOESM5_ESM.zip › Fig.1D/CD68 143B IgG.png]

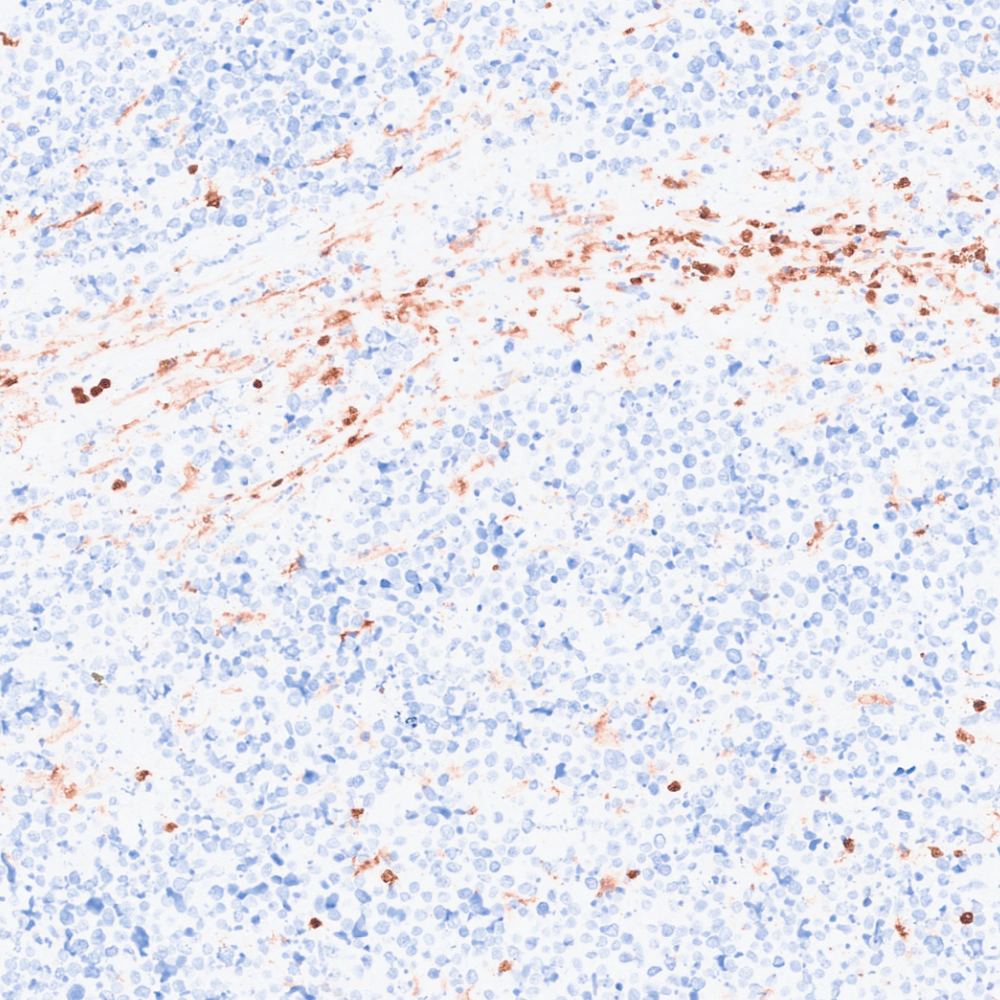

Supplement: Supplementary file 5 — Source data Fig. 1 [file 44321_2026_451_MOESM5_ESM.zip › Fig.1D/CD68 KHOS anti-CD3L1 zoom.png]

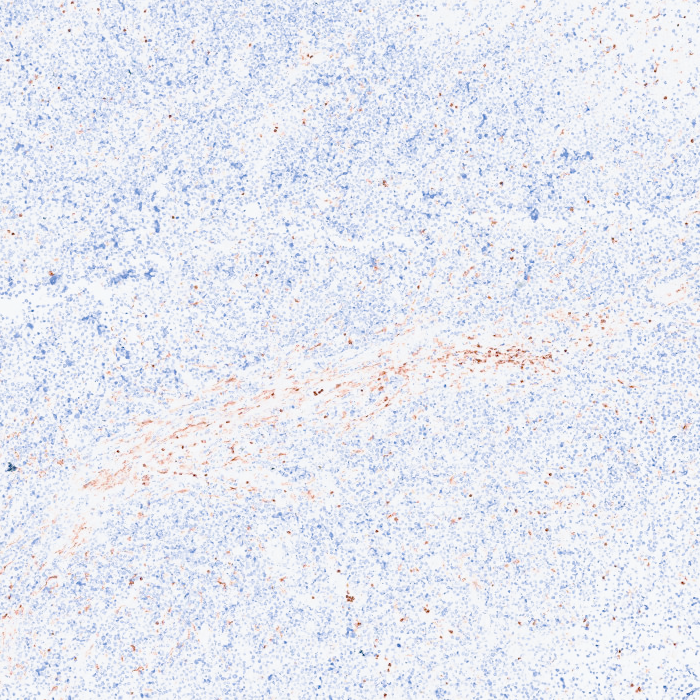

Supplement: Supplementary file 5 — Source data Fig. 1 [file 44321_2026_451_MOESM5_ESM.zip › Fig.1D/CD68 KHOS anti-CD3L1.png]

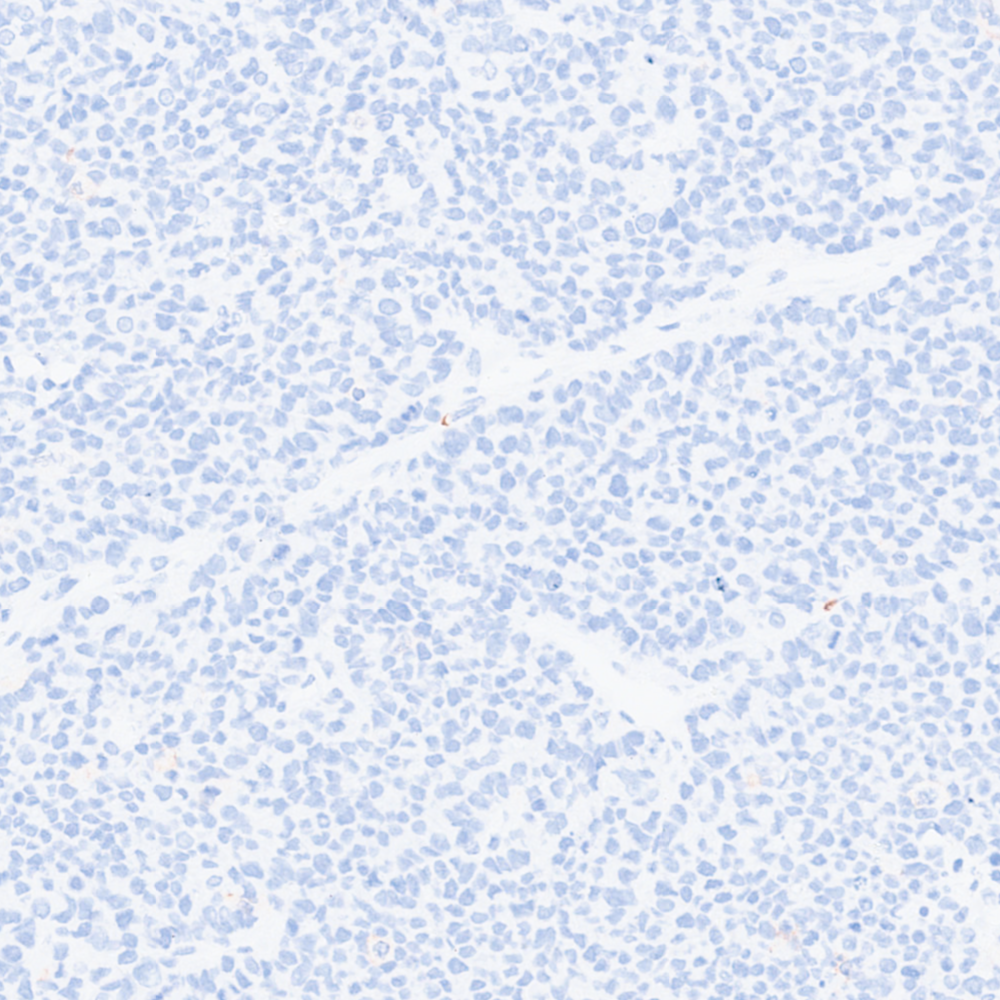

Supplement: Supplementary file 5 — Source data Fig. 1 [file 44321_2026_451_MOESM5_ESM.zip › Fig.1D/CD68 KHOS IgG zoom.png]

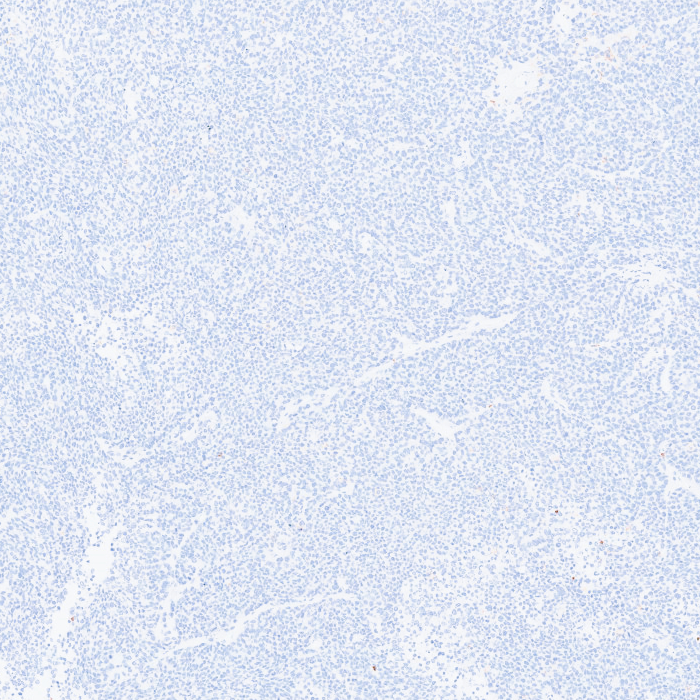

Supplement: Supplementary file 5 — Source data Fig. 1 [file 44321_2026_451_MOESM5_ESM.zip › Fig.1D/CD68 KHOS IgG.png]

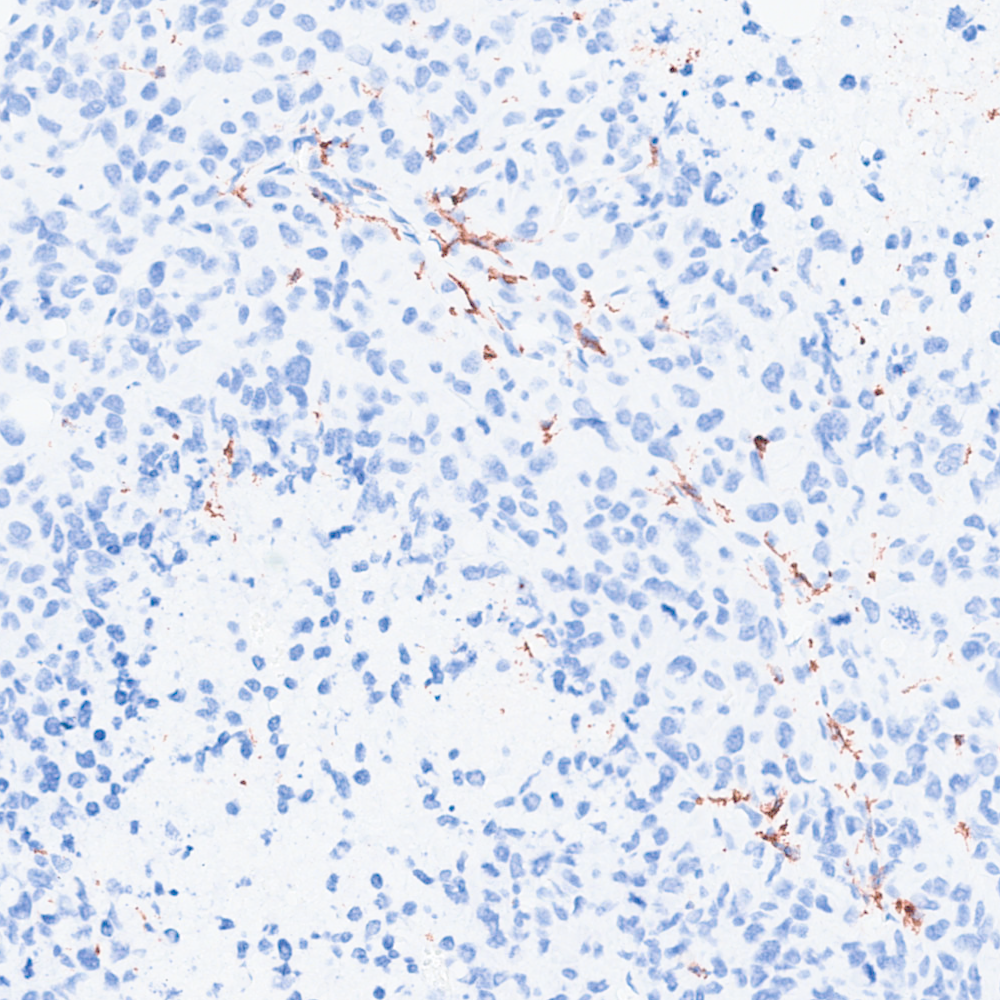

Supplement: Supplementary file 5 — Source data Fig. 1 [file 44321_2026_451_MOESM5_ESM.zip › Fig.1E/CD206 143B anti-CD3L1 zoom.png]

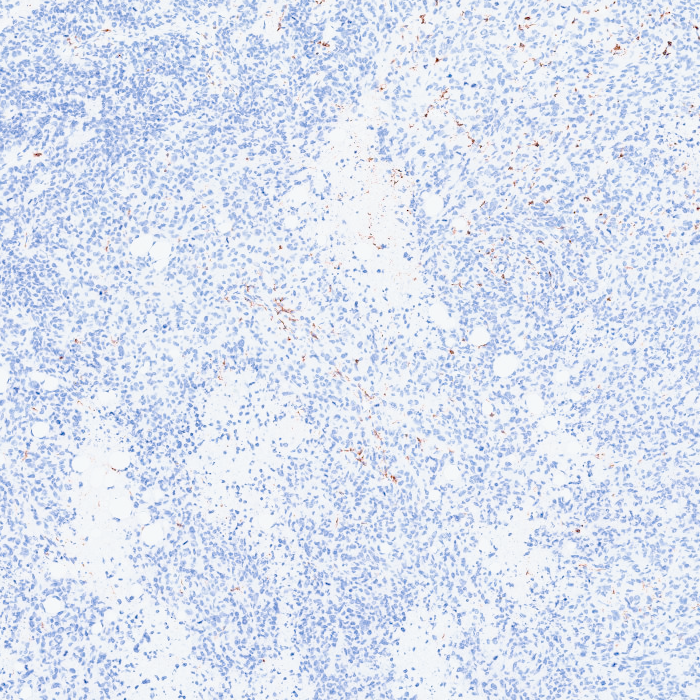

Supplement: Supplementary file 5 — Source data Fig. 1 [file 44321_2026_451_MOESM5_ESM.zip › Fig.1E/CD206 143B anti-CD3L1.png]

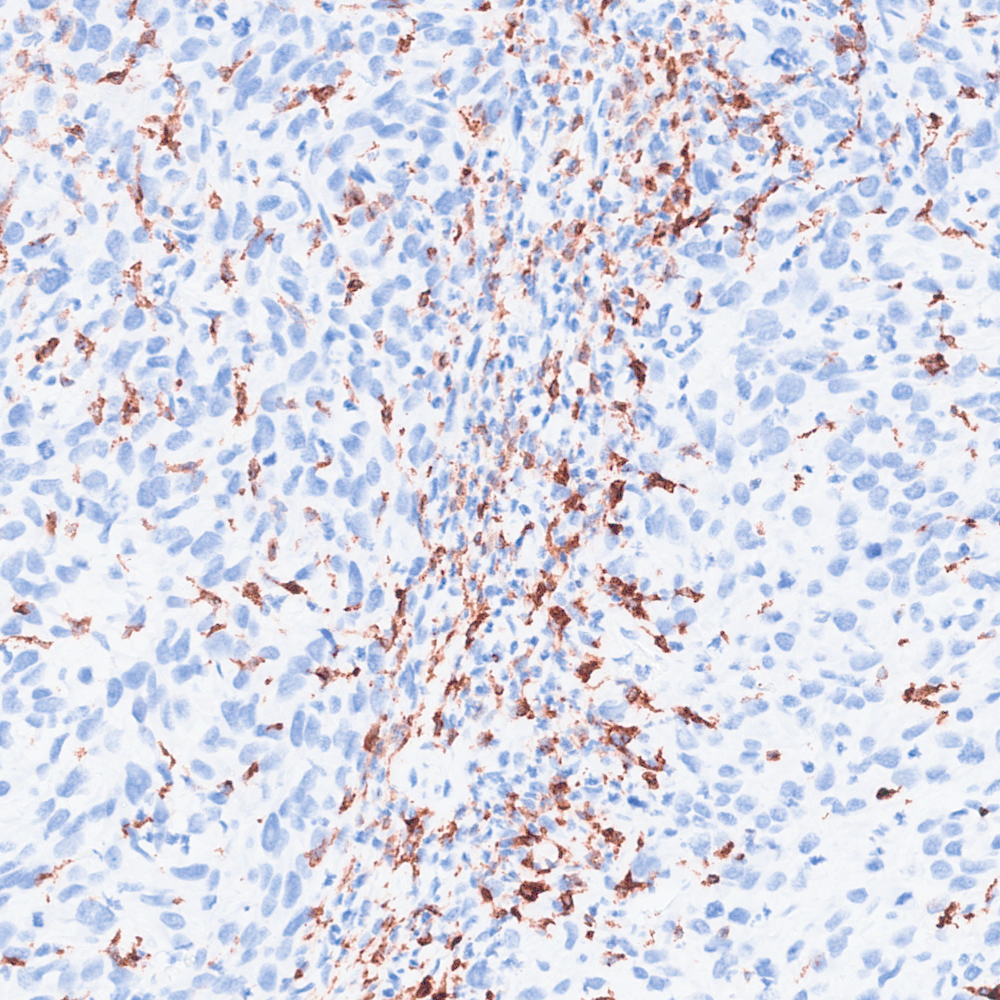

Supplement: Supplementary file 5 — Source data Fig. 1 [file 44321_2026_451_MOESM5_ESM.zip › Fig.1E/CD206 143B IgG zoom.png]

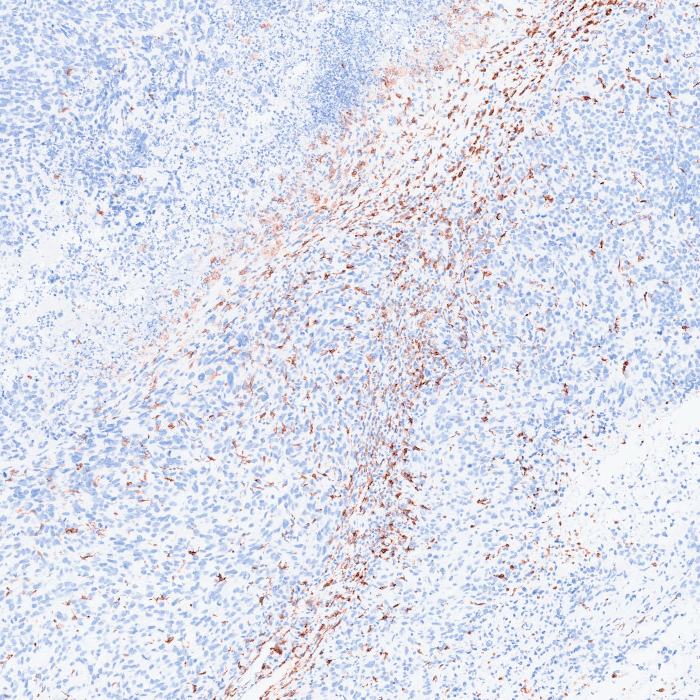

Supplement: Supplementary file 5 — Source data Fig. 1 [file 44321_2026_451_MOESM5_ESM.zip › Fig.1E/CD206 143B IgG.png]

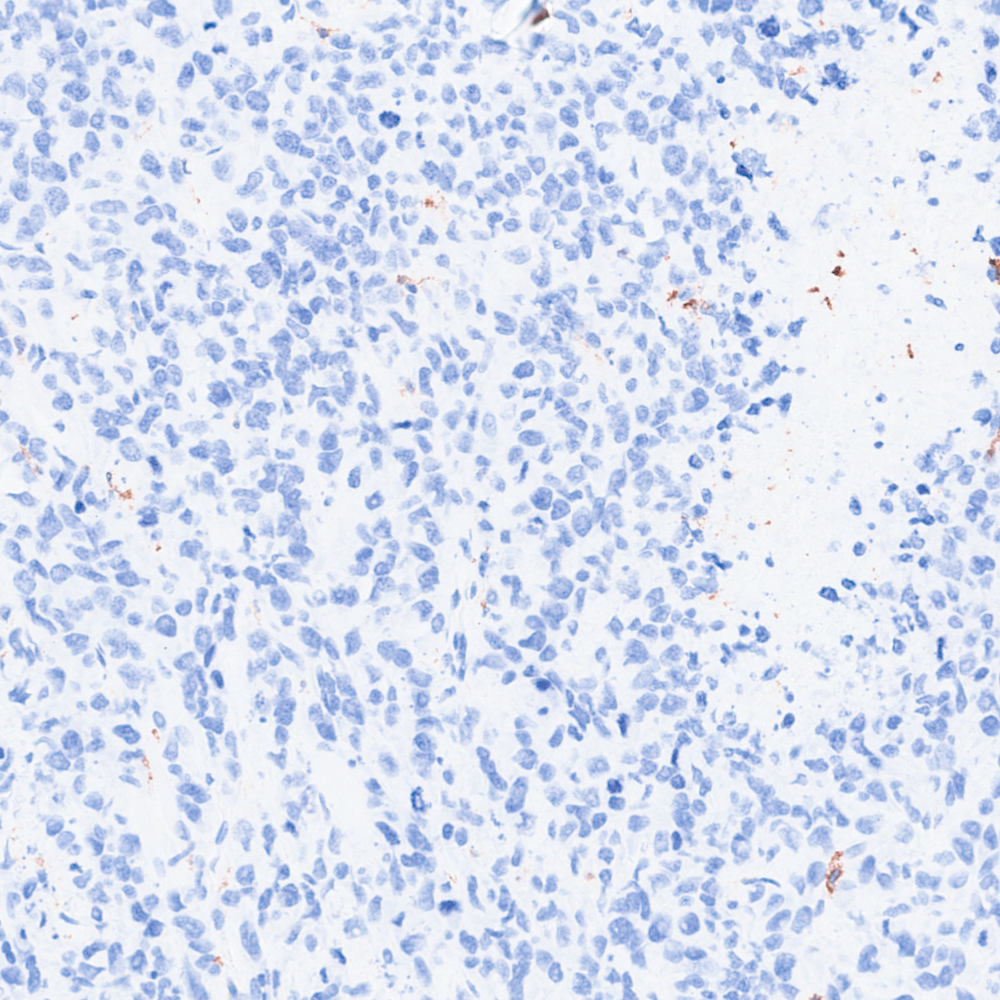

Supplement: Supplementary file 5 — Source data Fig. 1 [file 44321_2026_451_MOESM5_ESM.zip › Fig.1E/CD206 KHOS Anti-CD3L1 zoom.png]

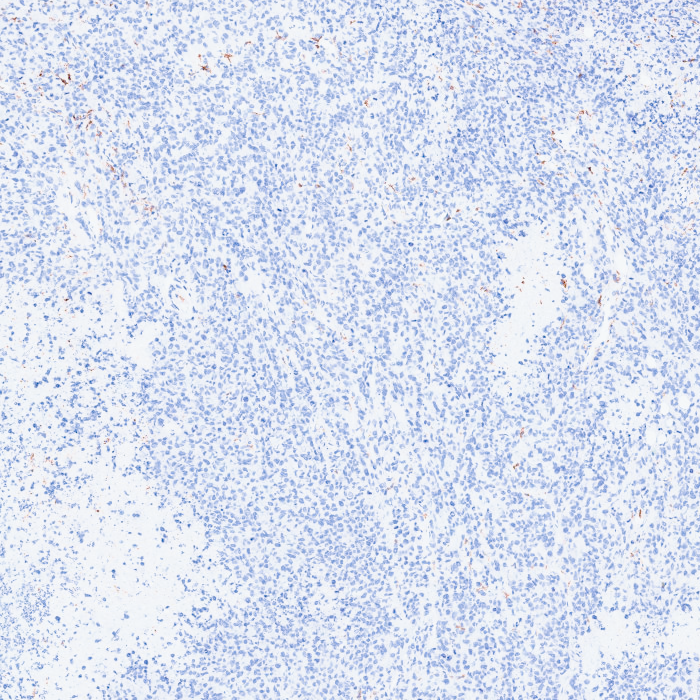

Supplement: Supplementary file 5 — Source data Fig. 1 [file 44321_2026_451_MOESM5_ESM.zip › Fig.1E/CD206 KHOS anti-CD3L1.png]

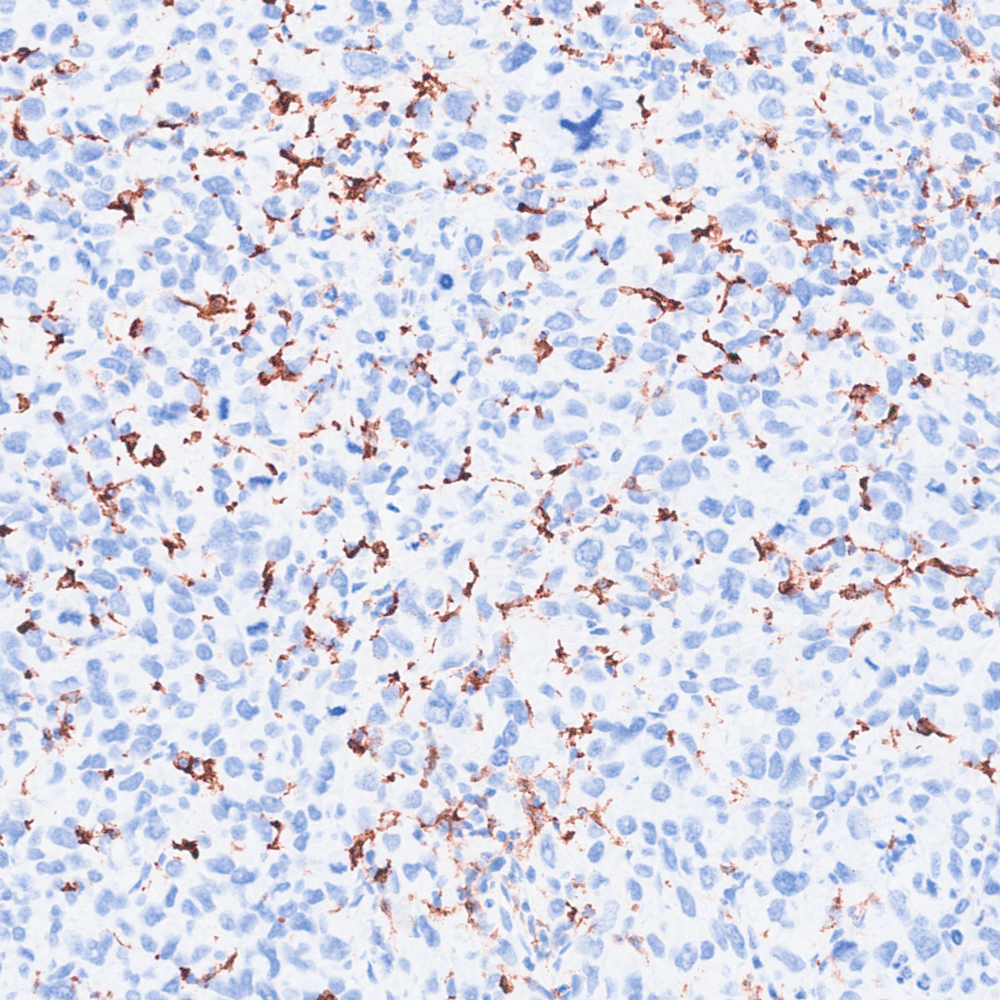

Supplement: Supplementary file 5 — Source data Fig. 1 [file 44321_2026_451_MOESM5_ESM.zip › Fig.1E/CD206 KHOS IgG zoom.png]

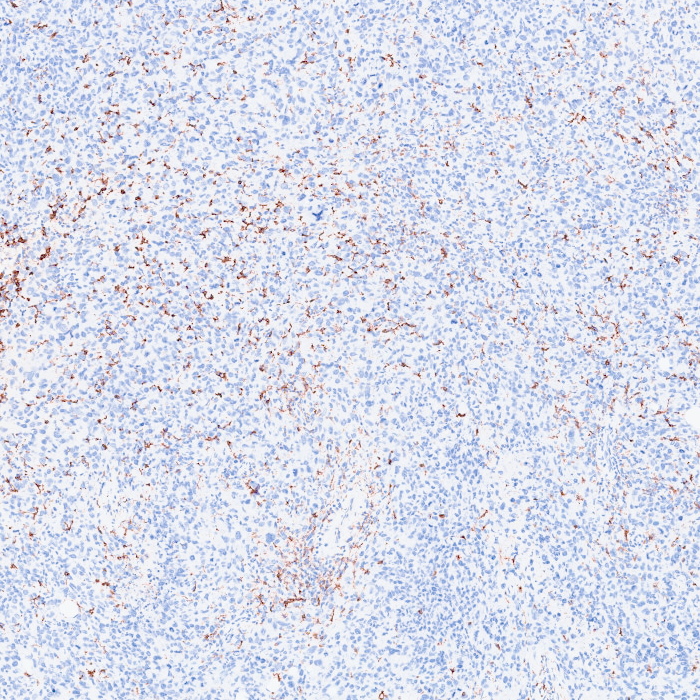

Supplement: Supplementary file 5 — Source data Fig. 1 [file 44321_2026_451_MOESM5_ESM.zip › Fig.1E/CD206 KHOS IgG.png]

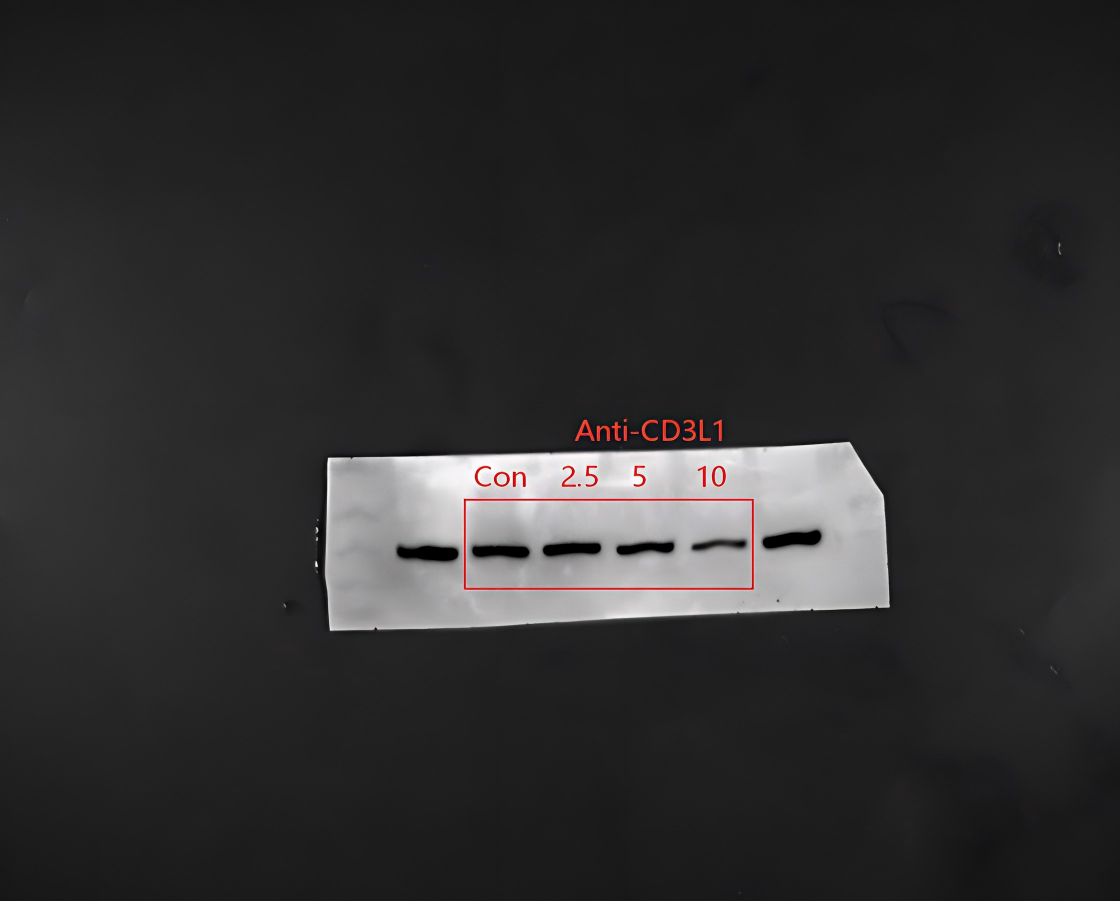

Supplement: Supplementary file 6 — Source data Fig. 3 [file 44321_2026_451_MOESM6_ESM.zip › Fig.3B/PBMC GAPDH replicate 2.jpg]

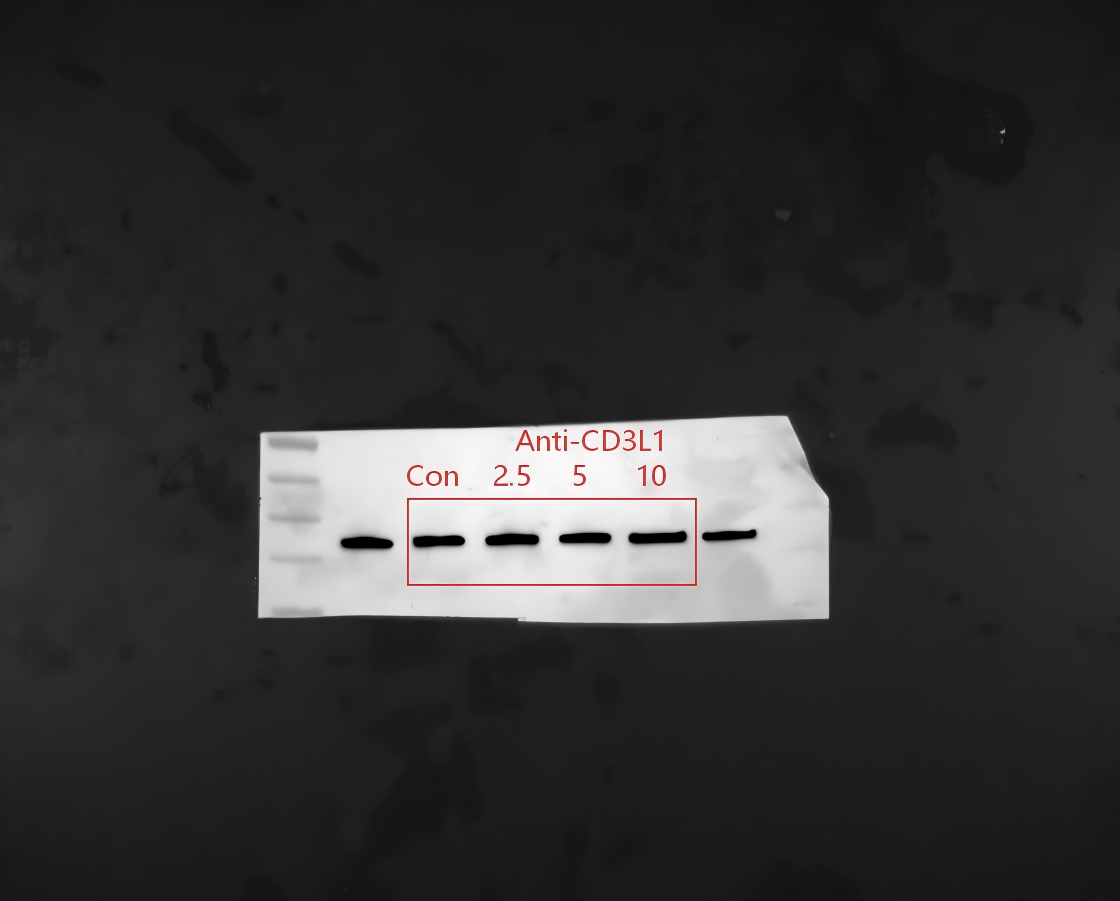

Supplement: Supplementary file 6 — Source data Fig. 3 [file 44321_2026_451_MOESM6_ESM.zip › Fig.3B/PBMC GAPDH replicate 3.jpg]

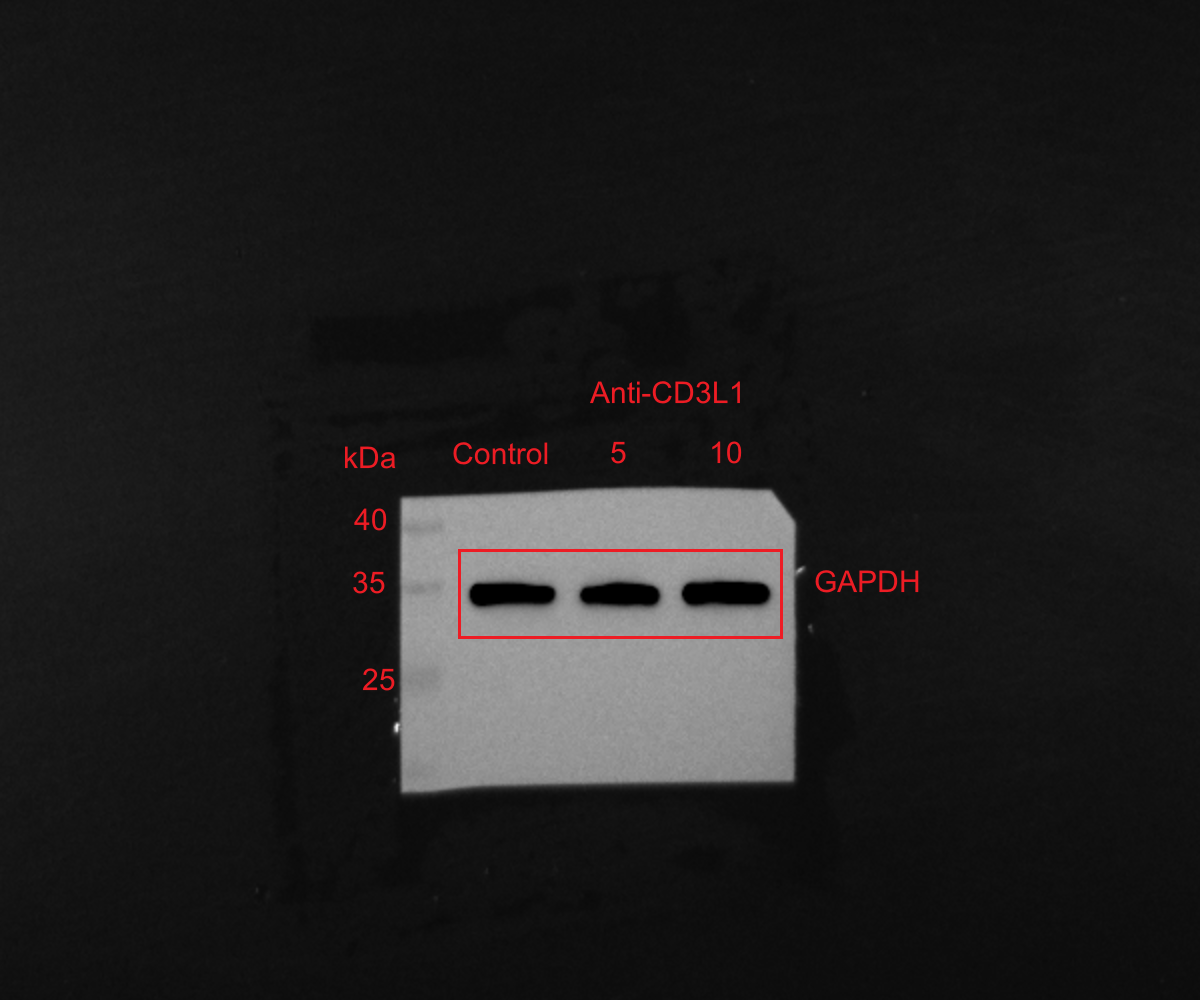

Supplement: Supplementary file 6 — Source data Fig. 3 [file 44321_2026_451_MOESM6_ESM.zip › Fig.3B/PBMC GAPDH.png]

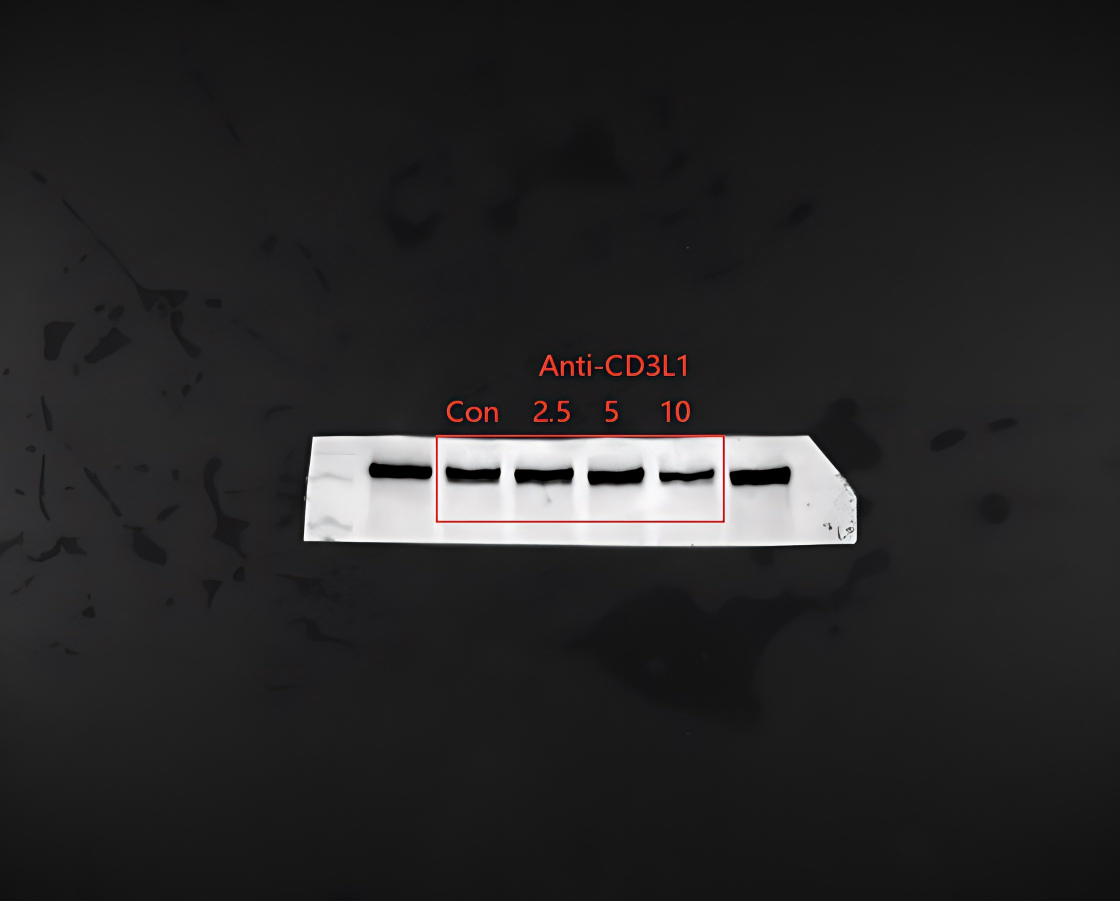

Supplement: Supplementary file 6 — Source data Fig. 3 [file 44321_2026_451_MOESM6_ESM.zip › Fig.3B/PBMC STAT6 replicate 2.jpg]

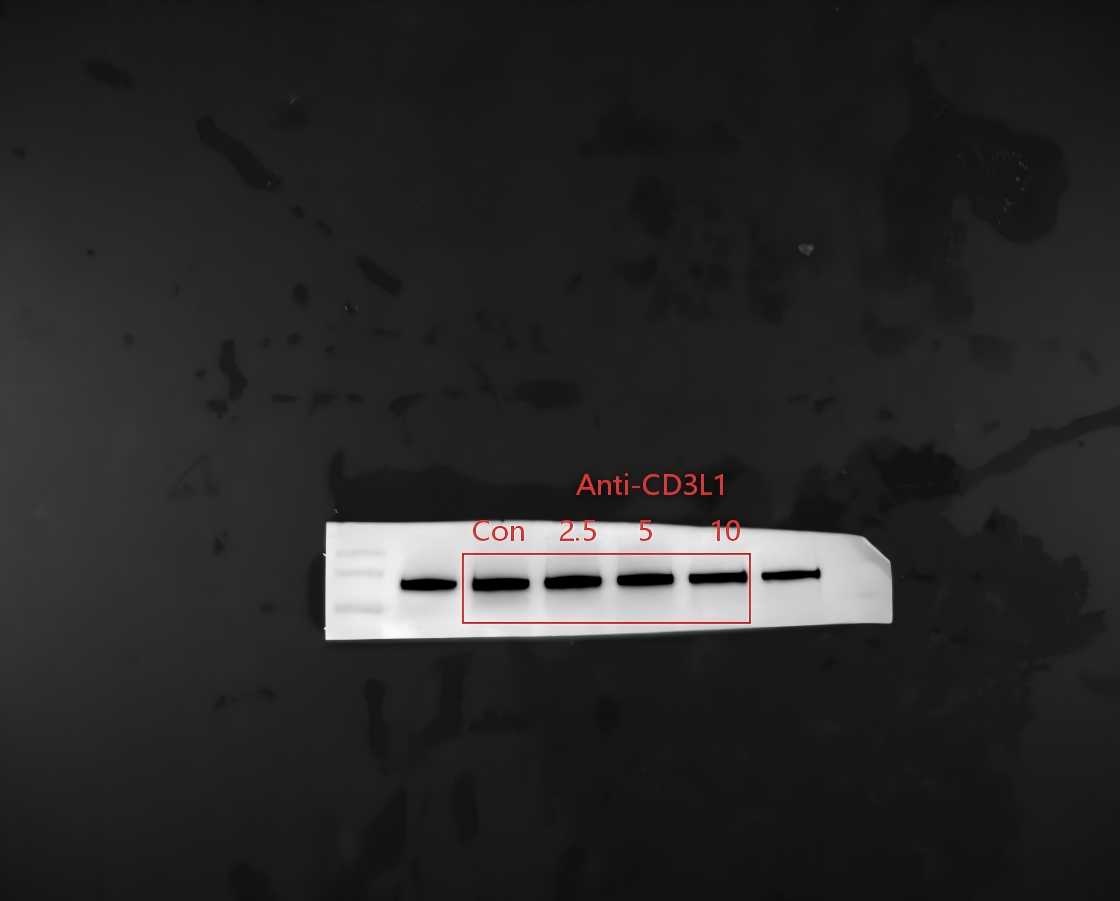

Supplement: Supplementary file 6 — Source data Fig. 3 [file 44321_2026_451_MOESM6_ESM.zip › Fig.3B/PBMC STAT6 replicate 3.jpg]

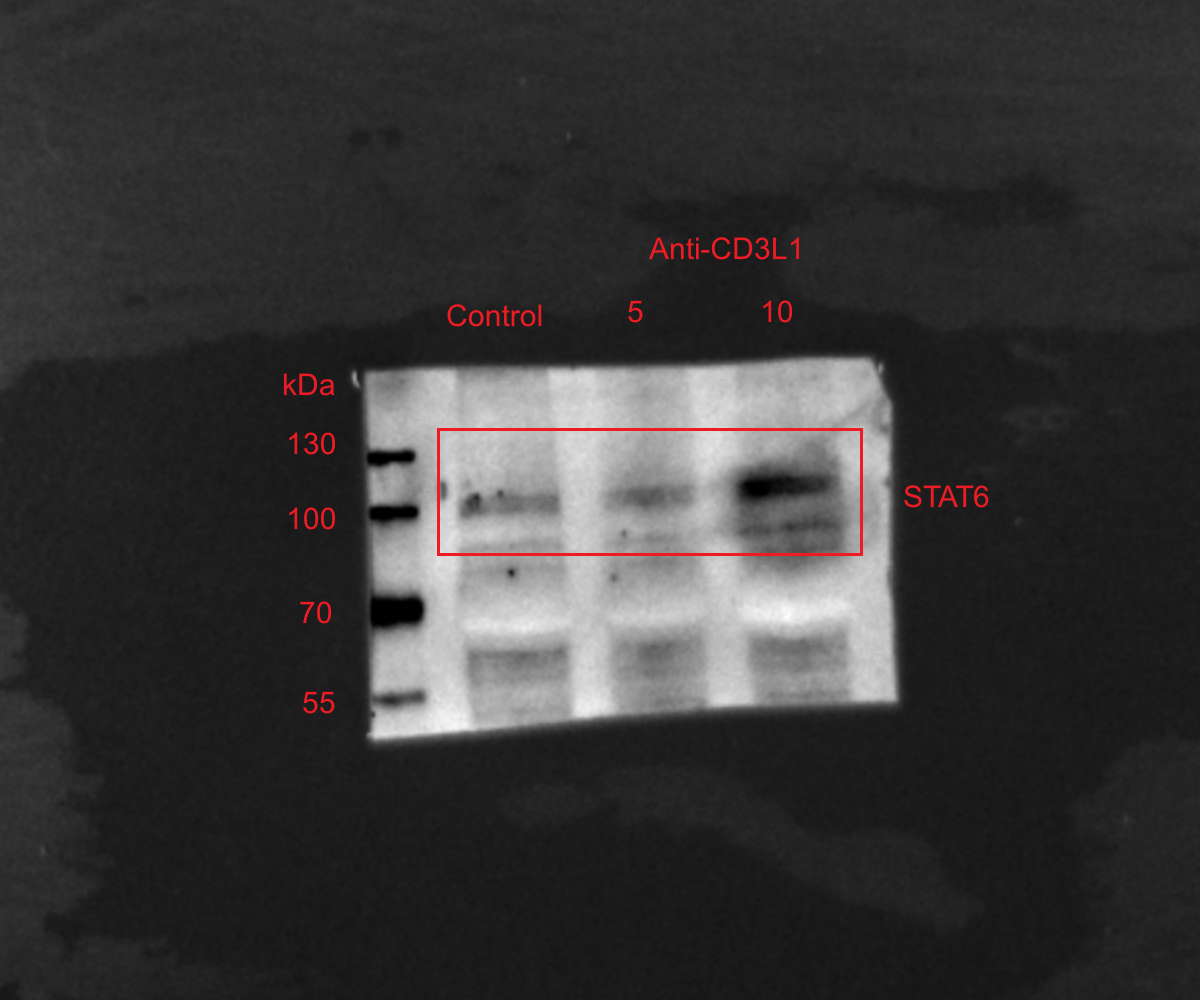

Supplement: Supplementary file 6 — Source data Fig. 3 [file 44321_2026_451_MOESM6_ESM.zip › Fig.3B/PBMC STAT6.png]

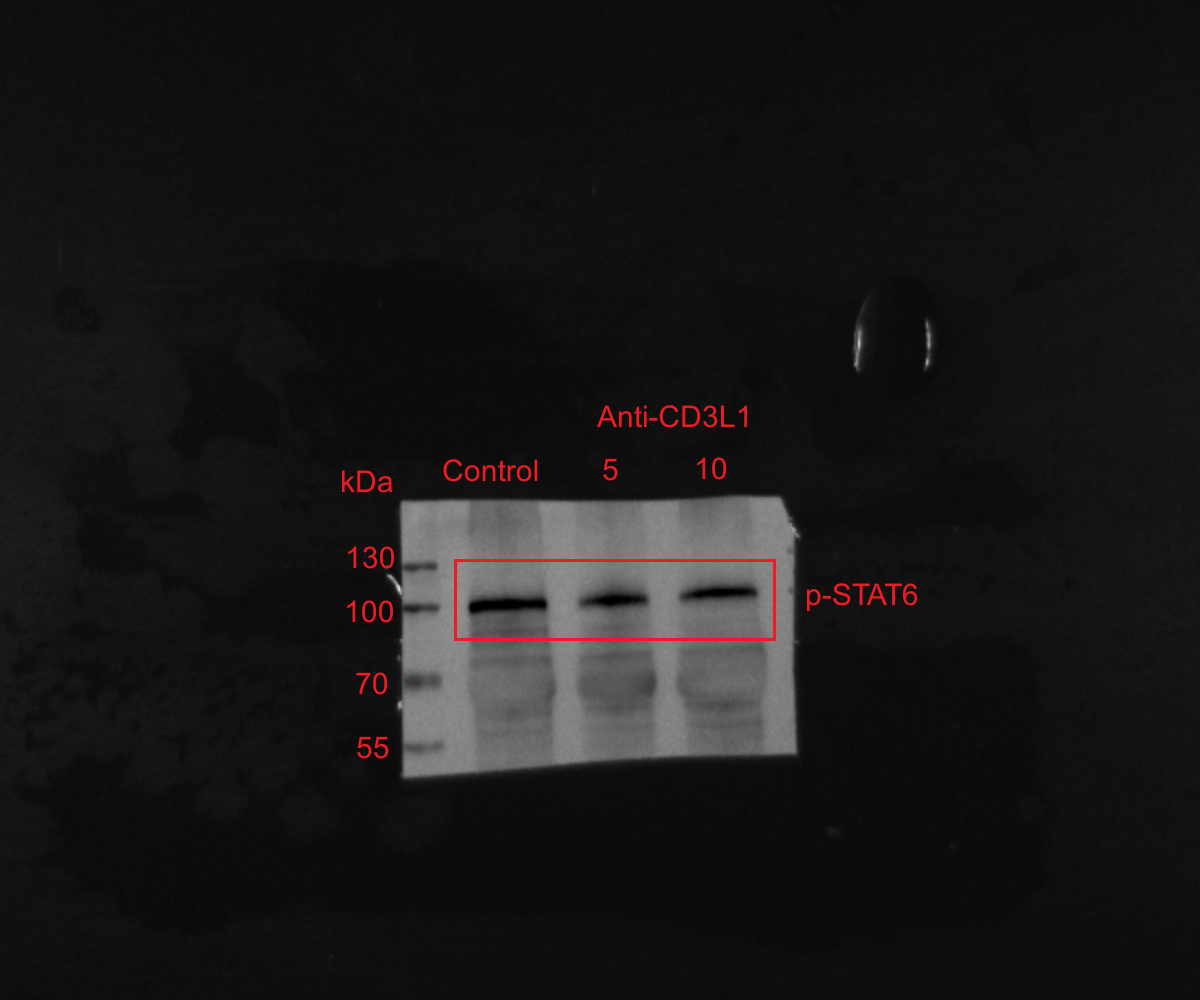

Supplement: Supplementary file 6 — Source data Fig. 3 [file 44321_2026_451_MOESM6_ESM.zip › Fig.3B/PBMC p-STAT6.png]

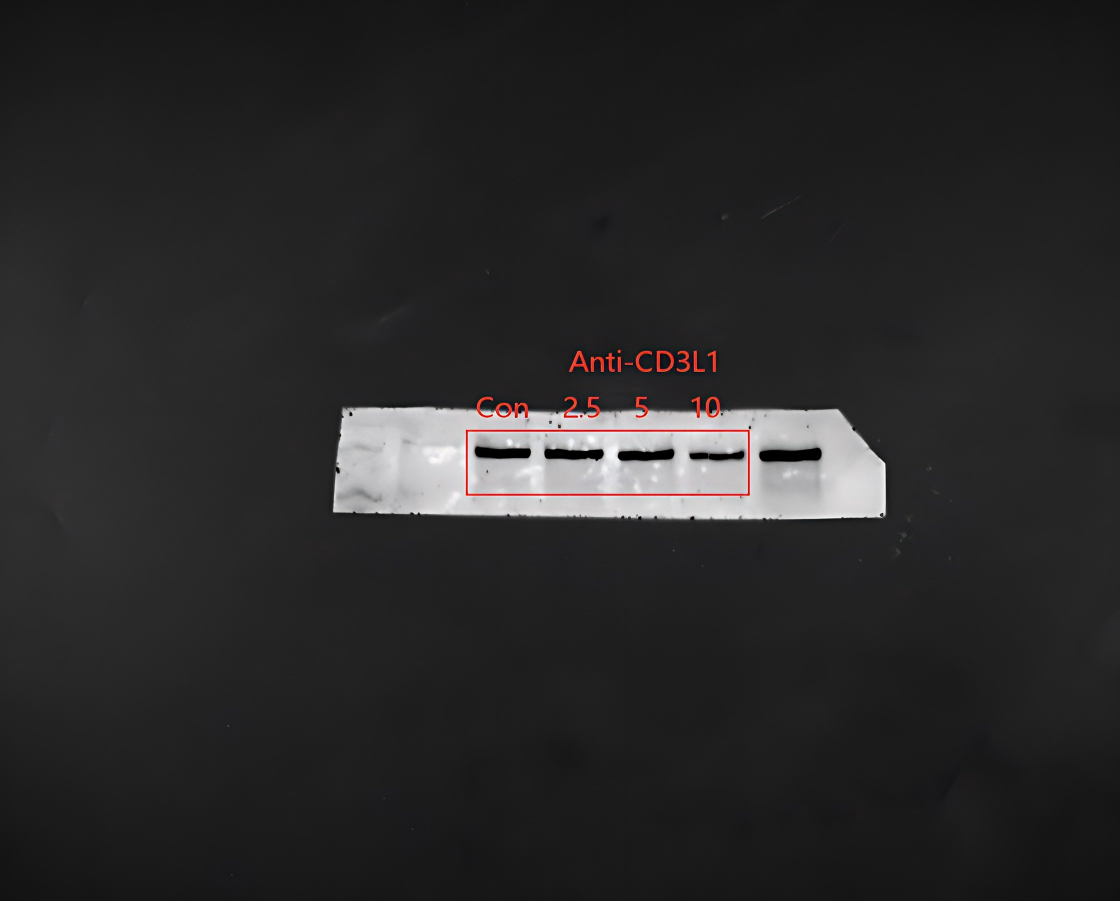

Supplement: Supplementary file 6 — Source data Fig. 3 [file 44321_2026_451_MOESM6_ESM.zip › Fig.3B/PBMC pSTAT6 replicate 2.jpg]

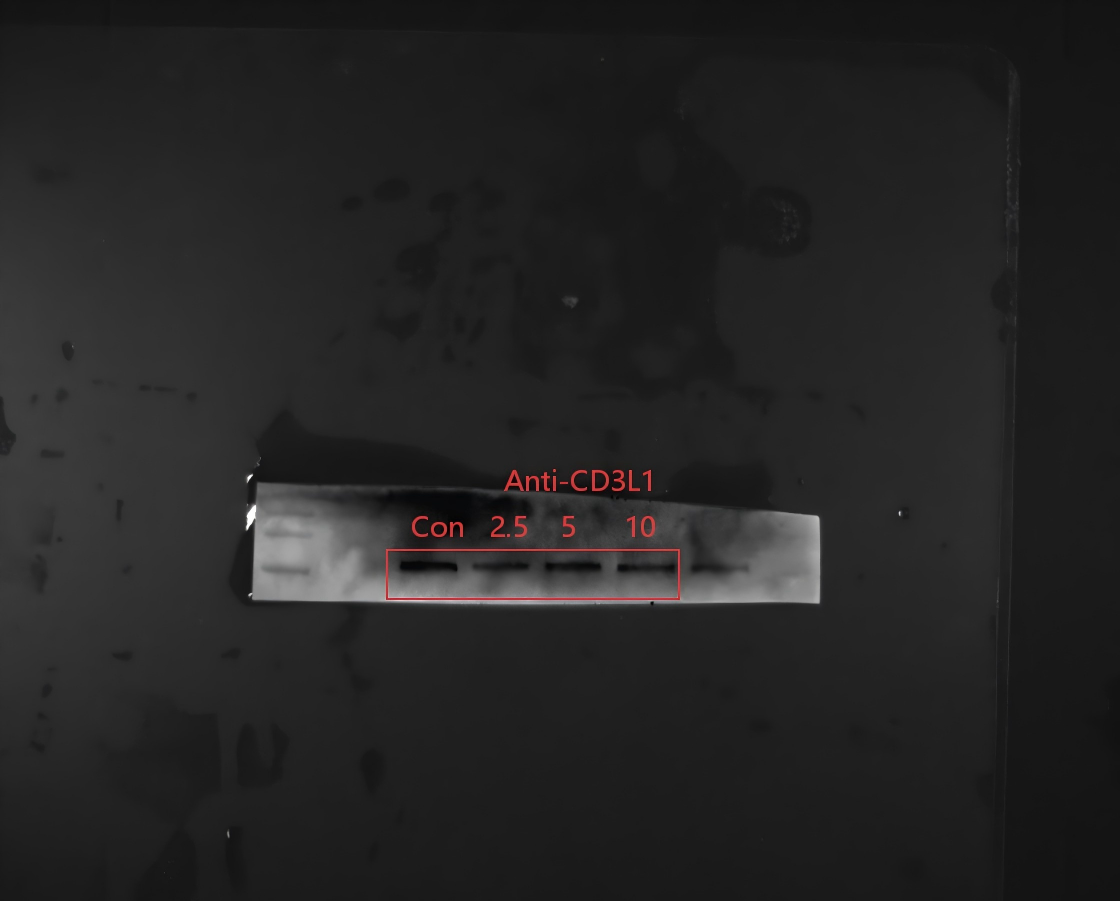

Supplement: Supplementary file 6 — Source data Fig. 3 [file 44321_2026_451_MOESM6_ESM.zip › Fig.3B/PBMC pSTAT6 replicate 3.jpg]

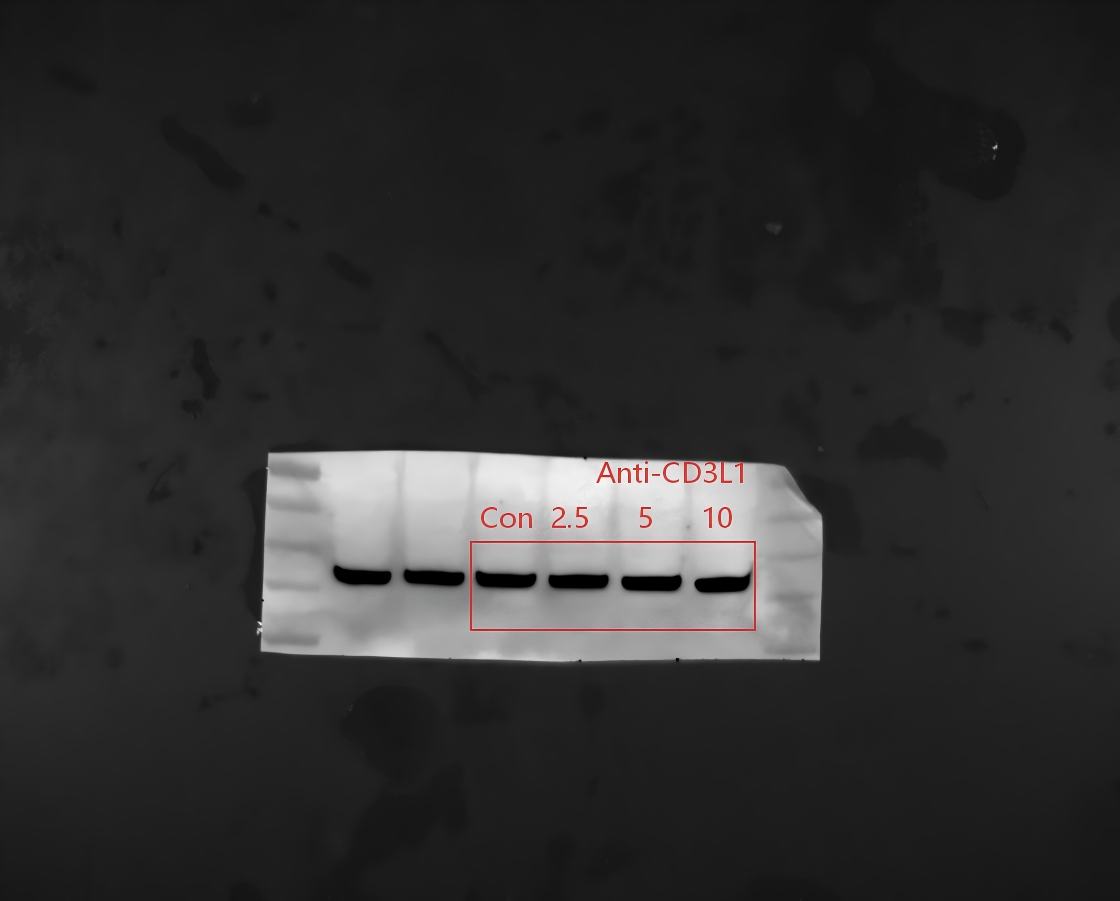

Supplement: Supplementary file 6 — Source data Fig. 3 [file 44321_2026_451_MOESM6_ESM.zip › Fig.3B/T G.jpg]

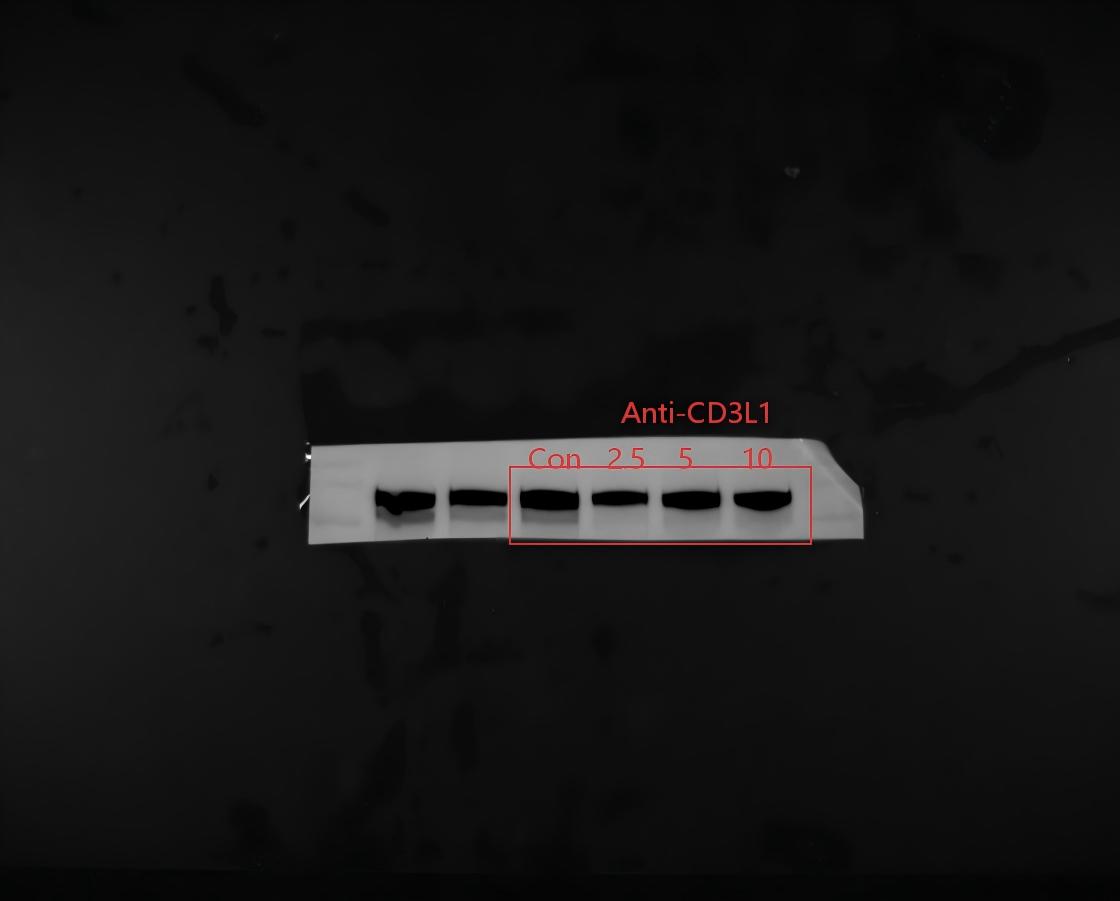

Supplement: Supplementary file 6 — Source data Fig. 3 [file 44321_2026_451_MOESM6_ESM.zip › Fig.3B/T JAK2(1).jpg]

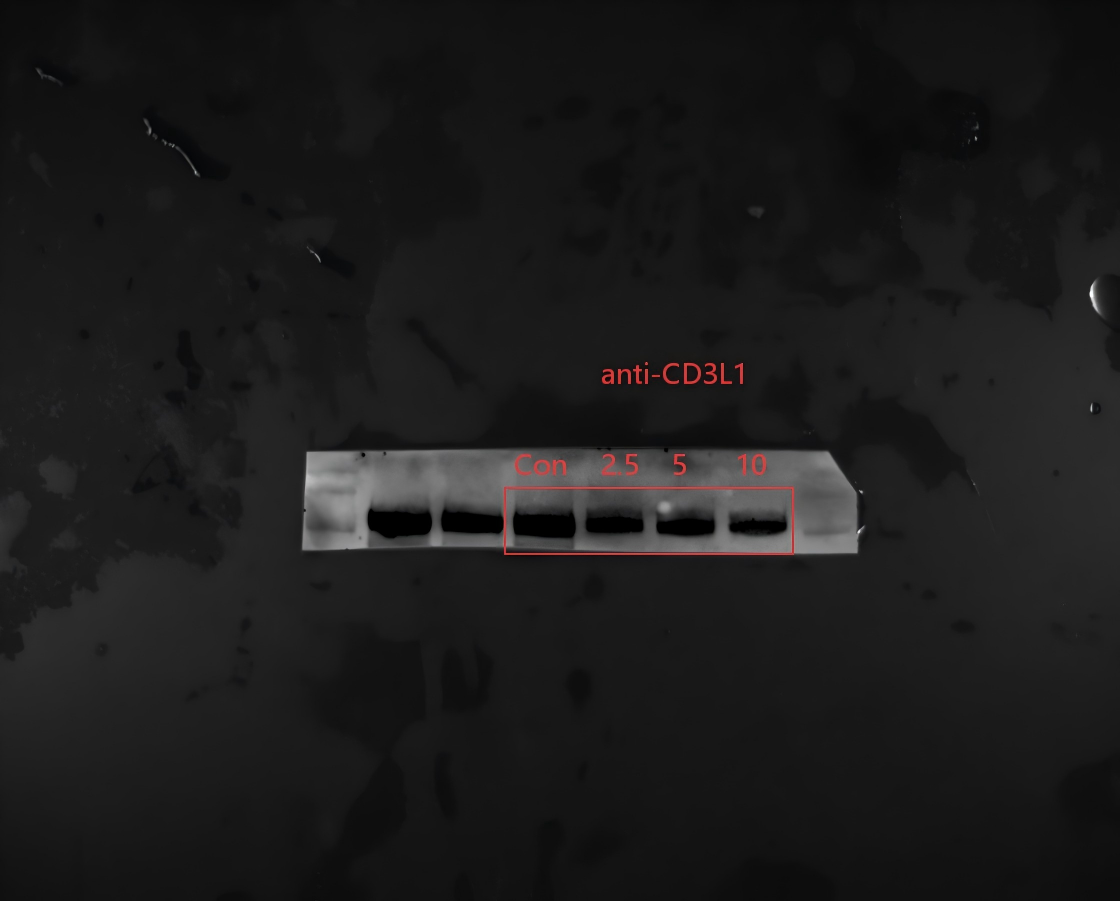

Supplement: Supplementary file 6 — Source data Fig. 3 [file 44321_2026_451_MOESM6_ESM.zip › Fig.3B/T PSTAT6 2.jpg]

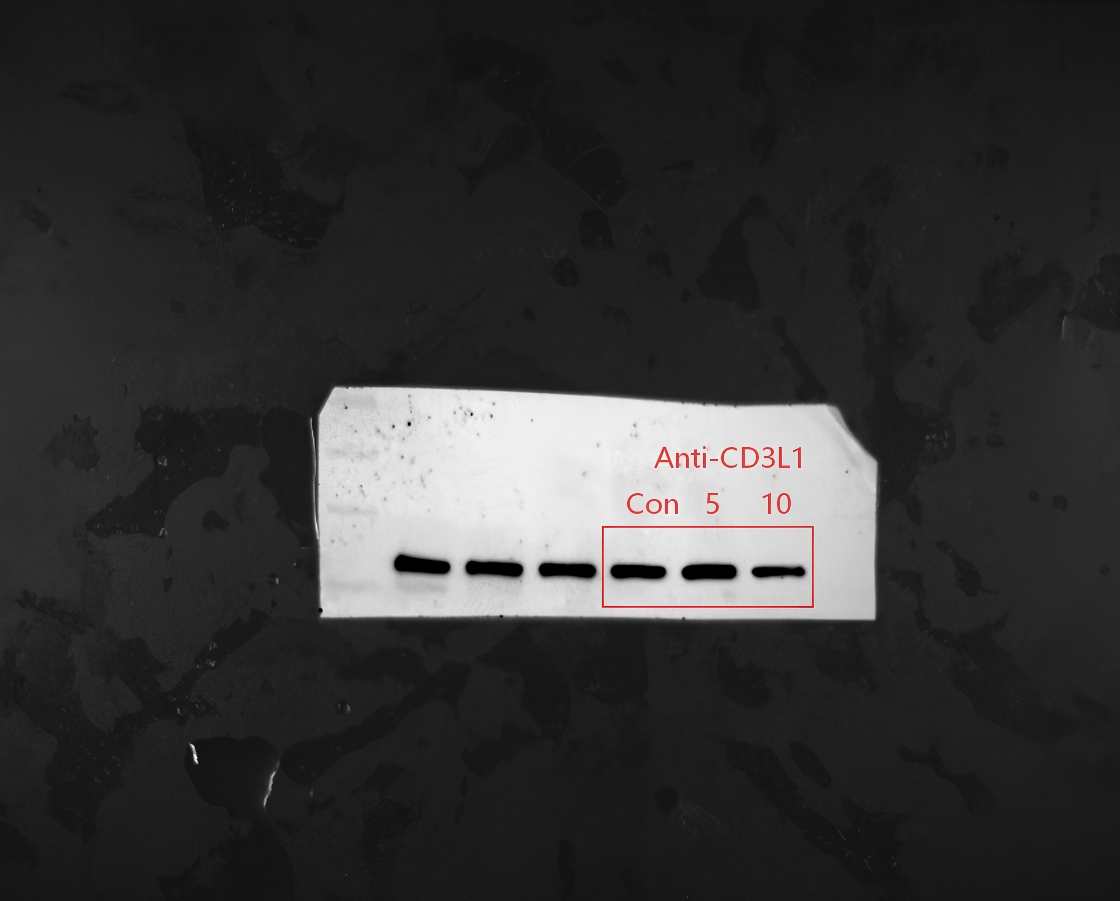

Supplement: Supplementary file 6 — Source data Fig. 3 [file 44321_2026_451_MOESM6_ESM.zip › Fig.3B/THP-1 GAPDH replicate 2.jpg]

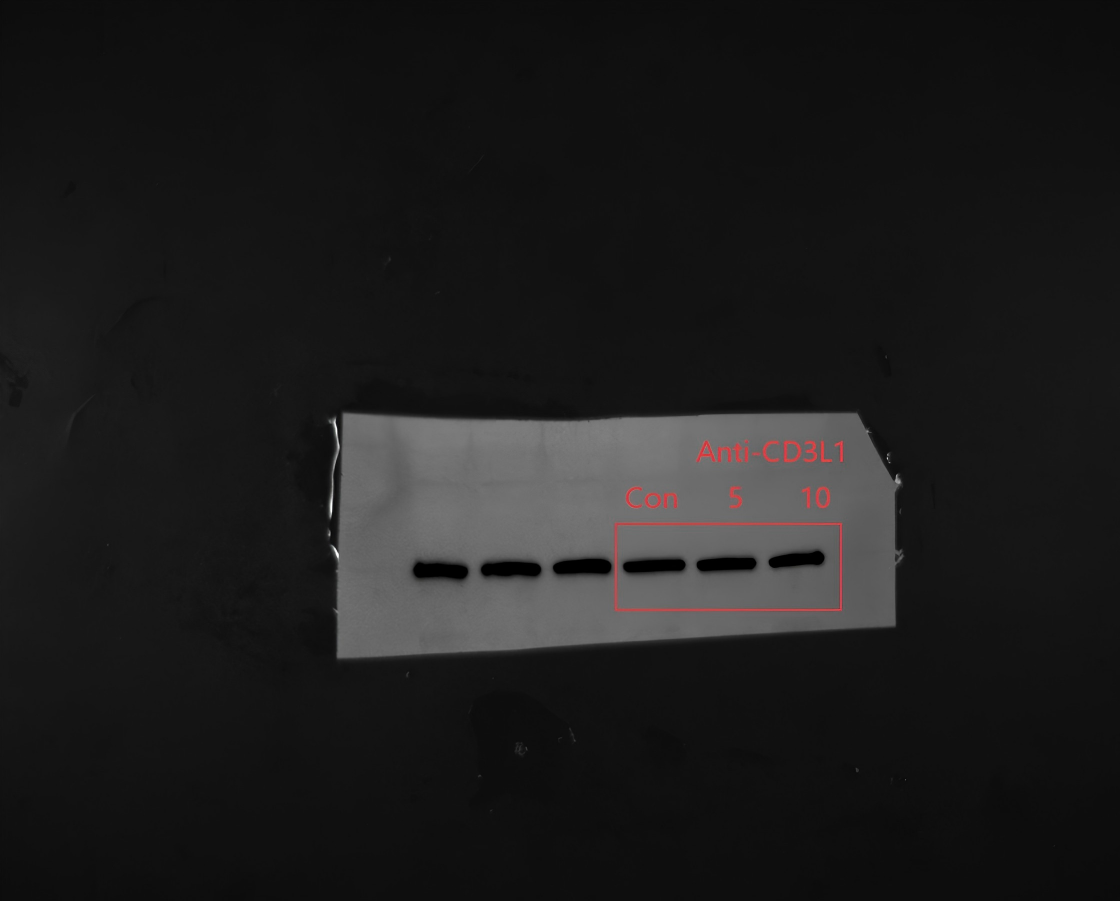

Supplement: Supplementary file 6 — Source data Fig. 3 [file 44321_2026_451_MOESM6_ESM.zip › Fig.3B/THP-1 GAPDH replicate 3.jpg]

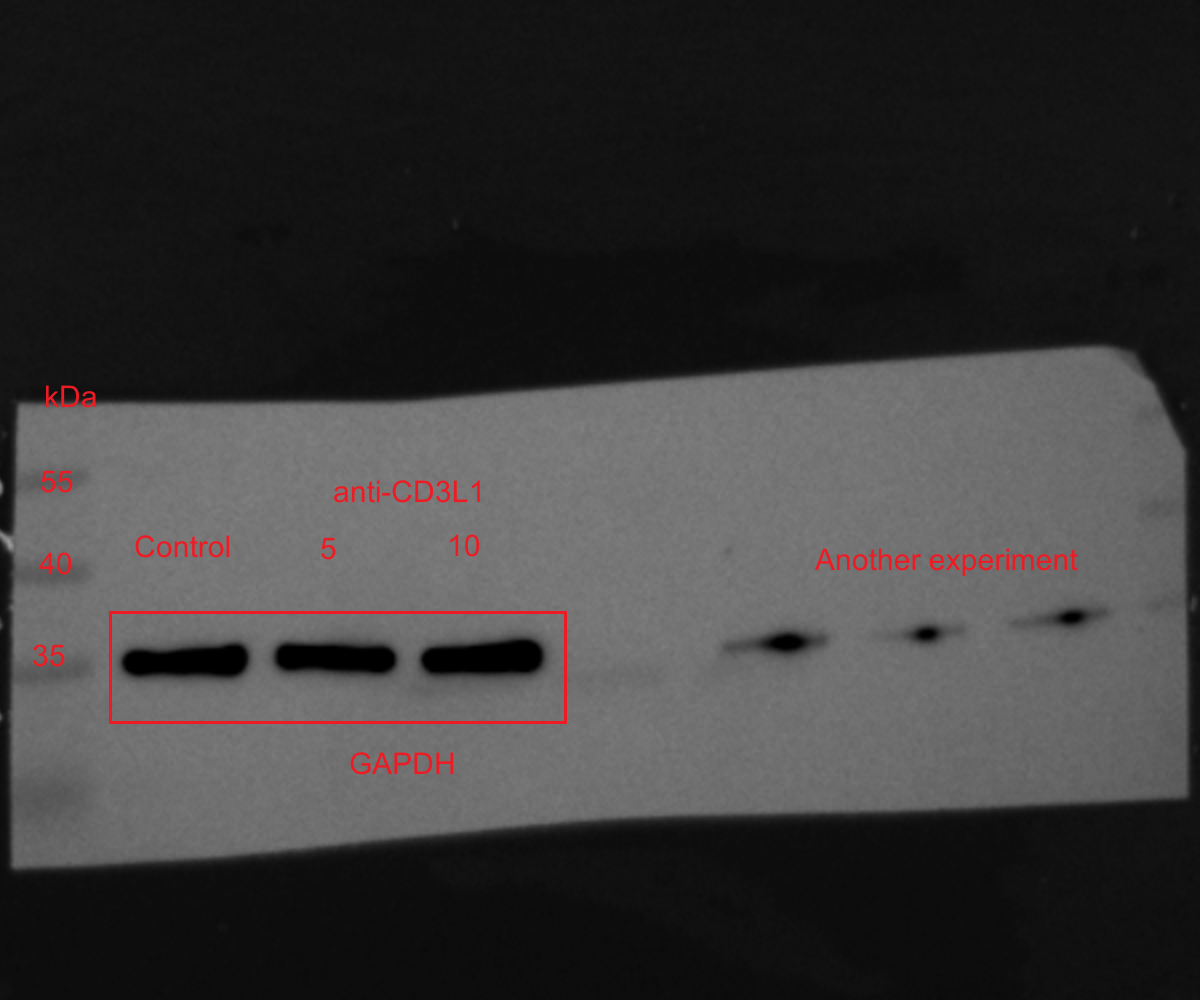

Supplement: Supplementary file 6 — Source data Fig. 3 [file 44321_2026_451_MOESM6_ESM.zip › Fig.3B/THP-1 GAPDH.png]

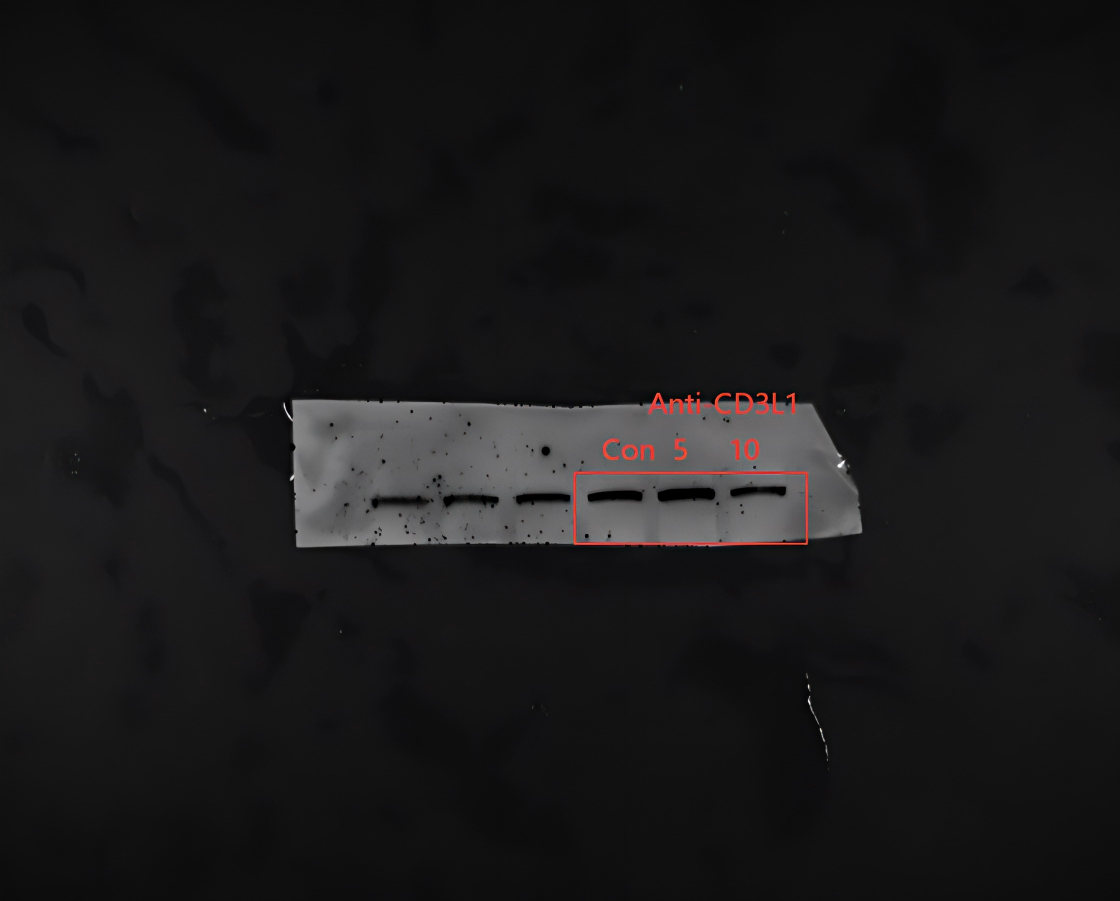

Supplement: Supplementary file 6 — Source data Fig. 3 [file 44321_2026_451_MOESM6_ESM.zip › Fig.3B/THP-1 STAT6 replicate 2.jpg]

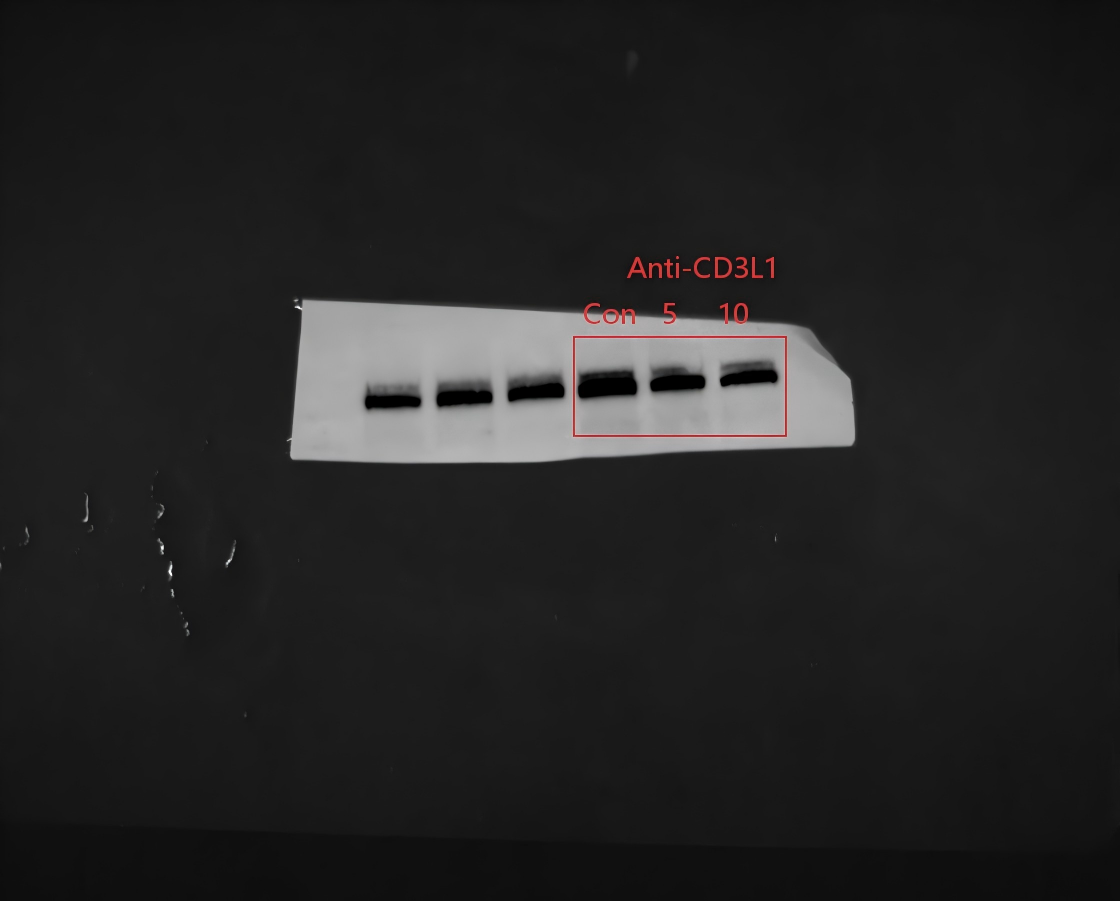

Supplement: Supplementary file 6 — Source data Fig. 3 [file 44321_2026_451_MOESM6_ESM.zip › Fig.3B/THP-1 STAT6 replicate3.jpg]

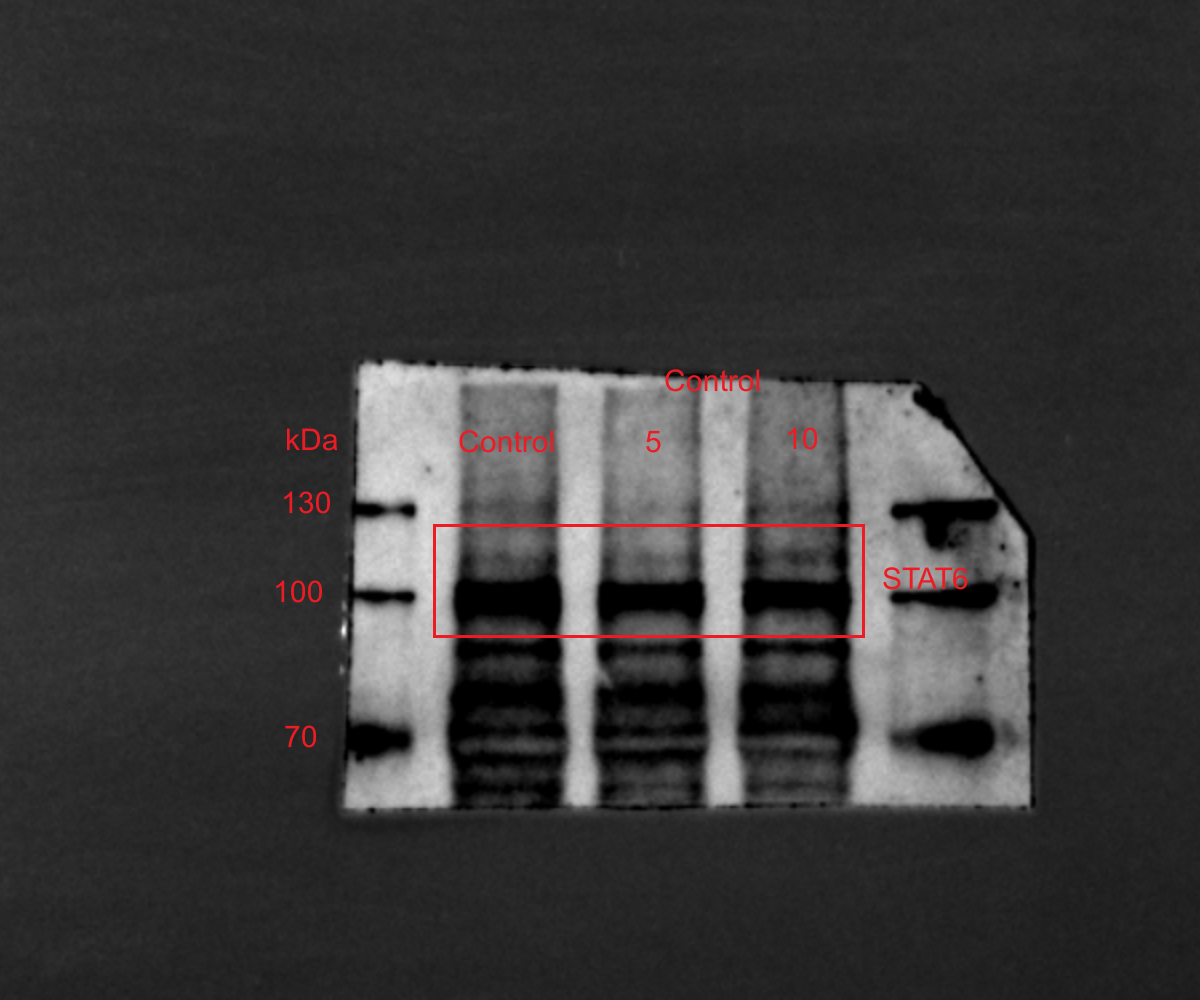

Supplement: Supplementary file 6 — Source data Fig. 3 [file 44321_2026_451_MOESM6_ESM.zip › Fig.3B/THP-1 STAT6.png]

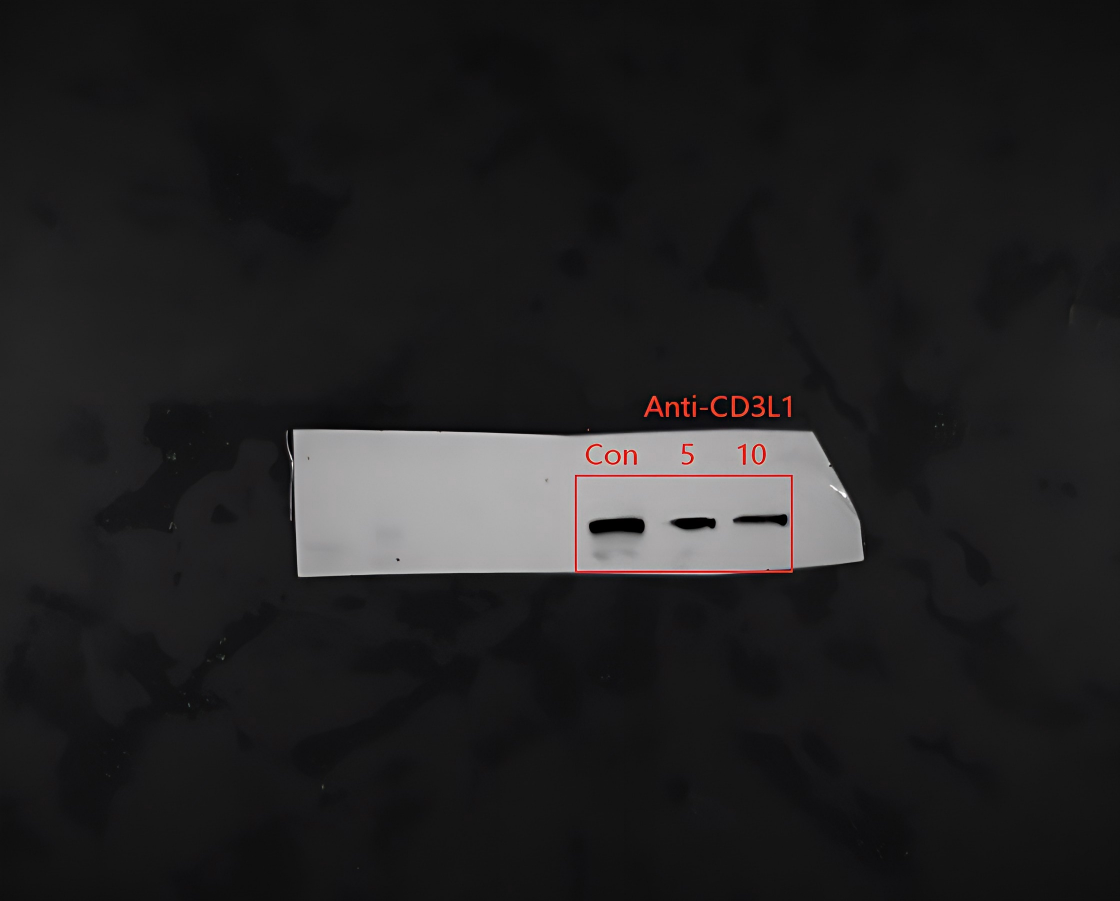

Supplement: Supplementary file 6 — Source data Fig. 3 [file 44321_2026_451_MOESM6_ESM.zip › Fig.3B/THP-1 pSTAT6 replicate 2.jpg]

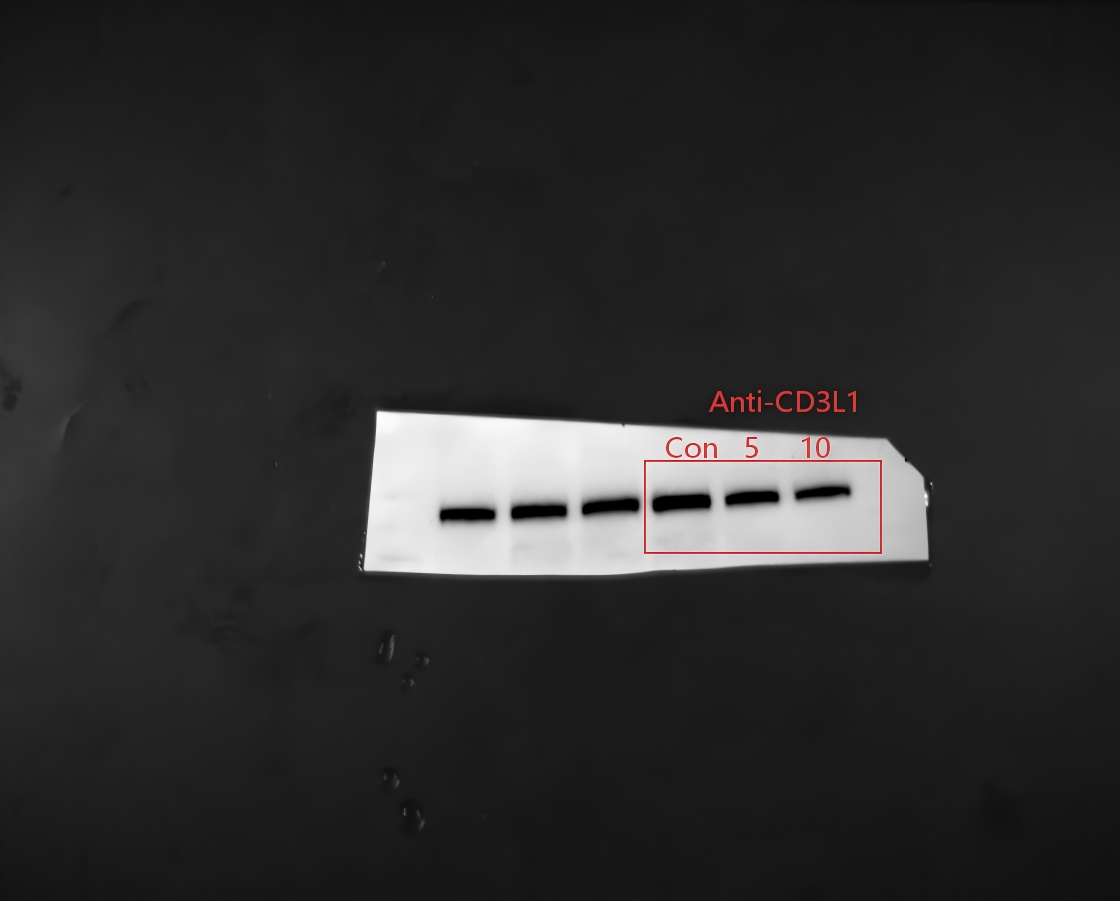

Supplement: Supplementary file 6 — Source data Fig. 3 [file 44321_2026_451_MOESM6_ESM.zip › Fig.3B/THP-1 pSTAT6 replicate 3.jpg]

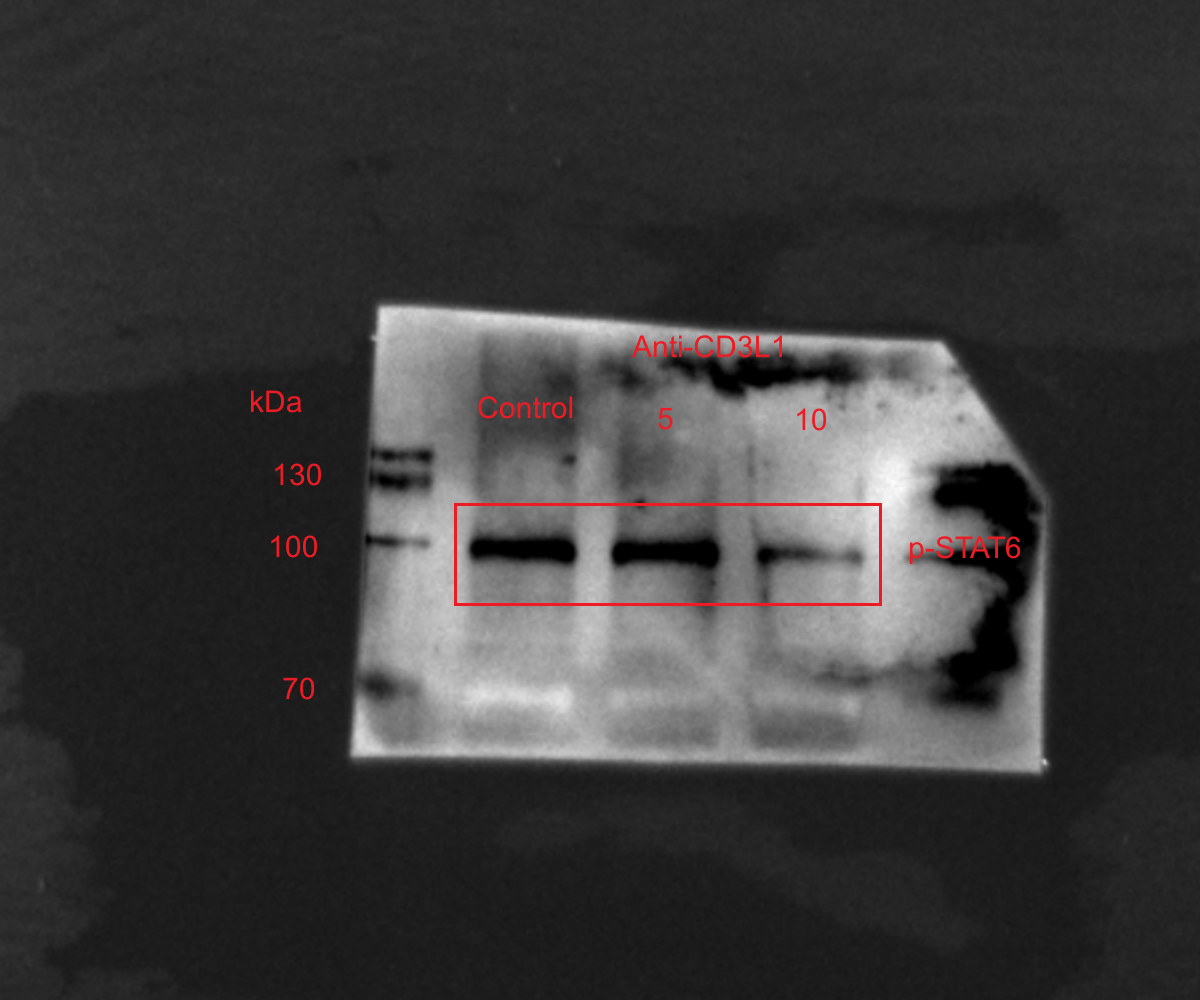

Supplement: Supplementary file 6 — Source data Fig. 3 [file 44321_2026_451_MOESM6_ESM.zip › Fig.3B/THP-1 pSTAT6.png]

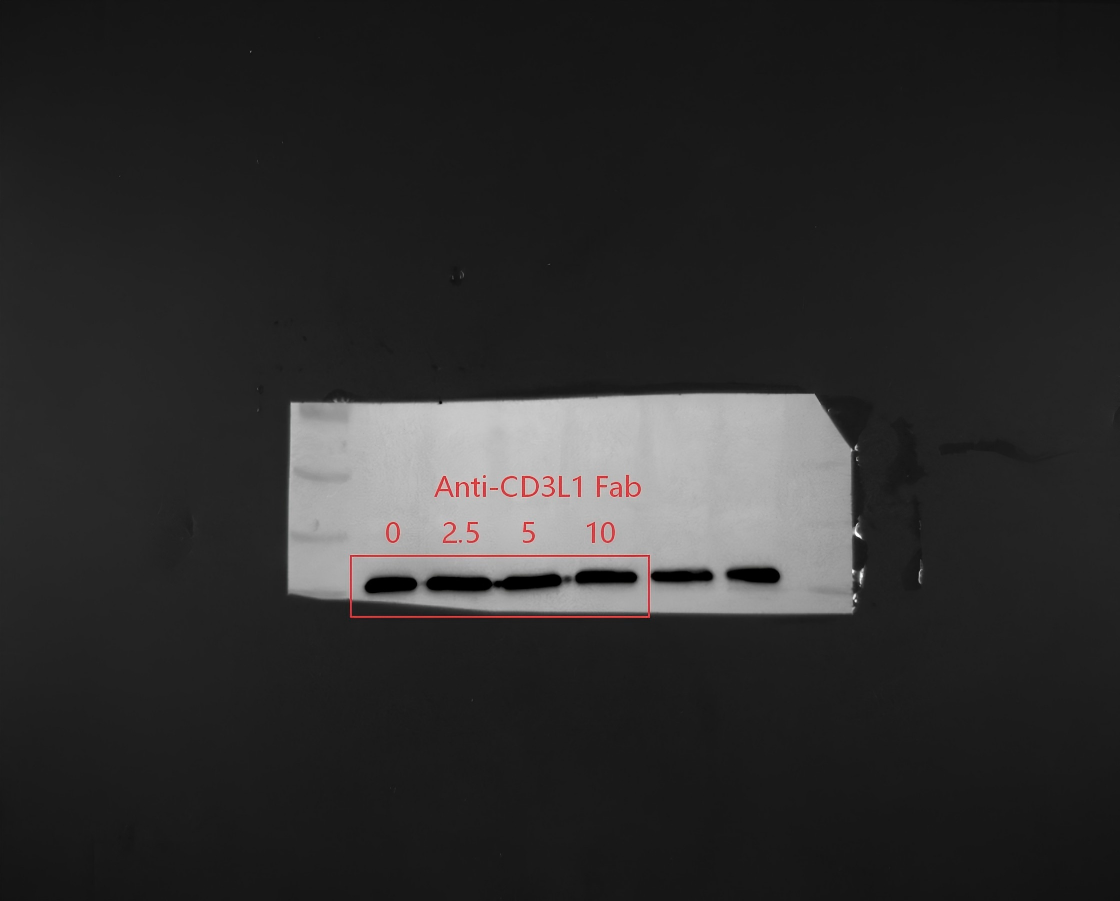

Supplement: Supplementary file 7 — Source data Fig. 4 [file 44321_2026_451_MOESM7_ESM.zip › Fig.4A/Fab GAPDH replicate 2.jpg]

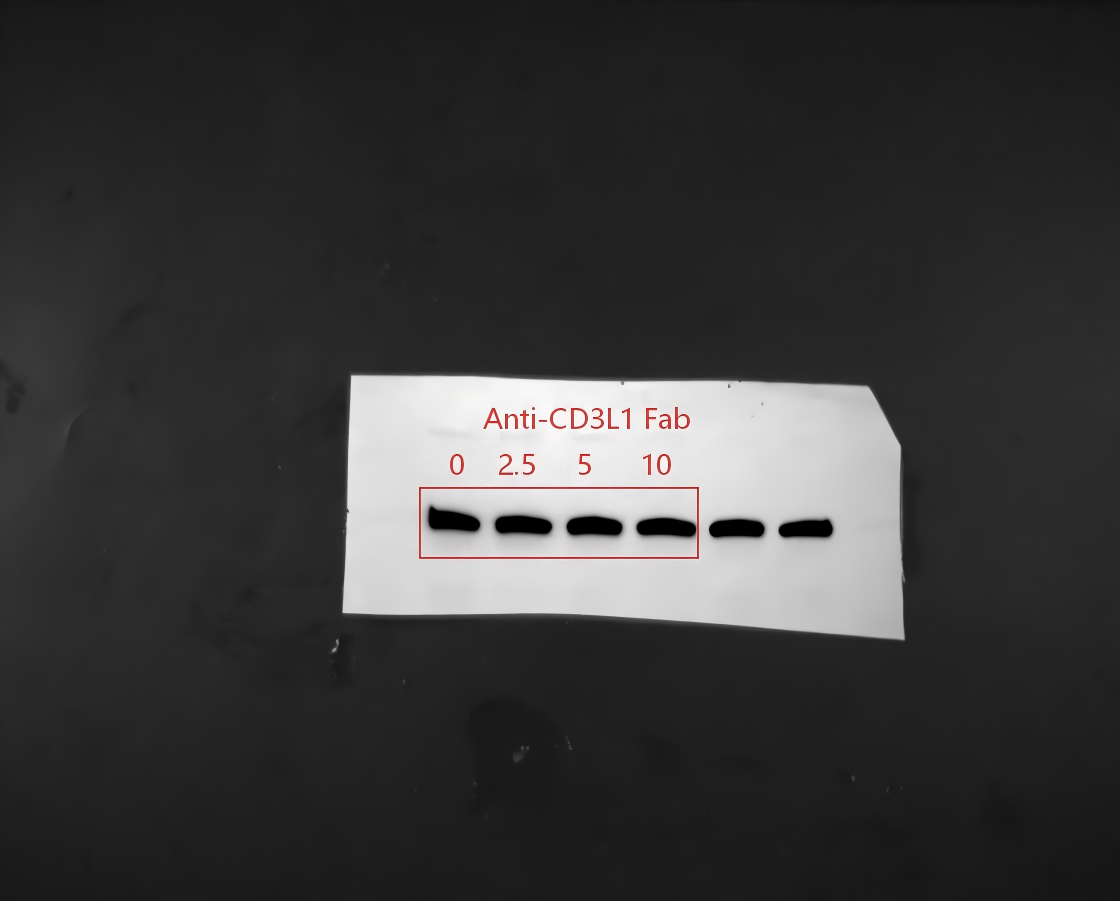

Supplement: Supplementary file 7 — Source data Fig. 4 [file 44321_2026_451_MOESM7_ESM.zip › Fig.4A/Fab GAPDH replicate 3.jpg]

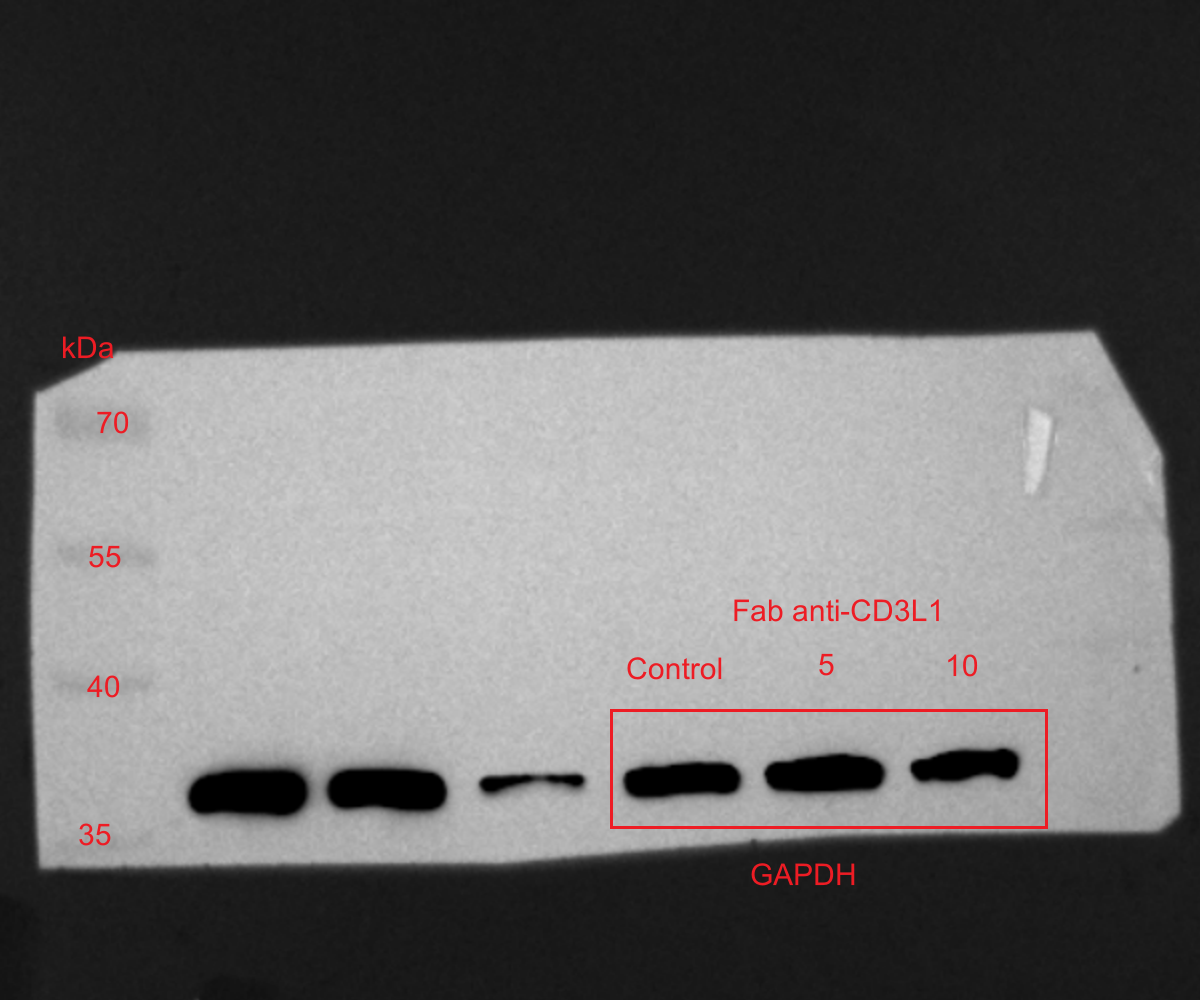

Supplement: Supplementary file 7 — Source data Fig. 4 [file 44321_2026_451_MOESM7_ESM.zip › Fig.4A/Fab GAPDH.png]

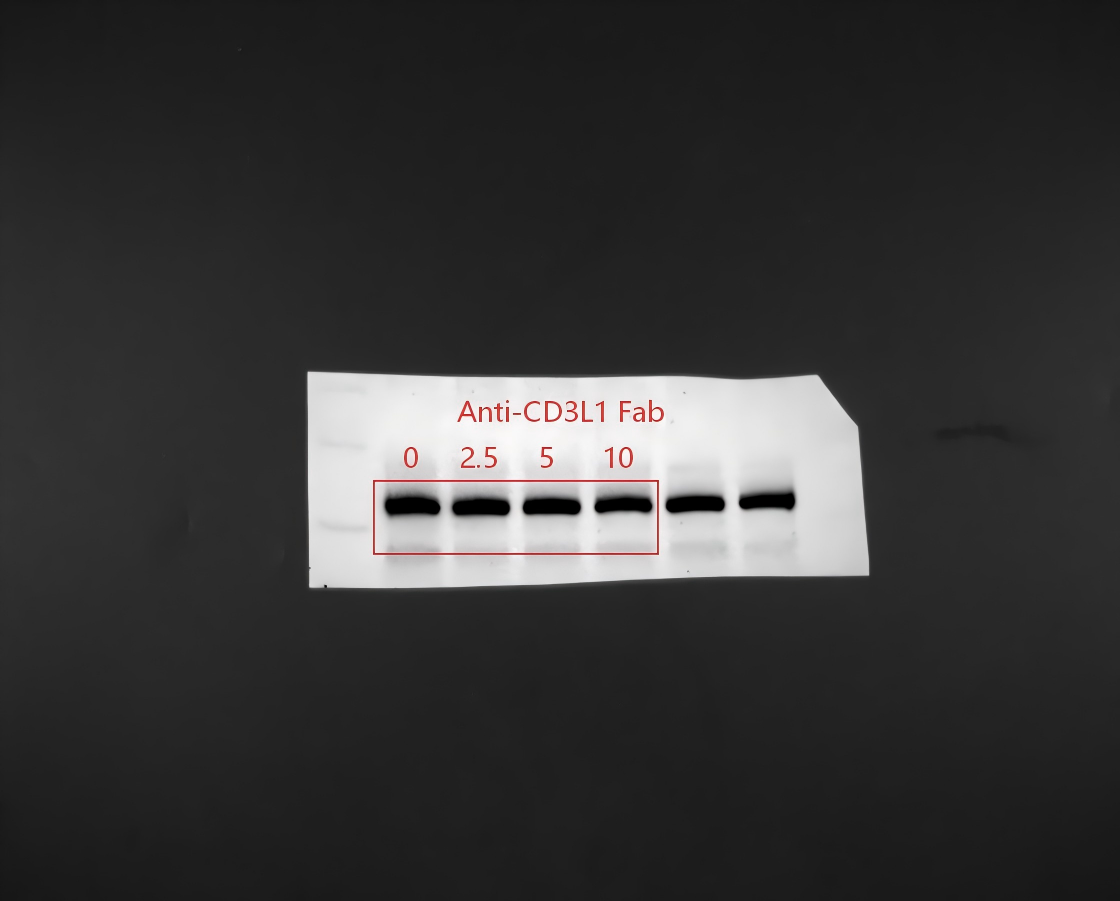

Supplement: Supplementary file 7 — Source data Fig. 4 [file 44321_2026_451_MOESM7_ESM.zip › Fig.4A/Fab pSTAT6 replicate 2.jpg]

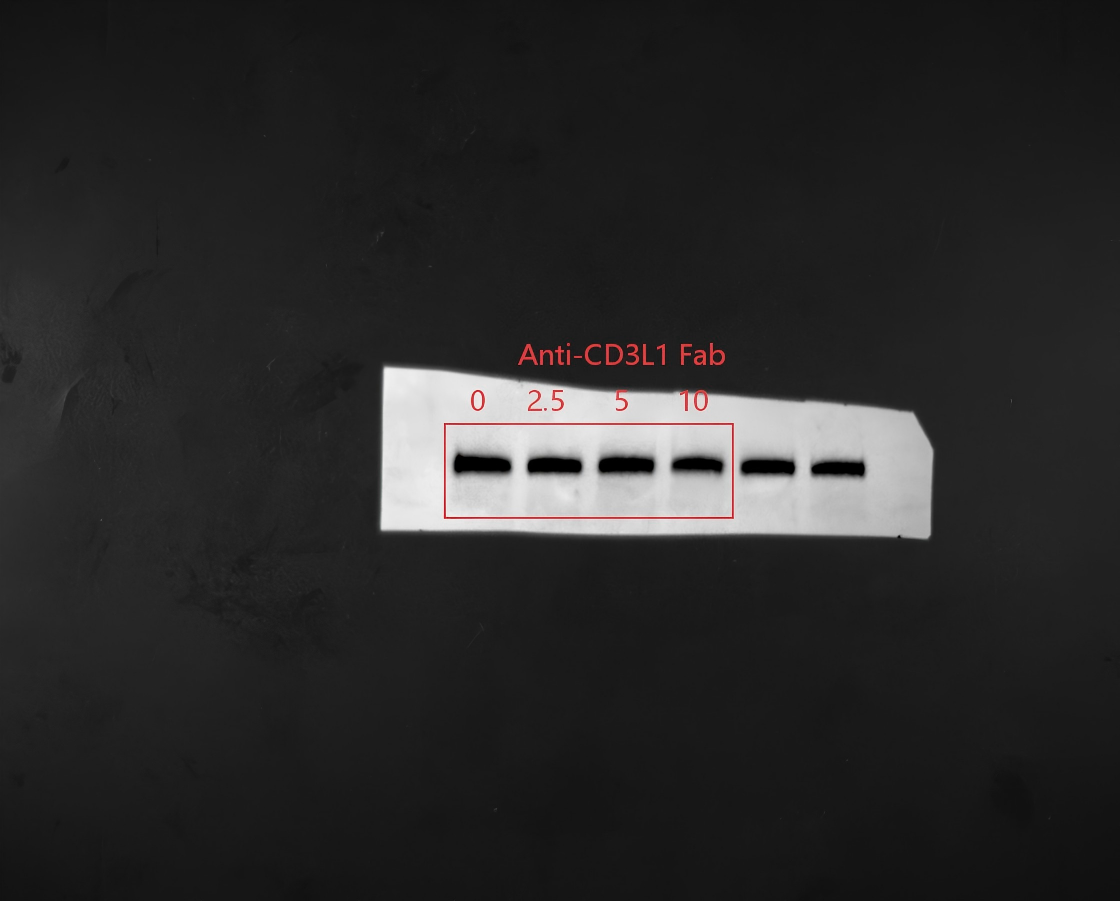

Supplement: Supplementary file 7 — Source data Fig. 4 [file 44321_2026_451_MOESM7_ESM.zip › Fig.4A/Fab pSTAT6 replicate 3.jpg]

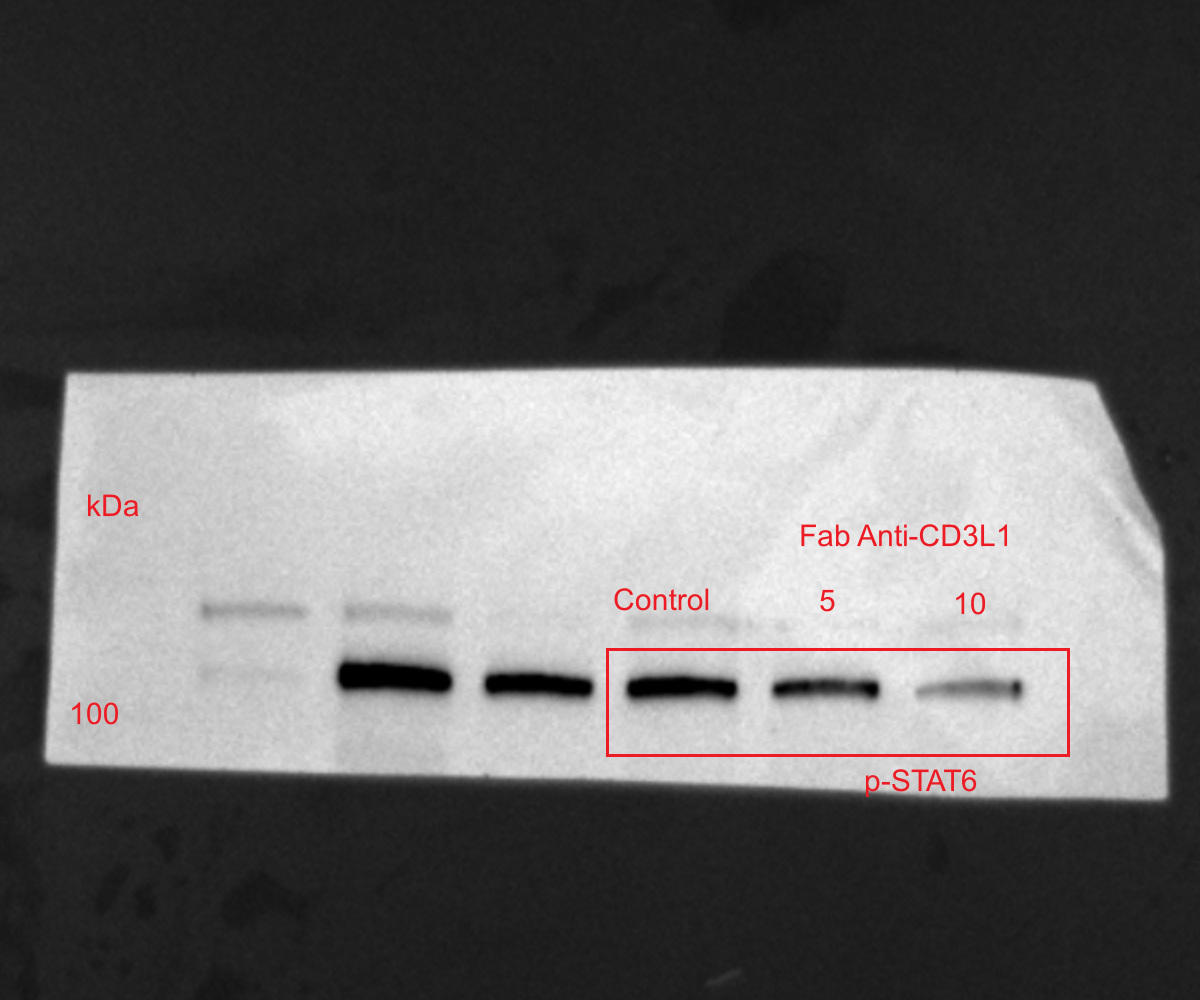

Supplement: Supplementary file 7 — Source data Fig. 4 [file 44321_2026_451_MOESM7_ESM.zip › Fig.4A/Fab pSTAT6.png]

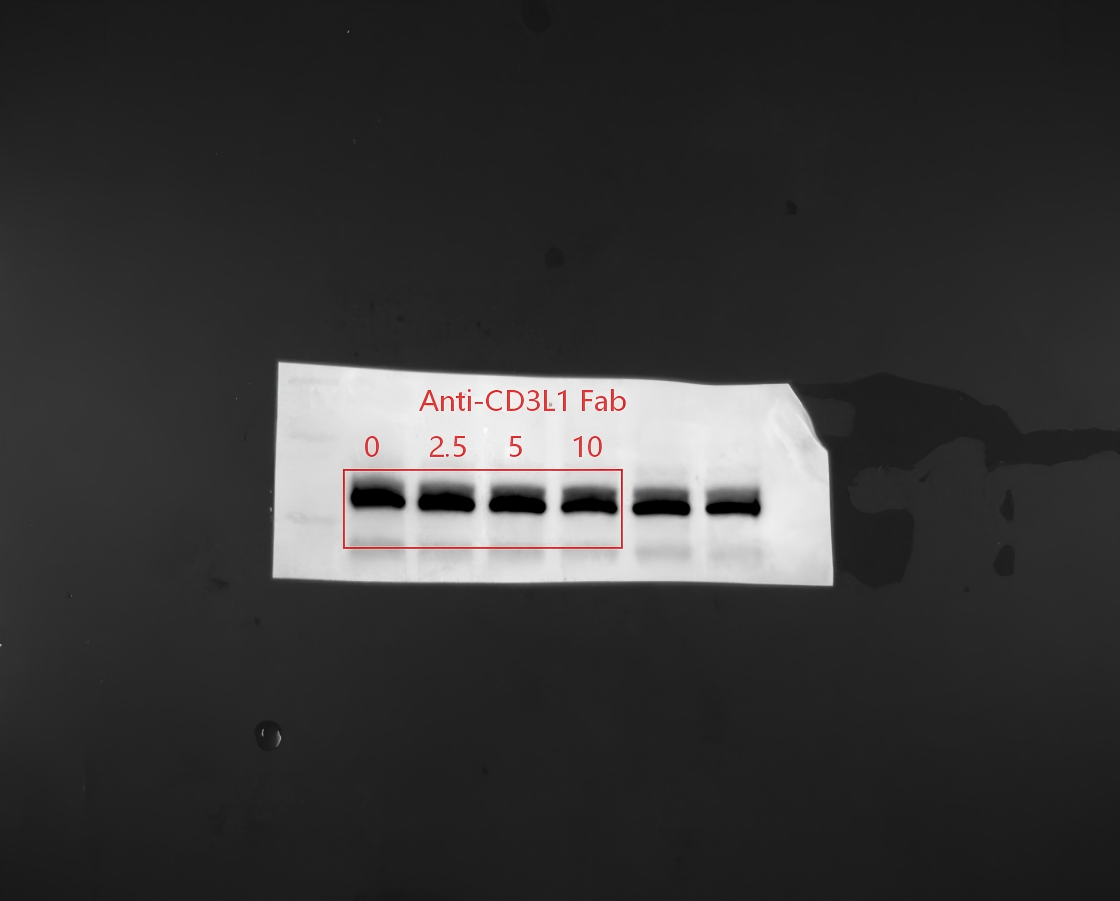

Supplement: Supplementary file 7 — Source data Fig. 4 [file 44321_2026_451_MOESM7_ESM.zip › Fig.4A/Fab STAT6 replicate 2.jpg]

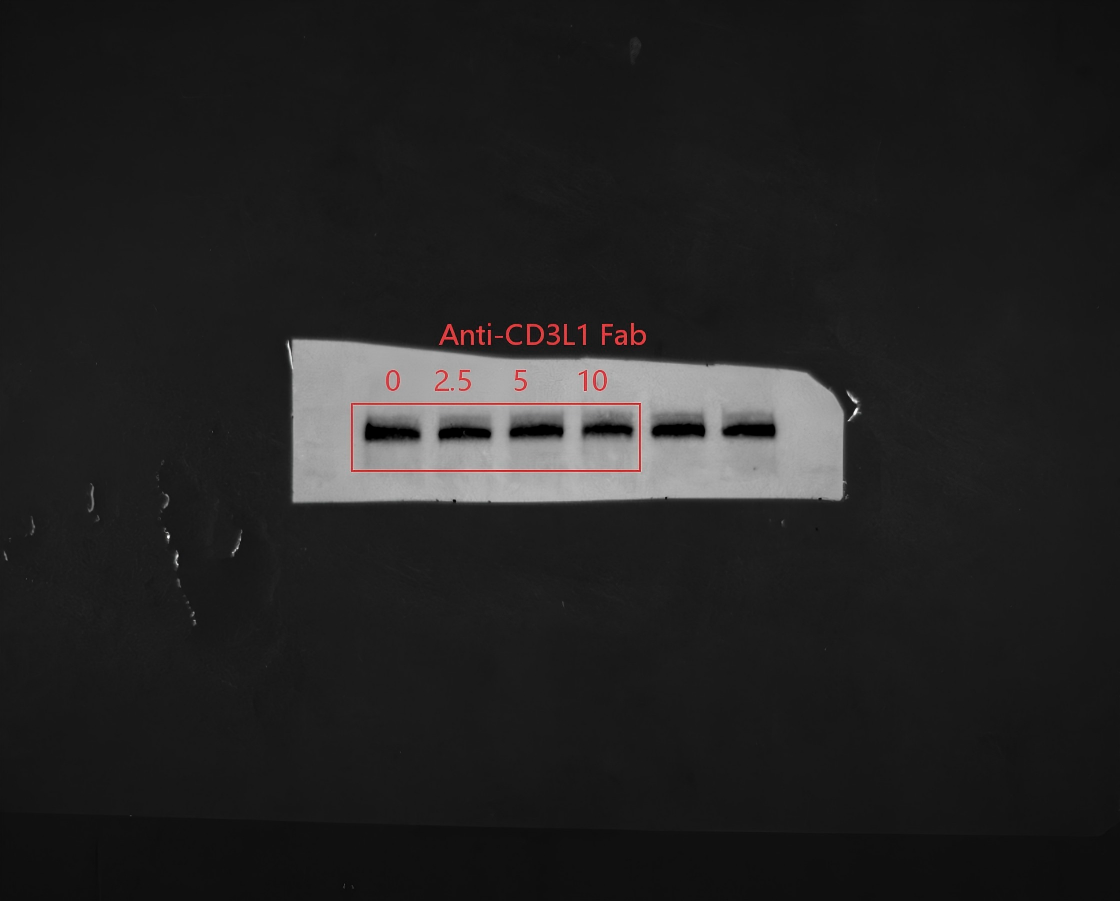

Supplement: Supplementary file 7 — Source data Fig. 4 [file 44321_2026_451_MOESM7_ESM.zip › Fig.4A/Fab STAT6 replicate 3.jpg]

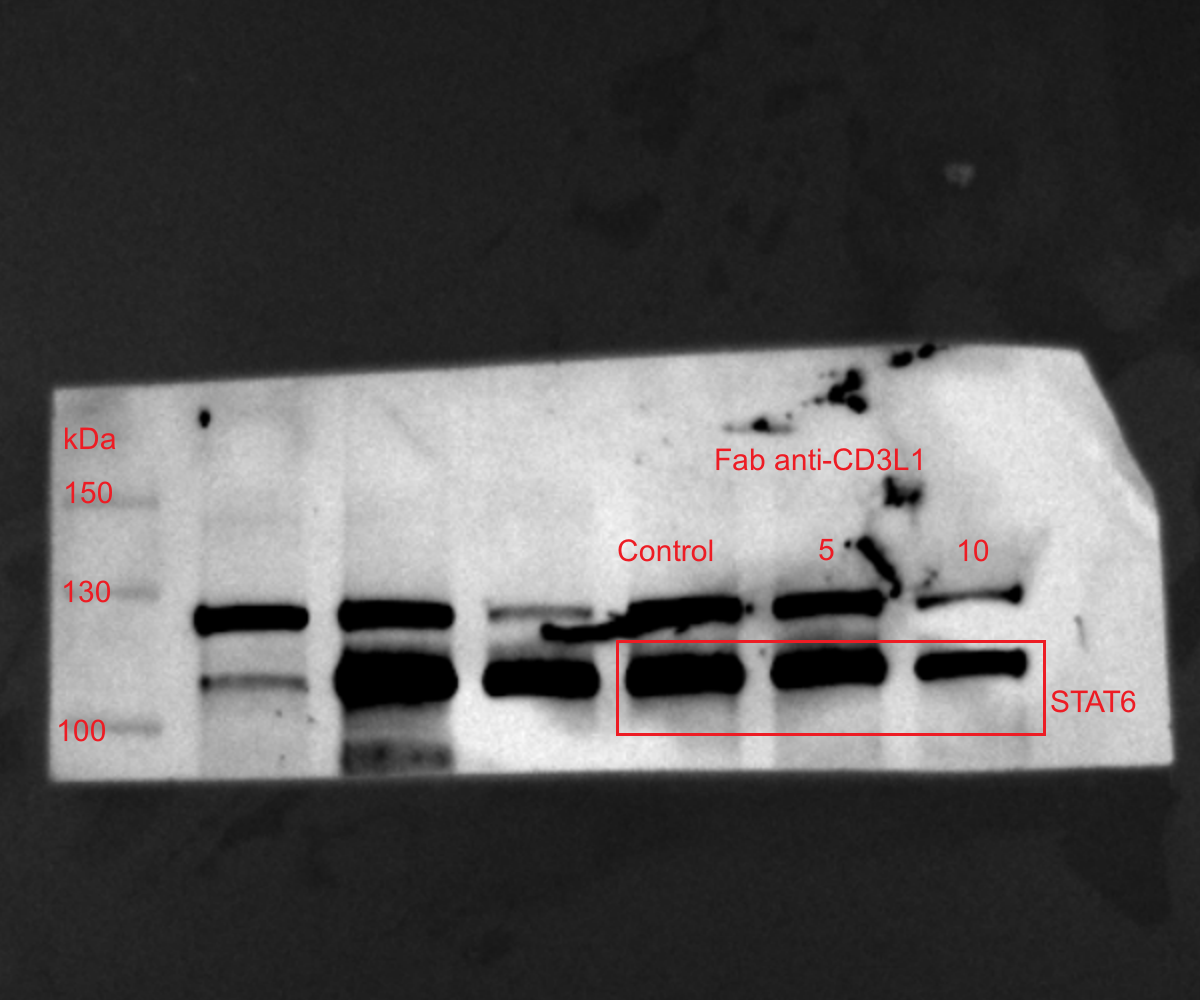

Supplement: Supplementary file 7 — Source data Fig. 4 [file 44321_2026_451_MOESM7_ESM.zip › Fig.4A/Fab STAT6.png]

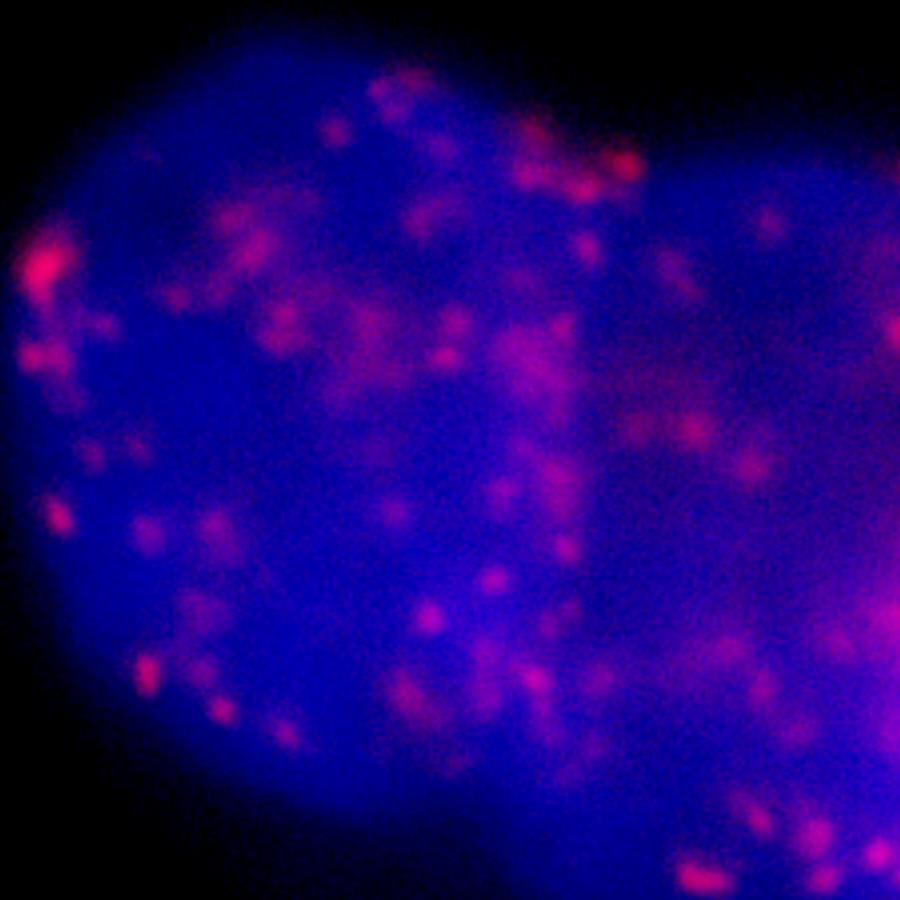

Supplement: Supplementary file 8 — Source data Fig. 5 [file 44321_2026_451_MOESM8_ESM.zip › Fig.5C/CD3L1-ECD-NRP2.png]

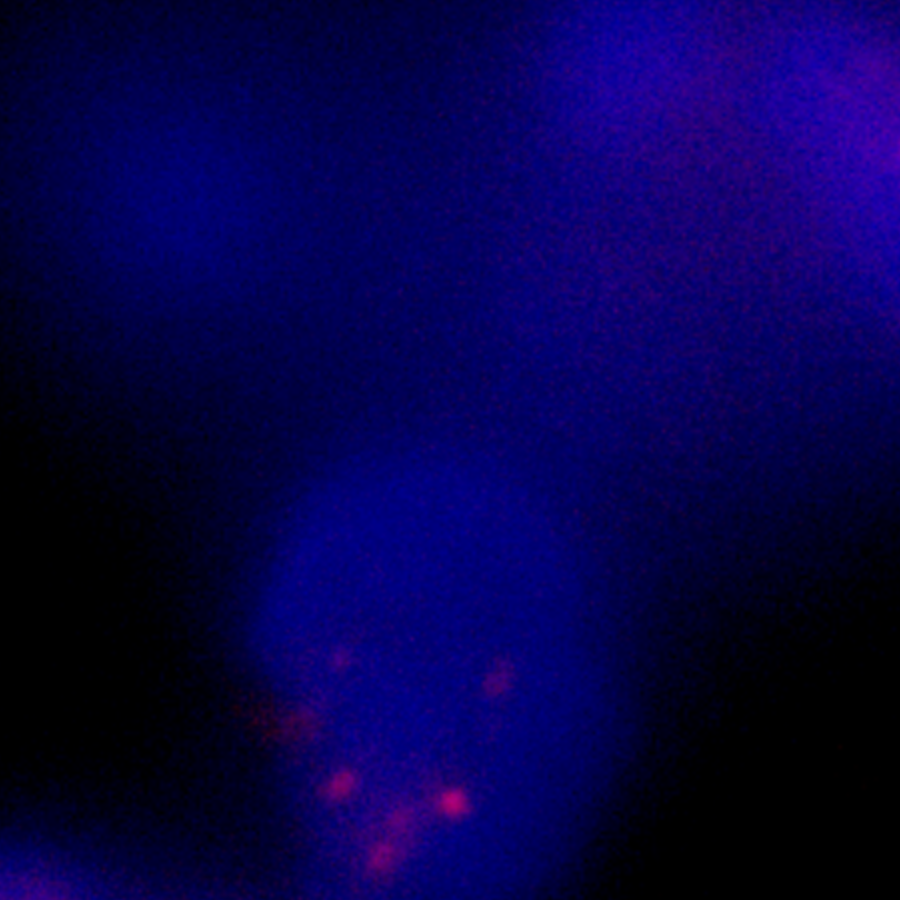

Supplement: Supplementary file 8 — Source data Fig. 5 [file 44321_2026_451_MOESM8_ESM.zip › Fig.5C/CD3L1-ICD-NRP2.png]

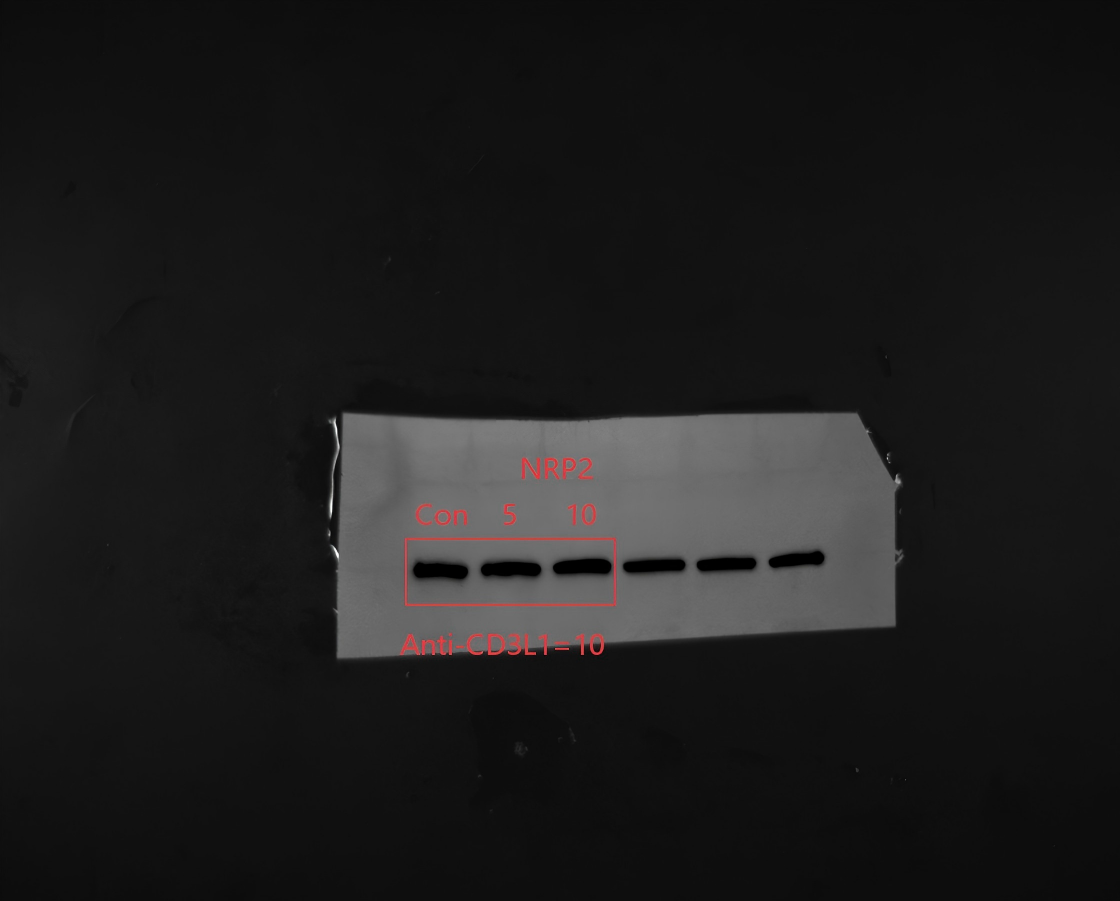

Supplement: Supplementary file 8 — Source data Fig. 5 [file 44321_2026_451_MOESM8_ESM.zip › Fig.5D/NRP2 GAPDH replicate 2.jpg]

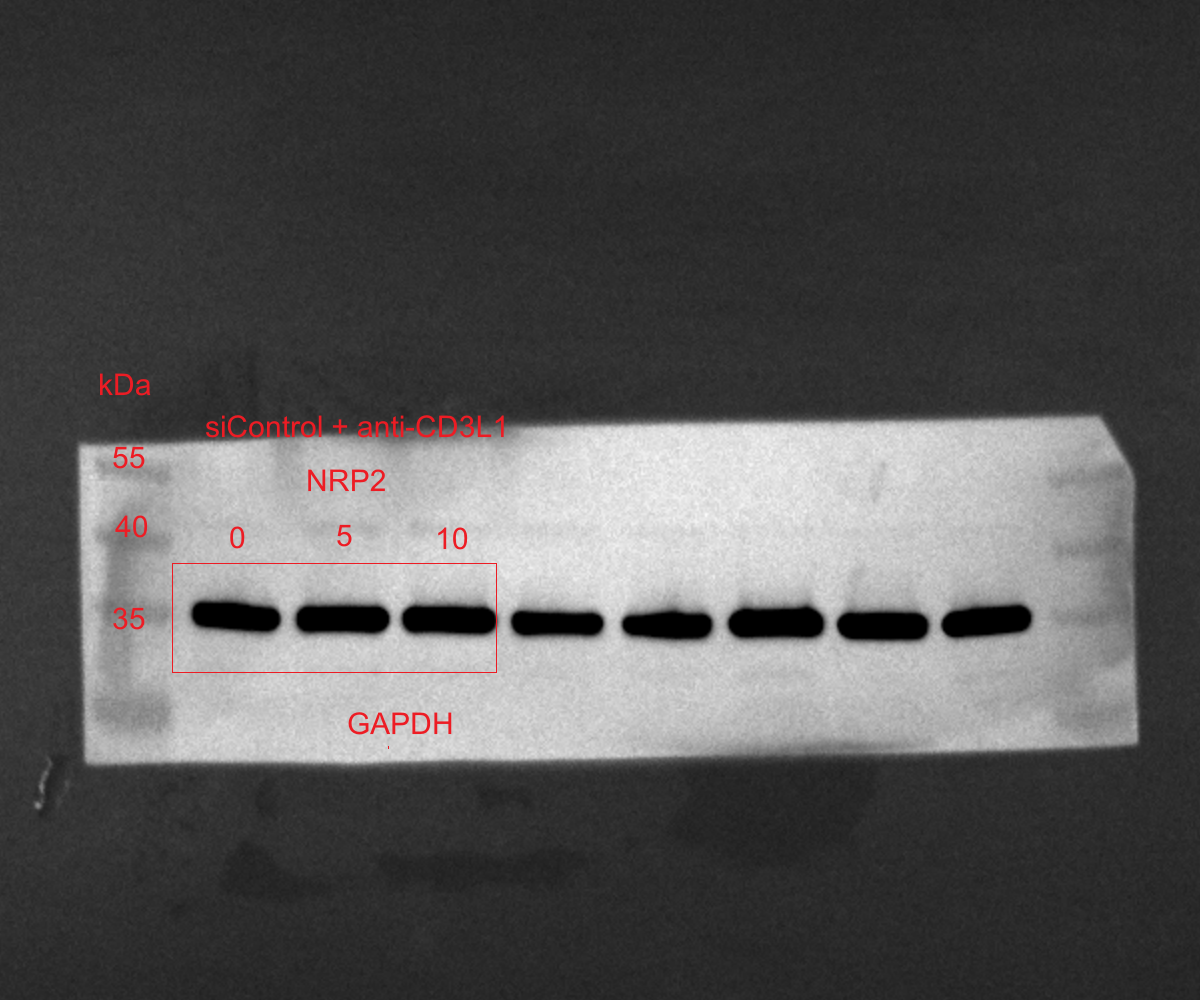

Supplement: Supplementary file 8 — Source data Fig. 5 [file 44321_2026_451_MOESM8_ESM.zip › Fig.5D/NRP2 GAPDH.png]

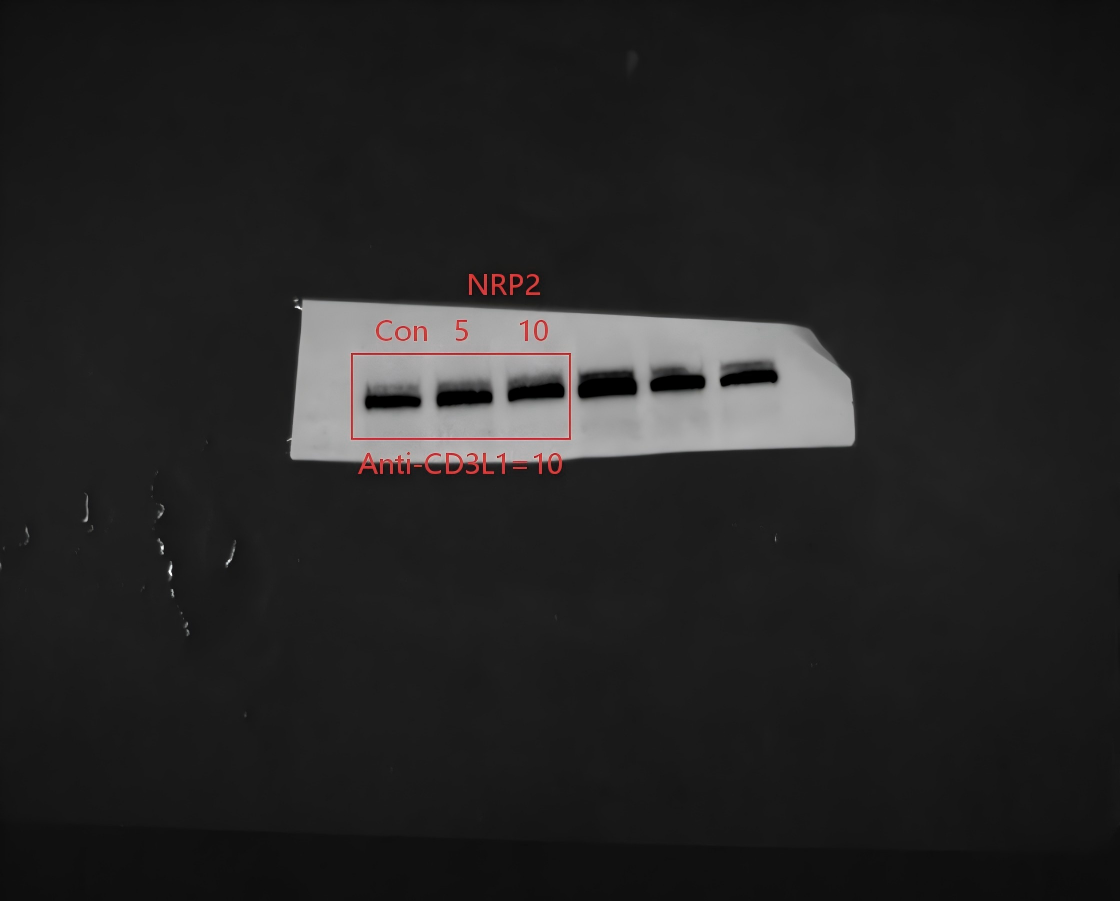

Supplement: Supplementary file 8 — Source data Fig. 5 [file 44321_2026_451_MOESM8_ESM.zip › Fig.5D/NRP2 STAT6 replicate 2.jpg]

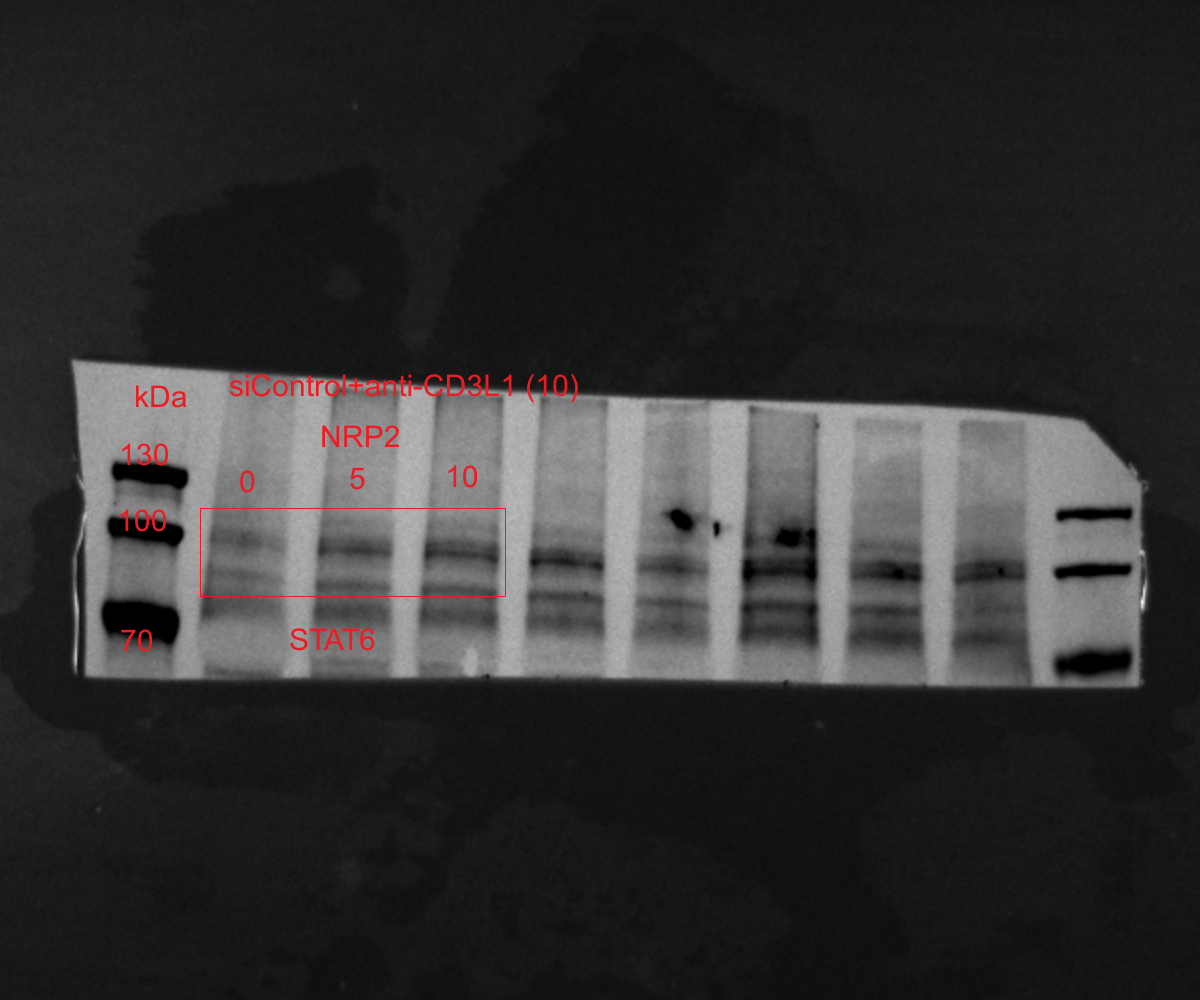

Supplement: Supplementary file 8 — Source data Fig. 5 [file 44321_2026_451_MOESM8_ESM.zip › Fig.5D/NRP2 STAT6.png]

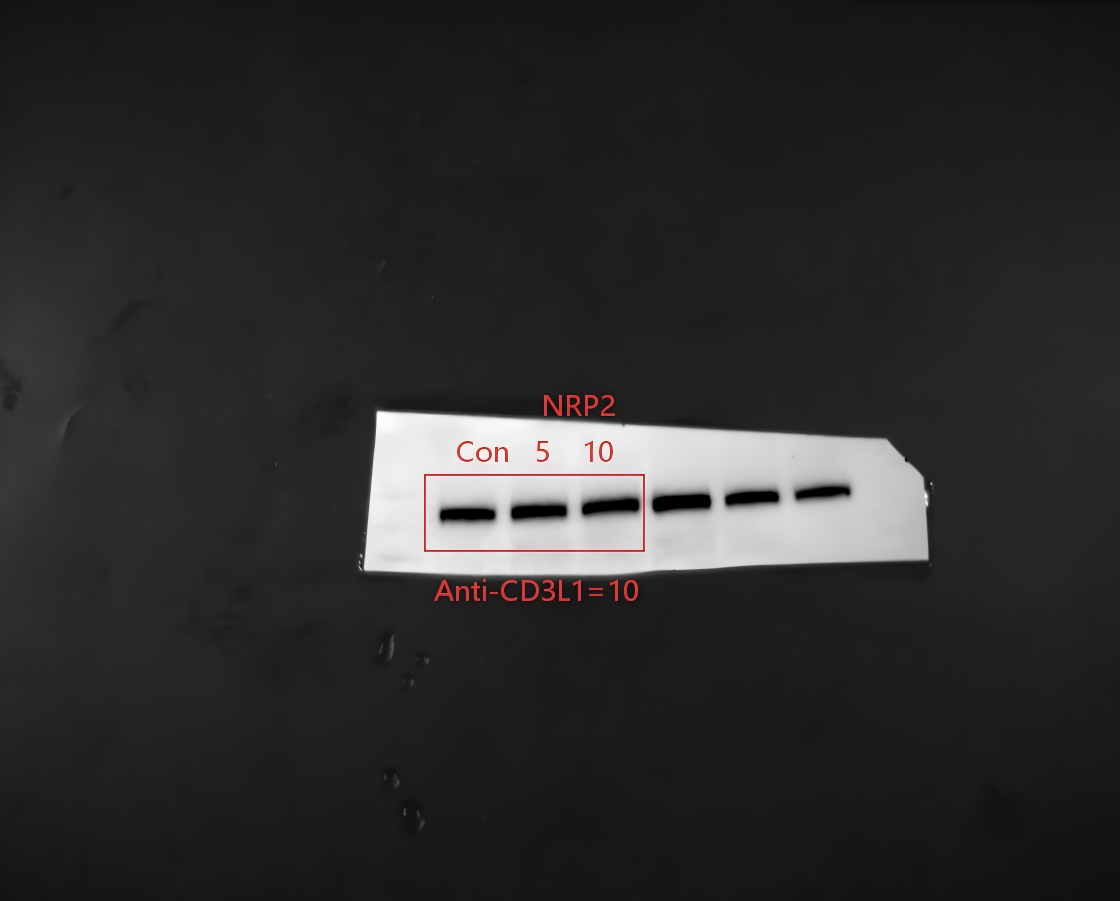

Supplement: Supplementary file 8 — Source data Fig. 5 [file 44321_2026_451_MOESM8_ESM.zip › Fig.5D/NRP2 pSTAT6 replicate 2.jpg]

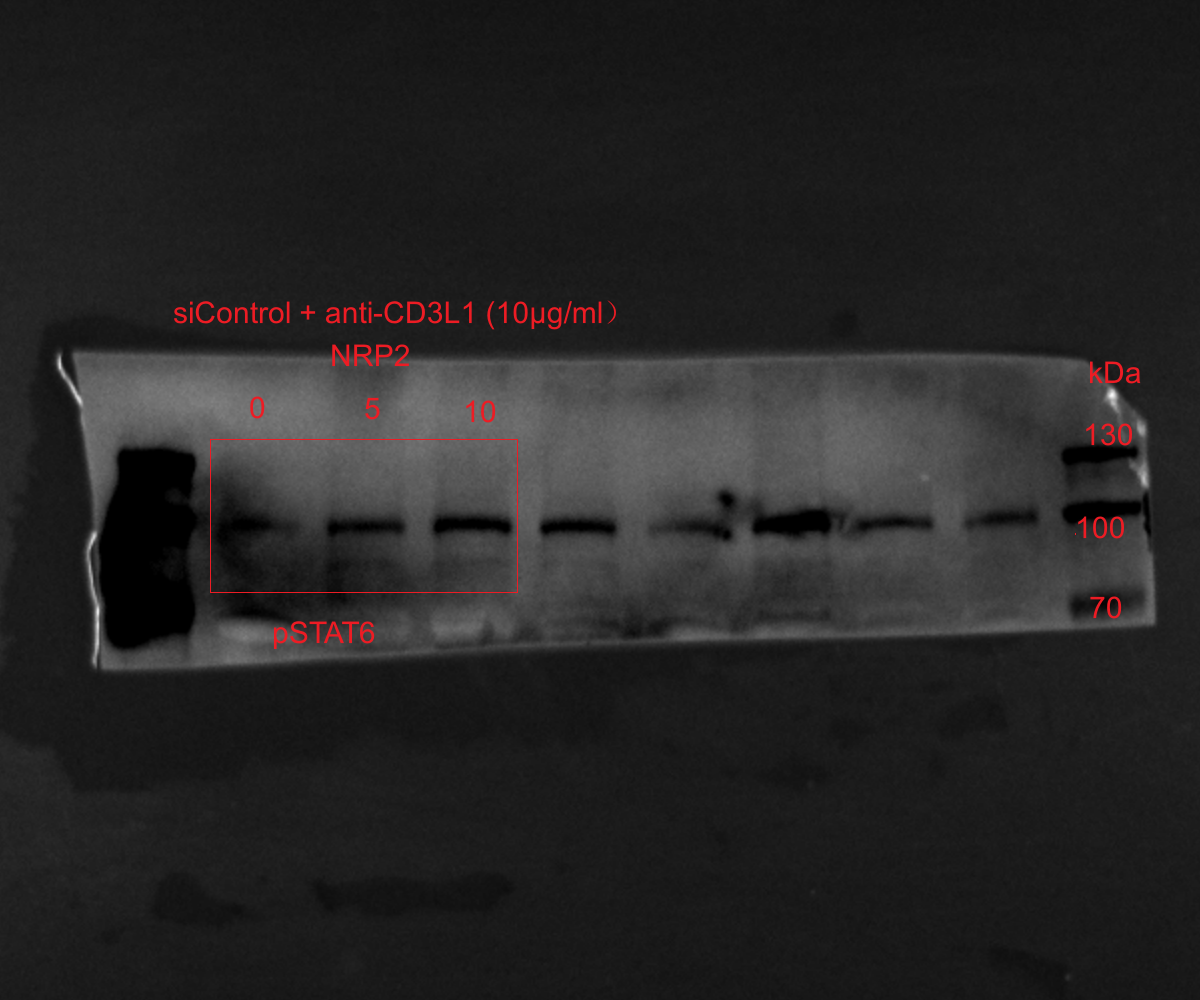

Supplement: Supplementary file 8 — Source data Fig. 5 [file 44321_2026_451_MOESM8_ESM.zip › Fig.5D/NRP2 pSTAT6.png]

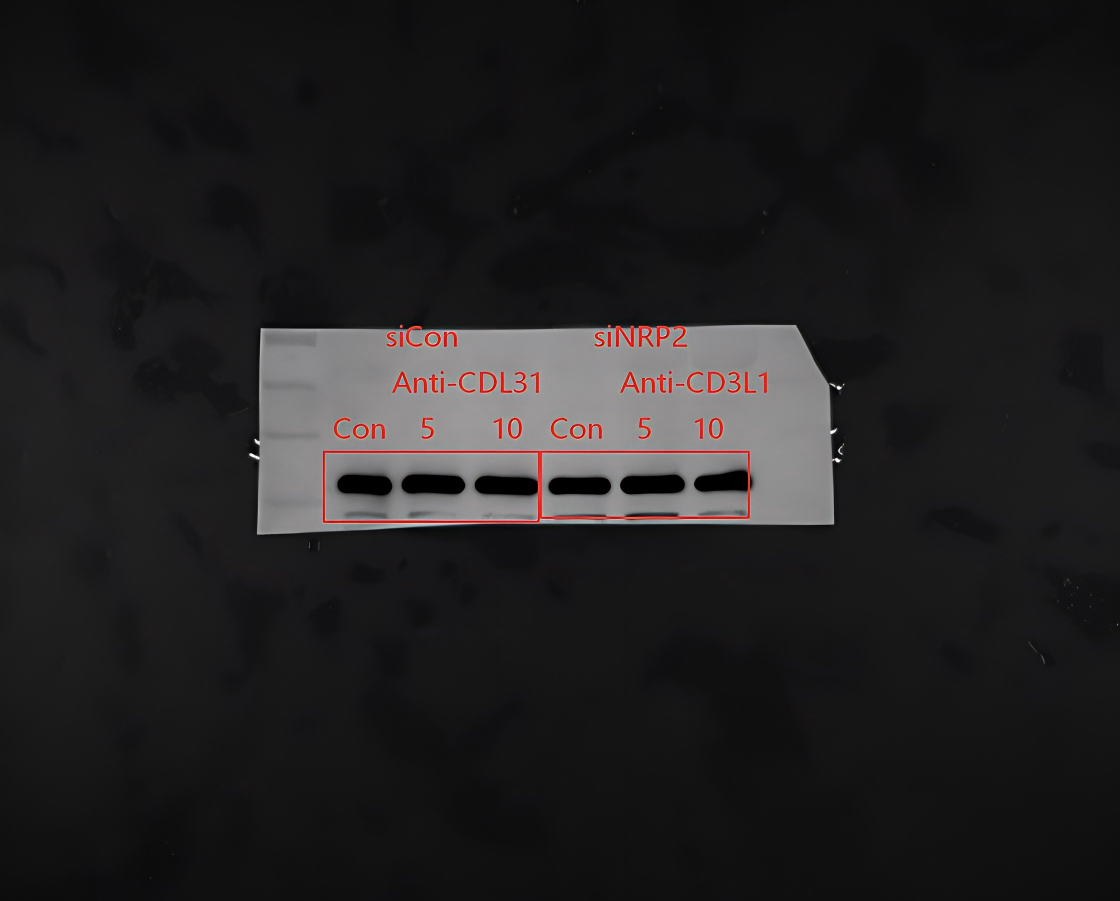

Supplement: Supplementary file 8 — Source data Fig. 5 [file 44321_2026_451_MOESM8_ESM.zip › Fig.5D/THP-1 siCon si NRP2 GAPDH Replicate 2.jpg]

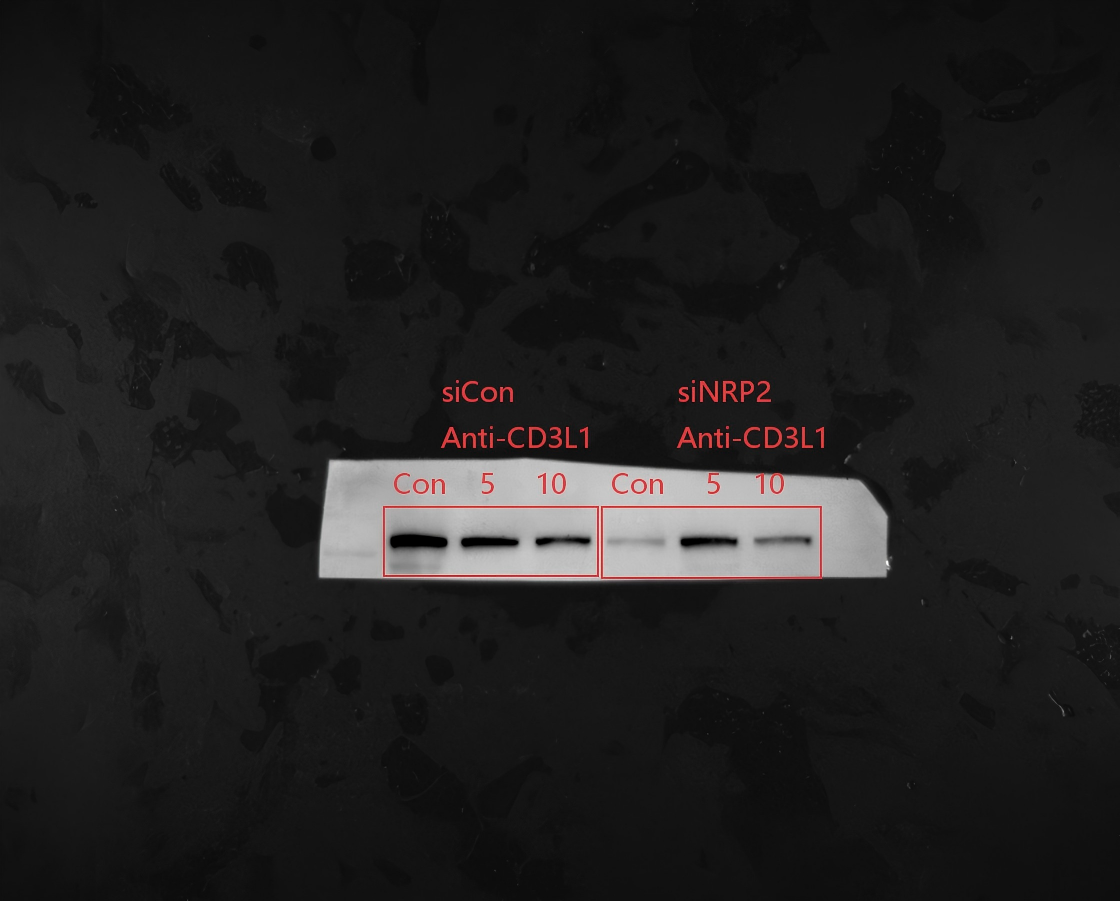

Supplement: Supplementary file 8 — Source data Fig. 5 [file 44321_2026_451_MOESM8_ESM.zip › Fig.5D/THP-1 siCon si NRP2 PSTAT6 Replicate 2.jpg]

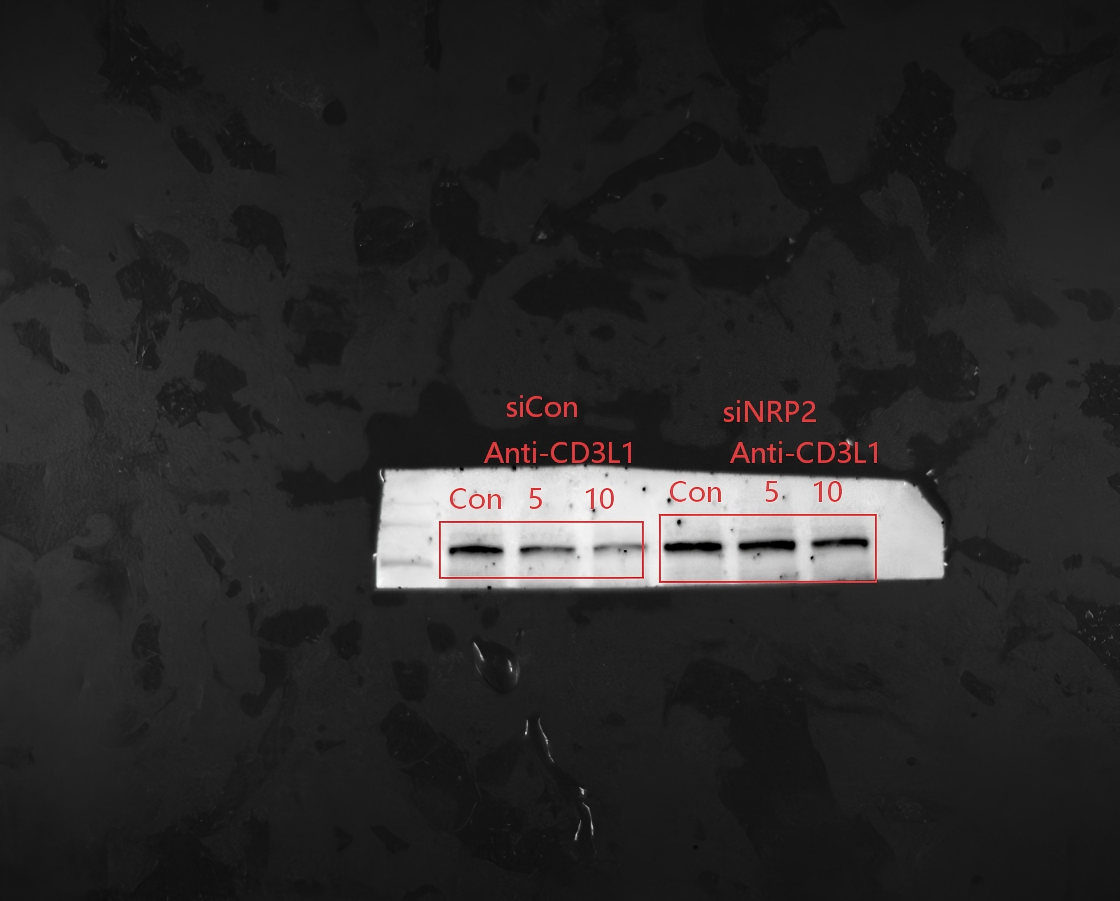

Supplement: Supplementary file 8 — Source data Fig. 5 [file 44321_2026_451_MOESM8_ESM.zip › Fig.5D/THP-1 siCon si NRP2 STAT6 Replicate 2.jpg]

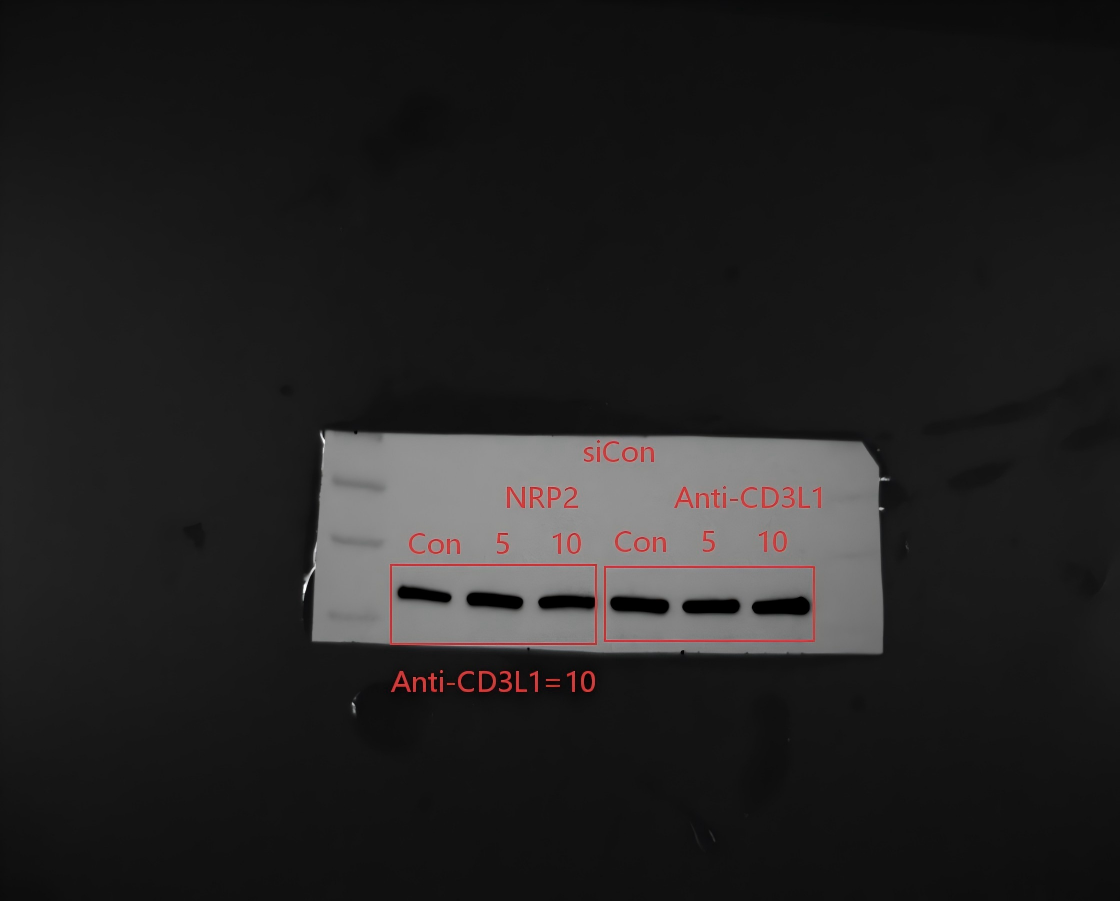

Supplement: Supplementary file 8 — Source data Fig. 5 [file 44321_2026_451_MOESM8_ESM.zip › Fig.5D/siCon + NRP2 GAPDH replicate 3.jpg]

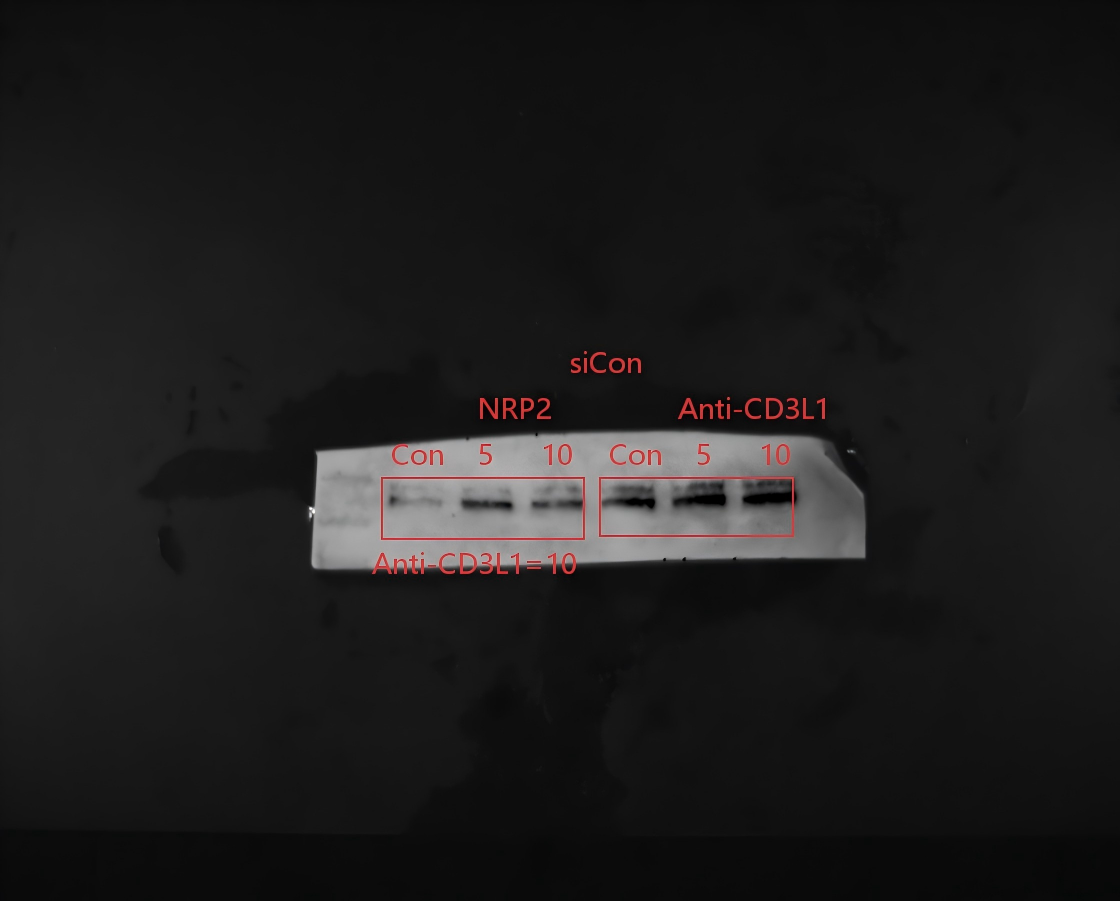

Supplement: Supplementary file 8 — Source data Fig. 5 [file 44321_2026_451_MOESM8_ESM.zip › Fig.5D/siCon + NRP2 STAT6 replicate 3.jpg]

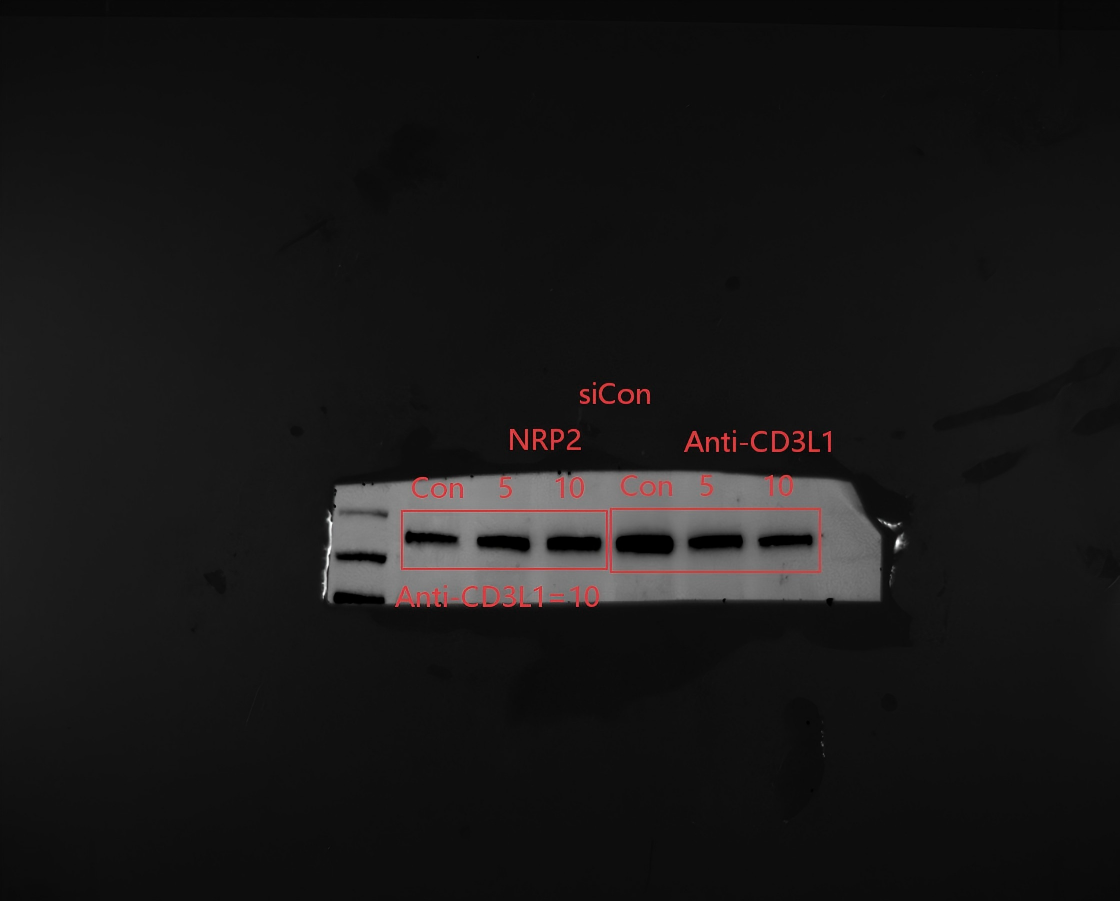

Supplement: Supplementary file 8 — Source data Fig. 5 [file 44321_2026_451_MOESM8_ESM.zip › Fig.5D/siCon + NRP2 pSTAT6 replicate 3.jpg]

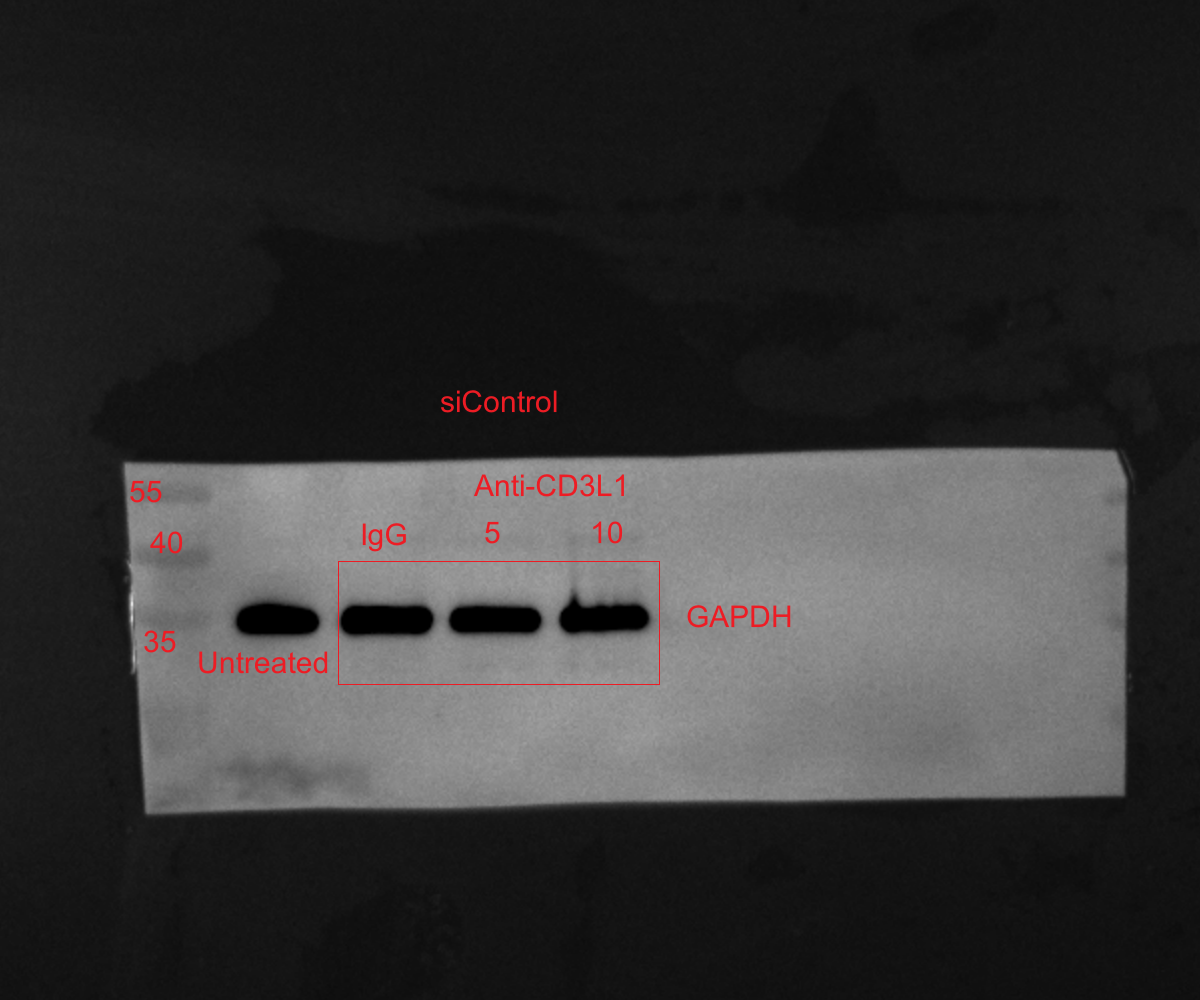

Supplement: Supplementary file 8 — Source data Fig. 5 [file 44321_2026_451_MOESM8_ESM.zip › Fig.5D/siControl GAPDH.png]

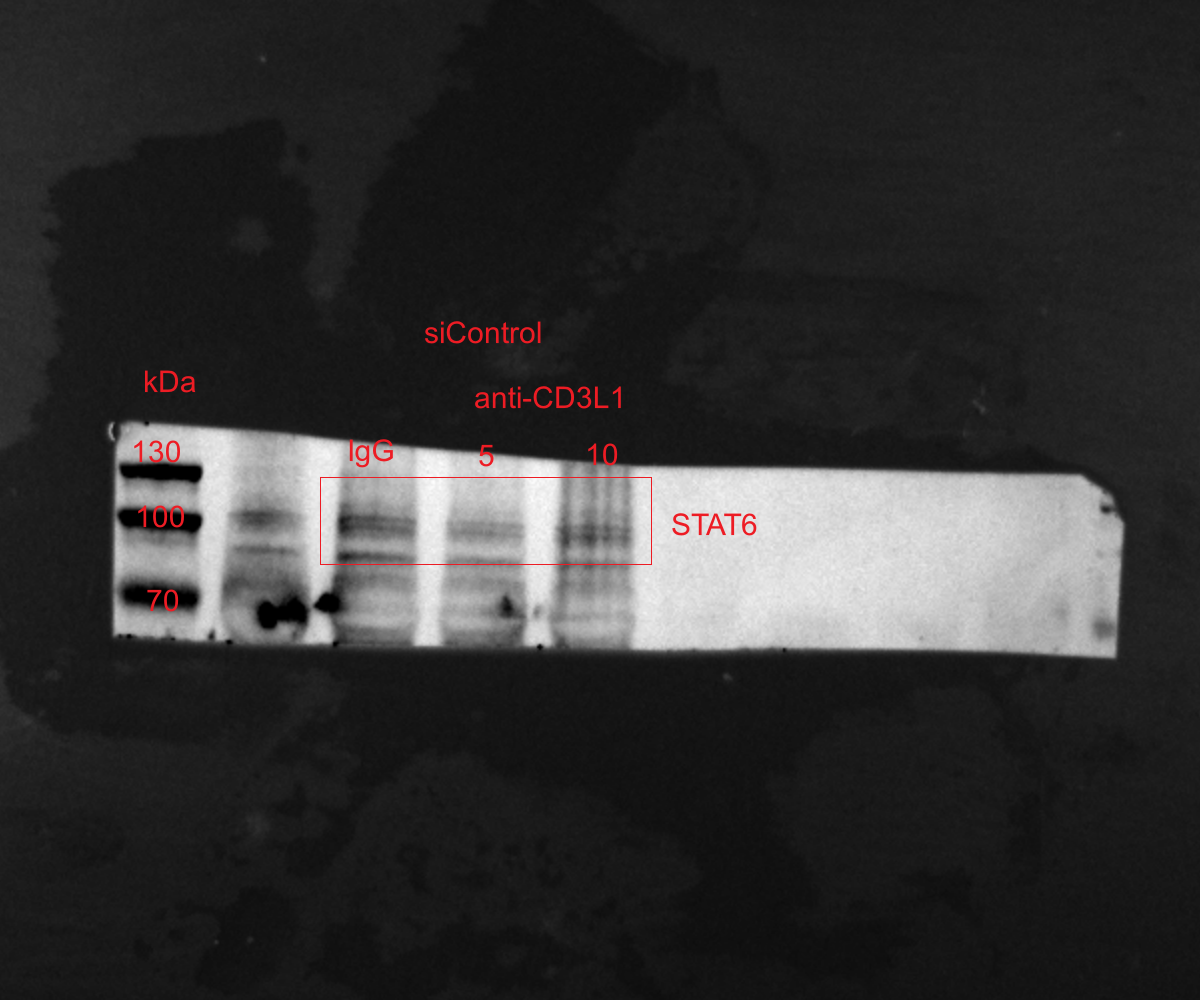

Supplement: Supplementary file 8 — Source data Fig. 5 [file 44321_2026_451_MOESM8_ESM.zip › Fig.5D/siControl STAT6.png]

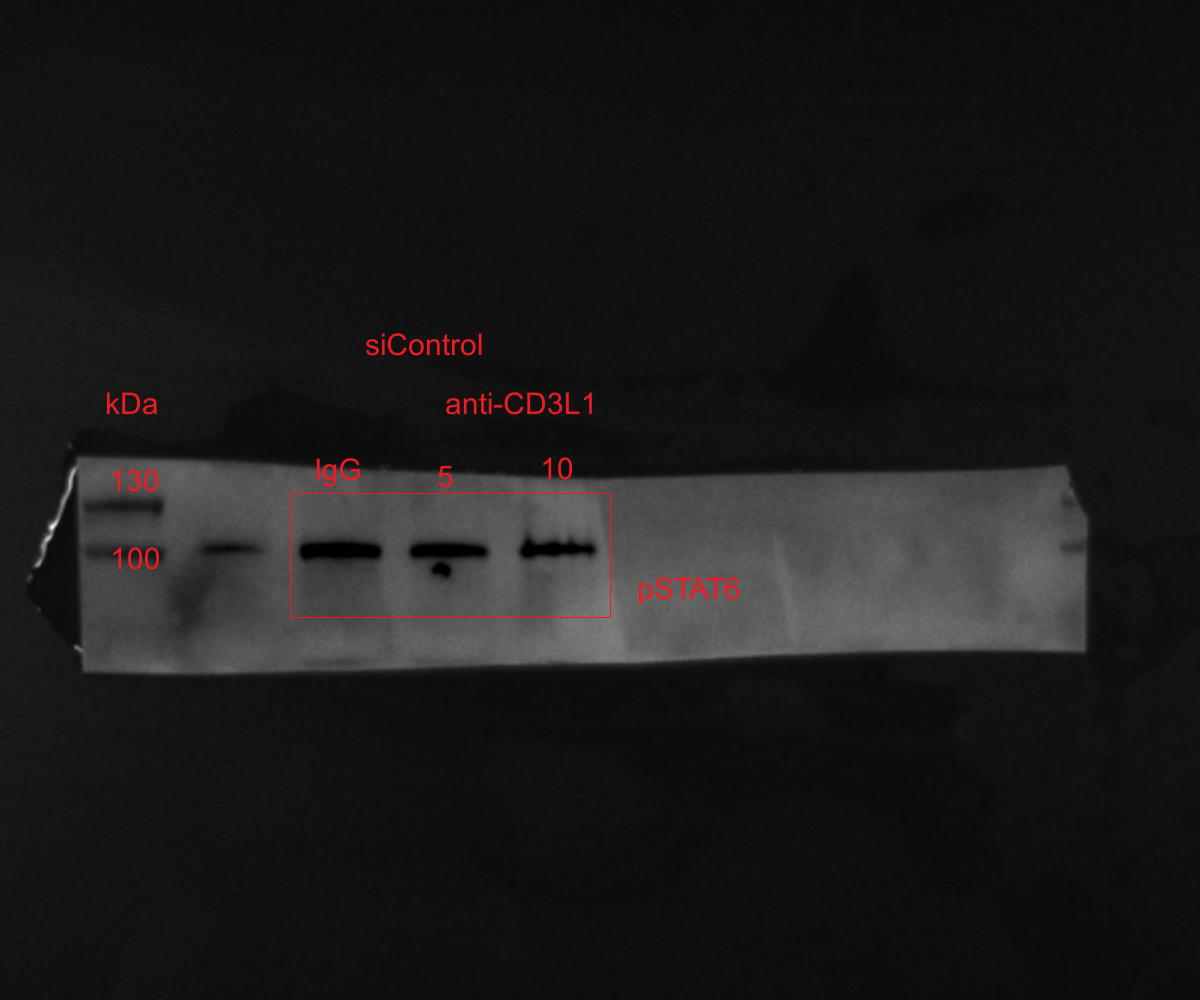

Supplement: Supplementary file 8 — Source data Fig. 5 [file 44321_2026_451_MOESM8_ESM.zip › Fig.5D/siControl pSTAT6.png]

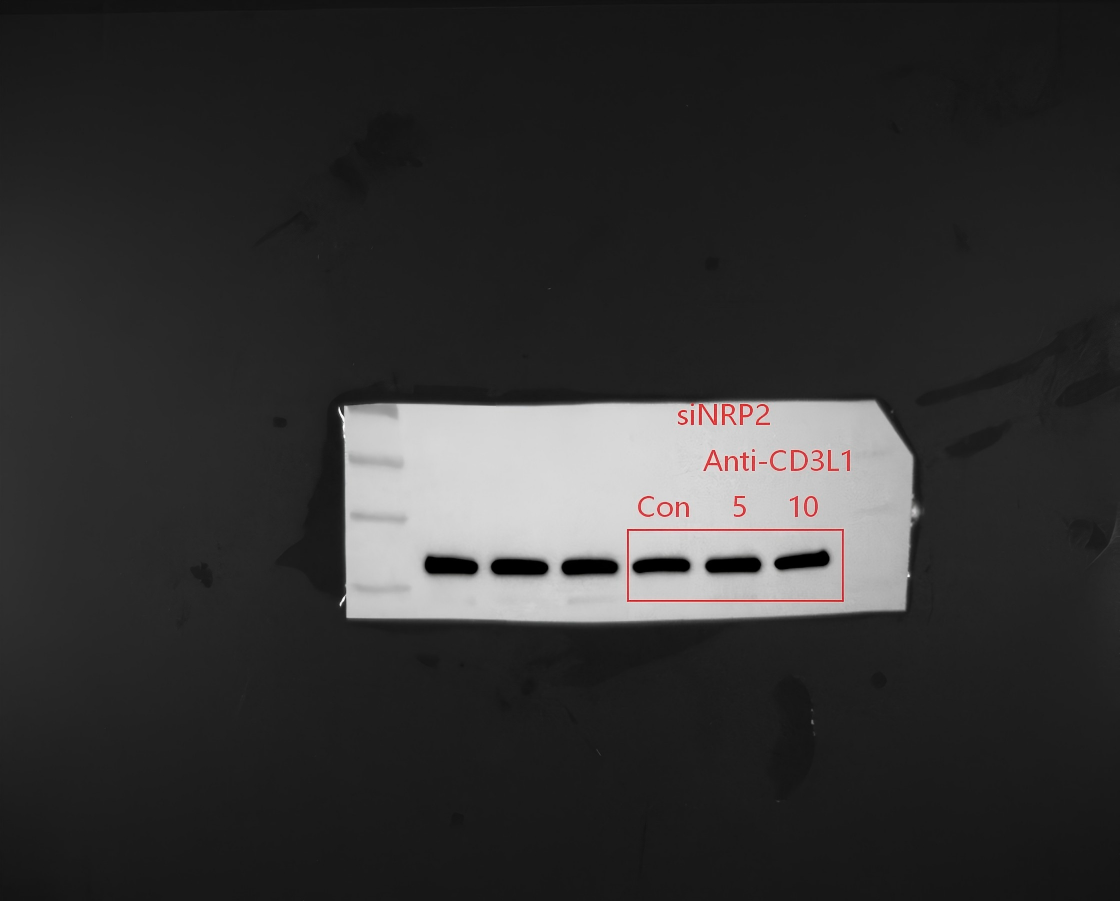

Supplement: Supplementary file 8 — Source data Fig. 5 [file 44321_2026_451_MOESM8_ESM.zip › Fig.5D/siNRP2 GAPDH replicate 3.jpg]

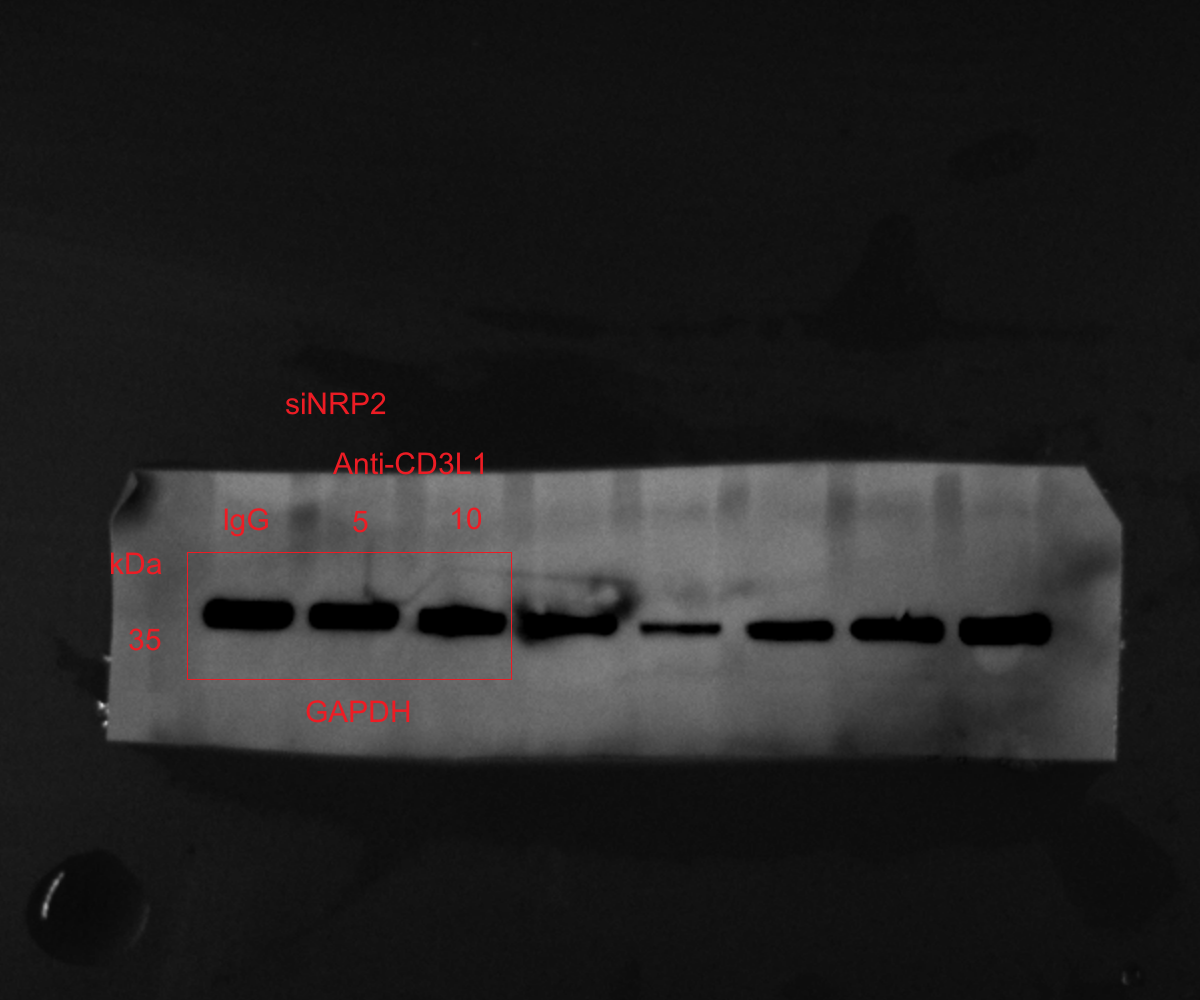

Supplement: Supplementary file 8 — Source data Fig. 5 [file 44321_2026_451_MOESM8_ESM.zip › Fig.5D/siNRP2 GAPDH.png]

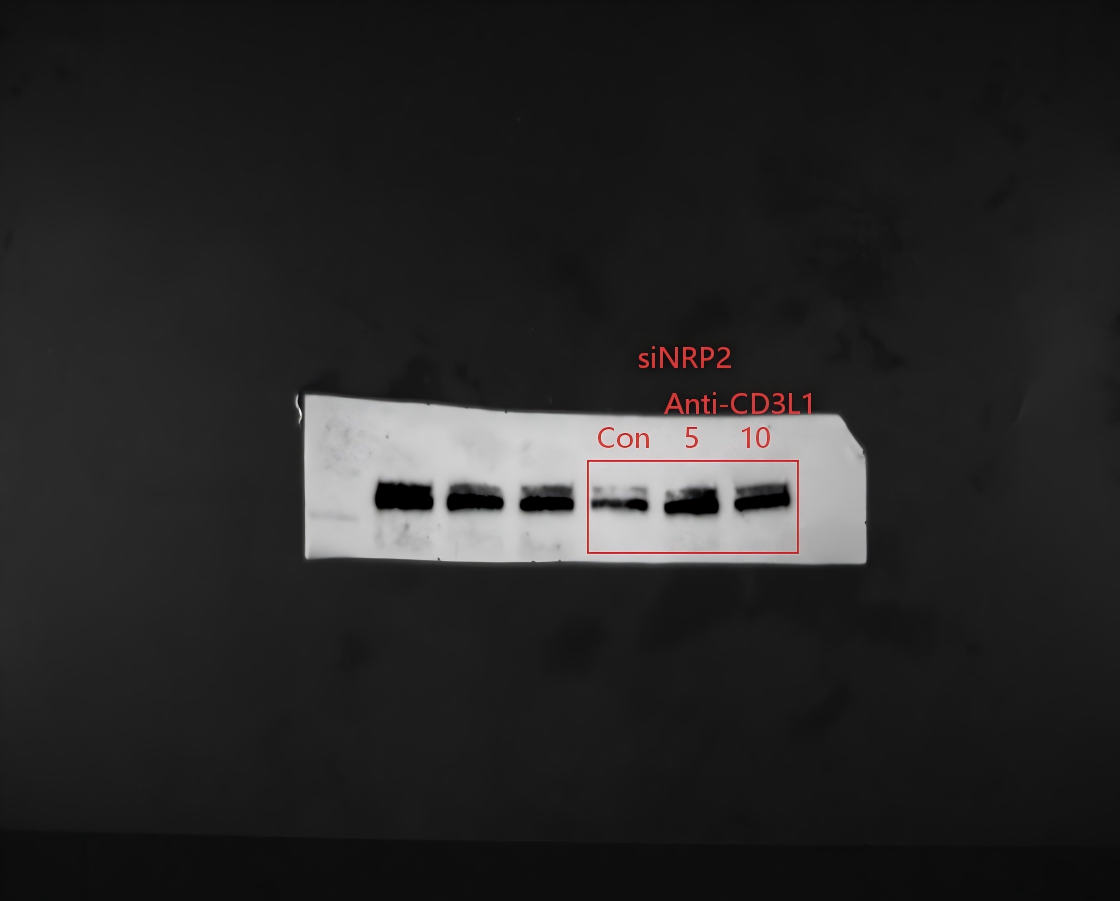

Supplement: Supplementary file 8 — Source data Fig. 5 [file 44321_2026_451_MOESM8_ESM.zip › Fig.5D/siNRP2 STAT6 replicate 3.jpg]

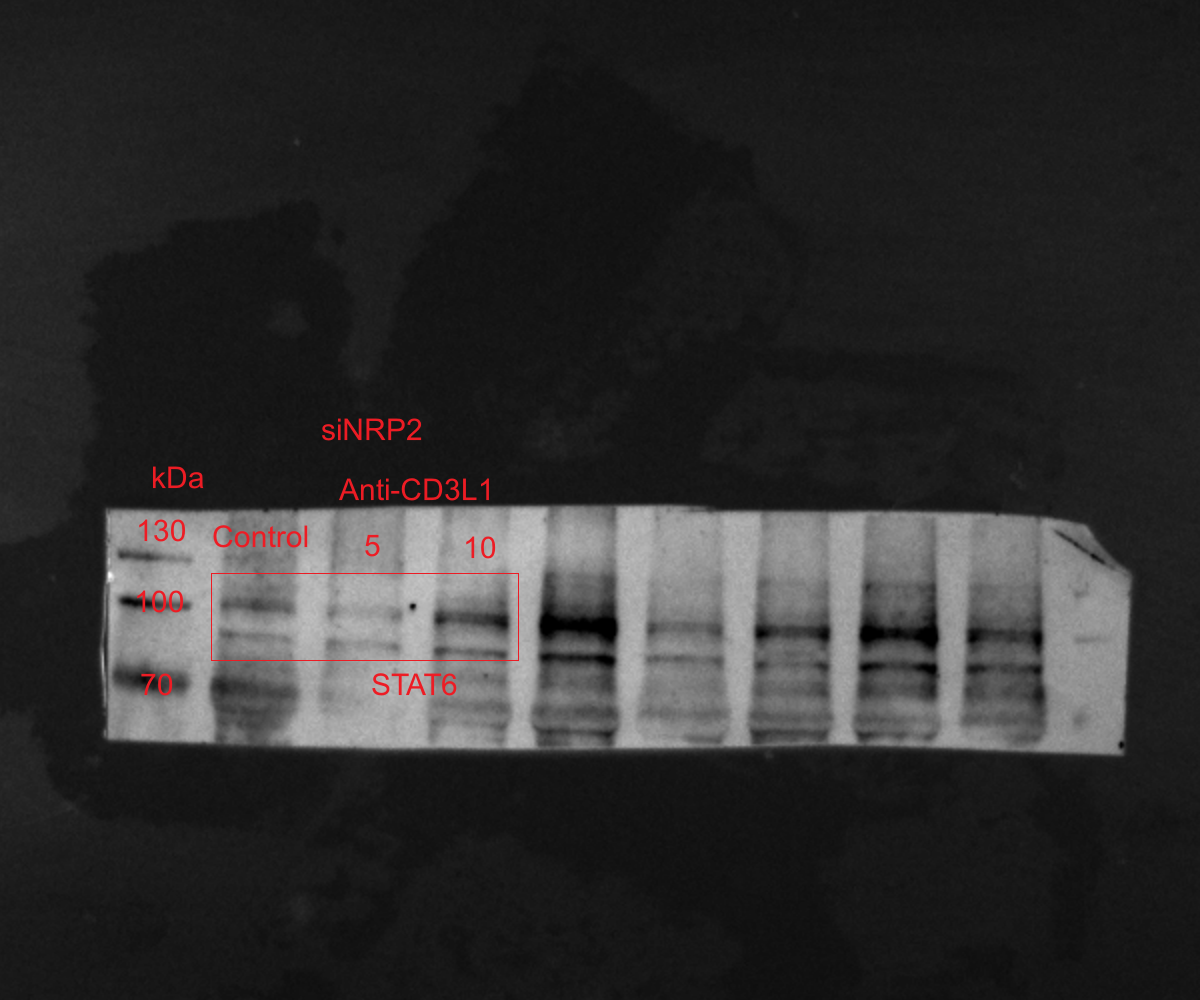

Supplement: Supplementary file 8 — Source data Fig. 5 [file 44321_2026_451_MOESM8_ESM.zip › Fig.5D/siNRP2 STAT6.png]

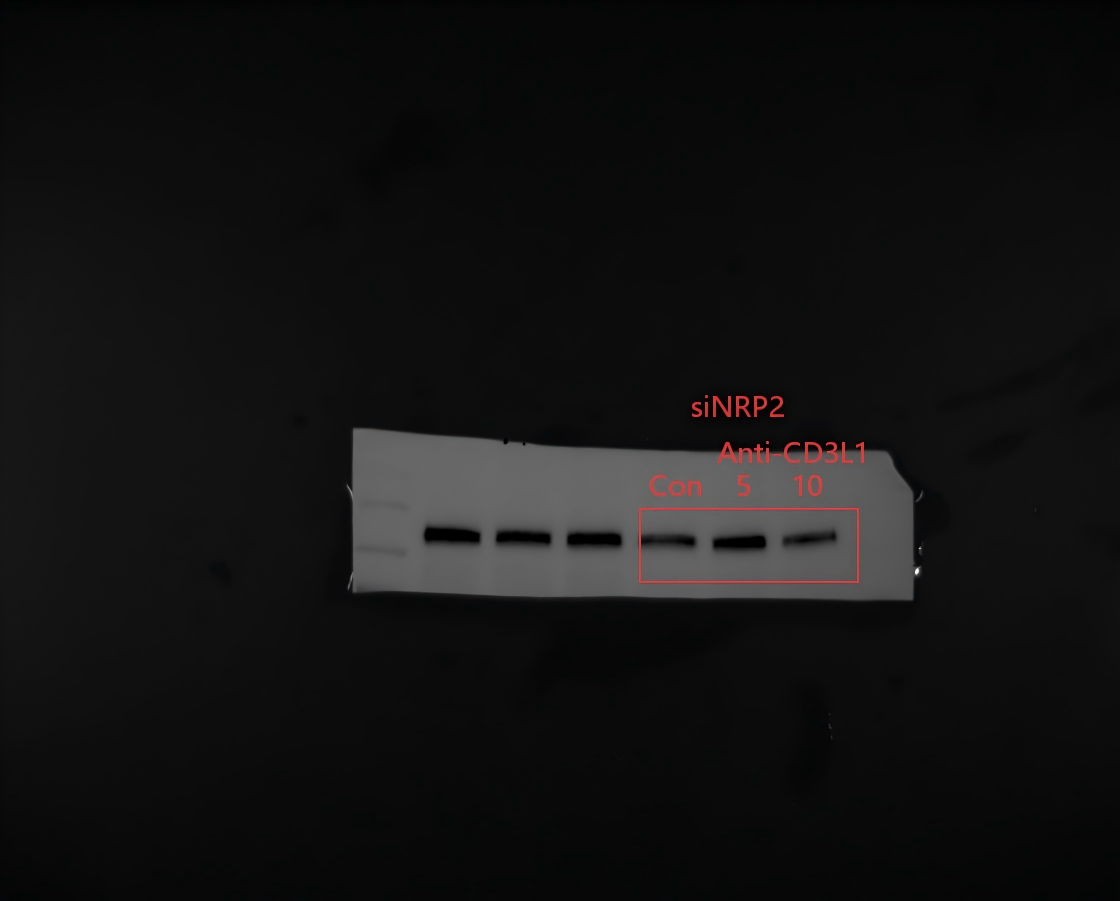

Supplement: Supplementary file 8 — Source data Fig. 5 [file 44321_2026_451_MOESM8_ESM.zip › Fig.5D/siNRP2 pSTAT6 replicate 3.jpg]

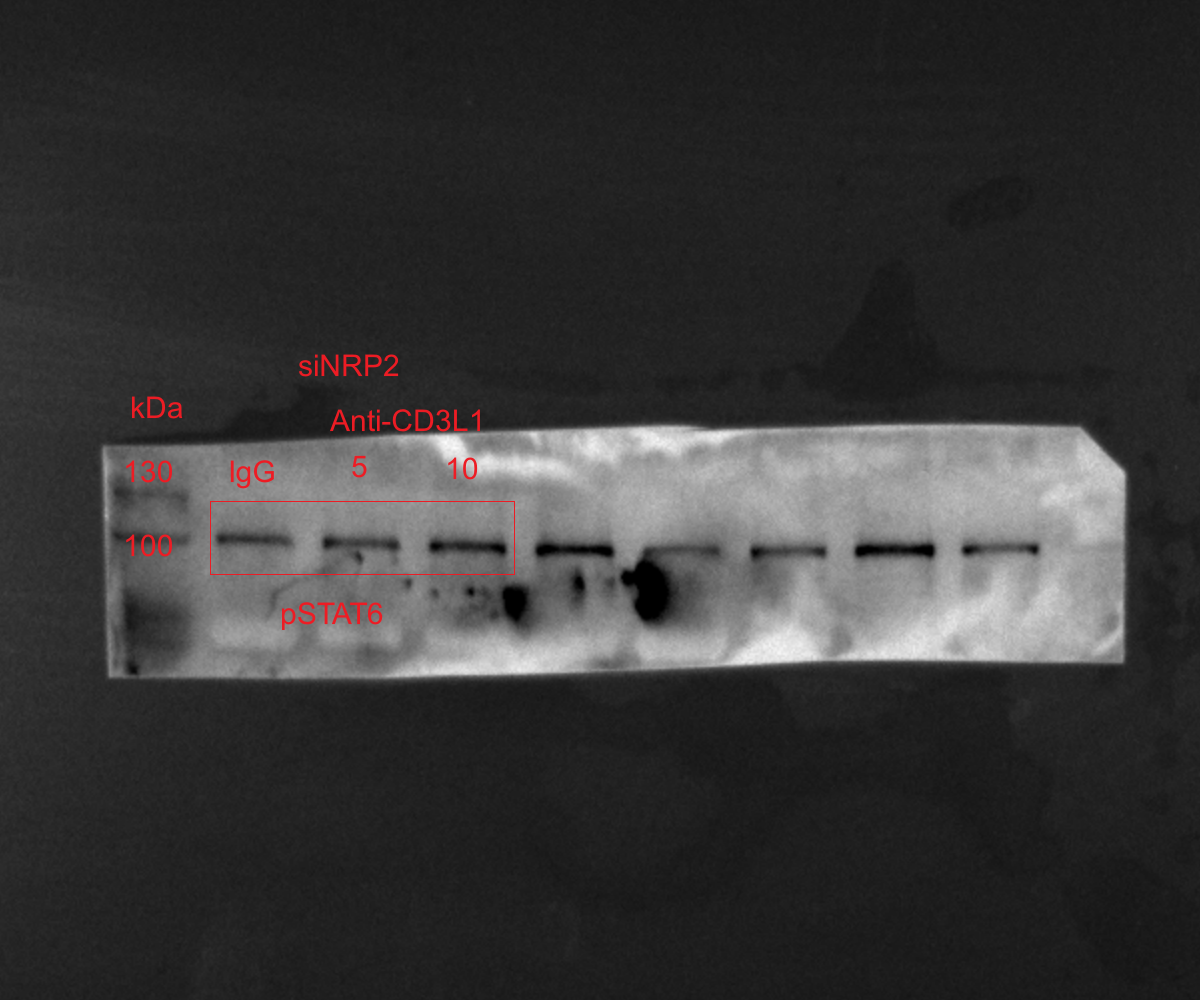

Supplement: Supplementary file 8 — Source data Fig. 5 [file 44321_2026_451_MOESM8_ESM.zip › Fig.5D/siNRP2 pSTAT6.png]

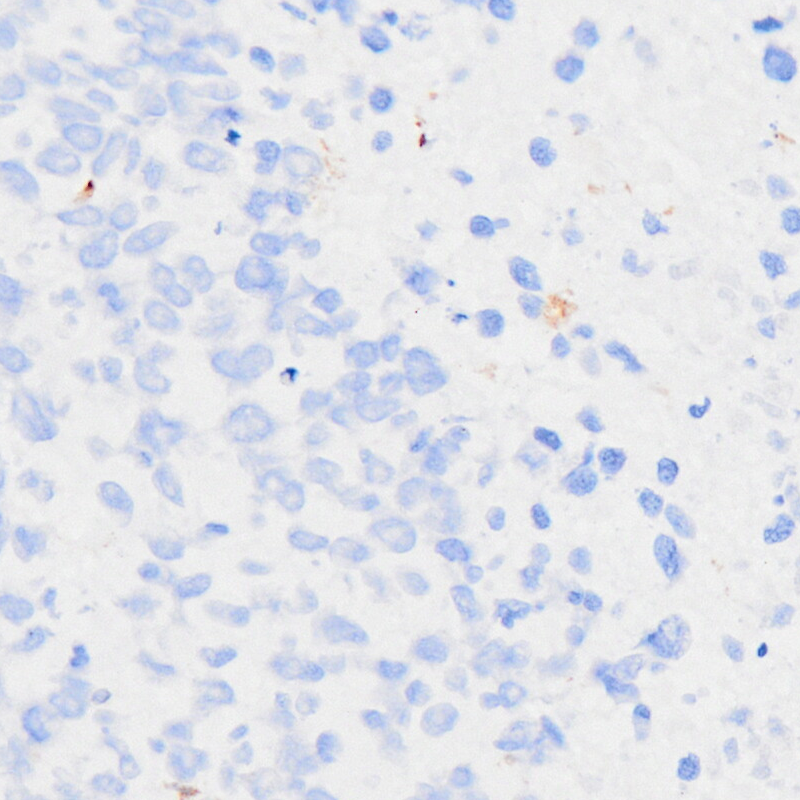

Supplement: Supplementary file 9 — Source data Fig. 6 [file 44321_2026_451_MOESM9_ESM.zip › Fig.6E/143B anti-CD3L1 CD206 zoom.png]

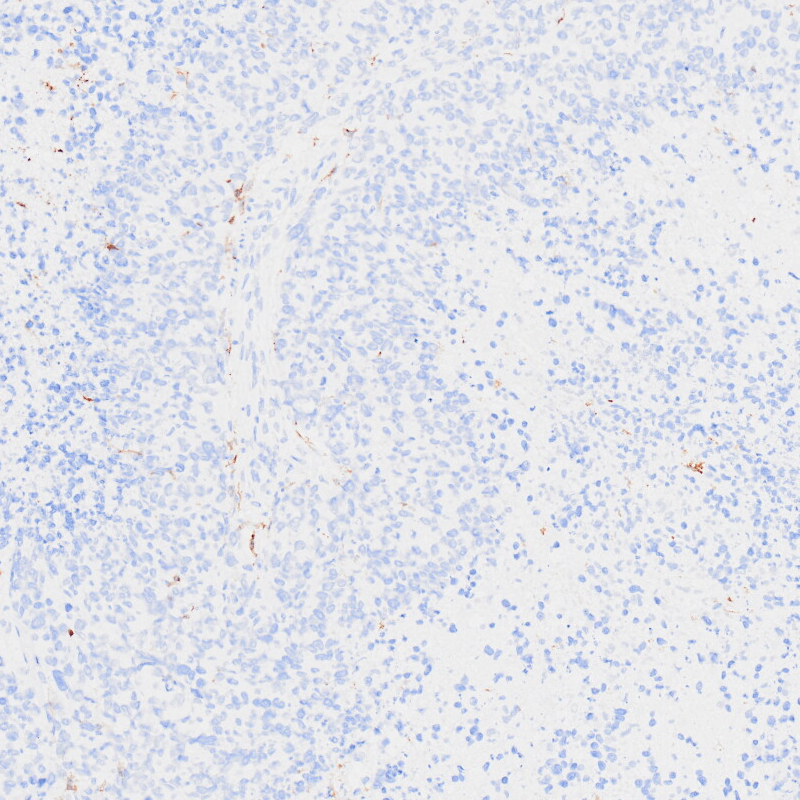

Supplement: Supplementary file 9 — Source data Fig. 6 [file 44321_2026_451_MOESM9_ESM.zip › Fig.6E/143B anti-CD3L1 CD206.png]

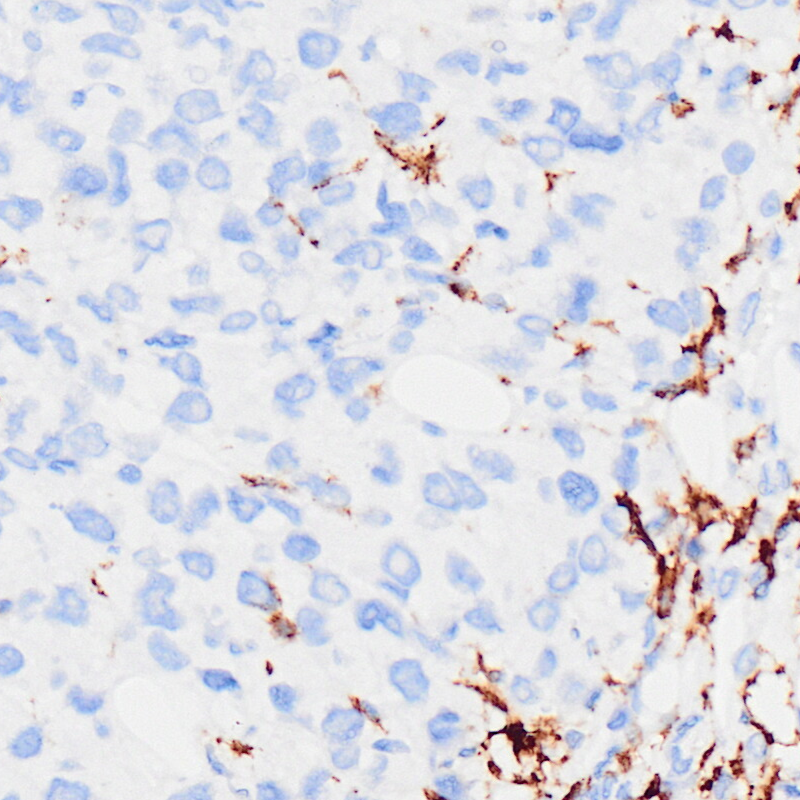

Supplement: Supplementary file 9 — Source data Fig. 6 [file 44321_2026_451_MOESM9_ESM.zip › Fig.6E/143B IgG CD206 zoom.png]

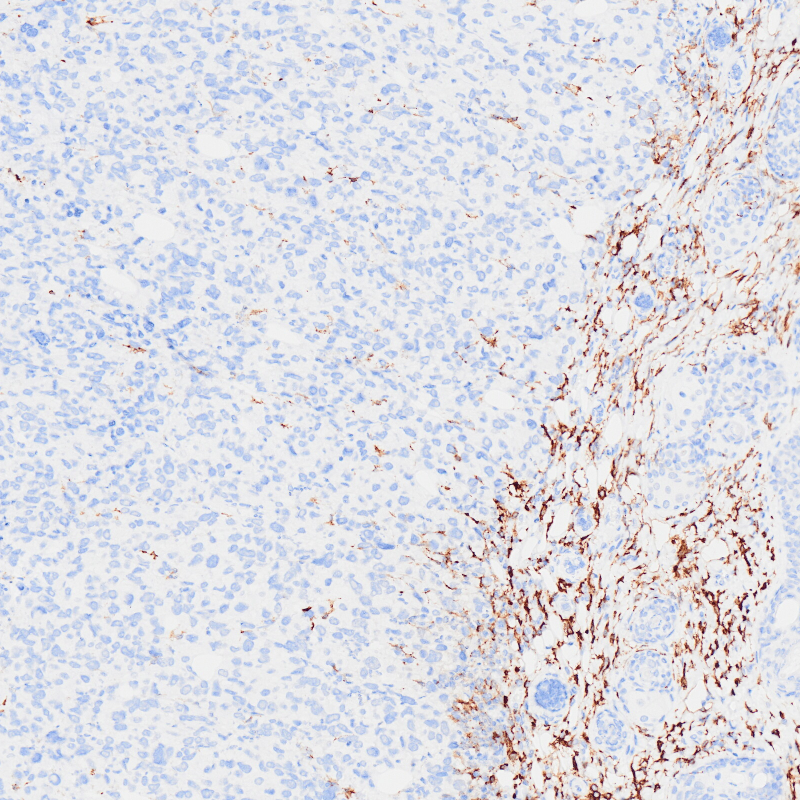

Supplement: Supplementary file 9 — Source data Fig. 6 [file 44321_2026_451_MOESM9_ESM.zip › Fig.6E/143B IgG CD206.png]

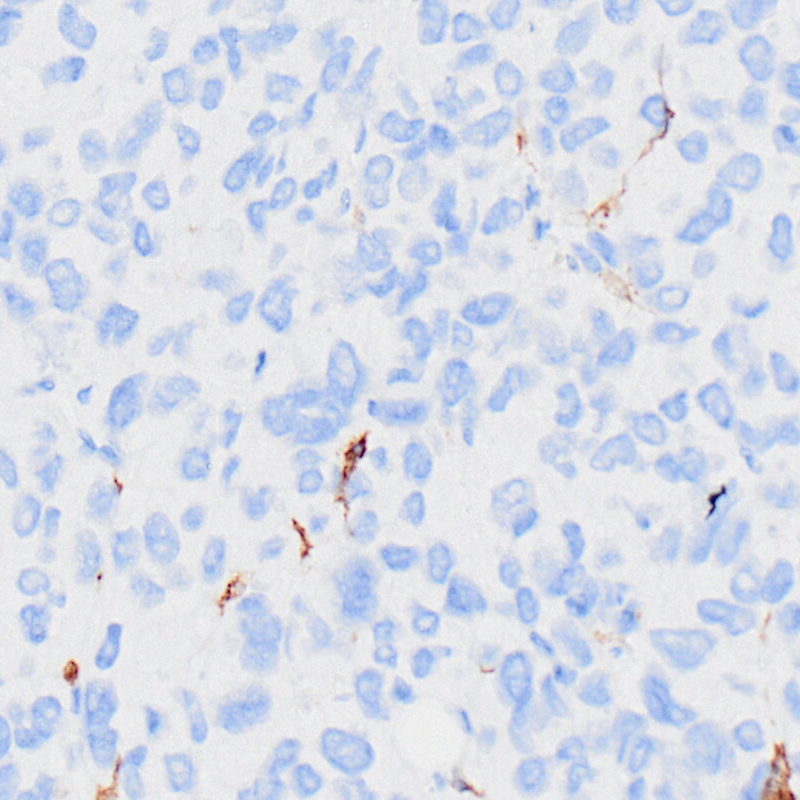

Supplement: Supplementary file 9 — Source data Fig. 6 [file 44321_2026_451_MOESM9_ESM.zip › Fig.6E/KHOS anti-CD3L1 CD206 zoom.png]

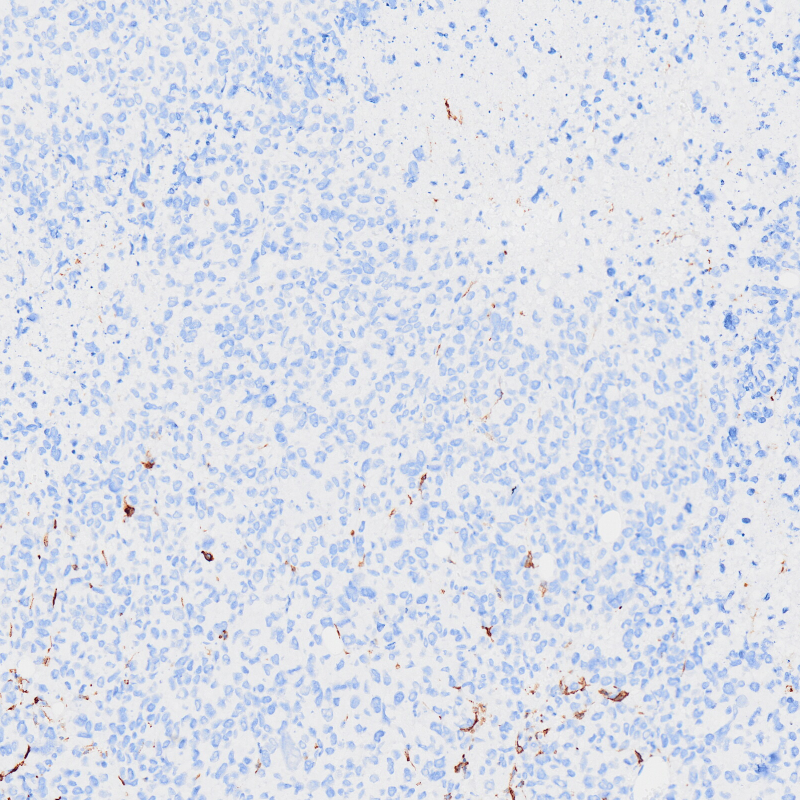

Supplement: Supplementary file 9 — Source data Fig. 6 [file 44321_2026_451_MOESM9_ESM.zip › Fig.6E/KHOS anti-CD3L1 CD206.png]

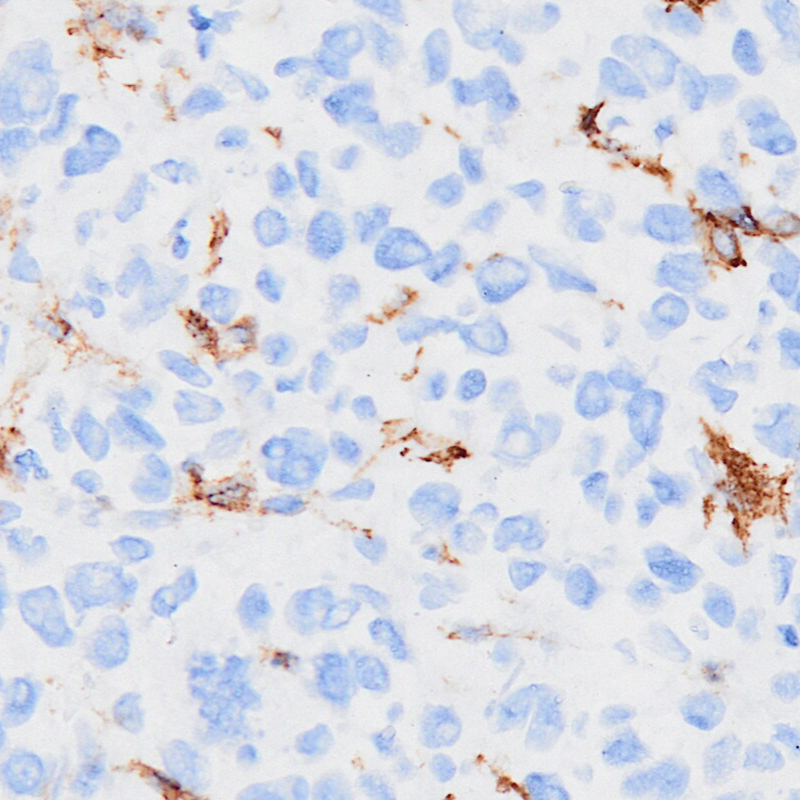

Supplement: Supplementary file 9 — Source data Fig. 6 [file 44321_2026_451_MOESM9_ESM.zip › Fig.6E/KHOS IgG CD206 zoom.png]

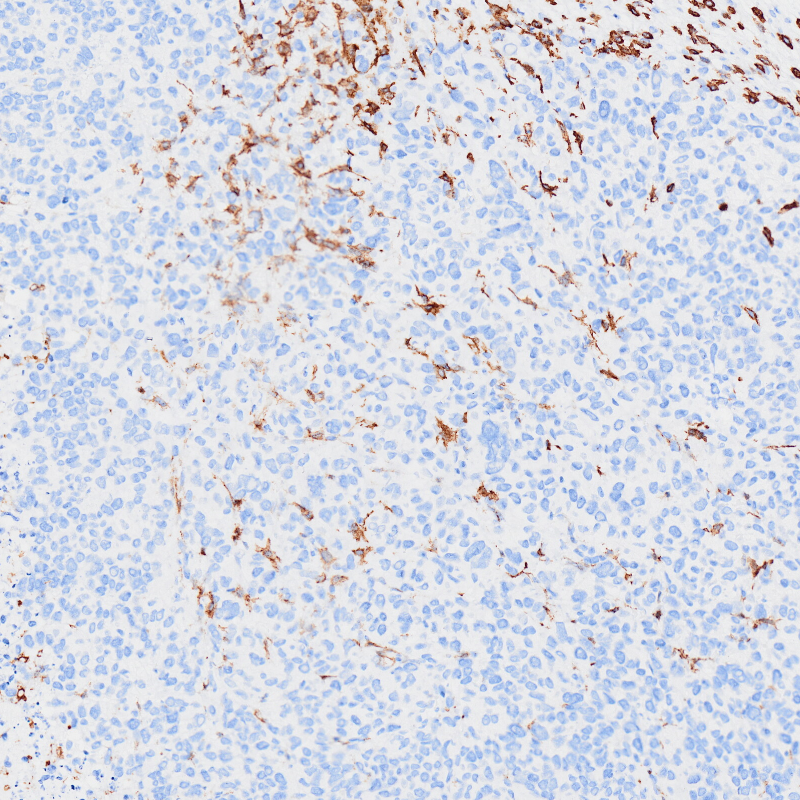

Supplement: Supplementary file 9 — Source data Fig. 6 [file 44321_2026_451_MOESM9_ESM.zip › Fig.6E/KHOS IgG CD206.png]

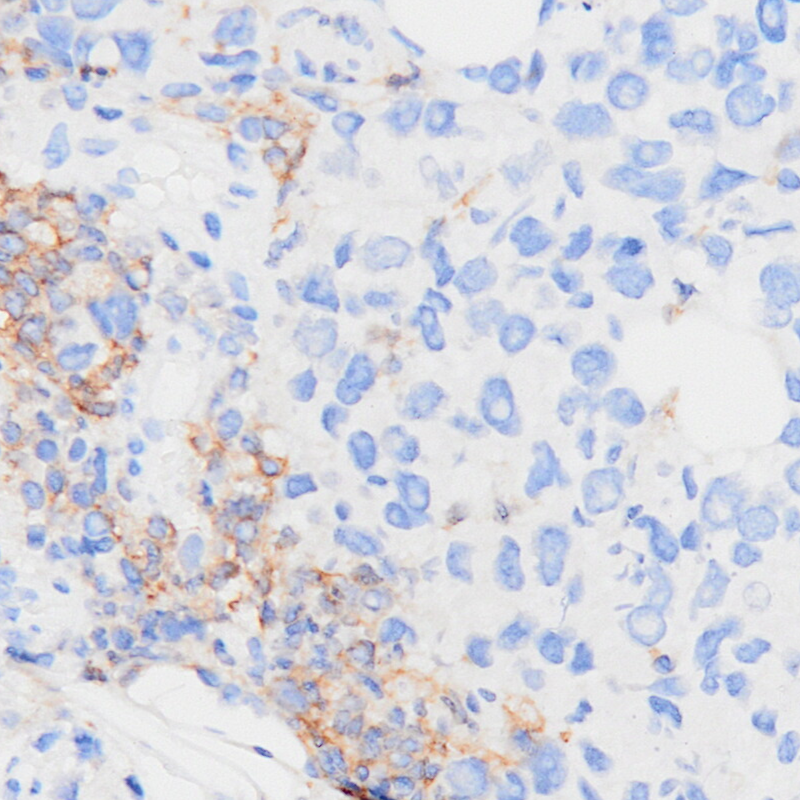

Supplement: Supplementary file 9 — Source data Fig. 6 [file 44321_2026_451_MOESM9_ESM.zip › Fig.6F/143B anti-CD3L1 CD86 zoom.png]

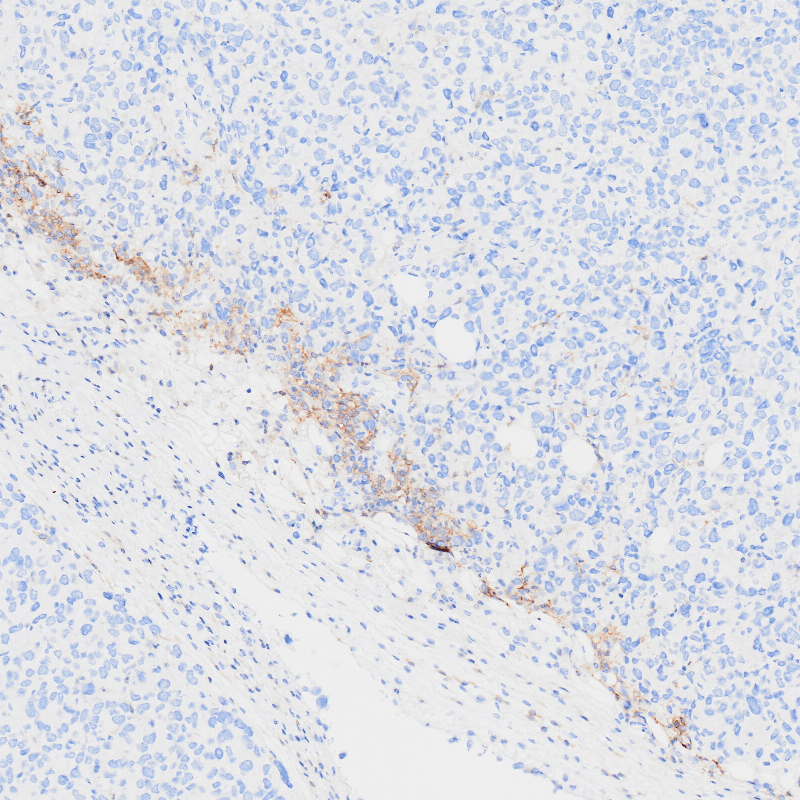

Supplement: Supplementary file 9 — Source data Fig. 6 [file 44321_2026_451_MOESM9_ESM.zip › Fig.6F/143B anti-CD3L1 CD86.png]

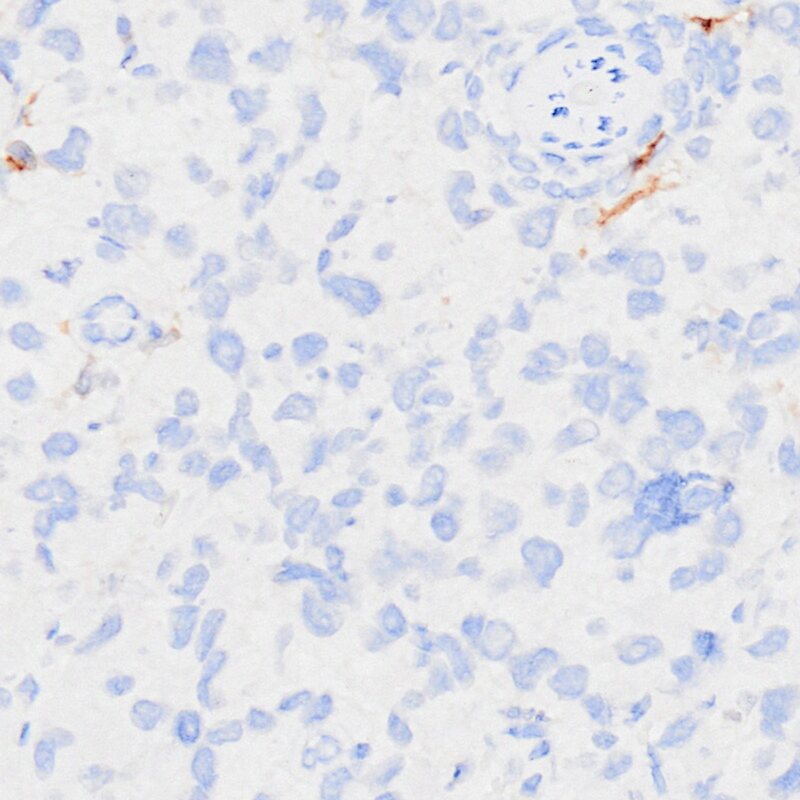

Supplement: Supplementary file 9 — Source data Fig. 6 [file 44321_2026_451_MOESM9_ESM.zip › Fig.6F/143B IgG CD86 zoom.png]

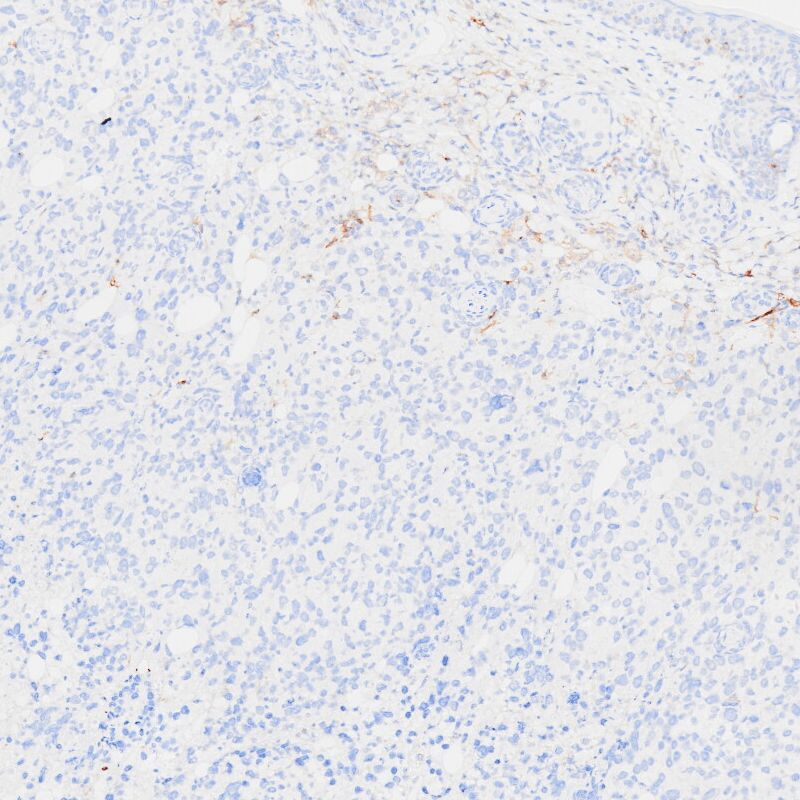

Supplement: Supplementary file 9 — Source data Fig. 6 [file 44321_2026_451_MOESM9_ESM.zip › Fig.6F/143B IgG CD86.png]

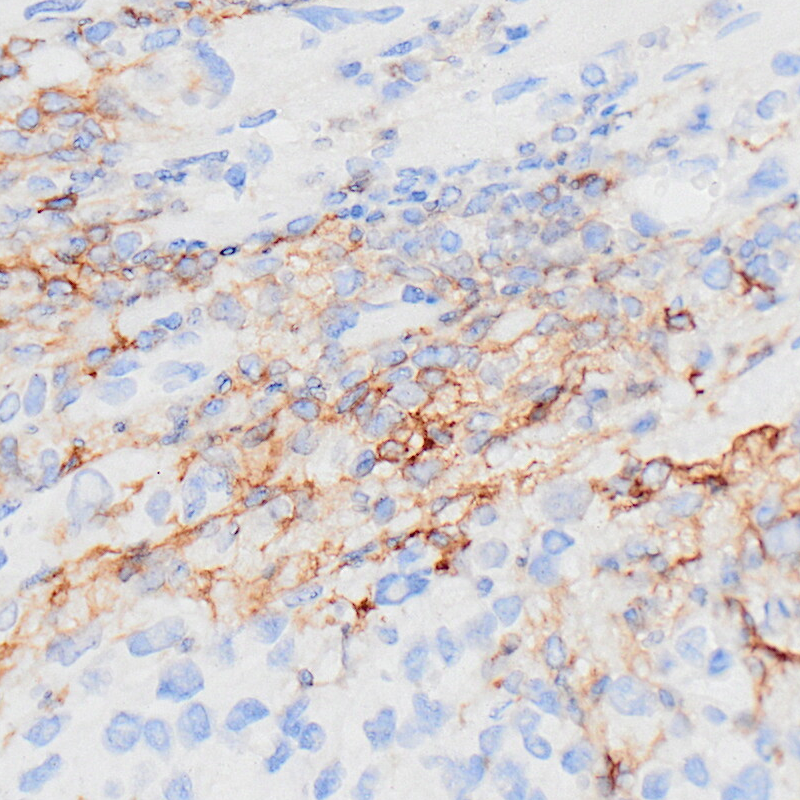

Supplement: Supplementary file 9 — Source data Fig. 6 [file 44321_2026_451_MOESM9_ESM.zip › Fig.6F/KHOS anti-CD3L1 CD86 zoom.png]

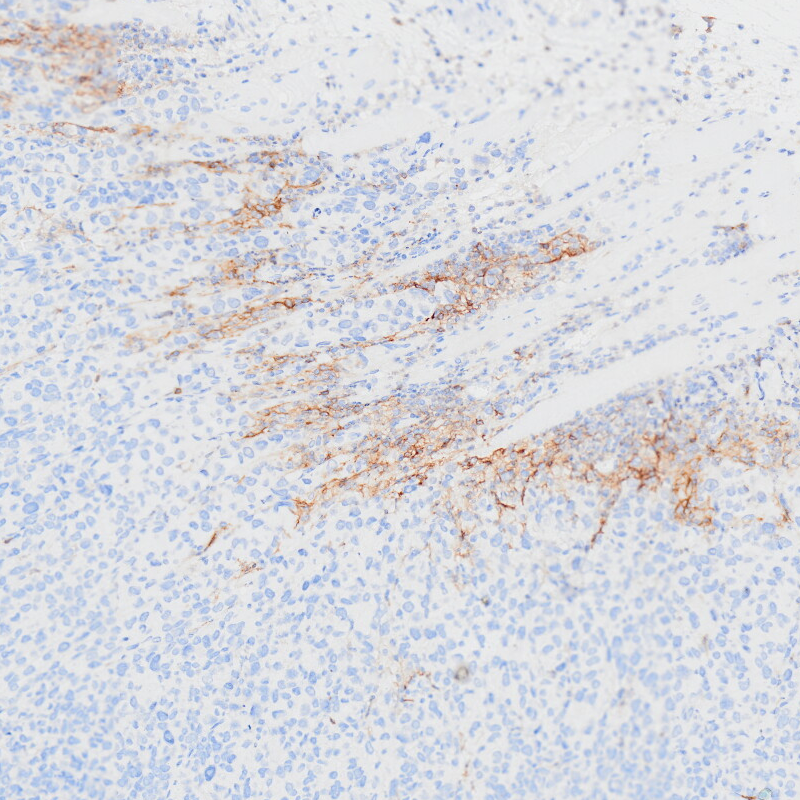

Supplement: Supplementary file 9 — Source data Fig. 6 [file 44321_2026_451_MOESM9_ESM.zip › Fig.6F/KHOS anti-CD3L1 CD86.png]

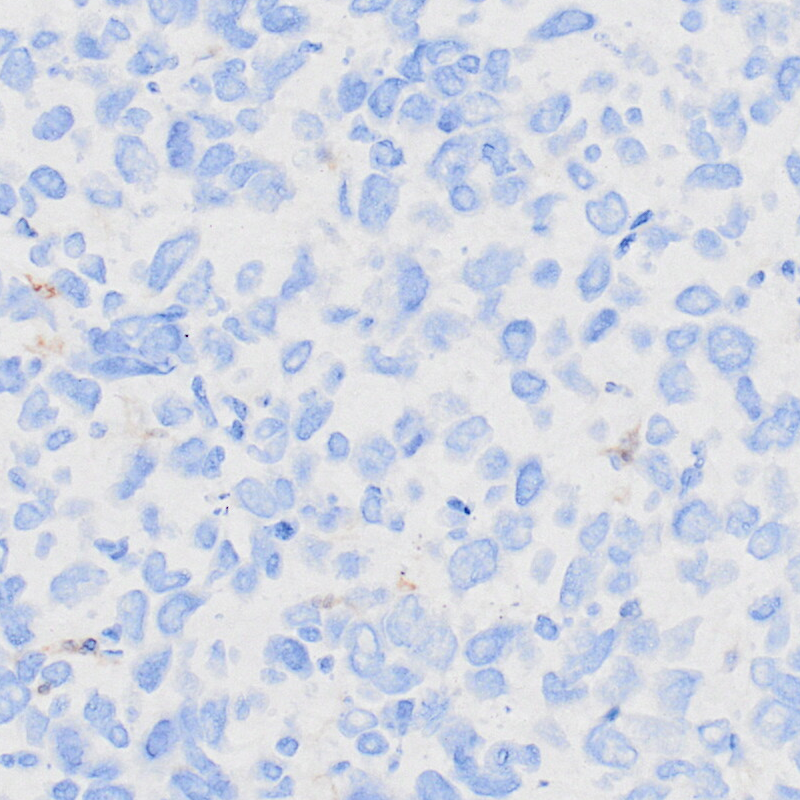

Supplement: Supplementary file 9 — Source data Fig. 6 [file 44321_2026_451_MOESM9_ESM.zip › Fig.6F/KHOS IgG CD86 zoom.png]

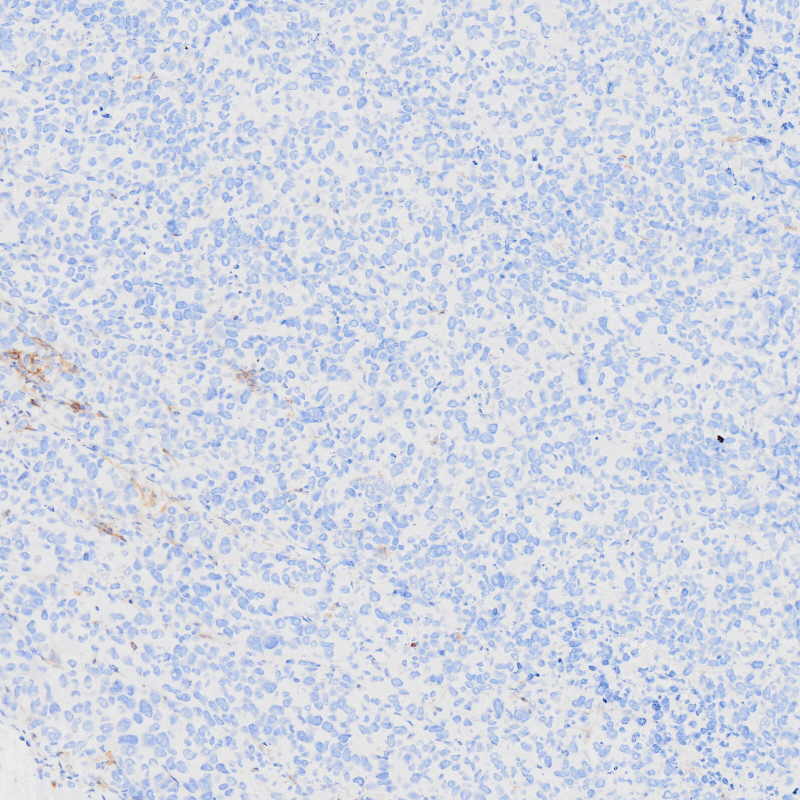

Supplement: Supplementary file 9 — Source data Fig. 6 [file 44321_2026_451_MOESM9_ESM.zip › Fig.6F/KHOS IgG CD86.png]
