# Supplementary material for: Targeting CD3L1-NRP2 disarms myeloid-driven tumor immune evasion
Source: EMBO Mol Med. 2026 May 15;18(7):2635–66. doi: 10.1038/s44321-026-00451-3 (PMC13365830; doi:10.1038/s44321-026-00451-3)
Supplement: Supplementary file 10 — Expanded View Figures [file 44321_2026_451_MOESM10_ESM.pdf]

Expanded View Figures

Figure EV1. Databases showing the negative correlation between CD3L1 and clinical prognosis or M2 TAM infiltration in patients with different advanced solid tumors, related to Fig. 1.

(**A–C**) Databases from GSE31210 and TCGA show the negative correlation between CD3L1 expression and survival rate in glioma, lung adenocarcinoma, and renal papillary cancer patients. (**D–I**) Databases from TIMER show the significant positive correlation between CD3L1 expression and M2 macrophage infiltration level in different cancers ((**D**) Low-grade glioma, LGG; (**E**) Kidney chromophobe, KICH; (**F**) Colon adenocarcinoma, COAD; (**G**) Pancreatic adenocarcinoma, PAAD; (**H**) Breast carcinoma-luminal B, BRCA-LumB; (**I**) Bladder cancer, BLCA) analyzed based on the TCGA database. (**J**, **K**) IHC staining of the human macrophage marker (**J**) CD11b and (**K**) M2-TAM marker CD206 in the additional 143B/KHOS osteosarcoma samples treated by 10 mg/kg control IgG or anti-CD3L1 from the under ×5 and ×20 microscopic views (scale bar = 100 μm) ($n = 3$ independent samples). (**A–C**) Kaplan–Meier survival analysis; (**D–I**) Correlation Analysis and Linear Regression Analysis (details in the methodological part).

© The Author(s)

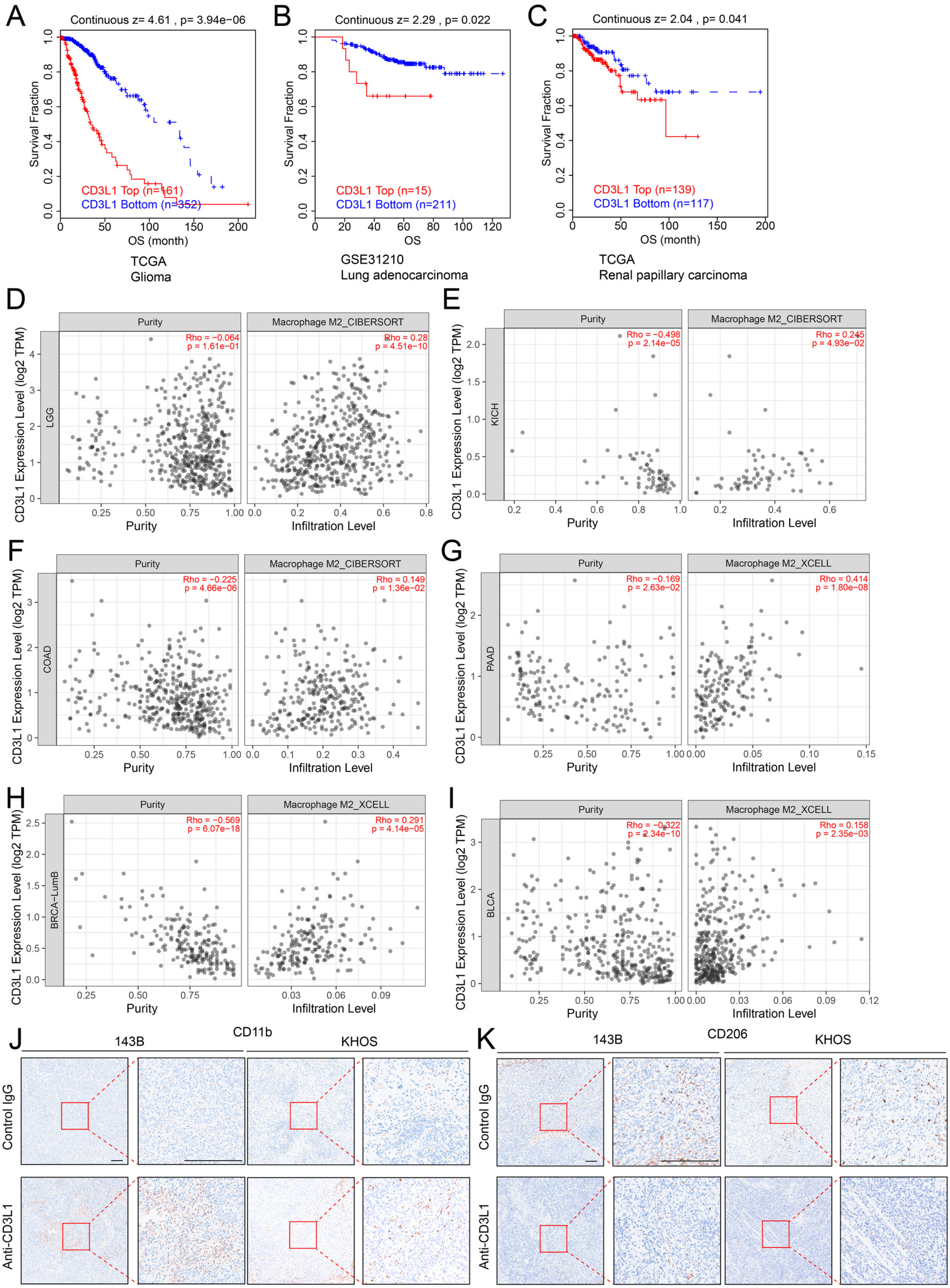
A
Continuous z= 4.61 , p= 3.94e−06
CD3L1 Top (n=161)
CD3L1 Bottom (n=352)
Survival Fraction
OS (month)
TCGA
Glioma
B
Continuous z= 2.29 , p= 0.022
CD3L1 Top (n=15)
CD3L1 Bottom (n=211)
OS
GSE31210
Lung adenocarcinoma
C
Continuous z= 2.04 , p= 0.041
CD3L1 Top (n=139)
CD3L1 Bottom (n=117)
OS (month)
TCGA
Renal papillary carcinoma
D
Purity
Macrophage M2_CIBERSORT
Rho = −0.064
p = 1.61e−01
Rho = 0.28
p = 4.51e−10
LGG
CD3L1 Expression Level (log2 TPM)
Purity
Infiltration Level
E
Purity
Macrophage M2_CIBERSORT
Rho = −0.498
p = 2.14e−05
Rho = 0.245
p = 4.93e−02
KICH
F
Purity
Macrophage M2_CIBERSORT
Rho = −0.225
p = 4.66e−06
Rho = 0.149
p = 1.36e−02
COAD
G
Purity
Macrophage M2_XCELL
Rho = −0.169
p = 2.63e−02
Rho = 0.414
p = 1.80e−08
PAAD
H
Purity
Macrophage M2_XCELL
Rho = −0.569
p = 6.07e−18
Rho = 0.291
p = 4.14e−05
BRCA−LumB
I
Purity
Macrophage M2_XCELL
Rho = −0.322
p = 2.34e−10
Rho = 0.158
p = 2.35e−03
BLCA
J
CD11b
143B
KHOS
Control IgG
Anti-CD3L1
K
CD206
143B
KHOS
Control IgG
Anti-CD3L1

 © The Author(s)

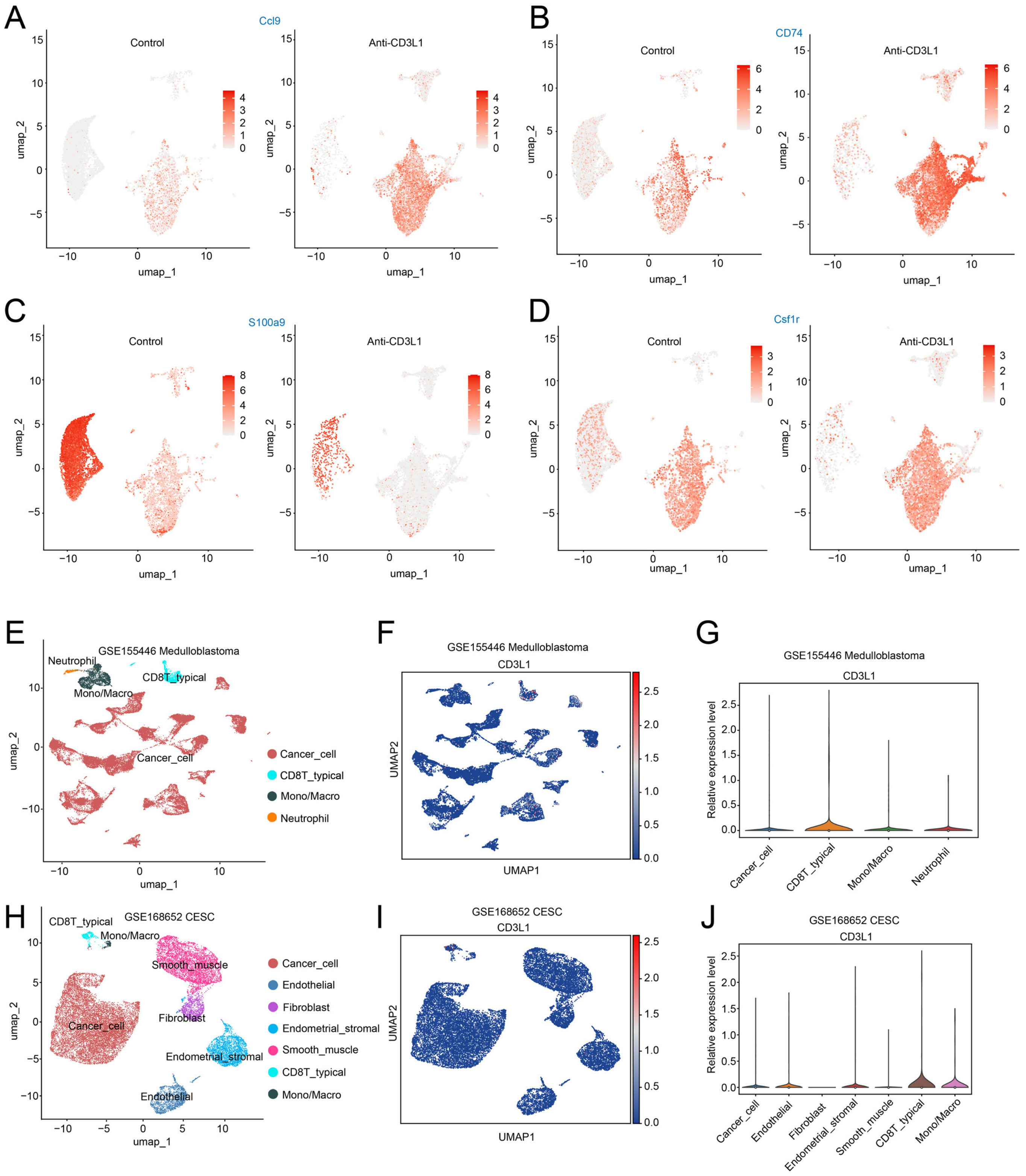
A
Ccl9
Control
Anti-CD3L1
umap_1
umap_2
B
CD74
Control
Anti-CD3L1
C
S100a9
Control
Anti-CD3L1
D
Csf1r
Control
Anti-CD3L1
E
GSE155446 Medulloblastoma
Neutrophil
CD8T_typical
Mono/Macro
Cancer_cell
F
GSE155446 Medulloblastoma
CD3L1
UMAP1
UMAP2
G
GSE155446 Medulloblastoma
CD3L1
Relative expression level
Cancer_cell
CD8T_typical
Mono/Macro
Neutrophil
H
GSE168652 CESC
CD8T_typical
Mono/Macro
Smooth_muscle
Fibroblast
Cancer_cell
Endometrial_stromal
Endothelial
I
GSE168652 CESC
CD3L1
UMAP1
UMAP2
J
GSE168652 CESC
CD3L1
Relative expression level
Cancer_cell
Endothelial
Fibroblast
Endometrial_stromal
Smooth_muscle
CD8T_typical
Mono/Macro

© The Author(s)

Figure EV2. Single-cell sequencing analysis reveals the positive correlation between CD3L1 expression and M2 TAMs in the TME of advanced solid tumors, related to Fig. 2.

(**A–D**) scRNA seq showing representative signature gene maps showing the upregulation of M1 TAM hallmark genes (**A**) CCL9 (**B**) CD74, and downregulation of M2 TAM hallmark genes (**C**) S100a9 (**D**) Csf1r after 10 mg/kg anti-CD3L1 treatment in comparison with control IgG treatment in the TME of osteosarcoma transplants isolated from nude mice. (**E**, **F**) UAMP plots showing (**E**) the cell clustering (**F**) the spatial expression of CD3L1 within the TME of public single-cell sequencing data from GSE155446 (Medulloblastoma) database. (**G**) The summary of CD3L1 expression level within the TME of GSE155446 (Medulloblastoma) database ($n = 28$ samples). (**H**, **I**) UAMP plots showing (**H**) the cell clustering (**I**) the spatial expression of CD3L1 within the TME of public single-cell sequencing data from GSE168652 (Cervical squamous cell carcinoma and endocervical carcinoma, CESC) database. (**J**) The summary of CD3L1 expression level within the TME of GSE168652 (CESC) database ($n = 2$ samples).

 © The Author(s)

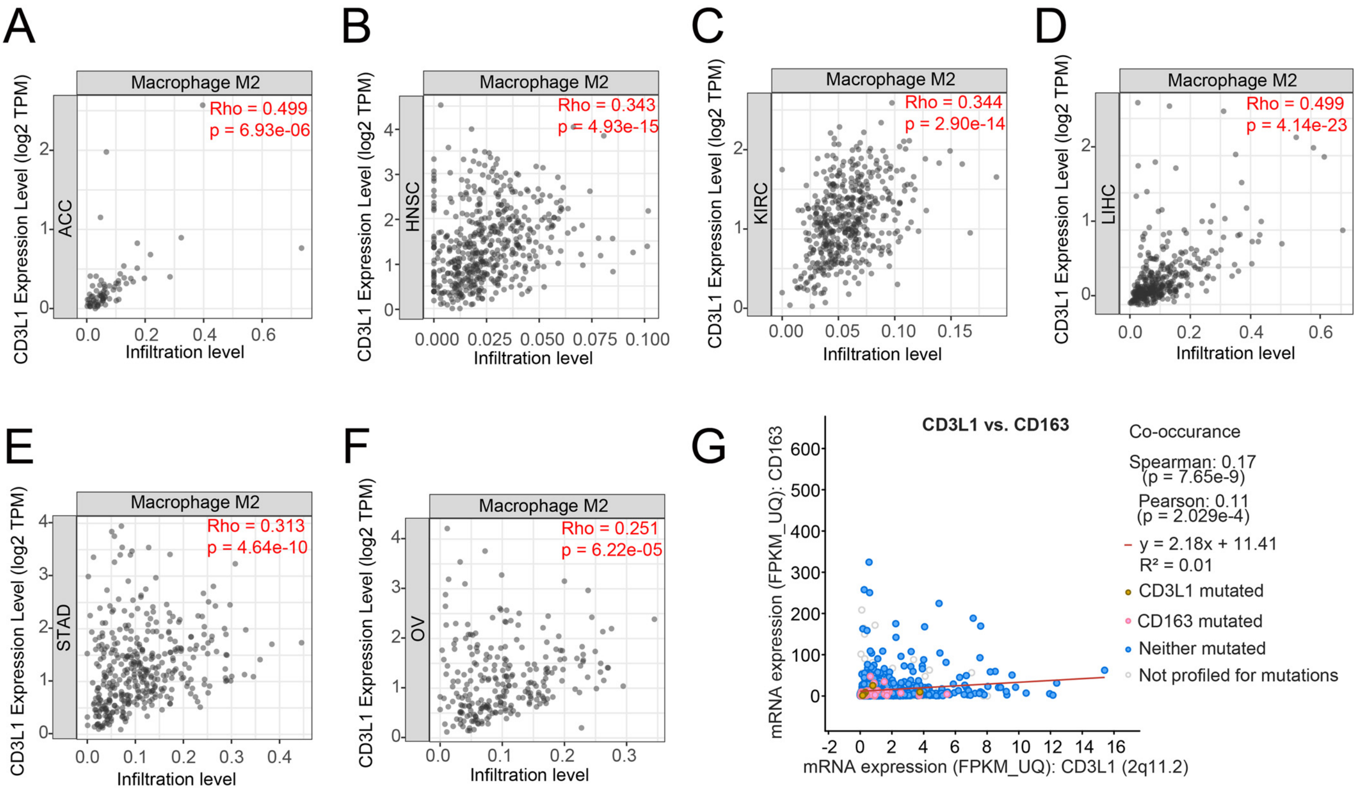

Figure EV3. Supplementary information of datasets analysis related to Fig. 2.

(**A–F**) Pan-cancer analysis of different solid tumor cohorts reveals a significant positive correlation between CD3L1 expression and M2 macrophage infiltration in the TME of (**A**) Adrenocortical carcinoma (ACC), (**B**) Head and neck squamous cell carcinoma (HNSC), (**C**) Kidney renal clear cell carcinoma (KIRC), (**D**) Liver hepatocellular carcinoma (LIHC), (**E**) Stomach adenocarcinoma (STAD), (**F**) Ovarian serous cystadenocarcinoma (OV). (**G**) TCGA pan-cancer atlas reveals the co-occurrence tendency and significant positive correlation between the CD3L1 expression level and the expression levels of M2 TAM marker genes CD163 in the TME across all types of cancer. (**A–G**) Correlation Analysis and Linear Regression Analysis (details in the methodological part).

© The Author(s)

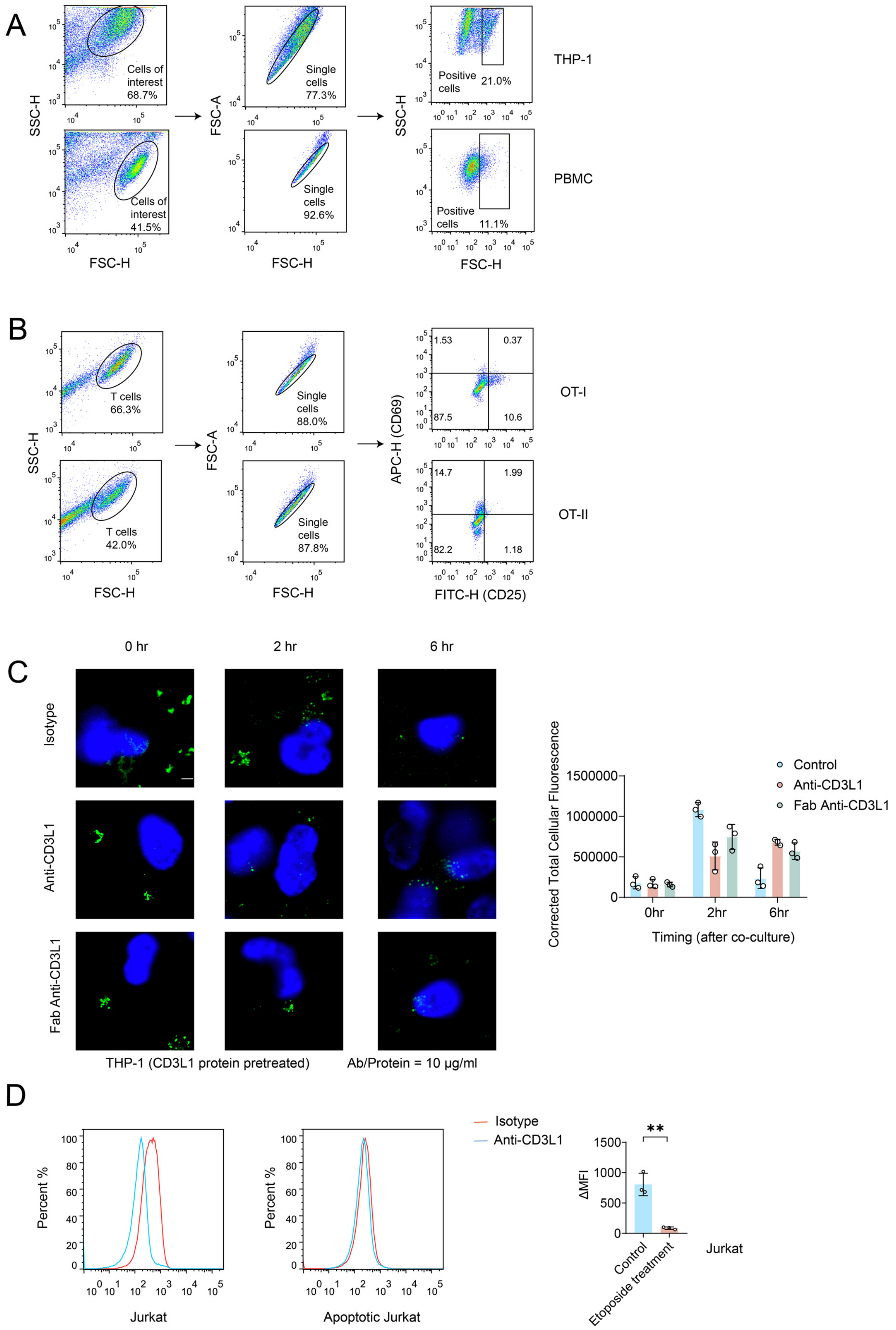

A
SSC-H
FSC-H
Cells of interest 68.7%
Cells of interest 41.5%
FSC-A
Single cells 77.3%
Single cells 92.6%
SSC-H
Positive cells 21.0%
Positive cells 11.1%
THP-1
PBMC
B
T cells 66.3%
T cells 42.0%
Single cells 88.0%
Single cells 87.8%
APC-H (CD69)
FITC-H (CD25)
1.53
0.37
87.5
10.6
14.7
1.99
82.2
1.18
OT-I
OT-II
C
0 hr
2 hr
6 hr
Isotype
Anti-CD3L1
Fab Anti-CD3L1
THP-1 (CD3L1 protein pretreated)
Ab/Protein = 10 μg/ml
Corrected Total Cellular Fluorescence
Control
Anti-CD3L1
Fab Anti-CD3L1
Timing (after co-culture)
0hr
2hr
6hr
D
Isotype
Anti-CD3L1
Percent %
Jurkat
Apoptotic Jurkat
ΔMFI
**
Control
Etoposide treatment
Jurkat

 © The Author(s)

Figure EV4. Supplementary information of the experiments related to Figs. 3-4.

(**A**) The gating strategy of the induced cells: cells were first gated by FSC-H/SSC-H to select out the main cell populations, and then gated by FSC-A/FSC-H to select out the single cells, and then gated by APC-H/SSC-H to select out the CD206-positive populations. (**B**) The gating strategy of the T cells: cells were first gated by FSC-H/SSC-H to select out the main cell populations, and then gated by FSC-A/FSC-H to select out the single cells, and then gated by APC-H/SSC-H and FITC-H/SSC-H to select out the CD69/CD25 positive populations. (**C**) The microscopic views (×60) of efferocytosis assay with induced THP-1 cells (pre-treated by CD3L1 protein) treated by control isotype or anti-CD3L1 or Fab anti-CD3L1 protein engulfing stained apoptotic Jurkat cells at timing 0, 2 and 6 h after the efferocytosis with the respective analysis showing significant downregulation in efferocytotic capacity of the induced M2-like TAMs from THP-1 cells after 10 μg/ml anti-CD3L1 treatment ($n = 3$ independent samples) (scale bar = 5 μm). (**D**) Representative flow cytometry results showing significant diminished binding between Jurkat cells and anti-CD3L1 after etoposide treatment ($n = 3$ independent samples). $^{**}P < 0.01$. Data are mean ± s.d. (**D**) Two-tailed unpaired Student's t test. Exact P values: Control vs Etoposide: $P = 0.0034$.

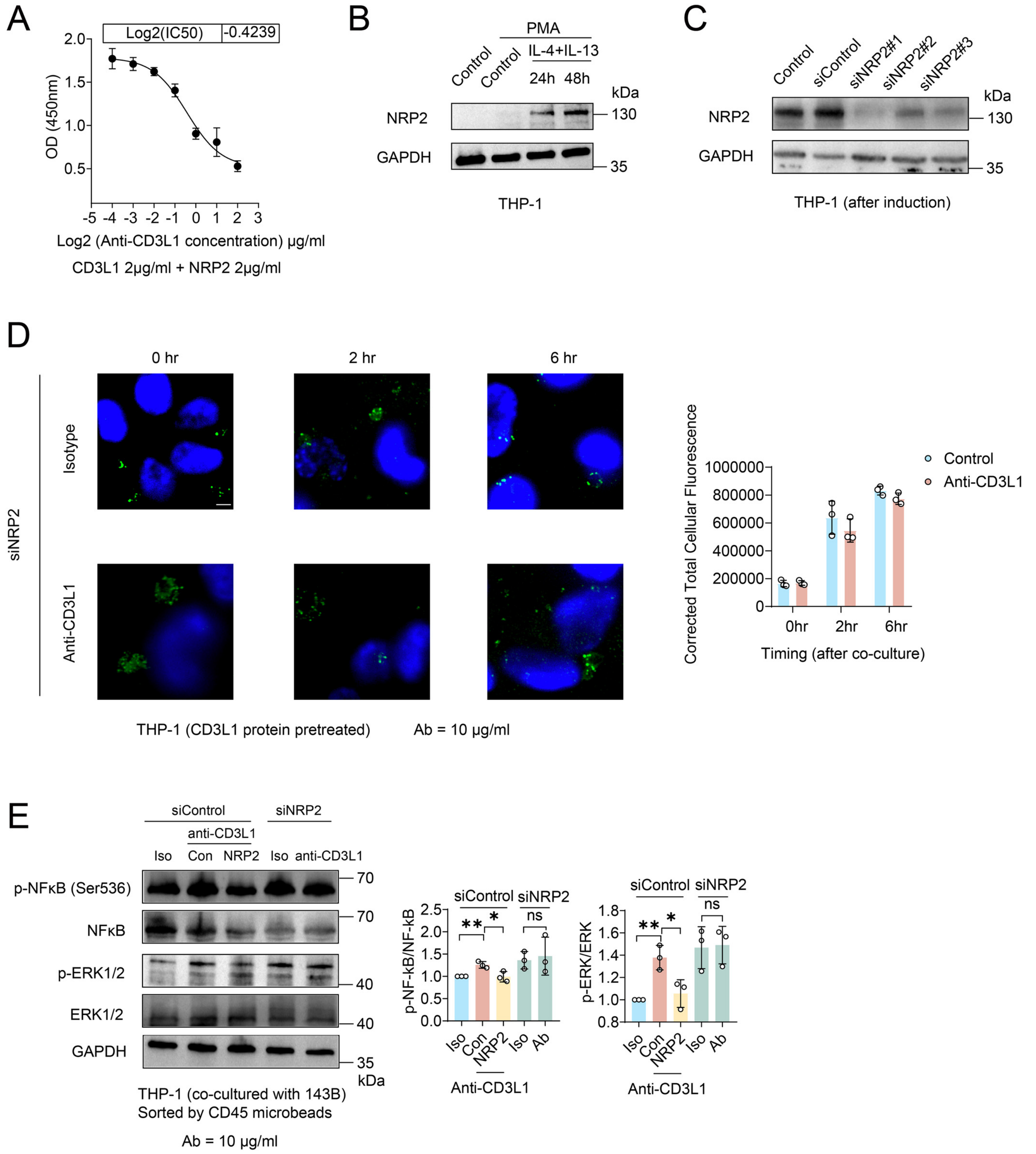
A
Log2(IC50) -0.4239
OD (450nm)
2.0
1.5
1.0
0.5
-5 -4 -3 -2 -1 0 1 2 3
Log2 (Anti-CD3L1 concentration) μg/ml
CD3L1 2μg/ml + NRP2 2μg/ml
B
PMA
IL-4+IL-13
Control
Control
24h 48h
kDa
NRP2
GAPDH
130
35
THP-1
C
Control
siControl
siNRP2#1
siNRP2#2
siNRP2#3
kDa
NRP2
GAPDH
130
35
THP-1 (after induction)
D
0 hr
2 hr
6 hr
siNRP2
Isotype
Anti-CD3L1
THP-1 (CD3L1 protein pretreated)
Ab = 10 μg/ml
Corrected Total Cellular Fluorescence
1000000
800000
600000
400000
200000
0
0hr 2hr 6hr
Timing (after co-culture)
Control
Anti-CD3L1
E
siControl
siNRP2
anti-CD3L1
Iso Con NRP2 Iso anti-CD3L1
p-NFκB (Ser536)
NFκB
p-ERK1/2
ERK1/2
GAPDH
70
70
40
40
35
kDa
THP-1 (co-cultured with 143B)
Sorted by CD45 microbeads
Ab = 10 μg/ml
p-NF-kB/NF-kB
siControl siNRP2
** * ns
2.5
2.0
1.5
1.0
0.5
0.0
Iso Con NRP2 Iso Ab
Anti-CD3L1
p-ERK/ERK
siControl siNRP2
** * ns
1.8
1.6
1.4
1.2
1.0
0.8
Iso Con NRP2 Iso Ab
Anti-CD3L1

 © The Author(s)

Figure EV5. Supplementary information of the experiments related to Fig. 5.

(**A**) ELISA showing blockade of CD3L1-NRP2 interaction by different concentrations of anti-CD3L1 ($n = 3$ independent wells). (**B**) Immunoblot showing the NRP2 expression in different stages of induced THP-1 cells ($n = 3$ independent experiments). (**C**) Immunoblot showing the knockdown effect of NRP2 by different siRNAs in THP-1 cells ($n = 3$ independent experiments). (**D**) The microscopic views (×60) of efferocytosis assay with induced THP-1 cells (pre-treated by CD3L1 protein) after siRNAs treatment treated by control isotype or anti-CD3L1 engulfing stained apoptotic Jurkat cells at timing 0, 2, and 6 h after the efferocytosis with the respective analysis showing no significant downregulation in efferocytotic capacity of the M2-like TAM induced THP-1 cells with siRNA knocking down NRP2 treatment after 10 µg/ml anti-CD3L1 treatment ($n = 3$ independent samples) (scale bar = 5 µm). (**E**) Representative immunoblot results showing that anti-CD3L1 can counteract the inhibition of NRP2 on the downstream signaling pathways (NFκB and MAPK-ERK), which can be restored by NRP2 protein and diminished by NRP2 knockdown ($n = 3$ independent experiments). *$P < 0.05$, **$P < 0.01$, with ns as no significance. Data are mean ± s.d. (**E**) Two-tailed unpaired Student's *t* test. Exact *P* values: pNF-κB/NF-κB: siControl: Isotype vs anti-CD3L1: $P = 0.0040$; anti-CD3L1 vs NRP2 protein: $P = 0.0296$; siNRP2: Isotype: anti-CD3L1: $P = 0.7501$;pERK/ERK: siControl: Isotype vs anti-CD3L1: $P = 0.0040$; anti-CD3L1 vs NRP2 protein: $P = 0.0281$; siNRP2: Isotype: anti-CD3L1: $P = 0.8837$.

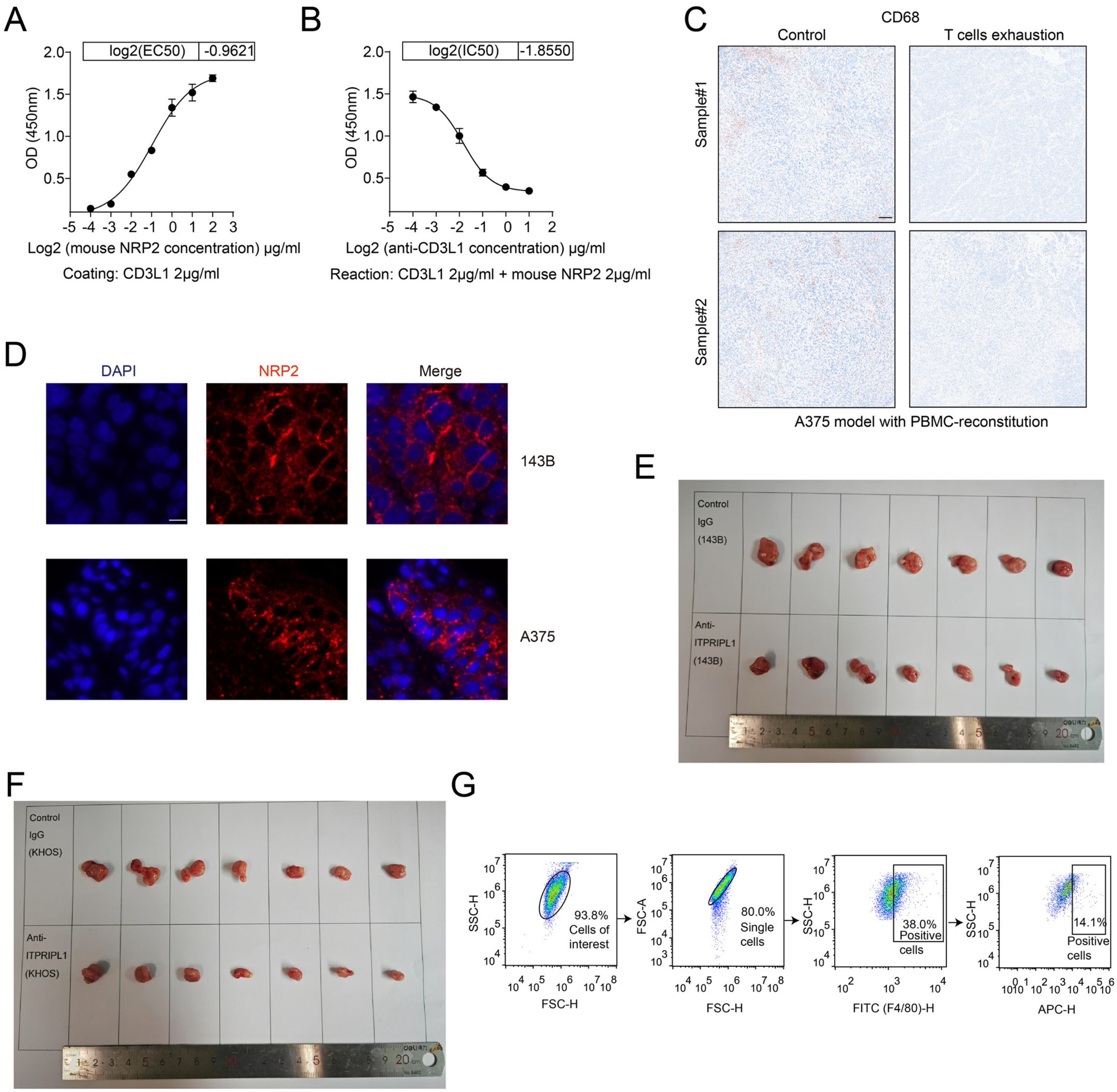

Figure EV6. Supplementary information of the in vivo experiments related to Fig. 6.

(**A**, **B**) ELISA binding curve showing great affinity between human CD3L1 and mouse NRP2 protein, which can be blocked by anti-CD3L1 antibody ($n = 3$ independent wells). (**C**) IHC staining of the human macrophage marker CD68 in the A375 transplant samples in our previous PBMC-reconstituted model under ×5 and ×20 microscopic views, showing a significant decrease in CD68-positive cells after T-cell exhaustion ($n = 3$ independent samples, scale bar = 50 μm). (**D**) Representative images showing the expression of NRP2 in the 143B (osteosarcoma) and A375 (melanoma) transplant samples under ×60 microscopic view ($n = 3$ independent samples) (scale bar = 10 μm). (**E**, **F**) Macroscopic views of the (**E**) 143B (**F**) KHOS tumors isolated from nude mice tumor transplant ($n = 7$ mice per group). (**G**) The gating strategy of the isolated immune cells infiltrated in the tumor microenvironment of the nude mice: cells were first gated by FSC-H/SSC-H to select out the main cell populations, and then gated by FSC-A/FSC-H to select out the single cells, and then gated by FITC-H/SSC-H to select out the F4/80-positive populations, and then gated by APC-H/SSC-H to select out the CD86/CD206-positive groups. Data are mean ± s.d.

 © The Author(s)
