# Supplementary material for: Targeting CD3L1-NRP2 disarms myeloid-driven tumor immune evasion
Source: EMBO Mol Med. 2026 May 15;18(7):2635–66. doi: 10.1038/s44321-026-00451-3 (PMC13365830; doi:10.1038/s44321-026-00451-3)
Supplement: Supplementary file 3 — Peer Review File [file 44321_2026_451_MOESM3_ESM.pdf]

EMBO Molecular Medicine

Targeting CD3L1-NRP2 disarms myeloid-driven tumor immune evasion

Shouyan Deng, Yuan Fang, Jieyuan Xue, Rui Wang, Xiaolin Zhou, Chushu Li, Ning Li, Shuhang Wang, Yibo Zhang, Huanbin Wang, Jianghong Yu, and Jie Xu

Corresponding author(s): Jie Xu (jie_xu@fudan.edu.cn) , Shouyan Deng (shouyandeng@fudan.edu.cn)

Review Timeline:

Submission Date:	9th Oct 25
Editorial Decision:	12th Nov 25
Revision Received:	16th Jan 26
Editorial Decision:	4th Mar 26
Revision Received:	20th Mar 26
Editorial Decision:	21st Apr 26
Revision Received:	29th Apr 26
Accepted:	7th May 26

Editor: Lise Roth

Transaction Report:

(Note: With the exception of the correction of typographical or spelling errors that could be a source of ambiguity, letters and reports are not edited. Depending on transfer agreements, referee reports obtained elsewhere may or may not be included in this compilation. Referee reports are anonymous unless the Referee chooses to sign their reports.)

12th Nov 2025

Dear Dr. Xu,

Thank you for submitting your manuscript to EMBO Molecular Medicine, and please accept my apologies for the delay in getting back to you as one of the referees needed more time to complete his/her review. We have now heard back from the three referees. As you will see below, they mention the potential interest and novelty of the findings, but also request stronger mechanistic demonstration and alternative in vivo models.

If you feel that you can satisfactorily address the points raised by the reviewers, you may wish to submit a revised version of your manuscript. Please include a covering letter detailing how you have addressed each of the points raised by the reviewers. A revised manuscript will be reviewed again, and we cannot guarantee at this stage that the final outcome will be favorable.

Revising the manuscript according to the reviewers' recommendations appears to require a lot of additional work and experimentation, and given the potential interest of your findings, we are prepared to extend the deadline to 6 months.

If you would like to discuss further the points raised by the referees or your revision plan, I am available to do so via email or video. Let me know if you are interested in this option.

EMBO Molecular Medicine encourages a single round of revision only and therefore, acceptance or rejection of the manuscript will depend on the completeness of your responses included in the next, final version of the manuscript. For this reason, and to save you from any frustrations in the end, I would strongly advise against returning an incomplete revision. Should you find that the requested revisions are not feasible within the constraints outlined here and prefer, therefore, to submit your paper elsewhere, we would welcome a message to this effect.

When submitting your revised manuscript, please carefully review the instructions that follow below. We perform an initial quality control of all revised manuscripts before re-review; failure to include requested items will delay the evaluation of your revision.

1) A .docx formatted version of the manuscript text (including legends for main figures, EV figures and tables). Please make sure that the changes are highlighted to be clearly visible.

2) Individual production quality figure files as .eps, .tif, .jpg (one file per figure). For guidance, download the 'Figure Guide PDF' (https://www.embopress.org/page/journal/17574684/authorguide#figureformat).

3) At EMBO Press we ask authors to provide source data for the main figures. Our source data coordinator will contact you to discuss which figure panels we would need source data for and will also provide you with helpful tips on how to upload and organize the files.

4) A .docx formatted letter INCLUDING the reviewers' reports and your detailed point-by-point responses to their comments. As part of the EMBO Press transparent editorial process, the point-by-point response is part of the Review Process File (RPF), which will be published alongside your paper.

5) A complete author checklist, which you can download from our author guidelines (https://www.embopress.org/page/journal/17574684/authorguide#submissionofrevisions). Please insert information in the checklist that is also reflected in the manuscript. The completed author checklist will also be part of the RPF.

6) All Materials and Methods need to be described in the main text using our 'Structured Methods' format. According to this format, the Methods section includes a Reagents and Tools Table (listing key reagents, experimental models, software and relevant equipment and including their sources and relevant identifiers) followed by a Methods and Protocols section describing the methods, ideally using a step-by-step protocol format. The aim is to facilitate adoption of the methodologies across labs.
Please download and fill our Reagents and Tools Table template (.docx), which you can find in our author guidelines: https://www.embopress.org/page/journal/14693178/authorguide#structuredmethods.
When submitting your revised manuscript, please do not include the Reagents and Tools Table in the Methods section of the manuscript but upload it as a separate file choosing the file type "Reagent Table".
An example of a Method paper with Structured Methods can be found here:
https://www.embopress.org/doi/10.15252/msb.20178071

7) Please note that all corresponding authors are required to supply an ORCID ID for their name upon submission of a revised manuscript.

8) It is mandatory to include a 'Data Availability' section after the Materials and Methods. Before submitting your revision, primary datasets produced in this study need to be deposited in an appropriate public database, and the accession numbers and database listed under 'Data Availability'. Please remember to provide a reviewer password if the datasets are not yet public (see https://www.embopress.org/page/journal/17574684/authorguide#dataavailability).

In case you have no data that requires deposition in a public database, please state so in this section. Note that the Data Availability Section is restricted to new primary data that are part of this study.

9) For data quantification: please specify the name of the statistical test used to generate error bars and P values, the number (n) of independent experiments (specify technical or biological replicates) underlying each data point and the test used to calculate p-values in each figure legend. The figure legends should contain a basic description of n, P and the test applied. Graphs must include a description of the bars and the error bars (s.d., s.e.m.). Please provide exact p values.

10) Our journal encourages inclusion of *data citations in the reference list* to directly cite datasets that were re-used and obtained from public databases. Data citations in the article text are distinct from normal bibliographical citations and should directly link to the database records from which the data can be accessed. In the main text, data citations are formatted as follows: "Data ref: Smith et al, 2001" or "Data ref: NCBI Sequence Read Archive PRJNA342805, 2017". In the Reference list, data citations must be labeled with "[DATASET]". A data reference must provide the database name, accession number/identifiers and a resolvable link to the landing page from which the data can be accessed at the end of the reference. Further instructions are available at .

11) We replaced Supplementary Information with Expanded View (EV) Figures and Tables that are collapsible/expandable online. EV Figures should be cited as 'Figure EV1, Figure EV2" etc... in the text and their respective legends should be included in the main text after the legends of regular figures.
- For the figures that you do NOT wish to display as Expanded View figures, they should be bundled together with their legends in a single PDF file called *Appendix*, which should start with a short Table of Content. Appendix figures should be referred to in the main text as: "Appendix Figure S1, Appendix Figure S2" etc.
- Additional Tables/Datasets should be labeled and referred to as Table EV1, Dataset EV1, etc. Legends have to be provided in a separate tab in case of .xls files. Alternatively, the legend can be supplied as a separate text file (README) and zipped together with the Table/Dataset file.
See detailed instructions here:

12) The paper explained: EMBO Molecular Medicine articles are accompanied by a summary of the articles to emphasize the major findings in the paper and their medical implications for the non-specialist reader. Please provide a draft summary of your article highlighting
- the medical issue you are addressing,
- the results obtained and
- their clinical impact.
This may be edited to ensure that readers understand the significance and context of the research. Please refer to any of our published articles for an example.

13) Author contributions: CRediT has replaced the traditional author contributions section because it offers a systematic machine readable author contributions format that allows for more effective research assessment. Please remove the Authors Contributions from the manuscript and use the free text boxes beneath each contributing author's name in our system to add specific details on the author's contribution. More information is available in our guide to authors.

14) Disclosure statement and competing interests: We updated our journal's competing interests policy in January 2022 and request authors to consider both actual and perceived competing interests. Please review the policy https://www.embopress.org/competing-interests and update your competing interests if necessary.

15) Every published paper now includes a 'Synopsis' to further enhance discoverability. Synopses are displayed on the journal webpage and are freely accessible to all readers. They include a short stand first (maximum of 300 characters, including space) as well as 2-5 one-sentences bullet points that summarizes the paper. Please write the bullet points to summarize the key NEW findings. They should be designed to be complementary to the abstract - i.e. not repeat the same text. We encourage inclusion of key acronyms and quantitative information (maximum of 30 words / bullet point). Please use the passive voice. Please attach these in a separate file or send them by email, we will incorporate them accordingly.

Please also suggest a visual abstract to illustrate your article as a PNG file 550 px wide x 300-600 px high. A cropped portion of this image will serve as thumbnail for the table of content on our webpage.

16) As part of the EMBO Publications transparent editorial process initiative (see our Editorial at http://embomolmed.embopress.org/content/2/9/329), EMBO Molecular Medicine will publish online a Review Process File (RPF) to accompany accepted manuscripts.
In the event of acceptance, this file will be published in conjunction with your paper and will include the anonymous referee reports, your point-by-point response and all pertinent correspondence relating to the manuscript. Let us know whether you agree with the publication of the RPF and as here, if you want to remove or not any figures from it prior to publication.
Please note that the Authors checklist will be published at the end of the RPF.

EMBO Molecular Medicine has a "scooping protection" policy, whereby similar findings that are published by others during review or revision are not a criterion for rejection. Should you decide to submit a revised version, I do ask that you get in touch after three months if you have not completed it, to update us on the status.

I look forward to receiving your revised manuscript.

Yours sincerely,

Lise Roth

Lise Roth, PhD
Senior Editor
EMBO Molecular Medicine

***** Reviewer's comments *****

Referee #1 (Remarks for Author):

In the present manuscript Deng S et al highlights a potential novel mechanism of an anti-CD3L1 antibody, which previously has been shown to restrain tumor growth and support the infiltration and activation of T cells.
The study convincingly shows anti-CD3L1 induced phenotypic changes in macrophages using different in vivo and in vitro model systems. Neuropilin 2 (NRP2) is proposed as major binding partner for CD3L1 on macrophages that is driving this phenotypic alteration.
While this study reveals novel aspects of the anti-CD3L1 treatment, the mechanistic part of the study requires further development and several points need further elaboration to clarify the underlying processes.

o Throughout the manuscript the authors highlighted the anti-CD3L1 mediated reprogramming of macrophages towards an M1 like phenotype, with enhanced T cell activation and reduced efferocytosis capabilities. However anti-CD3L1 treatment seem to control tumors even in the absence of T cells. At present the T cell independent anti-tumor effect of the anti-CD3L1 treatment is not sufficiently explored.
Further, the contribution of Macrophages in treatment induced tumor control has not been elaborated and would need experimental validation.

o The histological investigation of TAMs in the melanoma patient as well as the murine osteosarcoma model demonstrates elevated numbers of tumor associated macrophages (TAMs) after anti-CD3L1 treatment and suggests a phenotypic shift associated with loss of CD206+ expressing cells.
Quantitative image analysis of the histological data should be performed to compare the frequency of CD206+ and CD206- Macrophages in the TME before and after treatment in the different settings. This would disentangle the contribution of cellular infiltration and conversion.
As CD11b is rather a common myeloid marker and not exclusive for TAMs, the use of complementary markers or a multifluorescent imaging approach would be advisable.

o The manuscript would benefit from additional validation of the functional changes induced by the anti-CD3L1 treatment in macrophages using physiologically relevant cellular systems to support the proposed mechanism.
• Based on the data provided in S4A, the CD3L1 expression in THP1 cells is independent of the polarization status. To evaluate the expression level of CD3L1 in a physiological context similar data should be provided for PBMC derived Monocytes & differentiated Macrophages.
Along these lines, treatment induced changes in expression level of selected functional M2 markers are currently only shown for THP1 cells in Fig.3 E, although analysis in M2 TAMs from PBMCs is suggested in the figure legend.

• While the current ex vivo T cell activation assay illustrated in Fig. 3F suggests an enhanced T cell activation by anti-CD3L1 treated BMDMs, the experimental set-up does not allow any conclusions to be drawn about the exact mechanism.
A comprehensive investigation on the activation and maturation state of the anti-CD3L1 treated BMDMs combined with a functional T cell profiling (including cytokine production and proliferation) would be needed to evaluate the capabilities of these BMDMs to act as antigen-presenting cells.
Did the investigators also attempt to determine activation of CD4 T cells?

o Binding assays showed an interaction of NRP2 and CD3L1. However, the functional consequence of this interaction remains largely unclear.
Lack of the anti-CD3L1 mediated macrophage re-polarization in the absence of NRP2 supports the hypothesis that CD3L1 acts via the NRP2 axis. However, the absence of an additional effect could also suggest that the lack of NRP2 already elicits the maximal effect. Thus, further experimental data that explore the relevance of this interaction would be needed to strengthen the currently proposed mechanism of action in macrophages.

o While several data of bulk RNA sequencing analysis from publicly available data sets (e.g. Figure 2K-M) are provided in the manuscript, there is no description of how this analysis was performed. To guarantee transparency and reproducibility this needs to be added.

o The relative expression level based on the single cell data re-analysis shown in Figure 2J seems to be rather marginal. Statistical analysis should be provided to support the identification of the main target cell population.

Minor points:

o While in the text an elevated expression level of CD3L1, limited T cell infiltration and a crucial role of TAMs in osteosarcoma TME has been highlighted in the first results paragraph, there is at that point of the manuscript no data provided that supports this statement. Therefore, corresponding references should be provided.

o Although reference was made to the previous publication, this manuscript should also specify how the humanized mouse model was set-up for use in osteosarcoma model in Fig1 D.

o The description of Figure 2C is missing in the corresponding Figure Legend

o In the text the Figure 3C and D, are wrongly assigned.

o The correction for multiple testing for certain statistical tests should be indicated.

Referee #2 (Comments on Novelty/Model System for Author):

This is a strong and conceptually interesting follow-up to the first study, but the mechanistic discrepancy between the A375 and osteosarcoma models, and the reliance on xenografts, should be addressed directly. Demonstrating that macrophage reprogramming occurs in syngeneic models or explaining its absence in A375 tumors is in my view critical to reconcile the

group's two papers and to establish CD3L1 as a dual- (inate and adaptive) targeting immune modulator.

Referee #2 (Remarks for Author):

Summary
This manuscript extends on the group's previous study published in Cell (Deng et al., 2024) that described CD3L1 as a ligand of CD3ε and that showed by binding to CD3 it is suppressing T-cell activation. This study proposes a new and distinct, T-cell independent macrophage mechanism of immune suppression- suggesting that CD3L1 also interacts with Neuropilin-2 on tumor-associated macrophages (TAMs), driving macrophage into a different type of polarization. Their data suggests that their anti-CD3L1 antibody treatment blocks interaction with NRP2 and thereby reprograms TAMs toward an M1 phenotype, and inhibits tumor growth in T-cell-deficient osteosarcoma xenografts. Unsing single-cell RNA-seq, flow cytometry, and IHC analyses the authors suggest reduced M2 and increased M1 polarized TAMs after treatment. This study thus proposes a conceptual extension of the earlier study by shifting or extending the inhibitory activity from adaptive immunity - blocking T-cell activation, to innate immunity, changing macrophage polarization.

The strength of this study is that in proposes a plausible dual mechanism for their novel checkpoint inhibitor, CD3L1 (that they have patented for immunothereapy of cancer) in not only suppressing T cells (via binding CD3ε) but also modulating macrophages via binding to NRP2. The study presents coherent in-vitro and in-vivo data in which they try to link anti-CD3L1 blockade to TAM reprogramming and tumor inhibition in a xenogenic osteosarcoma model.

Major critique/concerns
1) Why was the contribution of "repolarizing" the myeloid cells not visible in their initial paper for the xenograft A375 tumor model after T cell depletion? In the Cell study, T cell depletion abolished anti-CD3L1 efficacy in A375 xenografts, implying a strick T-cell-dependent mechanism. Yet in the current study, anti-CD3L1 retains efficacy in T-cell-deficient nude mice. Therefore, why was no macrophage-mediated effect observed in the earlier A375 model? Do A375 tumors lack a significant myeloid compartment, or are their macrophages refractory to M1 conversion by CD3L1 blockade?
2) Concerning the choice of model why was a nude xenograft model used to test their hypothesis in-vivo instead of the earlier used syngeneic models? The authors use human osteosarcoma xenografts in nude mice to demonstrate T-cell independent activity. However, syngeneic murine models such as the B16 melanoma or the MC38 colorectal cancer models already used in the earlier study would have permitted controlled T-cell depletion or genetic NRP2 manipulation within a fully immunocompetent microenvironment and would have provided a realistic and relevant approximation of the T cell vs. inate contribution to the immunosupressive activity of CD3L1. Why not use these models to quantify the relative contributions of T cells and myeloid cells, instead of relying on correlative scRNA-seq data from xenografts?

3. The evidence for CD3L1-NRP2 binding is based primarily on ELISA. An independent confirmation e.g. by co-immunoprecipitation or proximity ligation in primary macrophages would have strengthened their claim and thus the central mechanism proposed.

Minor points
It would be interesting to clarify whether the antibody's effects depend on Fc receptor binding or whether a ligand blockade with Fab- Fragments is sufficient.

It might be also interesting to analyze NRP2 expression patterns in osteosarcoma versus melanoma.
In my view in the text it should be more explicitly explained that the observation stems from a single melanoma patient case (anecdotal).

Overall assesment
This is a strong and conceptually interesting follow-up to the first study, but the mechanistic discrepancy between the A375 and osteosarcoma models, and the reliance on xenografts, should be addressed directly. Demonstrating that macrophage reprogramming occurs in syngeneic models or explaining its absence in A375 tumors is in my view critical to reconcile the group's two papers and to establish CD3L1 as a dual- (inate and adaptive) targeting immune modulator.

Referee #3 (Comments on Novelty/Model System for Author):

The strategy employed to identify/describe the effect of anti-CD3L1 on macrophages is solid. The differences in the control and anti-CD3L1 treated samples (clinical and pre-clinical) are apparent and interesting. Overall the descriptive data (Fig 1 & 2) is adequate.
The novelty is high: a) working with a clinical stage drug/drug target; b) ID of a potential new ligand for an apparent ICB target.
The medical impact is medium due to challenges listed below with the model system. The model system to work out the biology of how CD3L1 impacts the immune microenvironment is unclear.
Challenges: 1) the authors point out in the summary figure and in the text that CD3L1 is likely mainly expressed on tumor cells. No effort is made to demonstrate that tumor cell CD3L1 signals in tumor cells or that tumor cell expression of CD3L1 is required

for the biology observed or the efficacy of the anti-CD3L1. 2) The CD3L1 signaling studies are performed in THP-1 (myeloid). The authors propose that CD3L1 on tumor cells or potential other cells interacts with Nrp2 on macrophages and this drives the M2-like phenotype. However, there are no studies demonstrating that Nrp2 signals, how it signals. They only look at siNrp2. Further the studies are in vitro and without tumor cells. I do not think the models employed are adequate to make the conclusions drawn, this impacts the medical impact and the technical quality.

Referee #3 (Remarks for Author):

Deng et al investigate the effect of anti-CD3L1 on the tumor immune microenvironment. Anti-CD3L1 are in clinical trial and the authors investigated the effect of ant-CD3L1 on macrophage infiltration and marker expression in clinical samples and preclinical tumor models. Interestingly, treatment of anti-CD3L1 resulted in an increase in M1-like macrophages and a decrease in macrophages with a M2-like phenotype. They show this by IHC and omics techniques. These data are convincing. The authors suggest that CD3L1 is expressed by tumor cells and other cells, including macrophages in tumors. They show in vitro treatment of M2-polarized THP-1 cells with anti-CD3L1 results in reduction of pSTAT6 and expression of cytokines are other M2-like markers and an increase in the APC capacity of BMDMs. They investigate potential CD3L1 binding partners via biochemistry with purified proteins from a candidate list and find that Nrp2 binds to CD3L1. This is interesting. They provide data that anti-CD3L1 blocks Nrp2-CD3L1 interaction. Overall it is an interesting study that has some strong points, namely it is centered around a clinical stage antibody and they have identified a putative new ligand/interactor for CD3L1.

Comments:

1. There is a disconnect between where CD3L1 is expressed and how it impacts macrophage phenotype. Is CD3L1 signaling on macrophages relevant or is it Nrp2 on macrophages that is signaling. In either case, how, what is driving the signaling. If as Fig 3 and 4 suggest CD3L1 on macrophages is signaling, what is driving that signal? Is it Nrp2 on the same cell, on adjacent macrophages, on other cells? The story was setup to suggest that CD3L1 on tumor cells was interacting with a partner on macrophages but that is not how it ended.

2. The importance of Nrp2 as a CD3L1 binder to the biology of anti-CD3L1 (the change in macrophage phenotype) is not clear based on the data shown. Demonstrate that Nrp2 expression on macrophages (if that is where you think it is important) matters to the phenotype in vivo.

3. The efferocytosis assays in Figure 3 and in supplement need clarification. Is the engulfment of apoptotic Jurkat cells Fc-dependent? Does anti-CD3L1 bind apoptotic Jurkat cells? Is the anti-CD3L1 still in the system when the target cell (Jurkat) added to the macrophages?

Referee #1 (Remarks for Author):

In the present manuscript Deng S et al highlights a potential novel mechanism of an anti-CD3L1 antibody, which previously has been shown to restrain tumor growth and support the infiltration and activation of T cells.
The study convincingly shows anti-CD3L1 induced phenotypic changes in macrophages using different in vivo and in vitro model systems. Neuropilin 2 (NRP2) is proposed as major binding partner for CD3L1 on macrophages that is driving this phenotypic alteration. While this study reveals novel aspects of the anti-CD3L1 treatment, the mechanistic part of the study requires further development and several points need further elaboration to clarify the underlying processes.

o Throughout the manuscript the authors highlighted the anti-CD3L1 mediated reprogramming of macrophages towards an M1 like phenotype, with enhanced T cell activation and reduced efferocytosis capabilities. However anti-CD3L1 treatment seem to control tumors even in the absence of T cells. At present the T cell independent anti-tumor effect of the anti-CD3L1 treatment is not sufficiently explored.
Further, the contribution of Macrophages in treatment induced tumor control has not been elaborated and would need experimental validation.

We deeply appreciate your comments. We have now provided more robust evidence to support our conclusion in accordance with your constructive suggestions, and have undertaken a comprehensive revision of the entire manuscript to strengthen our findings and improve the clarity.

o The histological investigation of TAMs in the melanoma patient as well as the murine osteosarcoma model demonstrates elevated numbers of tumor associated macrophages (TAMs) after anti-CD3L1 treatment and suggests a phenotypic shift associated with loss of CD206+ expressing cells.
Quantitative image analysis of the histological data should be performed to compare the frequency of CD206+ and CD206- Macrophages in the TME before and after treatment in the different settings. This would disentangle the contribution of cellular infiltration and conversion.
As CD11b is rather a common myeloid marker and not exclusive for TAMs, the use of complementary markers or a multifluorescent imaging approach would be advisable.

Thank you for your thoughtful concerns. In response to your valuable suggestions, we have conducted quantitative IHC analysis using IHC score analysis to assess CD68 and CD206 expression in the TME both before and after treatment. The detailed methodology for this

analysis has been elaborated in the corresponding section of our revised manuscript.

Immunohistochemistry

The tumor samples were fixed with 4% PFA for 24 hours and embedded by paraffin and cut into slides. The slides were first heated for 2 hours and sequentially washed by xylene, 100% ethanol, 90% ethanol, 70% ethanol, 50% ethanol, PBS, each for 10 minutes. Then the slides were tapped with 3% perhydrol for 15 minutes and washed with PBS for 5 minutes, 3 times. The slides were then repaired by sodium citrate solution by heat. Then the murine tumor specimens were incubated with antibodies against CD86 /CD206. The human tumor specimens were incubated with antibodies against CD68/CD206. After three times PBS washing, the tissues were then incubated with a biotin-conjugated secondary antibody, followed by avidin-biotin–peroxidase complex. The slides were sealed by neutral balsam. The images were all scanned and analyzed by IHC assessment standard. The cutoff standard was assessed by the IHC assessment standard, that the staining scores can be calculated by multiplying area (1: 0~25%, 2: 26~50%, 3: 51~75%, 4: 76~100%) and intensity (0: negative, no yellow staining; 1: shallow yellow staining; 2: brown yellow staining; 3: dark brown staining), that 0 should be considered as negative, 1~3 as low, 4~8 as medium and 9~12 as high according to pathological studies.

Furthermore, to provide a more comprehensive characterization of the macrophage population within the TME, we have now incorporated IHC staining results for CD68, a widely recognized macrophage marker. Representative staining images have been included in **Fig.1D-E** to illustrate the findings.

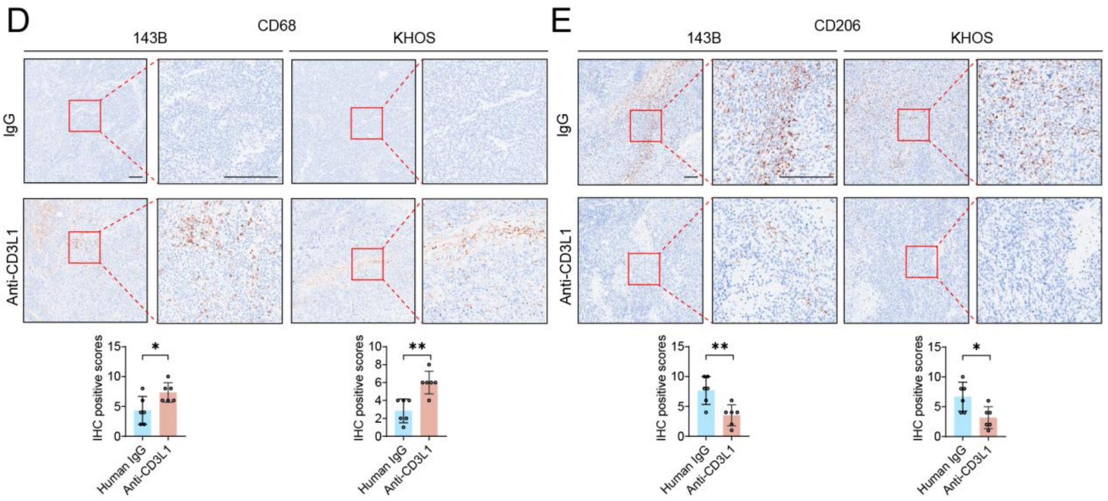

(The marker of functional macrophages has switched to CD68 with IHC positive scores calculation in independent fields as quantitative analysis)

We believe these additions will strengthen the interpretation of our data and address your concerns effectively.

o The manuscript would benefit from additional validation of the functional changes induced by the anti-CD3L1 treatment in macrophages using physiologically relevant

cellular systems to support the proposed mechanism.

• Based on the data provided in S4A, the CD3L1 expression in THP1 cells is independent of the polarization status. To evaluate the expression level of CD3L1 in a physiological context similar data should be provided for PBMC derived Monocytes & differentiated Macrophages.

Along these lines, treatment induced changes in expression level of selected functional M2 markers are currently only shown for THP1 cells in Fig.3 E, although analysis in M2 TAMs from PBMCs is suggested in the figure legend.

We sincerely appreciate your constructive feedback, which has prompted us to critically re-evaluate our experimental design and data interpretation. Following your guidance, we have redesigned all *in vitro* experiments using a co-culture system of tumor cells (expressing CD3L1) and induced macrophages (expressing NRP2), as this model more accurately reflects the proposed biological mechanism of CD3L1-NRP2 crosstalk in the TME. Our initial approach, which utilized THP-1-derived macrophages only, was based on the assumption that these cells could serve as a simplified system for cells expressing CD3L1 and macrophages expressing NRP2. However, we now recognize that this system fails to adequately recapitulate the physiological interactions between tumor cells and TAMs, particularly regarding CD3L1-NRP2-mediated signaling pathways. THP-1 cells are recognized as tumor cells, whereas CD3L1 exhibits a propensity for high expression in malignant cells, serving as a mechanism for immune evasion. This pattern is analogous to the observation that normal lung tissues do not express CD3L1, whereas lung cancer tissues overexpress CD3L1 (our research work published in 2023, Frontiers in Cell and Developmental Biology). Consequently, we have decided to abandon the use of tumor cell-derived models only in this particular section and instead adopt a more physiologically relevant co-culture model. We deeply regret any confusion caused by our initial oversight and have removed all data derived from the initial system from the revised manuscript. Our assessments of PBMCs revealed minimal expression of CD3L1 in immune cells, which might explain the effect of anti-CD3L1 in our initial manuscript, and our main focus now centers on direct CD3L1 (tumor cells expressing) - NRP2 (macrophages expressing) interactions. Additionally, we acknowledge and correct the misleading description in the original figure legend of **Fig.3E (new Fig.3E)** that only THP-1-derived model was utilized for this experiment. The revised figure captions now state the use of the tumor cell-macrophage co-culture system and clarify the experimental conditions. We thank you again for highlighting these critical issues, which have significantly strengthened the scientific rigor of our study.

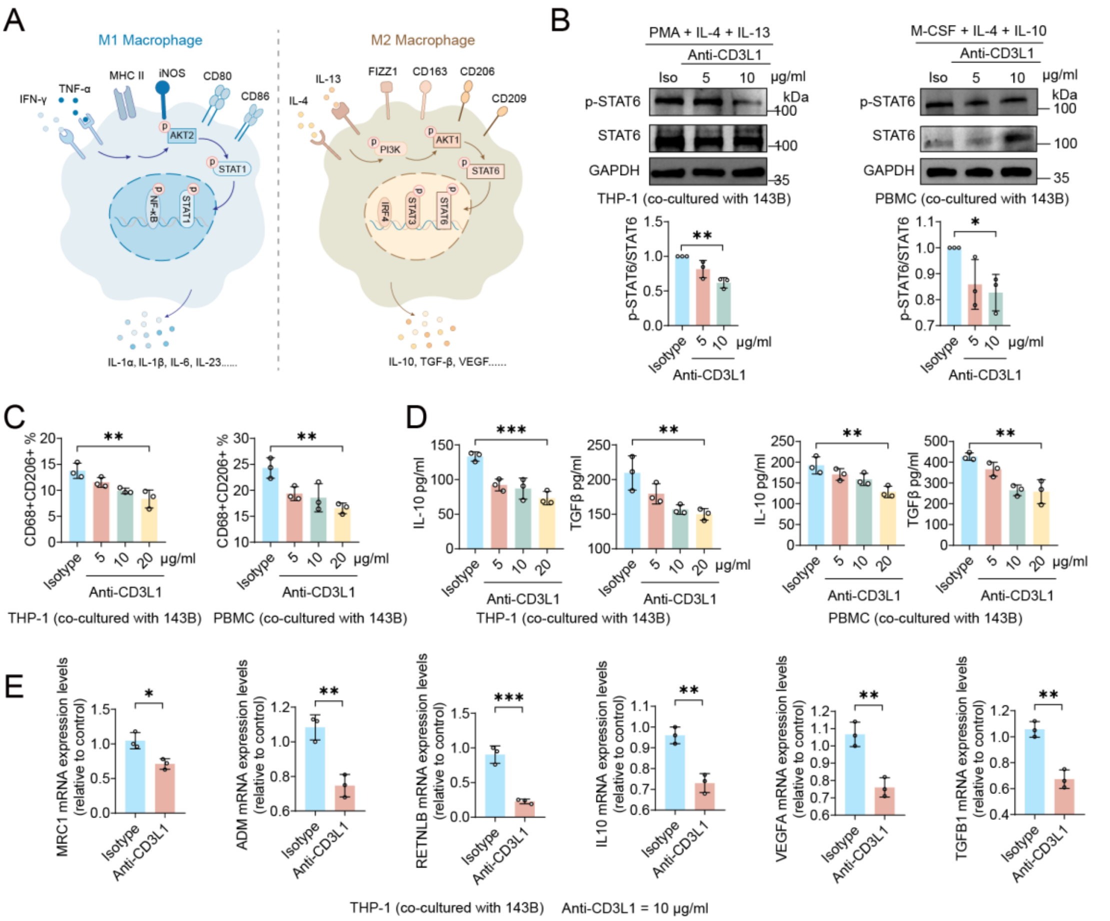

(We have changed the *in vitro* system into a tumor cells - macrophages co-culture system).

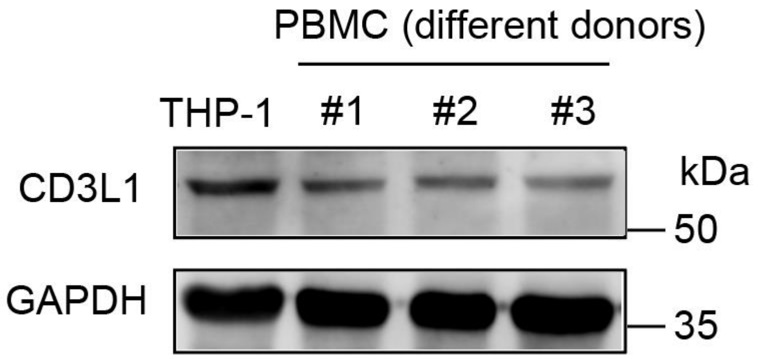

(The expression of CD3L1 in PBMCs were minimal).

• While the current ex vivo T cell activation assay illustrated in Fig. 3F suggests an enhanced T cell activation by anti-CD3L1 treated BMDMs, the experimental set-up does not allow any conclusions to be drawn about the exact mechanism.

A comprehensive investigation on the activation and maturation state of the anti-CD3L1 treated BMDMs combined with a functional T cell profiling (including cytokine production and proliferation) would be needed to evaluate the capabilities of these BMDMs to act as

antigen-presenting cells.

Did the investigators also attempt to determine activation of CD4 T cells?

We sincerely appreciate your insightful comments. Following your suggestions, we have reperformed the experiment utilizing T cells isolated from OT-I transgenic mice, which serve as a representative model for $CD8^+$ T cells, and OT-II transgenic mice, representative of $CD4^+$ T cells. In this revised experimental model, we employed a comprehensive approach to evaluate the functional characteristics of these T cells. Specifically, we utilized flow cytometry to assess the expression levels of activation markers of T cells, including CD25 and CD69. Additionally, we employed the CCK-8 staining assay to generate proliferation curves for the T cells, enabling us to accurately monitor their growth dynamics over time. Furthermore, we measured cytokine production, focusing on IFNG and IL-2 secreted by OT-II T cells, which offered valuable insights into their functional activity (**New Fig.3F-I**). We sincerely believe that we have significantly enhanced the quality and robustness of our tests according to your suggestion.

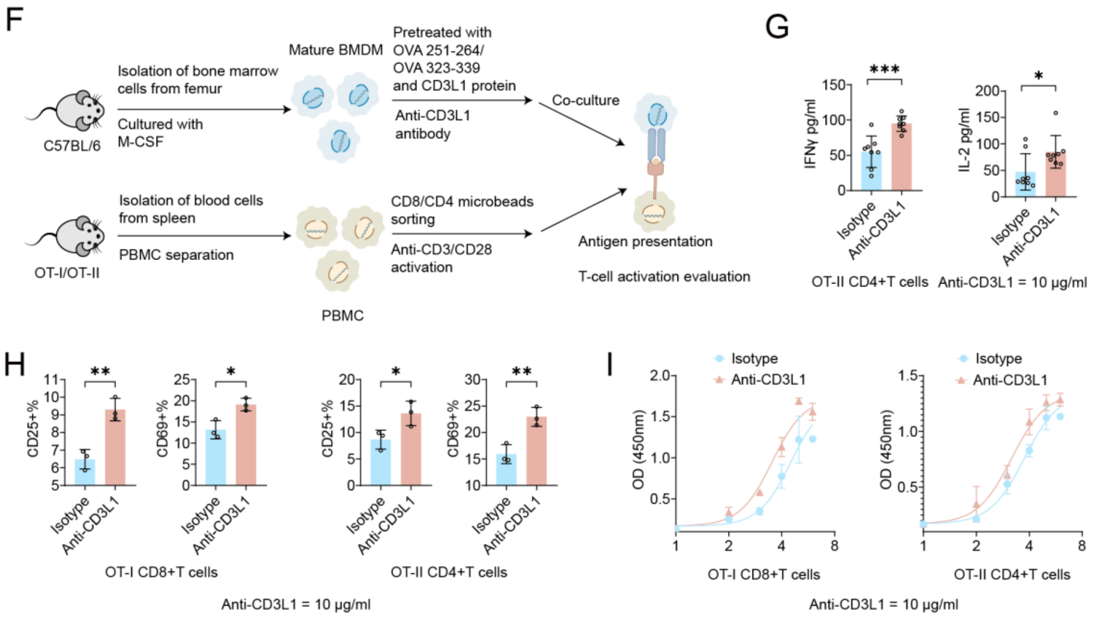

(A more thorough evaluation of the ex vivo T cell activation).

o Binding assays showed an interaction of NRP2 and CD3L1. However, the functional consequence of this interaction remains largely unclear.

Lack of the anti-CD3L1 mediated macrophage re-polarization in the absence of NRP2 supports the hypothesis that CD3L1 acts via the NRP2 axis. However, the absence of an additional effect could also suggest that the lack of NRP2 already elicits the maximal effect. Thus, further experimental data that explore the relevance of this interaction would be needed to strengthen the currently proposed mechanism of action in macrophages.

We extend our sincere gratitude for your constructive suggestions. In accordance with your recommendations, we have conducted a proximity ligation assay (PLA) using HEK293

cells engineered to overexpress either the extracellular domain or the intracellular domain of CD3L1, in conjunction with the extracellular domain of NRP2. The results demonstrate that the extracellular domain, but not the intracellular domain, of CD3L1 binds specifically to the extracellular domain of NRP2, which was consistent with our ELISA result (**New Fig.5C**). We acknowledge the valid concern that knockdown of NRP2 might induce a maximal effect, potentially masking any additional effects conferred by anti-CD3L1. To address this, we designed a series of experiments employing varying concentrations of human NRP2 protein as a competitive binder to NRP2 in the presence of anti-CD3L1. The results of these experiments revealed a significant rescue effect, providing compelling evidence that the influence of anti-CD3L1 on macrophages is indeed contingent upon the CD3L1-NRP2 axis. We believe that these experimental approaches have significantly enhanced the robustness and reliability of our conclusions (**New Fig.5D-G**).

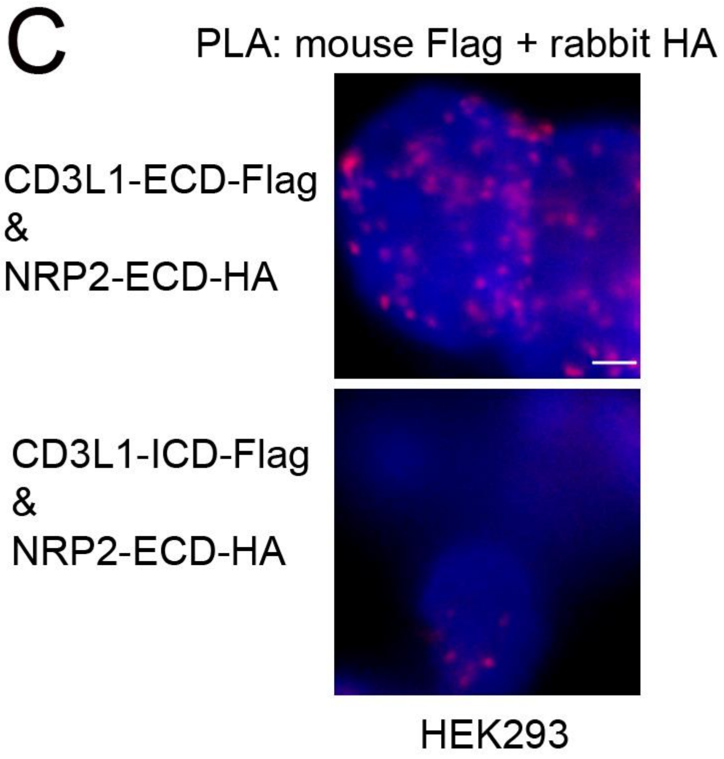

(PLA showing that the ECD but not the ICD of CD3L1 binds to NRP2).

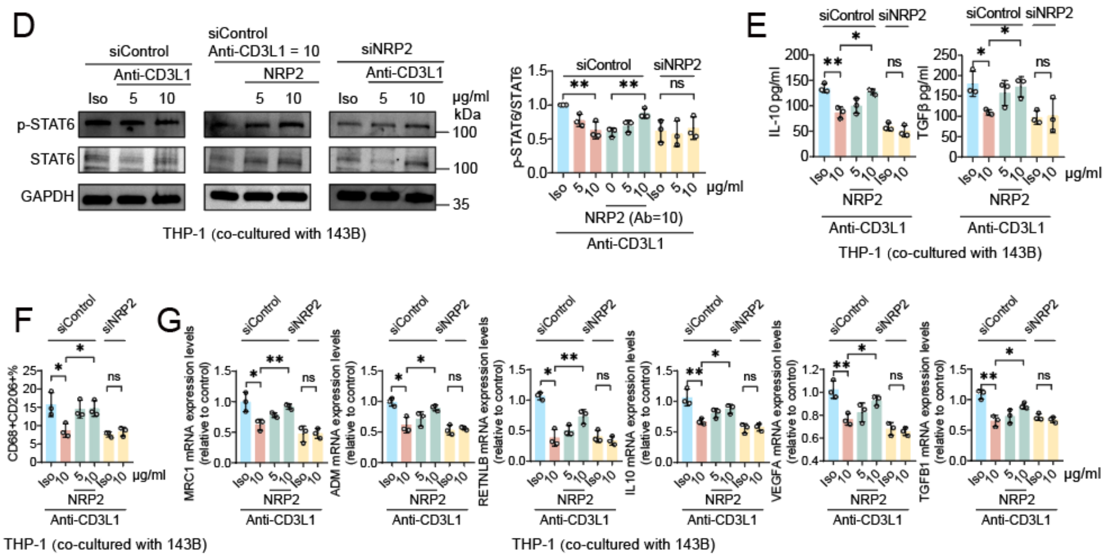

(NRP2 protein exhibits a rescue effect).

o While several data of bulk RNA sequencing analysis from publicly available data sets (e.g. Figure 2K-M) are provided in the manuscript, there is no description of how this analysis was performed. To guarantee transparency and reproducibility this needs to be added.

We sincerely appreciate your valuable feedback, which has been most helpful enhancing the rigor and transparency of our research. In response to your comments, we have now incorporated a comprehensive statistical analysis of the data, with detailed descriptions provided in the methodological section titled "Public Database Analysis." This section outlines the specific methodologies employed for the statistical analysis of the public databases, ensuring clarity and reproducibility of our analytical procedures. We are grateful for your constructive input that facilitated this enhancement.

Public database analysis

The selection standards of the data were in accordance with each cohort dataset.

For Figure 2K-2N, we investigated the relationships between CD3L1 and macrophage infiltration or IL10/TGFB1 mRNA expression levels through the following procedures:

Correlation Analysis: 1) Spearman's rank correlation coefficient: Rank transformation: convert the raw mRNA expression values for each gene into ranks, with ties handled by assigning the average rank; Difference calculation: for each paired observation, calculate the difference in ranks between the two genes; Correlation coefficient calculation: compute Spearman's ρ using the formula based on the sum of squared rank differences; Significance testing: assess the significance of ρ using a t-test with n-2 degrees of freedom, where n is the number of paired observations, to determine if the observed correlation is significantly different from zero. 2) Pearson's correlation coefficient: covariance and standard deviations: calculate the covariance between the mRNA expression levels of the two genes and their respective standard deviations; Correlation coefficient calculation: compute Pearson's r as the covariance divided by the product of the standard deviations; Significance testing: use a t-test to evaluate the significance of r to test the null hypothesis of no linear association.

Linear Regression Analysis: Specify the linear regression model as $y = mx + b + \varepsilon$, where y is the dependent variable (e.g., IL10 or TGFB1 mRNA expression), x is the independent variable (CD3L1 mRNA expression), m is the slope, b is the intercept, and ε is the error term. Use the ordinary least squares method to estimate the slope (m) and intercept (b) by minimizing the sum of squared residuals between the observed and predicted values of y. Calculate the coefficient of determination (R^2) to quantify the proportion of variance in the dependent variable explained by the independent variable. The co-occurance/mutual exclusion was determined based upon the slope (m).

o The relative expression level based on the single cell data re-analysis shown in Figure 2J seems to be rather marginal. Statistical analysis should be provided to support the identification of the main target cell population.

We appreciate your insightful comments. The data sourced from the public database primarily serve to delineate the relative expression patterns of CD3L1 across various cellular subpopulations within the tumor microenvironment (TME) of osteosarcoma. Notably, in certain cellular subsets, CD3L1 exhibits low or negligible expression levels, which may initially suggest limited biological significance. However, it is crucial to clarify that the primary objective of this particular panel (**Fig. 2J**) is to visually represent the relative abundance of CD3L1 within the osteosarcoma TME, rather than to conduct direct, quantitative comparative analyses among distinct cell populations. To enhance the clarity and scientific rigor of our manuscript, we have undertaken a thorough revision of our main

text description. Specifically, we have refrained from making claims of "upregulated expression" in specific cellular subsets into characterizing the relative CD3L1 expression profiles across the heterogeneous cell populations present in the osteosarcoma TME. This revised approach ensures that our interpretations remain consistent with the exploratory nature of the data and avoids any potential overstatements regarding differential regulation, and we sincerely incerely appreciate your valuable input, which has helped us to reinforce the importance of precise interpretation of exploratory data.

available single-cell sequencing datasets. Our analysis revealed the wide expression of CD3L1 in TME, including cancer cells, exhausted T cells, and TAMs within the TME of osteosarcoma and several other advanced solid malignancies (**Fig.2H-J, Fig.EV2E-J**). The spatial overexpression of CD3L1 in the TME provided further evidence of its potential correlation with tumor-promoting functional activities.

Minor points:

o While in the text an elevated expression level of CD3L1, limited T cell infiltration and a crucial role of TAMs in osteosarcoma TME has been highlighted in the first results paragraph, there is at that point of the manuscript no data provided that supports this statement. Therefore, corresponding references should be provided.

Thanks for your suggestions. We have now provided corresponding references in the main text to support our claim.

Given the widely elevated expression levels of CD3L1, the constrained infiltration of T-cells, and the notable significance of TAMs within the osteosarcoma TME, and the clinical efficacy, we selected osteosarcoma as the primary tumor model for investigating the correlation between CD3L1 and TAMs (22-26). Upon revisiting the osteosarcoma mouse models (143B/KHOS tumor cell lines implanted in

o Although reference was made to the previous publication, this manuscript should also specify how the humanized mouse model was set-up for use in osteosarcoma model in Fig1 D.

Thank you for your concerns. We have now incorporated the details of the osteosarcoma model building in Fig.1D in the methodological part for transparency.

Humanized PBMC anti-CD3L1 treatment mouse models

All animal experiments were performed in strict accordance with the relevant ethical guidelines, approved by the department of lab oratory animal science of Fudan University and the Institutional Animal Care. After one week of adaptation to the environment, each individual NPSG mouse (female, 5 weeks old) was randomized into two groups (n=6-8 for each group) and injected 1.8x10^6 pre-treated KHOS/143B cells with 6.0 x 10^5 PBMCs subcutaneously in the right flank. Since Day7 for the tumors after inoculation, the tumor sizes were recorded every 2-3 days using a vernier caliper and calculated with the formula 1/2 x A x a^2 (A and a, respectively, denote the length and the width of the tumor). We treated one group of the mice models with 10mg/kg human IgG1 isotype control and the other with anti-CD3L1 from the measurement day every three days, four times in total. In accordance with the ethical guidelines, mice would be sacrificed once the tumor volume reached 2cm^3 or ulcers happened. In this experiment, all the mice were sacrificed simultaneously when the average tumor volume of control group approached 2cm^3 and the tumors were resected and weighed, and pre-treated for further experiments.

o The description of Figure 2C is missing in the corresponding Figure Legend

We sincerely apologize for our initial neglect, and have provided the description of Fig. 2C in our revised manuscript.

Figure 2 scRNA reveals the negative correlation between anti-CD3L1 treatment and M2 TAMs in the TME.

(A-B) Single-cell RNA sequencing (scRNA seq) data of (**A**) Uniform manifold approximation and proximation (UMAP) plots showing the clustering of (**B**) Pie chart showing the differences in the proportion of $CD45^+$ cells infiltrated in the TME of osteosarcoma samples from nude mice (n=3 independent samples per group).

(C) A dot plot illustrating the expression profiles of various macrophage-related genes across different macrophage subsets in the context of Anti-CD3L1 and Control IgG treatment conditions (n=3 independent samples per group). The color gradient represents the average expression level of each gene, with red indicating higher expression and blue indicating lower expression. The size of each dot corresponds to the percentage of cells expressing the respective gene (25%, 50%, or 75%).

o In the text the Figure 3C and D, are wrongly assigned.

We sincerely apologize for the mistake, now we have assigned them in the right order.

o The correction for multiple testing for certain statistical tests should be indicated.

Thanks for the suggestion. We have now provided the correction for multiple testing for all

of the needed statistical tests in the methodological part.

To address multiple testing, we applied the Benjamini-Hochberg procedure to control the false discovery rate (FDR) at 5%. Significance was declared for tests with adjusted p-values ≤ 0.05, balancing Type I and Type II errors.

Referee #2 (Comments on Novelty/Model System for Author):

This is a strong and conceptually interesting follow-up to the first study, but the mechanistic discrepancy between the A375 and osteosarcoma models, and the reliance on xenografts, should be addressed directly. Demonstrating that macrophage reprogramming occurs in syngeneic models or explaining its absence in A375 tumors is in my view critical to reconcile the group's two papers and to establish CD3L1 as a dual- (inate and adaptive) targeting immune modulator.

Referee #2 (Remarks for Author):

Summary

This manuscript extends on the group's previous study published in Cell (Deng et al., 2024) that described CD3L1 as a ligand of CD3ε and that showed by binding to CD3 it is suppressing T-cell activation. This study proposes a new and distinct, T-cell independent macrophage mechanism of immune suppression- suggesting that CD3L1 also interacts with Neuropilin-2 on tumor-associated macrophages (TAMs), driving macrophage into a different type of polarization. Their data suggests that their anti-CD3L1 antibody treatment blocks interaction with NRP2 and thereby reprograms TAMs toward an M1 phenotype, and inhibits tumor growth in T-cell-deficient osteosarcoma xenografts. Unsing single-cell RNA-seq, flow cytometry, and IHC analyses the authors suggest reduced M2 and increased M1 polarized TAMs after treatment. This study thus proposes a conceptual extension of the earlier study by shifting or extending the inhibitory activity from adaptive immunity - blocking T-cell activation, to innate immunity, changing macrophage polarization.

The strength of this study is that in proposes a plausible dual mechanism for their novel checkpoint inhibitor, CD3L1 (that they have patented for immunothereapy of cancer) in not only suppressing T cells (via binding CD3ε) but also modulating macrophages via binding to NRP2. The study presents coherent in-vitro and in-vivo data in which they try to link anti-CD3L1 blockade to TAM reprogramming and tumor inhibition in a xenogenic osteosarcoma model.

We are profoundly grateful for your insightful comments. Following your constructive suggestions, we have gathered more compelling evidence to substantiate our conclusion. Additionally, we have carried out an extensive revision of the entire manuscript to enhance the robustness of our findings and improve overall clarity.

Major critique/concerns

1) Why was the contribution of "repolarizing" the myeloid cells not visible in their initial

paper for the xenograft A375 tumor model after T cell depletion? In the Cell study, T cell depletion abolished anti-CD3L1 efficacy in A375 xenografts, implying a strick T-cell-dependent mechanism. Yet in the current study, anti-CD3L1 retains efficacy in T-cell-deficient nude mice. Therefore, why was no macrophage-mediated effect observed in the earlier A375 model? Do A375 tumors lack a significant myeloid compartment, or are their macrophages refractory to M1 conversion by CD3L1 blockade?

We sincerely appreciate your insightful concerns. Upon re-evaluating our A375 models, we conducted IHC staining of tumor samples using CD68 as a marker of macrophages. Our findings indicate that following the depletion of $CD4^{+}$ and $CD8^{+}$ T cells in PBMC-reconstituted NPSG mice, there was a significant reduction in the infiltration of functional macrophages (**New Fig.EV6C**). This phenomenon may be attributed to the limitations of immune reconstitution in accurately replicating the natural clinical microenvironment. Specifically, given the intricate interplay between T cells and macrophages within the tumor microenvironment, the exclusion of T cells may compromise macrophage survival. We posit that this interdependence explains the absence of observable macrophage contributions in our previous A375 models. The intriguing correlation between T cells and macrophages in the TME warrants further investigation to elucidate the underlying mechanisms and potential therapeutic implications.

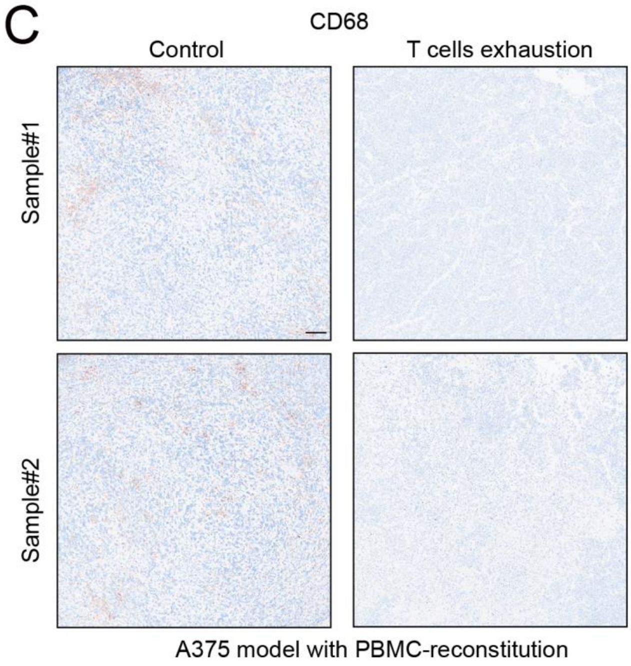

(IHC staining showing that after T cells exhaustion, the macrophage infiltration was significantly diminished in the TME of A375 model)

2) Concerning the choice of model why was a nude xenograft model used to test their hypothesis in-vivo instead of the earlier used syngeneic models? The authors use human osteosarcoma xenografts in nude mice to demonstrate T-cell independent activity.

However, syngeneic murine models such as the B16 melanoma or the MC38 colorectal cancer models already used in the earlier study would have permitted controlled T-cell depletion or genetic NRP2 manipulation within a fully immunocompetent microenvironment and would have provided a realistic and relevant approximation of the T cell vs. inate contribution to the immunosupressive activity of CD3L1. Why not use these models to quantify the relative contributions of T cells and myeloid cells, instead of relying on correlative scRNA-seq data from xenografts?

Thank you for your insightful comments and suggestions regarding the choice of our in-vivo model. We appreciate your recognition of the value of manipulating specific cell populations to observe their functions and impacts, as well as the potential of NRP2 manipulation in syngeneic models. You are absolutely right that syngeneic murine models, offering the advantage of allowing controlled T-cell depletion or genetic NRP2 manipulation within a fully immunocompetent microenvironment. This would indeed provide a more realistic and relevant approximation for dissecting the contributions of T cells versus innate cells to the immunosuppressive activity of CD3L1. Regarding our choice of the nude xenograft model, we would like to clarify our rationale as follows:

Firstly, the nude mouse model is naturally deficient in T cells while retaining myeloid immune components. This inherent characteristic allows us to directly assess the role of CD3L1 in the absence of T cells without the need to artificially alter the cellular composition of the immune microenvironment. It provides a straightforward approach to investigate T-cell-independent activity, which was a key aspect of our hypothesis. We have provided this statement in our revised manuscript.

> analyzed the TME of osteosarcoma transplanted in nude mice. This approach was taken to ensure that any observed effects were not confounded by potential T-lymphocyte interference, considering the nude mouse model was naturally deficient in T cells while retaining myeloid immune components, which allowed us to directly assess the role of CD3L1 in the absence of T cells without the need to artificially alter the cellular composition of the immune microenvironment (25, 26). After treatment by 10 mg/kg

Secondly, as we have observed in previous models, specific depletion of one cell type in murine immune modeling may inadvertently affect the number and function of other cell populations due to multiple confounding factors, just as you as can see in our response to major concern #1. This could complicate the interpretation of results and introduce uncertainties in attributing observed effects to the targeted cell population. The nude model, by virtue of its natural T-cell deficiency, circumvents this issue to some extent.

Lastly, we acknowledge that NRP2 manipulation is a valuable proposition. However, considering the costs and time required for generating and validating NRP2 knockout mice and the preservation of the genetic background in the following experiments, we were unable to incorporate this experiment into our current study. We believe that our current

model, despite its limitations, is sufficient to support our present hypothesis. Nevertheless, we completely agree that further investigation using syngeneic models with targeted cell depletion or genetic manipulation, as you suggested, would be highly beneficial. We plan to pursue such experiments in future studies to gain a more comprehensive understanding of the roles of different immune cell populations in CD3L1-mediated immunosuppression. Thank you again for your constructive feedback, which has inspired us to consider more in-depth research directions for our future work.

3. The evidence for CD3L1-NRP2 binding is based primarily on ELISA. An independent confirmation e.g. by co-immunoprecipitation or proximity ligation in primary macrophages would have strengthened their claim and thus the central mechanism proposed.

Thank you for your insightful suggestions. Considering that the tumor-cell co-culture model may lack sufficient simplicity to effectively elucidate the interaction between CD3L1 and NRP2, we have conducted a proximity ligation assay (PLA) using HEK293 cells engineered to overexpress either the extracellular domain or the intracellular domain of CD3L1, in conjunction with the extracellular domain of NRP2 (**New Fig.5C**). The results demonstrate that the extracellular domain, but not the intracellular domain, of CD3L1 binds specifically to the extracellular domain of NRP2, which was consistent with our ELISA results. We sincerely appreciate your concerns, which helped us further strenthen the evidence of CD3L1-NRP2 binding.

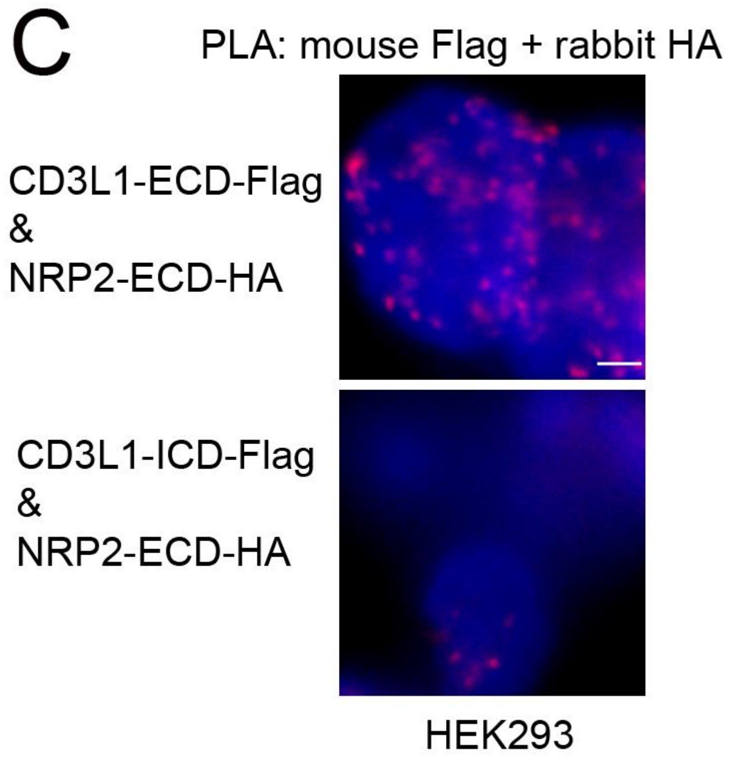

(PLA showing that the ECD but not the ICD of CD3L1 binds to NRP2).

Minor points

It would be interesting to clarify whether the antibody's effects depend on Fc receptor binding or whether a ligand blockade with Fab- Fragments is sufficient.

Thank you for your insightful and constructive feedback. It is indeed of significant scientific interest and necessity to ascertain whether the observed effects of the antibody are dependent on Fc receptor binding or if ligand blockade mediated by Fab fragments alone is sufficient. In response to this valuable query, we have conducted additional experiments using Fab fragments derived from anti-CD3L1 (sourced from Biotroy Therapeutics, which lack the Fc region). These experiments were designed to evaluate the specific effects attributable to anti-CD3L1 in the absence of Fc receptor engagement, thereby providing a clearer understanding of the antibody's mechanism of action (**New Fig.4**).

Figure 4

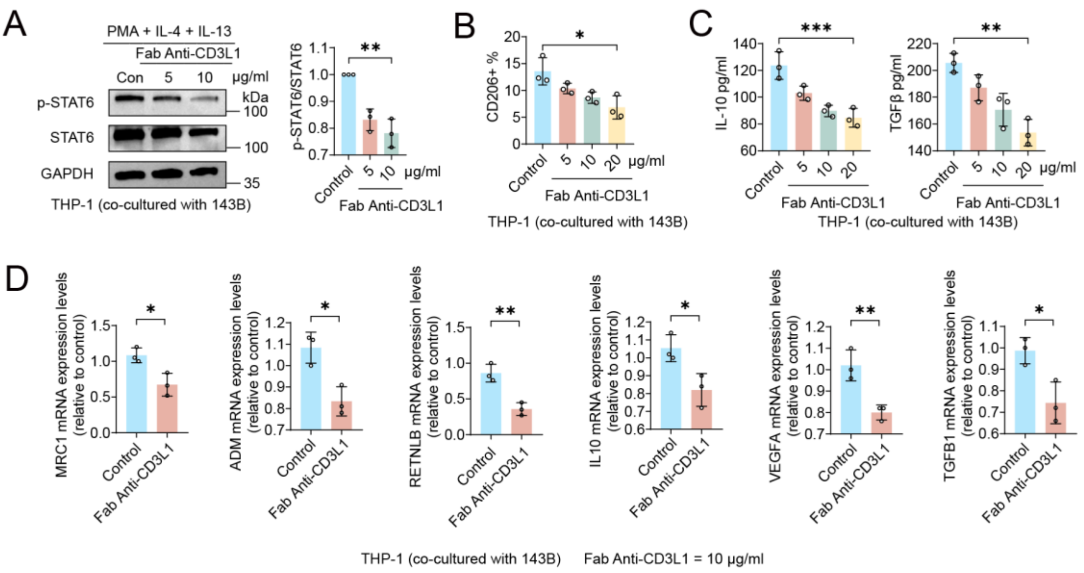

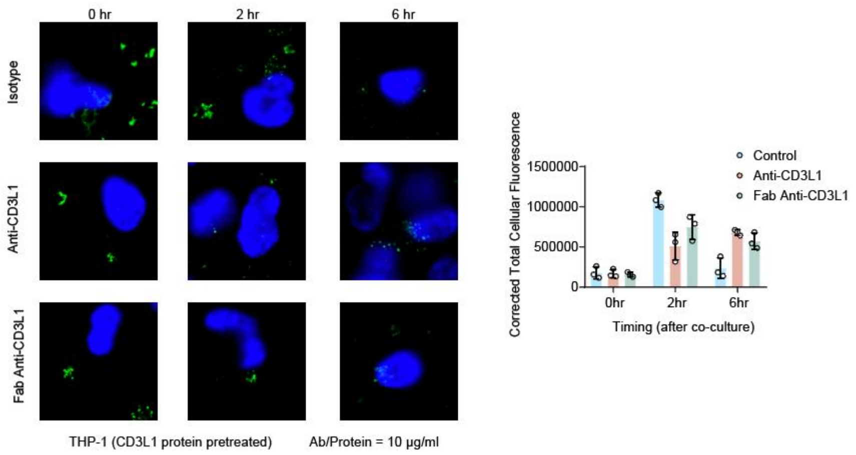

(The results of Fab Anti-CD3L1 treatment were comparable to the results treated by anti-

CD3L1)

It might be also interesting to analyze NRP2 expression patterns in osteosarcoma versus melanoma.

We appreciate your valuable comments. We have subsequently conducted immunofluorescence (IF) staining for NRP2 in both 143B (osteosarcoma) and A375 (melanoma) samples (**New Fig.EV6D**). The results from the microscopic examination of the stained slides indicate that there is no statistically significant difference in NRP2 expression between these two sample types, in terms of both expression level and distribution pattern. However, given the potential disparities in spatial transcriptomic expression patterns, which may unveil more nuanced regulatory mechanisms, further in-depth investigation is warranted to elucidate these aspects.

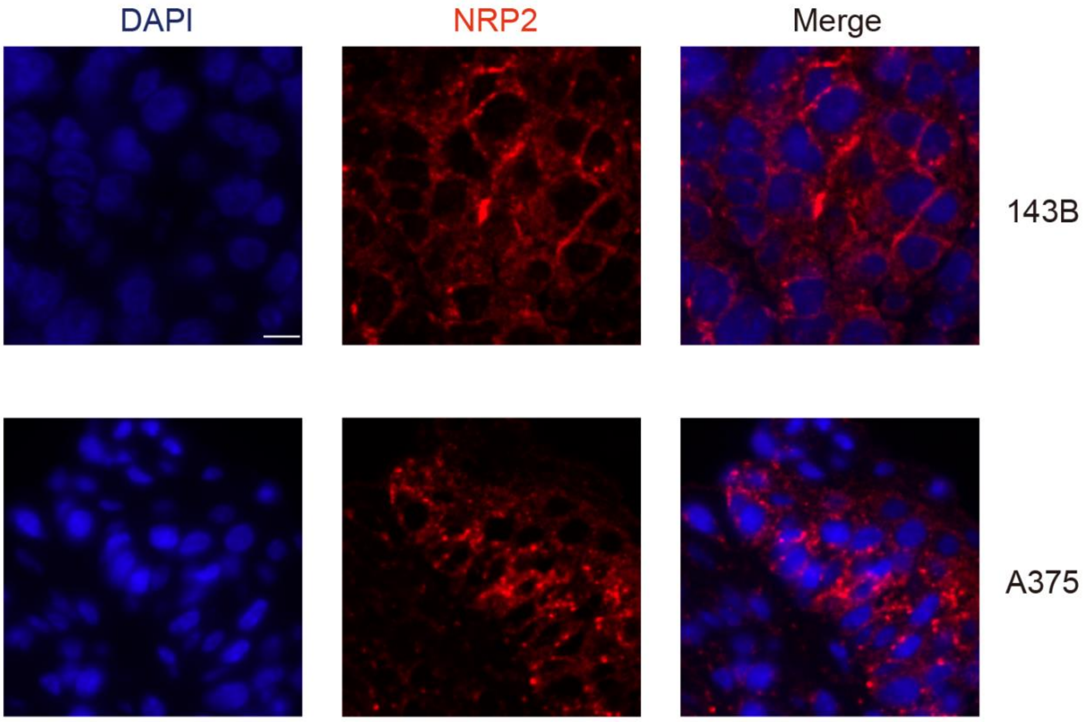

(The expression of NRP2 in the melanoma and osteosarcoma TME).

In my view in the text it should be more explicitly explained that the observation stems from a single melanoma patient case (anecdotal).

We sincerely appreciate your thoughtful concerns. Indeed, the phenomenon in question has been documented in a substantial number of patients exhibiting a response, with notable alterations in CD68 expression and macrophage infiltration. We acknowledge the oversight in our initial manuscript for not explicitly addressing this critical point. To rectify this, we have thoroughly revised the main text to enhance the clarity and precision of our arguments and findings.

The overall design of the entire study is illustrated in **Fig.1A**. In our finished Phase Ia clinical trial, we profiled tumor microenvironments using multiplex immunofluorescence and immunohistochemical analysis of immune cell markers. While anti-CD3L1 treatment induced the expected enhancement of T cell infiltration, we strikingly observed concurrent macrophage accumulation specifically in several treatment-responsive tumors (**Fig.1B-C**). Notably, in a representative case from a PD-1 refractory melanoma patient, a single 8 mg/kg intravenous dose of anti-CD3L1 elicited robust increases in both T cell and macrophage infiltration and functional activation within the TME.

Overall assesment

This is a strong and conceptually interesting follow-up to the first study, but the mechanistic discrepancy between the A375 and osteosarcoma models, and the reliance on xenografts, should be addressed directly. Demonstrating that macrophage reprogramming occurs in syngeneic models or explaining its absence in A375 tumors is in my view critical to reconcile the group's two papers and to establish CD3L1 as a dual- (inate and adaptive) targeting immune modulator.

Thank you once again for your thoughtful and valuable evaluation. We truly appreciate your time and constructive input.

Referee #3 (Comments on Novelty/Model System for Author):

The strategy employed to identify/describe the effect of anti-CD3L1 on macrophages is solid. The differences in the control and anti-CD3L1 treated samples (clinical and pre-clinical) are apparent and interesting. Overall the descriptive data (Fig 1 & 2) is adequate. The novelty is high: a) working with a clinical stage drug/drug target; b) ID of a potential new ligand for an apparent ICB target. The medical impact is medium due to challenges listed below with the model system. The model system to work out the biology of how CD3L1 impacts the immune microenvironment is unclear.
Challenges: 1) the authors point out in the summary figure and in the text that CD3L1 is likely mainly expressed on tumor cells. No effort is made to demonstrate that tumor cell CD3L1 signals in tumor cells or that tumor cell expression of CD3L1 is required for the biology observed or the efficacy of the anti-CD3L1. 2) The CD3L1 signaling studies are performed in THP-1 (myeloid). The authors propose that CD3L1 on tumor cells or potential other cells interacts with Nrp2 on macrophages and this drives the M2-like phenotype. However, there are no studies demonstrating that Nrp2 signals, how it signals. They only look at siNrp2. Further the studies are in vitro and without tumor cells. I do not think the models employed are adequate to make the conclusions drawn, this impacts the medical impact and the technical quality.

Referee #3 (Remarks for Author):

Deng et al investigate the effect of anti-CD3L1 on the tumor immune microenvironment. Anti-CD3L1 are in clinical trial and the authors investigated the effect of ant-CD3L1 on macrophage infiltration and marker expression in clinical samples and preclinical tumor models. Interestingly, treatment of anti-CD3L1 resulted in an increase in M1-like macrophages and a decrease in macrophages with a M2-like phenotype. They show this by IHC and omics techniques. These data are convincing. The authors suggest that CD3L1 is expressed by tumor cells and other cells, including macrophages in tumors. They show in vitro treatment of M2-polarized THP-1 cells with anti-CD3L1 results in reduction of pSTAT6 and expression of cytokines are other M2-like markers and an increase in the APC capacity of BMDMs. They investigate potential CD3L1 binding partners via biochemistry with purified proteins from a candidate list and find that Nrp2 binds to CD3L1. This is interesting. They provide data that anti-CD3L1 blocks Nrp2-CD3L1 interaction. Overall it is an interesting study that has some strong points, namely it is centered around a clinical stage antibody and they have identified a putative new ligand/interactor for CD3L1.

We extend our heartfelt gratitude for your insightful comments. Based on your valuable suggestions, we have thoroughly revised our manuscript and incorporated more compelling evidence to bolster our conclusion. We sincerely believe that we have improved

our manuscript through your constructive suggestions.

Comments:

1. There is a disconnect between where CD3L1 is expressed and how it impacts macrophage phenotype. Is CD3L1 signaling on macrophages relevant or is it Nrp2 on macrophages that is signaling. In either case, how, what is driving the signaling. If as Fig 3 and 4 suggest CD3L1 on macrophages is signaling, what is driving that signal? Is it Nrp2 on the same cell, on adjacent macrophages, on other cells? The story was setup to suggest that CD3L1 on tumor cells was interacting with a partner on macrophages but that is not how it ended.

We deeply appreciate your observation and constructive suggestion. We completely agree with your insightful enlightenment, which has prompted us to critically re-evaluate our experimental design and data interpretation. Following your guidance, we have redesigned all *in vitro* experiments using a co-culture system of tumor cells (expressing CD3L1) and induced macrophages (expressing NRP2), as this model more accurately reflects the proposed biological mechanism of CD3L1-NRP2 crosstalk in the TME. Our initial approach, which utilized THP-1-derived macrophages only, was based on the assumption that these cells could serve as a simplified system for cells expressing CD3L1 and macrophages expressing NRP2, considering their CD3L1-expressing nature. However, we now recognize that this system fails to adequately recapitulate the physiological interactions between tumor cells and TAMs, particularly regarding CD3L1-NRP2-mediated signaling pathways. THP-1 cells are recognized as tumor cells, whereas CD3L1 exhibits a propensity for high expression in malignant cells, serving as a mechanism for immune evasion. This pattern is analogous to the observation that normal lung tissues do not express CD3L1, whereas lung cancer tissues overexpress CD3L1 (our research work published in 2023, Frontiers in Cell and Developmental Biology). We deeply regret any confusion caused by our initial oversight and have removed all data derived from the initial system from the revised manuscript. We sincerely appreciate you again for highlighting these critical issues, which have significantly strengthened the scientific rigor of our study.

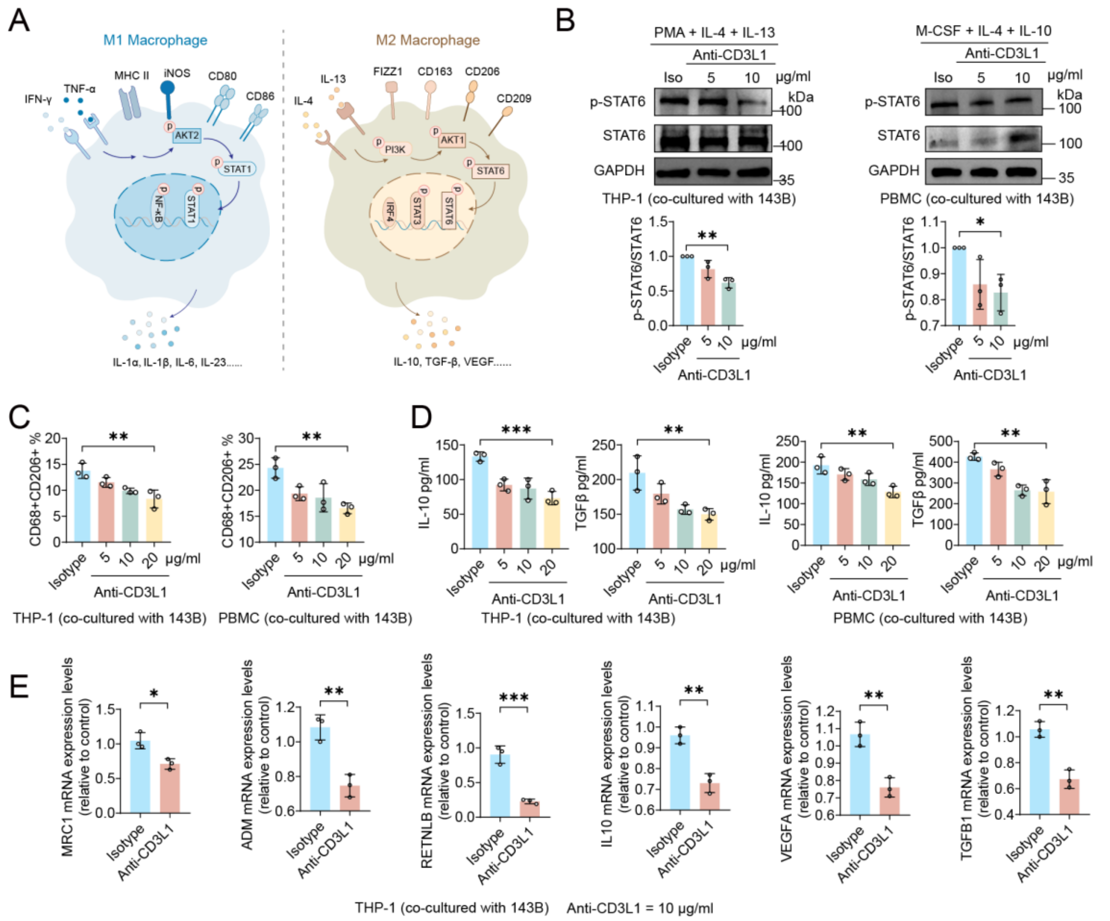

(We have changed the *in vitro* system into a tumor cells - macrophages co-culture system).

2. The importance of Nrp2 as a CD3L1 binder to the biology of anti-CD3L1 (the change in macrophage phenotype) is not clear based on the data shown. Demonstrate that Nrp2 expression on macrophages (if that is where you think it is important) matters to the phenotype in vivo.

We extend our sincere gratitude for your insightful comment. As documented in prior research, the expression of NRP2 across distinct macrophage phenotypes has been previously reported (Roy et al., Cancer Res, 2018). In alignment with these findings, we have conducted an assessment of NRP2 expression at various stages of macrophage differentiation and polarization (i.e., control macrophages, those treated with PMA, and those subsequently treated with PMA followed by IL-4/IL-13 for different durations) (**New Fig.EV5B**). Our results reveal evident differences in NRP2 expression that are consistent with the patterns reported in earlier studies.

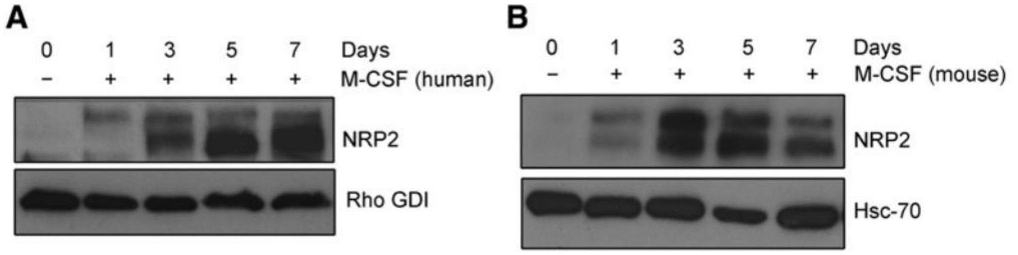

Figure 1.
Expression of NRP2 in macrophages. **A** and **B,** NRP2 expression in freshly isolated human monocytes and bone marrow cells from C57BL/6 mice differentiated to macrophages with M-CSF. **C,** Representative confocal image showing the presence of NRP2-expressing $CD68^+$ TAMs in human PDAC tissue.

Roy et al., Cancer Res, 2018.

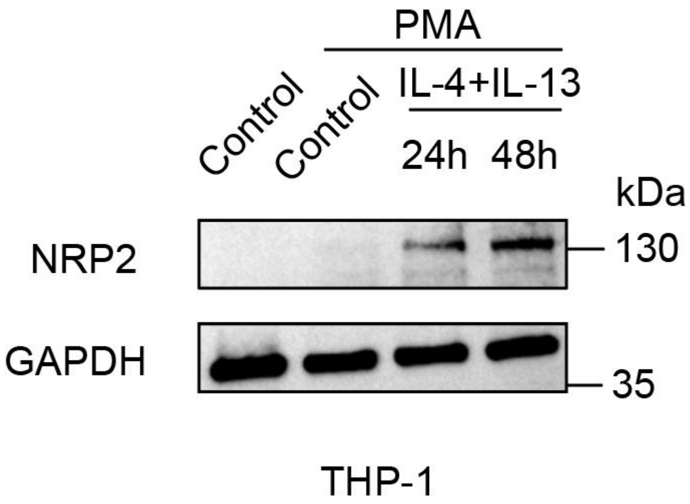

(Our results showing the expression of NRP2 on different stages of induced macrophages)

3. The efferocytosis assays in Figure 3 and in supplement need clarification. Is the engulfment of apoptotic Jurkat cells Fc-dependent? Does anti-CD3L1 bind apoptotic Jurkat cells? Is the anti-CD3L1 still in the system when the target cell (Jurkat) added to the macrophages?

We deeply appreciate your thoughtful concerns. Regarding the engulfment of apoptotic Jurkat cells, we have re-executed the experiment under the tumor cell co-culture conditions. Our results demonstrate that there is no statistically significant difference in the engulfment rate between the group treated with anti-CD3L1 and the group treated with Fab fragment proteins derived from anti-CD3L1 (**new Fig.EV4C**). As established in our prior study, we have assessed the expression of CD3L1 on Jurkat cells, and theoretically, anti-CD3L1 should bind to these cells. However, our new findings indicate that, although anti-CD3L1 binds to normal Jurkat cells, following apoptosis, anti-CD3L1 no longer exhibits binding affinity to the apoptotic cells (**new Fig.EV4D**). We hypothesize that this phenomenon may be attributed to the specific epitope recognized by the anti-CD3L1 antibody, which binds to the “RLLEMEFEERKRAAE” sequence (shown in our previous study, CELL, 2024) within the extracellular domain of the CD3L1 protein. Apoptosis may induce conformational

changes or degradation that result in the loss of this binding affinity. According to our experimental protocol, anti-CD3L1 antibodies remained present in the system when target cells were introduced to macrophages; nevertheless, these antibodies did not bind to the apoptotic Jurkat cells. Furthermore, the use of anti-CD3L1 Fab proteins suggests that the observed effects are not primarily Fc-dependent. To enhance clarity and reproducibility, we have provided additional methodological details in the revised manuscript.

Efferocytosis detection

Apoptosis was induced in Jurkat cells through the application of 50 mmol/L etoposide, with the cells being maintained at 37°C，5% CO2 for a duration of 12 hours. This treatment protocol typically resulted in the induction of apoptosis in 80% to 90% of the cells. Subsequently, the cells were centrifuged at 2,000 rpm for 10 minutes, and the cell pellet was washed twice with PBS. The cells were then incubated with pHrodo Green Maleimide, adhering strictly to the manufacturer's instructions. Following incubation, the cells were once again centrifuged at 2,000 rpm for 10 minutes. The pellet was washed twice with PBS and resuspended in RPMI-1640 medium. In parallel, THP-1 cells, which had been induced as described previously, were plated in eight-well chambers with 10μg/ml CD3L1 protein. Apoptotic Jurkat cells were then added to the macrophages at a target-to-effector ratio of 10:1 for a period of 1 hour (referred to as the pulse phase) at 37°C. Phagocytosis was halted by the addition of ice-cold PBS, and the cells were rigorously washed with ice-cold PBS to eliminate any non-phagocytosed or loosely bound cells. Fresh, complete RPMI-1640 medium was added to the cells, and the clearance of apoptotic cells was monitored over the specified time period (referred to as the chase phase). The slides were then sealed with DAPI and analyzed using confocal microscopy. The images were analyzed by ImageJ for the corrected total cellular fluorescence.

(More clarified methodological description)

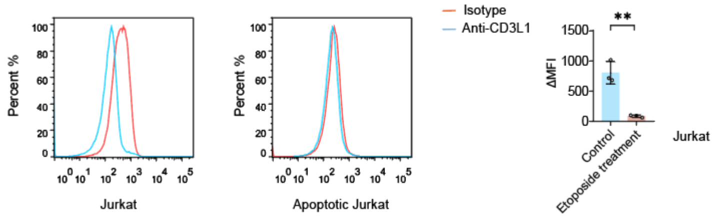

(Apoptotic Jurkat cells no longer binded to anti-CD3L1)

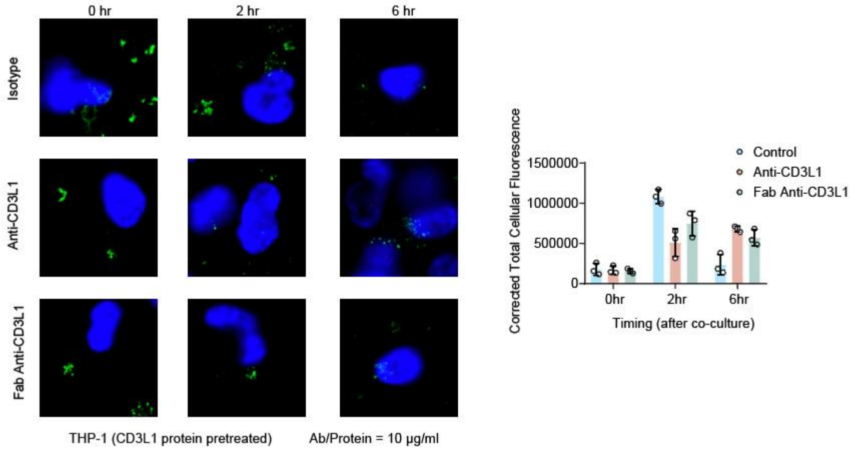

(The results of Fab Anti-CD3L1 treatment were comparable to the results treated by anti-CD3L1)

To wrap up, the reviewers have made constructive suggestions, for which we are most grateful. We have made revisions strictly according to these comments with optimized model building and more robust evidence. We have also revised the improper descriptions and unclarified methods with thorough editing to improve the clarity of the whole manuscript.

4th Mar 2026

Dear Dr. Xu,

Thank you for submitting your revised manuscript to EMBO Molecular Medicine, and please accept my apologies for the delay in getting back to you as one referee needed more time to complete their report. We have now received the reports from the 3 initial referees. As you will see below, while referee #3 is satisfied with the revisions, referees #1 and #2 still raise a few concerns regarding the lack of mechanistic insight and the need for more physiologically relevant systems.
EMBO Press usually encourages a single round of experimental revisions, however in this case, given the potential interest of the findings, we are willing to invite you to revise the manuscript further to address the remaining issues. Please be aware that this will be your last opportunity to address these issues. The revised manuscript will be reviewed again, and we cannot guarantee a positive outcome at this stage.

Should you find that the requested revisions are not feasible within the constraints outlined here and prefer, therefore, to submit your paper elsewhere, we would welcome a message to this effect.

I look forward to seeing a revised form of your manuscript as soon as possible.

With kind regards,

Lise Roth

Lise Roth, Ph.D
Senior Editor
EMBO Molecular Medicine

When preparing your revised manuscript, please refer to our guidelines: https://link.springer.com/journal/44321/submission-guidelines#cms-Revised-submissions. We perform an initial quality control of all revised manuscripts before re-review; failure to include requested items will delay the evaluation of your revision.

We require:

1) A .docx formatted version of the manuscript text (including legends for main figures, EV figures and tables). Please make sure that the changes are highlighted to be clearly visible.

2) Individual production quality figure files as .eps, .tif, .jpg (one file per figure). For guidance, download the 'Figure Guide PDF': https://media.springernature.com/original/springer-cms/rest/v1/content/27825798/data/v1.

3) A .docx formatted letter INCLUDING the reviewers' reports and your detailed point-by-point responses to their comments. As part of the EMBO Press transparent editorial process, the point-by-point response is part of the Review Process File (RPF), which will be published alongside your paper.

4) A complete author checklist, which you can download from our author guidelines. Please insert information in the checklist that is also reflected in the manuscript. The completed author checklist will also be part of the RPF.

5) Please note that all corresponding authors are required to supply an ORCID ID for their name upon submission of a revised manuscript.

6) It is mandatory to include a 'Data Availability' section after the Materials and Methods. Before submitting your revision, primary datasets produced in this study need to be deposited in an appropriate public database, and the accession numbers and database listed under 'Data Availability'. Please remember to provide a reviewer password if the datasets are not yet public.

In case you have no data that requires deposition in a public database, please state so in this section. Note that the Data Availability Section is restricted to new primary data that are part of this study.

7) For data quantification: please specify the name of the statistical test used to generate error bars and P values, the number (n) of independent experiments (specify technical or biological replicates) underlying each data point and the test used to

calculate p-values in each figure legend. The figure legends should contain a basic description of n, P and the test applied. Graphs must include a description of the bars and the error bars (s.d., s.e.m.).

8) At EMBO Press we ask authors to provide source data for the main manuscript figures. You will receive a separate email with instructions for providing source data with your revised manuscript, including how to upload and organize the files.

9) Our journal encourages inclusion of *data citations in the reference list* to directly cite datasets that were re-used and obtained from public databases. Data citations in the article text are distinct from normal bibliographical citations and should directly link to the database records from which the data can be accessed. In the main text, data citations are formatted as follows: "Data ref: Smith et al, 2001" or "Data ref: NCBI Sequence Read Archive PRJNA342805, 2017". In the Reference list, data citations must be labeled with "[DATASET]". A data reference must provide the database name, accession number/identifiers and a resolvable link to the landing page from which the data can be accessed at the end of the reference.

10) We replaced Supplementary Information with Expanded View (EV) Figures and Tables that are collapsible/expandable online. A maximum of 5 EV Figures can be typeset. EV Figures should be cited as 'Figure EV1, Figure EV2" etc... in the text and their respective legends should be included in the main text after the legends of regular figures.

- For the figures that you do NOT wish to display as Expanded View figures, they should be bundled together with their legends in a single PDF file called *Appendix*, which should start with a short Table of Content. Appendix figures should be referred to in the main text as: "Appendix Figure S1, Appendix Figure S2" etc.

- Additional Tables/Datasets should be labeled and referred to as Table EV1, Dataset EV1, etc. Legends have to be provided in a separate tab in case of .xls files. Alternatively, the legend can be supplied as a separate text file (README) and zipped together with the Table/Dataset file.

11) The paper explained: EMBO Molecular Medicine articles are accompanied by a summary of the articles to emphasize the major findings in the paper and their medical implications for the non-specialist reader. Please provide a draft summary of your article highlighting
- the medical issue you are addressing,
- the results obtained and
- their clinical impact.

This may be edited to ensure that readers understand the significance and context of the research. Please refer to any of our published articles for an example.

12) Author contributions: You will be asked to provide CRediT (Contributor Role Taxonomy) terms in the submission system. These replace a narrative author contribution section in the manuscript.

13) A Conflict of Interest statement should be provided in the main text.

14) Every published paper includes a 'Synopsis' to further enhance discoverability. Synopses are displayed on the journal webpage and are freely accessible to all readers. They include a short stand first (maximum of 300 characters, including space) as well as 2-5 one-sentences bullet points that summarizes the paper. Please write the bullet points to summarize the key NEW findings. They should be designed to be complementary to the abstract - i.e. not repeat the same text. We encourage inclusion of key acronyms and quantitative information (maximum of 30 words / bullet point). Please use the passive voice. Please attach these in a separate file or send them by email, we will incorporate them accordingly.

Please also suggest a striking image or visual abstract to illustrate your article as a PNG file 550 px wide x 300-600 px high.

15) Include a Reagents and Tools Table as part of the Methods section, which can be downloaded from our author guidelines.

EMBO Molecular Medicine has a "scooping protection" policy, whereby similar findings that are published by others during review or revision are not a criterion for rejection. Should you decide to submit a revised version, I do ask that you get in touch after three months if you have not completed it, to update us on the status.

***** Reviewer's comments *****

Referee #1 (Remarks for Author):

The authors have addressed several of the concerns raised and have strengthened the manuscript accordingly. The revised version improves transparency regarding the experimental procedures, confirms key findings through the inclusion of

complementary technological approaches, demonstrates that the described effect is Fc-independent and incorporates more representative model systems, including selected experiments using a co-culture system with tumor cells and PBMCs. Together, these additions increase confidence in the validity of the central observations.

While the manuscript has improved substantially, there are some remaining mechanistic aspects that are not fully addressed, and certain findings would benefit from further validation in more physiologically relevant systems. Greater clarification of the direct downstream signaling of Nrp2 in macrophages, as well as a more detailed investigation of CD3L1 expression levels on tumor cells and macrophages to better define the relative contribution of cis versus trans signaling and whether both modes of interaction can occur and induce comparable functional effects, would further strengthen the mechanistic framework. Furthermore, the link between the reported immune modulation and tumor control in T cell-deficient settings remain insufficiently explained.

Overall, the manuscript presents interesting and potentially novel findings and proposes a compelling dual mechanism for a promising new ICB agent. The study highlights several novel aspects of this compound, identifies Nrp2 as a binding partner for CD3L1, and provides evidence supporting the relevance of this interaction in driving the observed phenotypic switch in macrophages. A clear limitation of the study is that certain mechanistic aspects remain incompletely resolved and the translational robustness would benefit from broader validation in more physiologically relevant systems. These aspects warrant consideration when interpreting the findings and evaluating their broader applicability.

Referee #2 (Comments on Novelty/Model System for Author):

The revised study presents a potentially novel and conceptually interesting interaction between CD3L1 and NRP2, supported by several additional experiments that have strengthened study compared to the original submission. The in vitro co-culture system as well as the more detailed binding analyses, support the proposed macrophage-directed mechanism.
My remaining concern relates to the reliance on xenogeneic models to dissect T-cell-independent macrophage effects. In my view, such systems introduce inherent biological limitations due to the artificial cross-species microenvironment, which may affect tumor-stroma and immune interactions. While I do not consider it necessary to request additional experiments at this stage, the manuscript should more clearly and explicitly acknowledge these limitations and temper the interpretation accordingly

Referee #2 (Remarks for Author):

In their revised study the authors have made substantial changes and addressed many of the previous concerns. In my view, several of the additional experiments, including the improved in vitro co-culture system and the extended binding analyses, have strengthened the manuscript.

However, I remain concerned about the conceptual interpretation of the in vivo data, particularly regarding the choice of the xenograft model to dissect T-cell-independent macrophage effects. In their response, the authors argue that in the A375 human melanoma PBMC-reconstituted model, depletion of $CD4^+$ and $CD8^+$ T cells reduced macrophage infiltration, possibly due to incomplete immune reconstitution and the interdependence between T cells and macrophages. While this is an interesting observation, it primarily underscores the limitations of the humanized xenograft system rather than fully resolving the mechanistic discrepancy. If macrophage survival or function critically depends on T cells in this model, this suggests that the system does not adequately reflect physiological tumor-immune interactions. At the same time, the nude xenograft model is described in the response as a "straightforward" approach to study T-cell-independent macrophage reprogramming. I do not believe that this conclusion is sufficiently supported. A xenogeneic setting represents a highly artificial cross-species microenvironment. Soluble factors, receptor-ligand interactions, and cell-cell communication across the species barrier are only partially functional and incompletely characterized. While some interactions are conserved, many are probably not. Therefore, using a xenograft model to dissect the relative contribution of an immune intervention that affects both innate and adaptive compartments inevitably introduces additional confounders. Importantly, the limitations of antibody depletion or knockout strategies in syngeneic models do not logically justify the use of a xenogeneic system ,when the central question concerns tumor-stroma and immune microenvironment interactions. In my view, still syngeneic immunocompetent models would provide a more physiologically relevant framework to quantify the relative contributions of T cells and macrophages. I believe the data presented are potentially important. However, the manuscript should more explicitly and critically discuss the limitations of the xenograft system. Rather than framing the nude model as a straightforward solution, its artificial nature and the potential alterations in immune composition and microenvironment should be acknowledged. This would substantially strengthen the scientific rigor of the study and help reconcile the differences between the A375 and osteosarcoma models.

Referee #3 (Comments on Novelty/Model System for Author):

The revised manuscript addresses the concerns regarding the adequacy of the model system.

Referee #3 (Remarks for Author):

The addition of in vitro experiments with PBMCs and the PLA studies are significant and add value to the manuscript. These address the major concerns that I had with the original manuscript.

Referee #1 (Remarks for Author):

The authors have addressed several of the concerns raised and have strengthened the manuscript accordingly. The revised version improves transparency regarding the experimental procedures, confirms key findings through the inclusion of complementary technological approaches, demonstrates that the described effect is Fc-independent and incorporates more representative model systems, including selected experiments using a co-culture system with tumor cells and PBMCs. Together, these additions increase confidence in the validity of the central observations.

Thank you for your insightful comments.

While the manuscript has improved substantially, there are some remaining mechanistic aspects that are not fully addressed, and certain findings would benefit from further validation in more physiologically relevant systems. Greater clarification of the direct downstream signaling of Nrp2 in macrophages, as well as a more detailed investigation of CD3L1 expression levels on tumor cells and macrophages to better define the relative contribution of cis versus trans signaling and whether both modes of interaction can occur and induce comparable functional effects, would further strengthen the mechanistic framework. Furthermore, the link between the reported immune modulation and tumor control in T cell-deficient settings remain insufficiently explained.

We deeply appreciate your constructive comments. Accordingly, we have revised our manuscript in 3 sections:

1. Greater clarification of the direct downstream signaling of NRP2 in macrophages:

Previous studies have suggested that in macrophages, NRP2 is highly correlated with NF-κB and MAPK-ERK signaling pathways (Adan S. Soloff et al., 2024; Jin, K. et al., 2023; Vega M.A. et al., 2011). Based on these findings, we performed WB to assess NRP2 downstream signaling in macrophages, using the phosphorylation of NF-κB p65 (Ser536) and ERK as pathway representatives. We again employed the induced macrophage-tumor cell co-culture system and used CD45 microbeads to isolate macrophages. Our results showed that in the siRNA-control group, anti-CD3L1 significantly upregulated NF-κB and ERK phosphorylation; these effects were reversed by NRP2 protein **(New Fig.EV5E)**. After NRP2 knockdown, the signaling changes were abolished and the antibody had no significant impact, indicating that disruption of CD3L1-NRP2 axis can result in changes of the direct NRP2 downstream signaling.

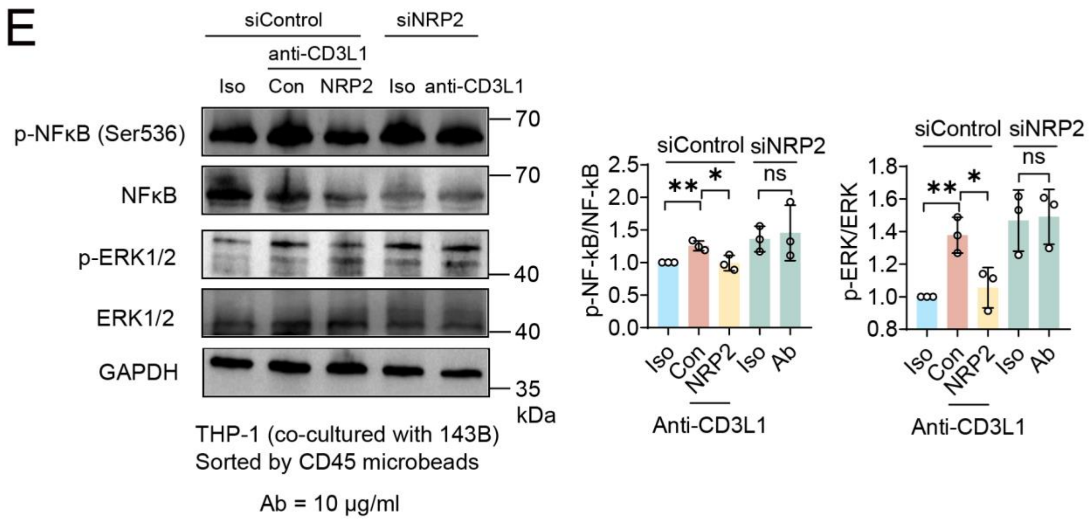

(**New Fig.EV5E**, Representative immunoblot images showing the changes of downstream NRP2 signaling and the correlation with CD3L1-NRP2 axis regulation).

We have also revised the respective manuscript text and figure legends.

Previous studies have demonstrated that in macrophages, NRP2 exhibits strong correlations with NF-κB and MAPK-ERK signaling pathways. Specifically, NRP2 activation has been shown to downregulate both NF-κB and MAPK-ERK pathways, thereby suppressing M1 macrophage polarization (66-70). Given that evaluating downstream NRP2 signaling provides a more direct reflection of the regulatory effects mediated by the CD3L1-NRP2 axis on macrophage function, we selected NF-κB p65 and ERK1/2 as representative markers of these pathways. We assessed the phosphorylation status of NF-κB p65 (Ser536) and ERK1/2 (Thr202/Tyr204) to investigate the impact of anti-CD3L1 treatment (**Fig.EV5E**). Our results indicated that anti-CD3L1 significantly counteracts the inhibitory effects of NRP2 on downstream signaling, an effect that could be restored by exogenous NRP2 protein supplementation. Conversely, NRP2 knockdown attenuated the regulatory effects of anti-CD3L1. These findings further elucidate the direct modulation of TAM polarization by anti-CD3L1 through the CD3L1-NRP2 signaling axis.

(The revised manuscript).

2. More detailed investigation of CD3L1 levels on tumor cells and macrophages:

This is indeed a very interesting topic. According to your suggestion, we assessed CD3L1 expression in 143B tumor cells, induced THP-1 cells, THP-1 cells and induced PBMCs. Notably, although THP-1, as a tumor-derived cell line, exhibited higher CD3L1 expression

than PBMC, its expression was substantially lower than that in 143B tumor cells. This disparity implies that within the TME, the trans signaling between CD3L1 on tumor cells and NRP2 on macrophages, may likely predominate over cis signaling, which was also consistent with our proposed mechanism. Further analysis (e.g. spatiotemporal transcriptomics of patient samples) should be interesting to investigate, and the results of systemic analysis of CD3L1 expression in different TME will be revealed in our following study.

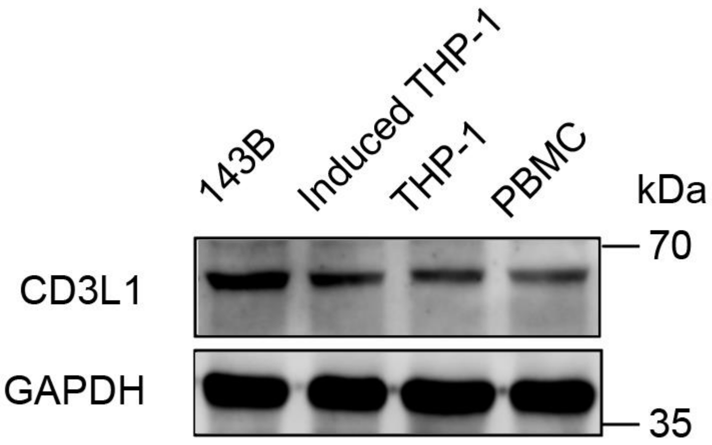

(Representative immunoblot results showing the CD3L1 expression of 143B tumor cells, THP-1 cells and induced PBMC).

3. A sufficiently explained link between the CD3L1-NRP2-TAM modulation and tumor control in T cell-deficient settings:

We have revised **the Discussion section** to incorporate a more comprehensive rationale and a more detailed explanation of the connection between CD3L1-NRP2-TAM regulation and the immunotherapy effect in the TME. We are also actively conducting new model studies focused on the modulation of macrophages or other immune subsets alone, the results of which will be disclosed in future studies, along with certain novel mechanistic and clinical findings we have uncovered.

In T cell-deficient settings, our study provides a scientifically rigorous explanation for the pivotal link between CD3L1-NRP2-TAM modulation and effective tumor control. Single-cell analysis revealed that macrophages constitute the most significantly altered cell population in the treatment group. Among $CD45^{+}$ immune cells, macrophages were the predominant subset, indicating their central role in the immune response within the tumor microenvironment. Further, based on previous studies, macrophages function as the main regulators of the remaining immune cells, orchestrating their activation, differentiation, and functional polarization (73-75). This regulatory capacity positions macrophages as critical mediators of the anti-tumor immune response, especially in settings where T cell function is compromised. Additionally, given the well-established association between M2 macrophage polarization and adverse clinical outcomes in cancer, strategies aimed at reprogramming these macrophages towards a pro-inflammatory phenotype emerge as promising therapeutic avenues (31, 75-78). Currently, we are actively engaged in model experiments designed to independently modulate macrophages alongside other immune cell types, with the aim of dissecting their specific contributions to tumor suppression; the results of these ongoing investigations will be detailed in forthcoming reports, ultimately aiming at overcoming the challenges posed by T cell deficiency in clinical cancer treatment.

(A more detailed discussion about the correlation between CD3L1-NRP2-TAM modulation and immunotherapy effect in **the Discussion section**).

We sincerely hope these revisions can address your concerns.

Overall, the manuscript presents interesting and potentially novel findings and proposes a compelling dual mechanism for a promising new ICB agent. The study highlights several novel aspects of this compound, identifies Nrp2 as a binding partner for CD3L1, and provides evidence supporting the relevance of this interaction in driving the observed phenotypic switch in macrophages. A clear limitation of the study is that certain mechanistic aspects remain incompletely resolved and the translational robustness would benefit from broader validation in more physiologically relevant systems. These aspects warrant consideration when interpreting the findings and evaluating their broader applicability.

Thank you once again for all of your efforts and comments. We have now provided more robust evidence to support our conclusion in accordance with your constructive suggestions.

Referee #2 (Comments on Novelty/Model System for Author):

The revised study presents a potentially novel and conceptually interesting interaction between CD3L1 and NRP2, supported by several additional experiments that have strengthened study compared to the original submission. The in vitro co-culture system as well as the more detailed binding analyses, support the proposed macrophage-directed mechanism.

My remaining concern relates to the reliance on xenogeneic models to dissect T-cell-independent macrophage effects. In my view, such systems introduce inherent biological limitations due to the artificial cross-species microenvironment, which may affect tumor-stroma and immune interactions. While I do not consider it necessary to request additional experiments at this stage, the manuscript should more clearly and explicitly acknowledge these limitations and temper the interpretation accordingly

We deeply appreciate your constructive feedback. We have now greatly revised **the Discussion part** to elucidate the limitations and the necessity to conduct further studies.

Referee #2 (Remarks for Author):

In their revised study the authors have made substantial changes and addressed many of the previous concerns. In my view, several of the additional experiments, including the improved in vitro co-culture system and the extended binding analyses, have strengthened the manuscript.

However, I remain concerned about the conceptual interpretation of the in vivo data, particularly regarding the choice of the xenograft model to dissect T-cell-independent macrophage effects. In their response, the authors argue that in the A375 human melanoma PBMC-reconstituted model, depletion of $CD4^+$ and $CD8^+$ T cells reduced macrophage infiltration, possibly due to incomplete immune reconstitution and the interdependence between T cells and macrophages. While this is an interesting observation, it primarily underscores the limitations of the humanized xenograft system rather than fully resolving the mechanistic discrepancy. If macrophage survival or function critically depends on T cells in this model, this suggests that the system does not adequately reflect physiological tumor-immune interactions. At the same time, the nude xenograft model is described in the response as a "straightforward" approach to study T-cell-independent macrophage reprogramming. I do not believe that this conclusion is sufficiently supported. A xenogeneic setting represents a highly artificial cross-species microenvironment. Soluble factors, receptor-ligand interactions, and cell-cell

communication across the species barrier are only partially functional and incompletely characterized. While some interactions are conserved, many are probably not. Therefore, using a xenograft model to dissect the relative contribution of an immune intervention that affects both innate and adaptive compartments inevitably introduces additional confounders. Importantly, the limitations of antibody depletion or knockout strategies in syngeneic models do not logically justify the use of a xenogeneic system ,when the central question concerns tumor-stroma and immune microenvironment interactions. In my view, still syngeneic immunocompetent models would provide a more physiologically relevant framework to quantify the relative contributions of T cells and macrophages. I believe the data presented are potentially important. However, the manuscript should more explicitly and critically discuss the limitations of the xenograft system. Rather than framing the nude model as a straightforward solution, its artificial nature and the potential alterations in immune composition and microenvironment should be acknowledged. This would substantially strengthen the scientific rigor of the study and help reconcile the differences between the A375 and osteosarcoma models.

We sincerely appreciate your thoughtful critique of our conceptual interpretation of the *in vivo* data, particularly regarding the limitations of xenograft models in dissecting T cell-independent macrophage effects. We fully agree that neither the A375 human melanoma PBMC-reconstituted model nor the nude xenograft system can perfectly mimic the physiological TME. As you correctly pointed out, the reduced macrophage infiltration observed upon CD4+/CD8+ T cell depletion in the humanized model likely reflects incomplete immune reconstitution and bidirectional crosstalk between T cells and macrophages, rather than a definitive mechanistic insight. Similarly, while the nude model was initially described as a "straightforward" approach to study macrophage reprogramming, we recognize that xenogeneic settings introduce artificial confounders—such as species-specific variations in soluble factors, receptor-ligand interactions, and cell-cell communication—that may alter immune cell composition and function. These limitations, as you noted, compromise the translational relevance of xenograft models when addressing questions about tumor-stroma and immune microenvironment interactions, particularly for interventions affecting both innate and adaptive immunity.

We wholeheartedly concur that syngeneic immunocompetent models represent a more physiologically relevant framework to quantify the relative contributions of T cells and macrophages. In response to this critique, we have significantly expanded the Discussion section to critically evaluate the limitations of our xenograft systems, explicitly acknowledging their artificial nature and potential distortions of immune dynamics. Rather than framing the nude model as a definitive solution, we now emphasize its role as a preliminary exploratory tool while highlighting the need for caution in interpreting results

due to cross-species incompatibilities.

Furthermore, we are actively addressing these limitations in ongoing studies involving CD3L1 blockade, which include multiple syngeneic tumor models (e.g., LLC, MC38 and B16-F10) to better capture physiological immune interactions. These experiments will provide a more robust platform to dissect macrophage-dependent mechanisms and their interplay with different immune subsets, will be disclosed in our subsequent studies (along with certain novel mechanistic and clinical findings we have uncovered).

However, it is important to acknowledge the limitations of the nude mouse model, which, despite its utility in initial investigations of CD3L1-NRP2-mediated macrophage modulation, introduces confounding factors that undermine its physiological relevance and translational applicability. The absence of functional adaptive immunity in nude mice—specifically, the lack of mature T and B cells—creates an artificial environment where macrophage behavior may be influenced by compensatory mechanisms or altered immune-stromal interactions that do not occur in immunocompetent hosts. For instance, in PBMC-reconstituted models, human immune cells must interact with murine stromal components, leading to potential mismatches in cytokine responsiveness, chemokine gradients, or adhesion molecule expression, which could skew macrophage recruitment, polarization, or survival independently of CD3L1-NRP2 signaling. These species-specific discrepancies are compounded by incomplete characterization of cross-species receptor-ligand interactions, such as human CD3L1 binding to murine NRP2, which may exhibit altered affinity or downstream signaling compared to human-human interactions, thereby introducing unquantified variability into experimental outcomes.

The interpretation of findings, such as the observed reduction in macrophage infiltration following $CD4^{+}/CD8^{+}$ T-cell depletion in A375 tumors, should be careful in this context, as it may reflect incomplete immune reconstitution, dysregulated cytokine networks, and artificial synergies between T cells and macrophages in xenogeneic settings rather than a direct regulatory mechanism. Similarly, the inconsistent reliance on TAMs between melanoma and osteosarcoma models underscores how tumor type-specific factors—such as stromal composition, baseline inflammation, or intrinsic immunogenicity—can interact with model-inherent artifacts to produce divergent results, further complicating efforts to isolate CD3L1-NRP2-specific effects.

Collectively, these limitations suggest that while CD3L1-NRP2 may influence macrophage function in xenograft models, the artificiality of these systems precludes definitive conclusions about its role in human tumors, particularly regarding T-cell-independent mechanisms. To address these gaps, future studies should prioritize syngeneic models with intact immune systems, which can better replicate human immune dynamics. Additionally, more thorough mechanistic dissections are essential to distinguish direct CD3L1-NRP2 effects from indirect confounders. Until such rigorous validations are performed, conclusions drawn from xenogeneic models should be interpreted with caution, emphasizing the need for translational research that bridges preclinical discoveries to clinically relevant contexts.

(**New Discussion section** addressing the limitations of the applied models and the

necessity for further syngeneic model).

We thank you again for helping us to strengthen the scientific rigor of our work. Your insights have been invaluable in refining our discussion of model limitations and ensuring that our conclusions are interpreted with appropriate caution. We sincerely hope that the revisions can address your concerns properly.

Referee #3 (Comments on Novelty/Model System for Author):

The revised manuscript addresses the concerns regarding the adequacy of the model system.

Thank you.

Referee #3 (Remarks for Author):

The addition of in vitro experiments with PBMCs and the PLA studies are significant and add value to the manuscript. These address the major concerns that I had with the original manuscript.

Thank you once again for your constructive feedback, which greatly helped us improve the quality of our work.

21st Apr 2026

Dear Dr. Xu,

Thank you for submitting your revised study, and please accept my apologies for the delay in getting back to you. Referee #1 has evaluated your revised manuscript and is satisfied with the revisions. We will therefore be able to accept your manuscript once the following minor editorial matters are addressed:

1/ Manuscript text:
- Please remove the red font text and only keep in track changes mode any new modification.
- Please correct the order and headings of the sections in the manuscript text to: Abstract / The Paper Explained / Introduction / Results / Discussion / Methods / Data Availability / Acknowledgements / Disclosure and Competing Interests Statement / References / Figure Legends / Expanded View Figure Legends
- Remove the sections " Lead contact" and "Materials Availability".
- Methods:
o Cell lines: please indicate origin, and whether the cells were authenticated and tested for mycoplasma contamination.
o Animals: please describe origin, housing and husbandry conditions.
o Human samples: please include a statement confirming that the experiments conformed to the principles set out in the WMA Declaration of Helsinki and the Department of Health and Human Services Belmont Report.
o Antibodies: please provide dilutions / concentrations.
o Check the paragraph 'BMDM harvest and induction', as the style is different from the rest of the methods.
o Statistics: please move statistical test and p values to the respective legends. Please include a statement about sample size and exclusion/inclusion criteria, and adjust the checklist accordingly.
o If you used an image data base for scientific iconography (e.g., BioRender), indicate it at the end of the Methods section, and let us know if you have a license that allows for publication in an academic journal.
- Data Availability: remove "This paper does not report original code" and "Any additional information required to reanalyze the data reported in this paper is available from the lead contact upon request". Please provide a link to the deposited datasets and note that they must be public before acceptance of the manuscript.
- Please rename "Competing interest" to "Disclosure and competing interests statement". Mention that Rui Wang works for BioTroy Therapeutics.
- References should be corrected to alphabetical order, with 10 author names listed before et al.

2/ Figures:
- Table EV1 should be renamed Dataset EV1 and the other EV tables should be renumbered. Please adjust the Reagent table accordingly. All table legends should be removed from the manuscript text and added to the corresponding tables.
- Please note that figure reuse is allowed, but should be justified and mentioned in the figure legends (i.e. Figure 2F and Figure EV2C)
- Please address the queries from our data editors in the figure legends:
1. Please define the annotated p values ****/***/**/* as well as provide the exact p-values for the same in the legend of figure 1D, E; 3B, C, D, E, G, H; 4A-D; 5D-G; 6B-D; EV4 D as appropriate.
2. Please indicate the statistical test used for data analysis in the legends of figures 1D, E; 2K, L; 3B, C, D, E, G, H; 4A-D; 5D, E, F, G; 6B-D; EV1 A-C, D-I; EV3 A-F; EV4 D
3. Please note that information related to n is missing in the legends of figures 2J; EV2 G, J.
4. Please note that the error bars are not defined in the legends of figures 1D, E; 3B, C, D, E, G, H, I; 4A-D; 5A, B, D, E, F, G; 6B-D; EV4 C, D; EV5 A, D; EV6 A, B
5. Please note that the scale bar needs to be defined for figure EV6 C

3/ Thank you for providing source data. Please also provide the facs raw data and the western blot replicates.

4/ I introduced minor modifications to The paper explained, please let me know if you agree or amend as you see fit:
Problem
Incomplete characterization of the immune evasion mechanisms within the tumor microenvironment (TME) continues to limit the efficacy of immune checkpoint blockade (ICB) therapies.
Results
This study identifies CD3L1 (ITPRIPL1) as a dual immunoregulatory molecule that facilitates tumor immune evasion by modulating both T cell and macrophage functions. Targeting CD3L1 with a monoclonal antibody demonstrated potent antitumor activity in preclinical models and in patients, unexpectedly activating TAMs within the TME beyond its anticipated role in T-cell reactivation. Mechanistic studies revealed that CD3L1 interacts with neuropilin-2 (NRP2) on macrophages, driving the polarization of immunosuppressive TAMs. In T-cell-deficient osteosarcoma models, anti-CD3L1 treatment reprogrammed TAMs toward an anti-tumor phenotype, suppressing tumor progression. Clinical data corroborated these findings, showing deep TME remodeling in advanced solid tumors, with reduced immunosuppresive TAM infiltration and enhanced T cell activation. Furthermore, the modulation of TAMs by anti-CD3L1 was found to be Fc-independent, highlighting the direct role of CD3L1-

NRP2 interaction in macrophage polarization
Impact
These findings contribute to a deeper understanding of the pivotal role of CD3L1 in immune evasion within the TME and provide valuable insights for guiding anti-CD3L1 treatment in clinical trials.

5/ I introduced minor modifications in your synopsis, please let me know if you agree or amend as you see fit. Please also remove it from the manuscript and upload it as a separate file.

CD3L1 targeting is proposed as a bifunctional strategy to overcome immune evasion, offering a promising avenue for the treatment of immunotherapy-resistant malignancies and guiding the clinical development of anti-CD3L1 therapies.
- Targeting CD3L1 disrupts its interaction with NRP2, reprogramming TAMs toward an immunostimulatory phenotype while revitalizing T-cell effector functions-even in T-cell sparse tumors like osteosarcoma.
- In an ongoing Phase I trial, anti-CD3L1 monotherapy induced striking TME remodeling in PD-1 refractory tumors, with concomitant T-cell activation and macrophage infiltration correlating with therapeutic efficacy.
- Integrated multi-omics and pan-cancer analyses reveal that CD3L1 overexpression predicts poor prognosis and is tightly linked to M2 TAM dominance across solid tumors.

Please also suggest a visual abstract to illustrate your article as a PNG file 550 px wide x 300 px high.

6/ As part of the EMBO Publications transparent editorial process initiative (see our Editorial at http://embomolmed.embopress.org/content/2/9/329), EMBO Molecular Medicine will publish online a Review Process File (RPF) to accompany accepted manuscripts.
This file will be published in conjunction with your paper and will include the anonymous referee reports, your point-by-point response and all pertinent correspondence relating to the manuscript. Let us know whether you agree with the publication of the RPF and as here, if you want to remove or not any figures from it prior to publication.
Please note that the Authors checklist will be published at the end of the RPF.

I look forward to receiving your revised manuscript.

Yours sincerely,

Lise Roth

Lise Roth, PhD
Senior Editor
EMBO Molecular Medicine

***** Reviewer's comments *****

Referee #1 (Comments on Novelty/Model System for Author):

The addition of further experiments dissecting the molecular mechanism of the CD3L1-NRP2 axis has overall strengthened the manuscript and provides further evidence for a direct link between targeting this axis and the observed phenotype switching. While the model system used has limitations, the expanded discussion in the revised manuscript clearly outlines these constraints and emphasizes that the findings should be interpreted with appropriate caution, which is valuable.

Referee #1 (Remarks for Author):

The inclusion of further in vitro experiments dissecting the downstream effects of targeting the CD3L1-NRP2 axis enhances the mechanistic understanding and provides additional evidence for a direct association with the observed phenotype switching. Together with the expanded discussion of the underlying mechanisms of the observed therapeutic effects and the clearly stated limitations of the model system, the revised version largely addresses the remaining concerns.

All minor editorial requests have been addressed by the authors.

7th May 2026

Dear Dr. Xu,

Thank you for submitting your revised manuscript. I am pleased to inform you that your manuscript is accepted for publication and is now being sent to our publisher to be included in the next available issue of EMBO Molecular Medicine!

Please note that deposited datasets must be public before online publication.

Your manuscript will be processed for publication by EMBO Press. It will be copy edited and you will receive page proofs prior to publication. Please note that you will be contacted by Springer Nature Author Services to complete licensing and payment information.

You may qualify for financial assistance for your publication charges - either via a Springer Nature fully open access agreement or an EMBO initiative. Check your eligibility: https://link.springer.com/journal/44321/how-to-publish-with-us

Should you be planning a Press Release on your article, please get in contact with embo_production@springernature.com as early as possible in order to coordinate publication and release dates.

If you have any questions, please do not hesitate to contact the Editorial Office. Thank you for your contribution to EMBO Molecular Medicine.

Yours sincerely,

Lise Roth

Lise Roth, Ph.D
Senior Editor
EMBO Molecular Medicine

>>> Please note that it is EMBO Molecular Medicine policy for the transcript of the editorial process (containing referee reports and your response letter) to be published as an online supplement to each paper. If you do NOT want this, you will need to inform the Editorial Office via email immediately. More information is available here: https://link.springer.com/partners/embo-press/editorial-policies#Peer%20review

EMBO Press Author Checklist

Corresponding Author Name: Jie Xu, Shouyan Deng	
Journal Submitted to: EMBO Molecular Medicine	
Manuscript Number: EMM-2025-22767-T	

USEFUL LINKS FOR COMPLETING THIS FORM
The EMBO Journal - Author Guidelines
EMBO Reports - Author Guidelines
Molecular Systems Biology - Author Guidelines
EMBO Molecular Medicine - Author Guidelines

Reporting Checklist for Life Science Articles (updated January
This checklist is adapted from Materials Design Analysis Reporting (MDAR) Checklist for Authors. MDAR establishes a minimum set of requirements in transparent reporting in the life sciences (see Statement of Task: 10.31222/osf.io/9sm4x). Please follow the journal's guidelines in preparing your
Please note that a copy of this checklist will be published alongside your article.

Abridged guidelines for figures
1. Data
The data shown in figures should satisfy the following conditions:
- the data were obtained and processed according to the field's best practice and are presented to reflect the results of the experiments in an accurate and unbiased manner.
- ideally, figure panels should include only measurements that are directly comparable to each other and obtained with the same assay.
- plots include clearly labeled error bars for independent experiments and sample sizes. Unless justified, error bars should not be shown for technical
- if n<5, the individual data points from each experiment should be plotted. Any statistical test employed should be justified.
- Source Data should be included to report the data underlying figures according to the guidelines set out in the authorship guidelines on Data

2. Captions
Each figure caption should contain the following information, for each panel where they are relevant:
- a specification of the experimental system investigated (eg cell line, species name).
- the assay(s) and method(s) used to carry out the reported observations and measurements.
- an explicit mention of the biological and chemical entity(ies) that are being measured.
- an explicit mention of the biological and chemical entity(ies) that are altered/varied/perturbed in a controlled manner.
- the exact sample size (n) for each experimental group/condition, given as a number, not a range;
- a description of the sample collection allowing the reader to understand whether the samples represent technical or biological replicates (including how many animals, litters, cultures, etc.).
- a statement of how many times the experiment shown was independently replicated in the laboratory.
- definitions of statistical methods and measures:
  - common tests, such as t-test (please specify whether paired vs. unpaired), simple χ2 tests, Wilcoxon and Mann-Whitney tests, can be unambiguously identified by name only, but more complex techniques should be described in the methods section;
  - are tests one-sided or two-sided?
  - are there adjustments for multiple comparisons?
  - exact statistical test results, e.g., P values = x but not P values < x;
  - definition of 'center values' as median or average;
  - definition of error bars as s.d. or s.e.m.

Please complete ALL of the questions below.
Select "Not Applicable" only when the requested information is not relevant for your study.

Materials

Newly Created Materials	Information included in the manuscript?	In which section is the information available? (Reagents and Tools Table, Materials and Methods, Figures, Data Availability Section)
New materials and reagents need to be available; do any restrictions apply?	Yes	Regants and Tools Table

Antibodies	Information included in the manuscript?	In which section is the information available? (Reagents and Tools Table, Materials and Methods, Figures, Data Availability Section)
For **antibodies** provide the following information:<br>- Commercial antibodies: RRID (if possible) or supplier name, catalogue number and or/clone number<br>- Non-commercial: RRID or citation	Yes	Regants and Tools Table

DNA and RNA sequences	Information included in the manuscript?	In which section is the information available? (Reagents and Tools Table, Materials and Methods, Figures, Data Availability Section)
**Short novel DNA or RNA including primers, probes**: provide the sequences.	Yes	EV tables

Cell materials	Information included in the manuscript?	In which section is the information available? (Reagents and Tools Table, Materials and Methods, Figures, Data Availability Section)
**Cell lines:** Provide species information, strain. Provide accession number in repository **OR** supplier name, catalog number, clone number, and/**OR** RRID.	Yes	Regants and Tools Table
**Primary cultures:** Provide species, strain, sex of origin, genetic modification status.	Yes	Regants and Tools Table
Report if the cell lines were recently **authenticated** (e.g., by STR profiling) and tested for mycoplasma contamination.	Yes	Regants and Tools Table

Experimental animals	Information included in the manuscript?	In which section is the information available? (Reagents and Tools Table, Materials and Methods, Figures, Data Availability Section)
**Laboratory animals or Model organisms:** Provide species, strain, sex, age, genetic modification status. Provide accession number in repository **OR** supplier name, catalog number, clone number, **OR** RRID.	Yes	Regants and Tools Table
**Animal observed in or captured from the field:** Provide species, sex, and age where possible.	Not Applicable	
Please detail **housing and husbandry conditions**.	Yes	Materials and Methods

Plants and microbes	Information included in the manuscript?	In which section is the information available? (Reagents and Tools Table, Materials and Methods, Figures, Data Availability Section)
**Plants:** provide species and strain, ecotype and cultivar where relevant, unique accession number if available, and source (including location for collected wild specimens).	Not Applicable	
**Microbes:** provide species and strain, unique accession number if available, and source.	Not Applicable	

Human research participants	Information included in the manuscript?	In which section is the information available? (Reagents and Tools Table, Materials and Methods, Figures, Data Availability Section)
If collected and within the bounds of privacy constraints report on age, sex and gender or ethnicity for all study participants.	Yes	Materials and Methods

Core facilities	Information included in the manuscript?	In which section is the information available? (Reagents and Tools Table, Materials and Methods, Figures, Data Availability Section)
If your work benefited from core facilities, was their service mentioned in the acknowledgments section?	Not Applicable	

Design

**Study protocol**	**Information included in the manuscript?**	**In which section is the information available?** (Reagents and Tools Table, Materials and Methods, Figures, Data Availability Section)
If study protocol has been **pre-registered, provide DOI in the manuscript**. For clinical trials, provide the trial registration number **OR** cite DOI.	Not Applicable	
Report the **clinical trial registration number** (at ClinicalTrials.gov or equivalent), where applicable.	Yes	Materials and Methods

**Laboratory protocol**	**Information included in the manuscript?**	**In which section is the information available?** (Reagents and Tools Table, Materials and Methods, Figures, Data Availability Section)
Provide DOI OR other citation details if **external detailed step-by-step protocols** are available.	Not Applicable	

**Experimental study design and statistics**	**Information included in the manuscript?**	**In which section is the information available?** (Reagents and Tools Table, Materials and Methods, Figures, Data Availability Section)
Include a statement about **sample size** estimate even if no statistical methods were used.	Yes	Figure legends
Were any steps taken to minimize the effects of subjective bias when allocating animals/samples to treatment (e.g. **randomization procedure**)? If yes, have they been described?	Yes	Materials and Methods
Include a statement about **blinding** even if no blinding was done.	Yes	Materials and Methods
Describe **inclusion/exclusion criteria** if samples or animals were excluded from the analysis. Were the criteria pre-established?<br><br>If sample or data points were omitted from analysis, report if this was due to attrition or intentional exclusion and provide justification.	Yes	Materials and Methods
For every figure, are **statistical tests** justified as appropriate? Do the data meet the assumptions of the tests (e.g., normal distribution)? Describe any methods used to assess it. Is there an estimate of variation within each group of data? Is the variance similar between the groups that are being statistically compared?	Yes	Materials and Methods

**Sample definition and in-laboratory replication**	**Information included in the manuscript?**	**In which section is the information available?** (Reagents and Tools Table, Materials and Methods, Figures, Data Availability Section)
In the figure legends: state number of times the experiment was **replicated** in laboratory.	Yes	Figure legends
In the figure legends: define whether data describe **technical or biological replicates**.	Yes	Figure legends

Ethics

**Ethics**	**Information included in the manuscript?**	**In which section is the information available?** (Reagents and Tools Table, Materials and Methods, Figures, Data Availability Section)
Studies involving **human participants**: State details of **authority granting ethics approval** (IRB or equivalent committee(s), provide reference number for approval.	Yes	Materials and Methods
Studies involving **human participants**: Include a statement confirming that **informed consent** was obtained from all subjects and that the experiments conformed to the principles set out in the WMA Declaration of Helsinki and the Department of Health and Human Services Belmont Report.	Yes	Materials and Methods
Studies involving **human participants:** For publication of **patient photos**, include a statement confirming that consent to publish was obtained.	Not Applicable	
Studies involving experimental **animals**: State details of **authority granting ethics approval** (IRB or equivalent committee(s), provide reference number for approval. Include a statement of compliance with ethical regulations.	Yes	Materials and Methods
Studies involving **specimen and field samples:** State if relevant **permits** obtained, provide details of authority approving study; if none were required, explain why.	Not Applicable	

**Dual Use Research of Concern (DURC)**	**Information included in the manuscript?**	**In which section is the information available?** (Reagents and Tools Table, Materials and Methods, Figures, Data Availability Section)
Could your study fall under dual use research restrictions? Please check biosecurity documents and list of **select agents and toxins** (CDC): https://www.selectagents.gov/sat/list.htm	Not Applicable	
If you used a select agent, is the security level of the lab appropriate and reported in the manuscript?	Not Applicable	
If a study is subject to dual use research of concern regulations, is the name of the **authority granting approval and reference number** for the regulatory approval provided in the manuscript?	Not Applicable	

Reporting

The MDAR framework recommends adoption of discipline-specific guidelines, established and endorsed through community initiatives. Journals have their own policy about requiring specific guidelines and recommendations to complement MDAR.

**Adherence to community standards**	**Information included in the manuscript?**	**In which section is the information available?** (Reagents and Tools Table, Materials and Methods, Figures, Data Availability Section)
State if relevant guidelines or checklists (e.g., **ICMJE, MIBBI, ARRIVE, PRISMA**) have been followed or provided.	Yes	Materials and Methods
For **tumor marker prognostic studies,** we recommend that you follow the **REMARK** reporting guidelines (see link list at top right). See author guidelines, under 'Reporting Guidelines'. Please confirm you have followed these guidelines.	Not Applicable	
For **phase II and III randomized controlled trials**, please refer to the **CONSORT** flow diagram (see link list at top right) and submit the CONSORT checklist (see link list at top right) with your submission. See author guidelines, under 'Reporting Guidelines'. Please confirm you have submitted this list.	Not Applicable	

Data Availability

**Data availability**	**Information included in the manuscript?**	**In which section is the information available?** (Reagents and Tools Table, Materials and Methods, Figures, Data Availability Section)
Have **primary datasets** been deposited according to the journal's guidelines (see 'Data Deposition' section) and the respective accession numbers provided in the Data Availability Section?	Yes	Data Availability Section
Were **human clinical and genomic datasets** deposited in a public access-controlled repository in accordance to ethical obligations to the patients and to the applicable consent agreement?	Not Applicable	
Are **computational models** that are central and integral to a study available without restrictions in a machine-readable form? Were the relevant accession numbers or links provided?	Not Applicable	
If publicly available data were reused, provide the respective **data citations in the reference list**.	Not Applicable	
